# Photocatalytic Nitrene Radical Anion Generation from Sulfonyl Azides for Intermolecular Aziridination of Unactivated Alkenes

**DOI:** 10.1021/acs.joc.5c00595

**Published:** 2025-05-03

**Authors:** Dennis Dam, Joeri Schoenmakers, Elisabeth Bouwman, Jeroen D. C. Codée

**Affiliations:** Leiden Institute of Chemistry, Universiteit Leiden, 2333 CC Leiden, The Netherlands

## Abstract

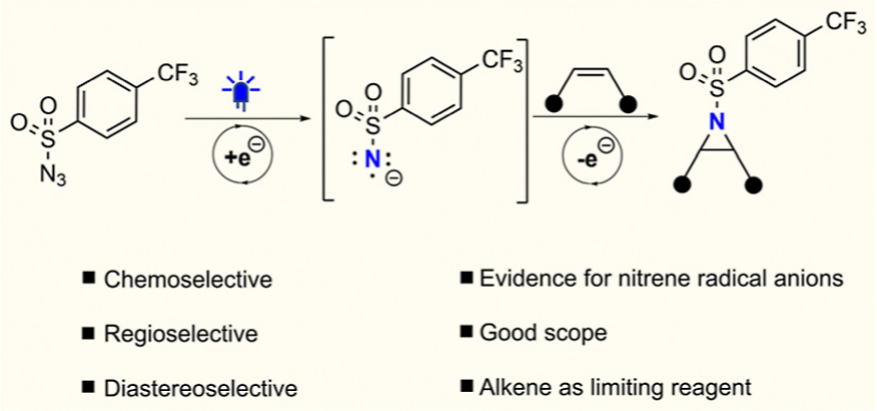

Aziridines are important structures in the contemporary organic synthesis and are used for several biological applications. Herein, we show that aziridines can be readily synthesized from alkenes by the reductive activation of sulfonyl azides, mediated by photoredox catalysis. Mechanistic studies indicate that the reaction proceeds through reactive nitrene radical anions instead of the more commonly encountered triplet nitrenes. A substrate scope is performed that showed good functional group compatibility.

## Introduction

Aziridines, three-membered heterocycles containing one nitrogen atom in the ring, are important structural elements in a variety of natural products and derivatives thereof, where they, by virtue of their ring strain, often serve as a reactive electrophile to covalently trap nucleophilic residues of their biological targets.^1−4^ They have also found widespread application as synthetic intermediates in the assembly of nitrogen-containing molecules.^5−8^ In this regard, *N*-sulfonylaziridines represent attractive moieties as the electron-withdrawing capacity of the sulfonyl group renders the aziridine an even better electrophile. In addition, phenylsulfonyl groups may serve as transient protecting groups that can be readily removed to liberate the free N–H aziridine for further modification.^9^

**Figure 1:**
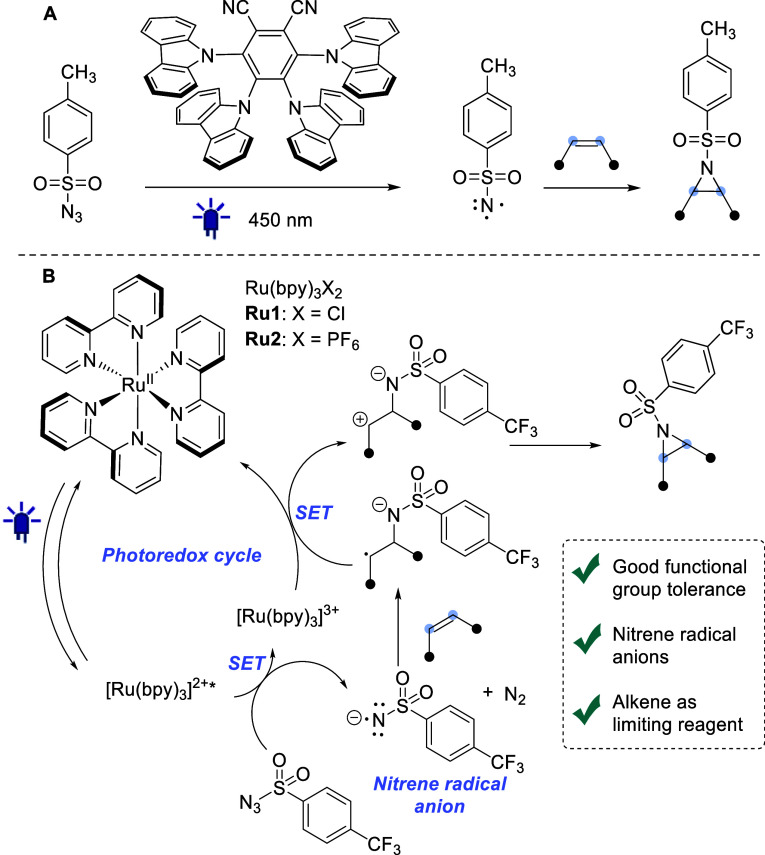
(A) Cyanoarene-photosensitized triplet nitrene-mediated aziridination. (B) Current work: nitrene radical anion-mediated aziridination through photoredox catalysis.

One of the most attractive methods for aziridine synthesis is the direct insertion of a nitrene into an alkene, which can be achieved by triplet sensitization of azide precursors, such as azidoformates or trifluoromethyl azide.^10−12^ Recently, it was reported by us and by others that excitation of a cyanoarene-based organic dye as a photosensitizer can lead to energy transfer to a sulfonyl azide to generate a triplet nitrene upon expulsion of dinitrogen, which inserts in C–C double bonds to generate an *N*-sulfonyl aziridine (Figure 1A).^13,14^ These methodologies are compatible with a wide range of complex, unactivated alkenes and tolerate the presence of a variety of functional groups. Importantly, the complex alkene substrates can be used as the limiting reagent.

However, matching the excited states of a photosensitizer and azide partner can be challenging, and often, no correlation is apparent between the triplet energy (*E*_T_) of the photocatalyst and the efficiency of the aziridination reactions. We found that unsuccessful combinations of cyanoarene-based photosensitizers and sulfonyl azides could be attributed to unproductive oxidative quenching processes, leading to the formation of nitrene radical anions and photosensitizer degradation.^13^

We reasoned that it would be advantageous to leverage the undesired oxidative quenching into a productive photoredox catalytic cycle in which the reactivity of the generated nitrene radical anions is exploited to generate aziridines from alkene substrates (Figure 1B). Nitrene radical anions are much less reactive than their one-electron oxidized nitrene equivalents in competing reactions such as hydrogen-atom transfer (HAT) and may therefore be compatible with many functional groups.^15^ Of note, generally, only triplet nitrene intermediates are considered as product-evolving intermediates in photocatalytic reactions involving sulfonyl azides,^16,17^ whereas competing SET reduction to give nitrene radical anions could in theory also be a viable reaction pathway. Our proposed strategy would therefore (1) contribute to the understanding of the reactivity of sulfonyl azides under photocatalytic conditions and (2) provide a new strategy in azide activation for the synthesis of aziridines. Encouragingly, nitrene radical anions generated from iminoiodinanes have recently been shown to be effective aziridinating agents;^18−20^ however, their use for preparative aziridine synthesis is limited as 5-fold excesses of alkenes are required and/or exhibit a limited substrate scope.

We therefore sought to develop an electron-transfer-mediated photoredox catalytic reaction by coupling a photocatalyst, having a sufficiently low *E*_T_ to prevent the generation of triplet nitrenes, with a matching sulfonyl azide for SET (Figure 1B). Herein, it is demonstrated that by judicious choice of the photocatalyst and sulfonyl azide, nitrene radical anion-mediated aziridination can be achieved. Importantly, the conditions described allow the use of alkenes bearing a range of functional groups as the limiting reagent, which positively impacts the practicality of the described method for complex alkene substrates.

## Results and Discussion

We turned to polypyridyl ruthenium complexes, as privileged photoredox catalysts,^21^ that can be excited by visible light to provide relatively long-lived excited states with the required relatively low *E*_T_ compared to the previously evaluated cyanoarene-based photosensitizers (49 versus 56–65 kcal mol^–1^).^13,22,23^ We first evaluated [Ru(bpy)_3_]Cl_2_ (**Ru1**) in the aziridination of cyclohexene in combination with *p*-toluenesulfonylazide **1**-*p*-**CH**_**3**_ in DMSO (Table 1, entry 1). The reaction provided the desired cyclohexyl aziridine in only 7% yield, as determined by NMR spectroscopy, but also led to imidation of DMSO, to give the corresponding sulfoximine alongside the formation of *para*-toluenesulfonamide. Next, DMSO was replaced with MeCN, and **Ru1** was replaced with [Ru(bpy)_3_](PF_6_)_2_ (**Ru2**, entry 2). These modifications gave aziridine **2**-*p*-**CH**_**3**_ in an increased 44% yield. The increased aziridine yield for entry 2 can be ascribed to the suppression of competitive sulfoximine formation. The counterion of the ruthenium photocatalyst (chloride for **Ru1** versus hexafluorophosphate for **Ru2**) was changed as **Ru1** was poorly soluble in MeCN. We then probed the effect of the substituents on the phenylsulfonyl azide (entries 5–12) at 1 mol % **Ru2** loading. Under these conditions, **1**-*p*-**CH**_**3**_ afforded 29% yield of aziridine **2**-*p*-**CH**_**3**_ (entry 3). Fluorophenyl sulfonyl azides delivered the aziridine product in slightly higher yield (entries 4–6), as compared with when **1**-*p*-**CH**_**3**_ was used, suggesting that electron-withdrawing groups are advantageous for the reaction. The use of CF_3_-substituted sulfonyl azides (entries 7–9) provided the aziridines in ∼40% yield, showing that the position of the withdrawing substituent on the phenyl ring did not affect the reaction. However, the presence of two *meta*-CF_3_ groups (entry 10) led to a diminished yield of the aziridine. The use of nitrophenylsulfonyl azide **1**-*p*-**NO**_**2**_ (entry 11) did not provide any aziridine, presumably due to the competing reduction of the nitro group.^24^ Methoxyphenyl sulfonyl azide (**1**-*p*-**OCH**_**3**_) gave a low yield of aziridine **2**-*p*-**OCH**_**3**_ (entry 12). Taken together, it appears that the introduction of electron-withdrawing groups is favorable for the reaction, which can be rationalized by a more facile SET from the photocatalyst to the more electron-poor sulfonyl azides to initiate the aziridination reaction.

**Table 1:**
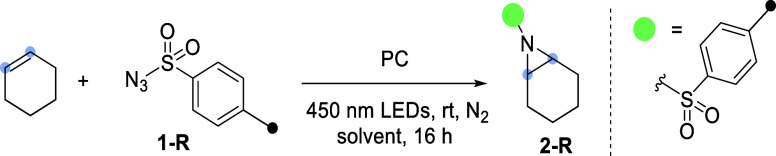
Reaction Optimization

entry	PC (mol %)	azide	yield (%)^a^
1^b^	**Ru1** (5.0)	*p*-CH_3_	7
2	**Ru2** (5.0)	*p*-CH_3_	44
3	**Ru2** (1.0)	*p*-CH_3_	29
4	**Ru2** (1.0)	*o*-F	35
5	**Ru2** (1.0)	*m*-F	41
6	**Ru2** (1.0)	*p*-F	31
7	**Ru2** (1.0)	*o*-CF_3_	40
8	**Ru2** (1.0)	*m*-CF_3_	42
9	**Ru2** (1.0)	*p*-CF_3_	42
10	**Ru2** (1.0)	*m,m*-(CF_3_)_2_	21
11	**Ru2** (1.0)	*p*-NO_2_	0
12	**Ru2** (1.0)	*p*-OMe	16
13	**Ru2** (5.0)	*p*-CF_3_	73
14^c^	**Ru2** (5.0)	*p*-CF_3_	50
15^d^	**Ru2** (5.0)	*p*-CF_3_	74
16	**Ru2** (2.0)	*p*-CF_3_	74
17^e^	**Ru2** (2.0)	*p*-CF_3_	74
18^f^	**Ru2** (2.0)	*p*-CF_3_	18^g^
19	None	*p*-CF_3_	0
20	**Ru2** (2.0)^h^	*p*-CF_3_	0

a All reactions were performed on a 0.25 mmol scale of cyclohexene with 5 equiv of azide in 0.50 mL MeCN under a N_2_ atmosphere at room temperature in a Schlenk tube unless otherwise noted. Yields were determined by ^1^H NMR spectroscopy using 1,3,5-trimethoxybenzene as an internal standard.

b DMSO was used as a solvent. Sulfoximine and sulfonamide products were observed.

c 2.5 mL of MeCN was used.

d 0.25 mL MeCN was used.

e Reaction was performed in CD_3_CN (0.25 mL) in a J Young NMR tube.

f 0.25 mL MeCN/CD_3_OD (1:1) was used.

g 1,2-amino-ether observed.

h No light irradiation.

Azide **1**-*p*-**CF**_**3**_ was selected for further studies because of the relatively good yield in the aziridination reaction and excellent solubility in virtually all organic solvents, and the precursor sulfonyl chloride is cheap and readily accessible. By increasing the catalyst loading 5-fold (entry 13), a 73% yield of the aziridine **2**-*p*-**CF**_**3**_ could be obtained. Attempts at further improving the yield by modifying the amount of solvent did not lead to a higher yield (entries 14 and 15). A catalyst loading of 2 mol % provided an optimal yield (entry 16), and it was found that the reaction could be performed in deuterated solvent in an NMR tube with the same efficiency as in a Schlenk flask (entry 17). Performing the reaction in the presence of CD_3_OD provided the aziridine in low yield as the formation of the ring-opened product cyclohexane 1,2-amino ether was observed (entry 18), showing that weakly nucleophilic solvents can react either with the product or an intermediate formed en route toward the aziridine. Control experiments (entries 19 and 20), either in the absence of light or in the absence of a photocatalyst, showed no conversion, implying the need for the excited state of **Ru2** to drive the reaction.

The observation that electron-withdrawing substituents are required for an efficient reaction suggests that the reaction may be initiated by photoinduced SET, and we further probed the mechanism with a series of experiments. Quenching of the excited state of **Ru2** by **1**-*p*-**CF**_**3**_ was investigated to construct a Stern–Volmer plot, which revealed **1**-*p*-**CF**_**3**_ to be a relatively inefficient quencher for the excited state of **Ru2** (*k*_q_ of 1.6 × 10^5^ M^–1^ s^–1^; see Supporting Information Section S3 for the plot). Azide **1**-*p*-**CH**_**3**_ proved to be an even less efficient quencher than **1**-*p*-**CF**_**3**_ as no significant quenching could be detected. The inefficient quenching observed for both sulfonyl azides suggests that there is little driving force for the photoinduced SET to occur as the rate of photoinduced electron transfer is correlated with the reaction free energy, Δ*G*, as initially reported by Rehm and Weller.^26^ We calculated the estimated Δ*G* of the proposed photoinduced SET with the simplified Rehm–Weller equation, a commonly utilized tool to assess the feasibility of photoinduced electron transfer processes^27^ (see Supporting Information Section S3 for details), and found that Δ*G* ≈ +0.27 eV ≈ +6 kcal mol^–1^. The calculation suggests that the reaction is initiated by a slightly endergonic photoinduced SET, which provides a rationalization for the low quenching rate (*k*_q_) observed in the Stern–Volmer plot. Of note, endergonic SET processes are possible when coupled to a fast and irreversible subsequent step to prevent back electron transfer.^28^ Our reaction classifies as such a reaction since dinitrogen loss from the azide following one electron reduction is irreversible, as previously corroborated by us in cyclic voltammetry experiments.^13^

**Figure 2:**
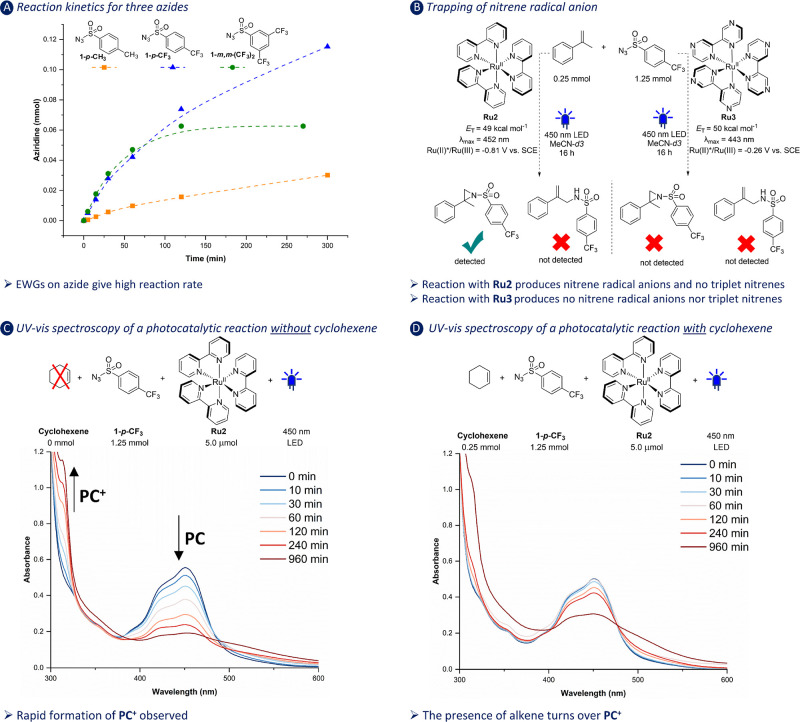
Mechanistic investigations. (A) Kinetic profile of aziridinations involving different azides. (B) Nitrene radical anion trapping using α-methylstyrene. (C) UV–vis reaction monitoring to probe the redox state of the photocatalyst in the absence of cyclohexene. (D) UV–vis reaction monitoring to probe the redox state of the photocatalyst in the presence of cyclohexene. Photophysical data taken from literature.^22,25^

Next, we investigated the kinetics of the aziridination reactions for **1**-*p*-**CH**_**3**_, **1**-*p*-**CF**_**3**_, and **1**-*m*,*m*-(**CF**_**3**_)_**2**_ using ^1^H NMR spectroscopy (Figure 2A). From the plots of product development in time, it is clear that **1**-*p*-**CF**_**3**_ and **1**-*m*,*m*-(**CF**_**3**_)_**2**_ display a similar reaction rate at the start of the reaction, while the reaction of **1**-*p*-**CH**_**3**_ is significantly slower. This outcome provides further lenience to the observation that **1**-*p*-**CH**_**3**_ is a relatively poor quencher of the excited state of **Ru2**, leading to relatively slow product formation. We previously showed that the kinetics of the triplet nitrene-mediated aziridination reactions were independent of the nature of the substituent of the sulfonyl azide.^13^ Therefore, the kinetics observed in Figure 2 in combination with the quenching data supports a SET-based mechanism.

We then performed a trapping experiment using α-methylstyrene. As described by Koenigs and co-workers, triplet sulfonyl nitrenes react with this substrate to provide a C–H amination product, while aziridines are formed upon reaction with sulfonyl nitrene radical anions.^18^ Irradiation of α-methylstyrene with **Ru2** with **1**-*p*-**CF**_**3**_ exclusively gave the aziridine product (Figure 2B), providing strong support for the nitrene radical anion involvement. Photocatalyst **Ru3** was also probed in this experiment, and this provided neither alkene aziridination nor C–H amination products; **Ru3** has similar structural and photophysical properties (λ_abs,max_ and *E*_T_, see Figure 2B) as **Ru2** but is a much weaker reductant in its excited state and therefore less likely to be oxidatively quenched by **1**-*p*-**CF**_**3**_. This indicates that a strong enough reductive potential of the photocatalyst is required to drive the aziridination reaction, an outcome that agrees with the proposed photoinduced SET from the photocatalyst to **1**-*p*-**CF**_**3**_.

Having established that the reaction likely involves nitrene radical anions that are formed upon blue-light-induced SET from **Ru2** to **1**-*p*-**CF**_**3**_, the regeneration of the oxidized photocatalyst after the SET event was investigated. Reactions in the absence and in the presence of cyclohexene were monitored with UV–vis spectroscopy (see Figure 2C,D, respectively). The reaction in the absence of cyclohexene displayed a rapid decrease in the absorption band of **Ru2** at 452 nm (Figure 2C). At the same time, an increase in a band at 314 nm was observed, which can be ascribed to the formation of oxidized **Ru2** ([Ru(bpy)_3_]^3+^). The changes in the absorption bands agree well with previously described one-electron oxidations of **Ru2** both under oxidative photochemical^29^ and oxidative spectroelectrochemical conditions.^30^ Thus, under light irradiation, **Ru2** is converted to [Ru(bpy)_3_]^3+^ in the presence of **1**-*p*-**CF**_**3**_, which takes place through a visible light-induced SET.

The presence of cyclohexene clearly retards the formation of [Ru(bpy)_3_]^3+^, and the characteristic absorption of **Ru2** decreases at a much lower rate (Figure 2D). This outcome suggests either (1) that the alkene directly reduces [Ru(bpy)_3_]^3+^ via SET or (2) that it forms a new species that reduces [Ru(bpy)_3_]^3+^. Option (1) is deemed unlikely as [Ru(bpy)_3_]^3+^ is not strong enough of an oxidant (*E*_1/2_^III/II^ = +1.29 V vs SCE in MeCN)^25^ to directly oxidize cyclohexene (*E*_1/2_^OX^ = +1.47 V vs SCE in MeCN).^31^ It is likely that pathway (2) is operative (shown in Figure 1B): the nitrene radical anion that is formed after SET reacts with the cyclohexene to form a carbon-centered radical, which then reduces [Ru(bpy)_3_]^3+^ to **Ru2**. The regeneration of **Ru2** via this pathway is deemed feasible as secondary carbon-centered radicals are readily oxidized to the carbocation by [Ru(bpy)_3_]^3+^.^32^ The reacted substrate is then poised for a ring-closing reaction to give the aziridine product.

**Figure 3:**
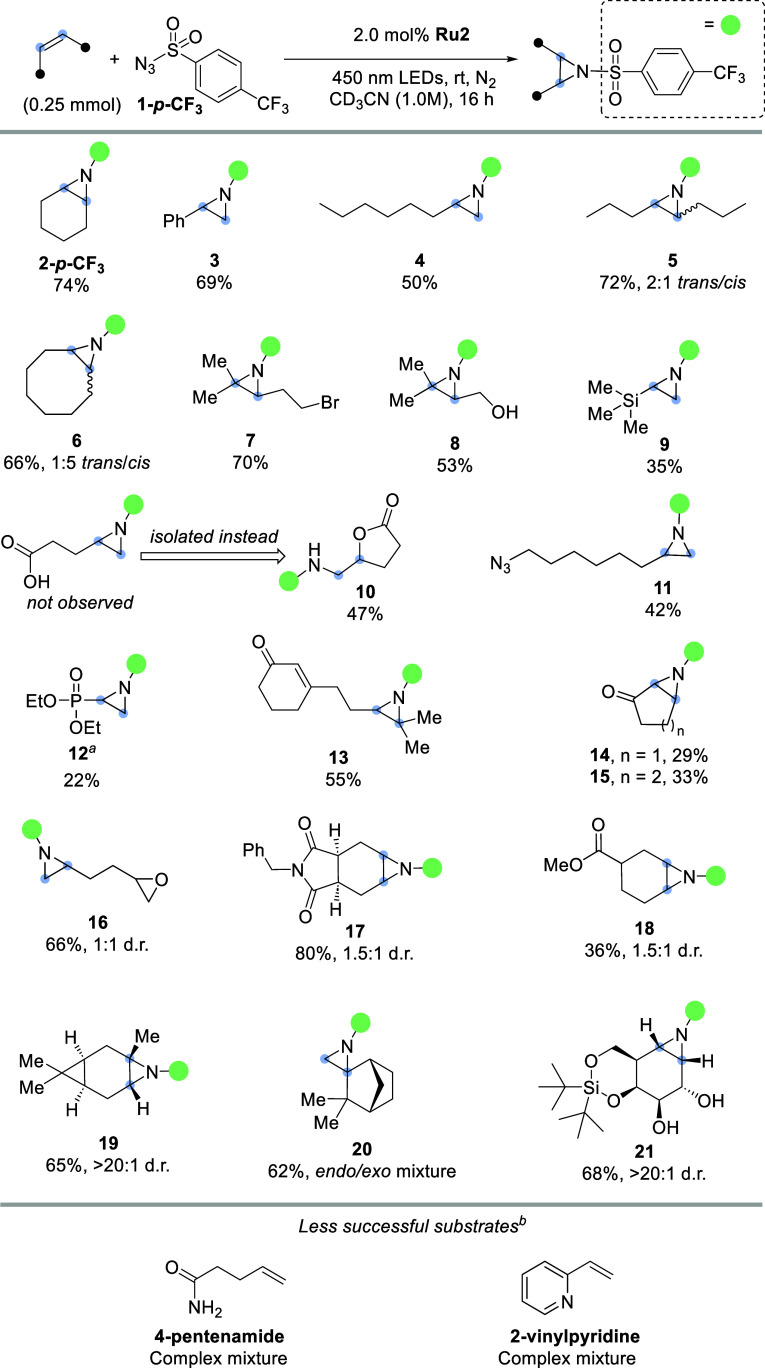
Substrate scope. Reaction conditions: 0.25 mmol alkene, 1.25 mmol **1**-*p*-**CF**_**3**_, and 5.0 μmol **Ru2** in 0.25 mL CD_3_CN under blue light irradiation for 16 h in a dinitrogen atmosphere. ^*a*^Reaction performed with 5 mol % **Ru2**. ^*b*^Details in Supporting Information Section S8.

Next, the substrate scope of the reaction was evaluated, as summarized in Figure 3. The optimized reaction conditions were used on different alkenes in an airtight J. Young NMR tube. First, simple aliphatic alkenes were evaluated, and they smoothly afforded the corresponding aziridines **2**-*p*-**CF**_**3**_, **3**, and **4**. When *cis*-4-octene was subjected to the aziridination conditions, a diastereomeric mixture (2:1 *trans*/*cis*) of aziridines **5** was obtained. Similarly, *cis*-cyclooctene gave a 66% yield of 1:5 *trans*/*cis* aziridines **6**.

The presence of a bromide is tolerated under the reaction conditions, and aziridine **7** could be obtained in 70% yield. Free hydroxyl functionalities are also compatible with the aziridination reaction, and aziridine **8** was formed in 50% yield. Along the same vein, a vinylsilane gave aziridine **9**. The reaction with 4-pentenoic acid showed no sign of the aziridine product, but instead lactone **10** was formed, which could be isolated in 47% yield. Product **10** can originate from either the ring closure of the zwitterionic intermediate that is formed prior to aziridine formation or the pendent acid that can open the aziridine ring through a protonation-nucleophilic attack sequence. Whatever the exact reaction mechanism, the result shows that carboxylic acids do not interfere with the catalytic cycle. The presence of an aliphatic azide was tolerated as aziridine **11** could be obtained. The absence of competing nitrene and/or nitrene radical anion formation from the aliphatic azide is likely a result of the relatively high *E*_T_ and low reduction potential of such alkyl azides.^33^

Next, we attempted diethyl vinylphosphonate using the optimized conditions but only found traces of the intended aziridine **12** in the crude mixture. We expected phosphonates to be compatible with our photocatalytic reaction and concluded that the low yield could originate from the decreased nucleophilicity of this alkene. Increasing the catalyst loading to 5 mol % supports this hypothesis as aziridine **12** could be isolated in an increased 22% yield. The sluggish conversion of the vinyl phosphonate further revealed that the nitrene radical anions formed in this study behave as electrophilic species at nitrogen.

The reaction to give aziridine **12** suggests that electrophilic alkenes react less readily compared to more nucleophilic alkenes; thus, nitrene radical anions may allow regioselective aziridination reactions. We demonstrated the potential for such regioselective transformations with the formation of aziridine **13**, which was formed with excellent selectivity with respect to the electron-poor α,β-unsaturated ketone. It should be noted that such regioselective reactions are likely to succeed only in substrates that exhibit a very pronounced difference in the relative nucleophilicity of the alkene partners as simple alkyl-substituted terminal and internal alkenes show no significant difference in reaction rates. In the absence of a more nucleophilic alkene reaction partner, α,β-unsaturated ketones do provide the corresponding aziridine products, as demonstrated with aziridines **14** and **15**, albeit in diminished yield.

We continued the evaluation of functional group compatibility with an epoxide-containing substrate, which smoothly afforded product **16** as a diastereomeric mixture. Likewise, an imide-containing alkene delivered a good yield (80%) of tricyclic aziridine **17**, which formed a separable diastereomeric mixture, with a slight preference for the formation of aziridine *syn* with respect to the imide. Ester groups are also amenable to the reaction conditions, as shown with the formation of aziridine **18**. Monoterpene (+)-3-carene gave the *trans*-aziridine **19** exclusively, and the reaction of (±)-camphene resulted in the corresponding spirocyclic aziridine **20** as a mixture of *endo*- and *exo*-products, highlighting the applicability of the methodology for the aziridination of 1,1-disubstituted alkenes and illustrating that highly strained molecules can be obtained. Finally, aziridine **21** was obtained in a good yield using the developed methodology. Of note, cyclophellitol-like aziridines are promising enzyme-inhibiting compounds.^34^ The synthesis from alkenes, however, can be challenging and generally requires multiple synthesis steps.^35,36^

## Conclusions

In summary, we have reported a synthetic methodology that transforms alkenes in sulfonyl-protected aziridines using nitrene radical anions, generated from a sulfonyl azide precursor, mediated by a photoredox catalytic cycle. The reaction mechanism has been investigated with luminescence quenching studies, reaction kinetics, trapping of reactive species, and UV–vis spectroscopy to bring convincing evidence that the reaction proceeds through the intermediacy of a nitrene radical anion, thereby presenting a new strategy for azide activation in the context of alkene aziridination. Crucially, the alkene can be used as the limiting reagent, and low catalyst loadings are used. The explored substrate scope indicates a good functional group tolerance as hydroxy, bromo, phosphonate, azido, carboxyl, silyl, epoxide, and imide groups were shown to be compatible with the reaction conditions. Furthermore, diastereoselective and regioselective reactions can be achieved. These features were demonstrated with the aziridination of relatively complex (derivatives of) biomolecules, showing that the methodology may be applied for the synthesis of complex aziridine-containing (bio)molecules.

## Data Availability

The data underlying this study are available in the published article and its Supporting Information.

## Supporting Information Available

Catalytic and synthetic procedures, procedures for mechanistic studies, and characterization of compounds (PDF)
