# Supplementary material for: Photocatalytic Nitrene Radical Anion Generation from Sulfonyl Azides for Intermolecular Aziridination of Unactivated Alkenes
Source: J Org Chem. 2025 May 3;90(19):6577–83. doi: 10.1021/acs.joc.5c00595 (PMC12090207; doi:10.1021/acs.joc.5c00595)
Supplement: Supplementary file 1 — jo5c00595_si_001.pdf [file jo5c00595_si_001.pdf]

Supporting information for

Photocatalytic Nitrene Radical Anion Generation from Sulfonyl Azides for Intermolecular Aziridination of Unactivated Alkenes

Dennis Dam[a#], Joeri Schoenmakers[a], Elisabeth Bouwman[a*], Jeroen D. C. Codée[a*]

[a]*Leiden Institute of Chemistry, Universiteit Leiden, 2333 CC Leiden, the Netherlands.*

Present address: [#] D.D.: Department of Chemical Engineering, Delft University of Technology, 2629 HZ Delft , the Netherlands.

Correspondence to: *Elisabeth Bouwman: bouwman@lic.leidenuniv.nl; *Jeroen D. C. Codée: jcodee@chem.leidenuniv.nl.

Table of Contents

1. General Experimental Details S2
2. General procedures S4
3. Luminescence Quenching Study S5
4. NMR reaction kinetics S6
5. Trapping nitrene radical anions S8
6. Reaction monitoring by UV-vis spectroscopy S9
7. Substrate Scope S10
8. Substrate Scope – Low yielding reactions or challenging purifications S19
9. Scale-up experiment S25
10. References S26
11. NMR Spectra S27

1. General Experimental Details

All commercial chemicals were used directly without purification unless stated otherwise. Solvents were dried over flame-dried molecular sieves of the appropriate size or obtained from a Pure-Solv 400 solvent purification system. Air and water sensitive reactions were performed under a dry nitrogen atmosphere using standard Schlenk techniques. TLC analysis was performed using TLC Silica gel (Kieselgel 60 F_{254}, Merck) with UV detection at 254 nm and by spraying a solution of $(NH_4)_6Mo_7O_{24}\cdot H_2O$ (25 g/L) and $(NH_4)_4Ce(SO_4)_4\cdot H_2O$ (10 g/mL) in 10% sulfuric acid and subsequently charred at ~300 ℃ using a hot plate. Flash column chromatography was performed manually using silica gel 60Å (40-63 µm) from Screening Devices. All mixed solvents are reported as v/v solutions. High-resolution mass spectra were recorded on a Thermo Finnigan LTQ Orbitrap mass spectrometer equipped with an electrospray ion source in positive mode (source voltage 3.5 kV, sheath gas flow 10, capillary temperature 275 °C) with resolution R=60.000 at m/z=400 (mass range = 150-4000). ^{1}H NMR, ^{13}C{^{1}H}-APT NMR, ^{19}F{^{1}H}-NMR and ^{31}P{^{1}H}-NMR spectra were recorded on a Bruker AV-500 (500/126/471 MHz), and a Bruker AV-400 (400/101/376/162 MHz) spectrometer. Chemical shifts are given in ppm relative to tetramethyl silane (TMS). 2D NMR experiments (HSQC, COSY, NOESY) were conducted to assign protons and carbons and the stereochemistry of the diastereoselective reactions. Reactions were irradiated using the EvoluChem PhotoRedOx box™ equipped with a 30 W blue LED (λ_{max} = 450 nm, HCK1012-01-002, EvoluChem™). The temperature was maintained between 27 °C and 29 °C by air cooling using the built-in fan. A custom-made holder was fitted to allow the use of small glass vials and NMR tubes (Figure S1).

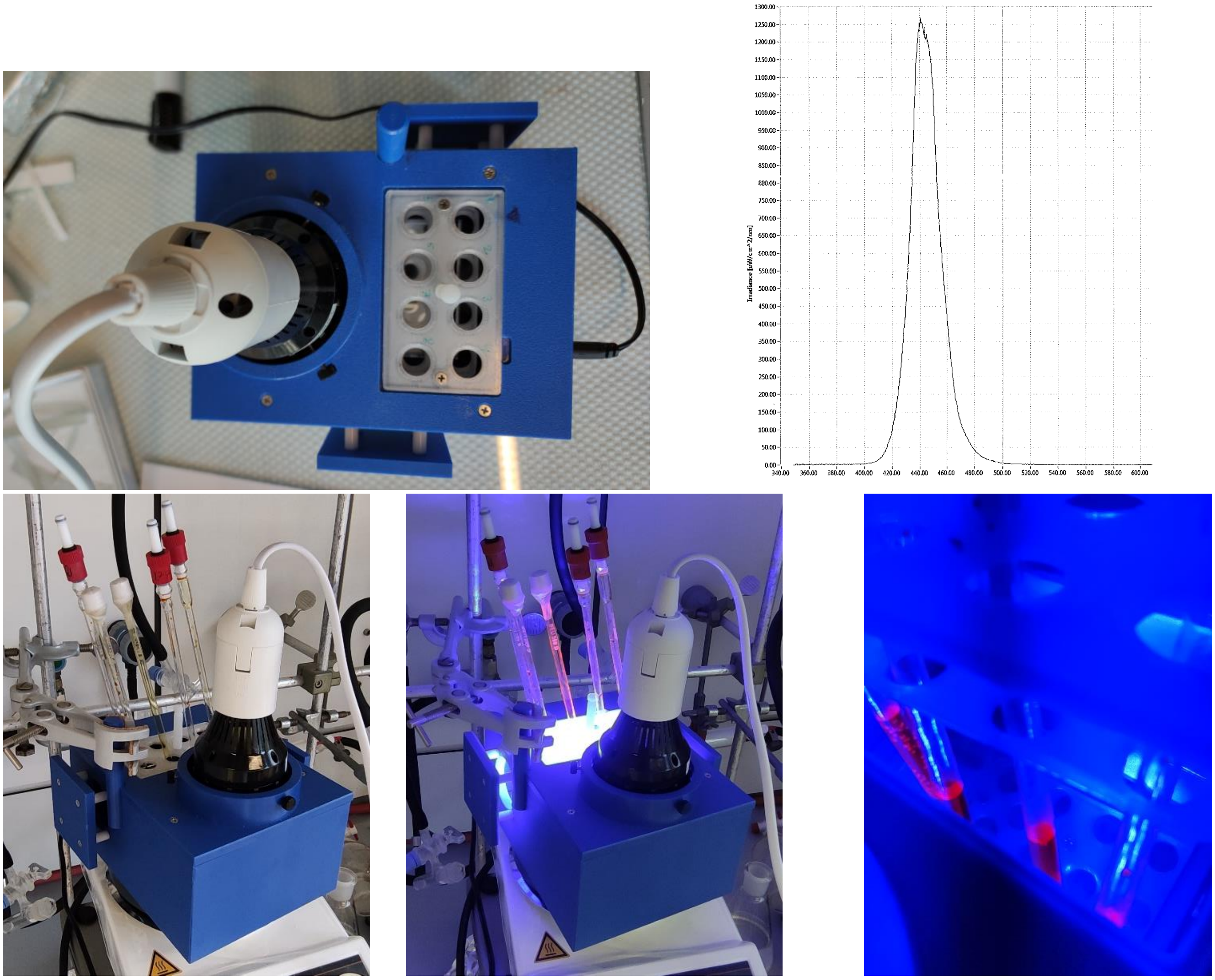

Figure S1. Up Left: Photochemical setup using the EvoluChem PhotoRedOx box™ including the custom-made holder. **Up Right:** Emission spectrum of the 450 nm LED lamp. **Bottom pictures:** Screw cap and J Young NMR tube reaction mixtures in the photoreactor.

All sulfonyl azides were prepared by reacting the respective sulfonyl chlorides with sodium azide according to a procedure by Laughlin and co-workers.[1] The precursor alkene used for the construction of **11** was prepared from 8-bromo-1-octene as previously described.[2] Alkene precursor for **13** was prepared as previously described.[3] The precursor alkene used for the construction of **17** was prepared by benzylation of *cis*-1,2,3,6-tetrahydrophthalimide following the benzylation procedure of Cossy and co-workers.[4] Spectral data matched that of the literature.[5] The synthesis of the precursor alkene used for the construction of **21** is described in our previous report.[6]

CAUTION! Sulfonyl azides are potentially explosive materials, hence appropriate personal protective equipment should be used when handling these energetic compounds. The authors recommend not to excessively heat these compounds nor exposing them to metal objects.

2. General procedures

General Procedure for Reactions in a Schlenk tube (GP1)

To a Schlenk tube charged with a PTFE stir bar, the indicated amounts of **Ru2** and sulfonyl azide were added. Then dried solvent was added followed by cyclohexene (25 µL, 0.25 mmol, 1.0 eq.) that had been passed over a short alumina column. The reaction mixture was subjected to three freeze-pump-thaw cycles ending on dinitrogen. The Schlenk tube was then positioned in the photoreactor and irradiated for the indicated time under constant stirring. Upon completion, the reaction mixture was concentrated, and the NMR yield was determined using 1,3,5-trimethoxybenzene as internal standard.

General Procedure for Reactions in an NMR tube (GP2)

To a screw-cap NMR tube, **Ru2** (4.30 mg, 0.020 eq., 5.00 µmol) and **1-*p*-CF_3** (314 mg, 5.0 eq., 1.25 mmol) were added. The tube was introduced in a long Schlenk tube and brought under an inert atmosphere by vacuum-dinitrogen cycling (3x), ending on dinitrogen. Degassed and dried CD_3CN (250 µL) was added to the NMR tube. Then the alkene was added (0.25 mmol, 1 eq.), the NMR tube was closed with the screw cap and shaken well to ensure homogeneity. The NMR tube was then placed in the photoreactor and irradiated. The reaction was monitored periodically by ^1H NMR spectroscopy. Upon completion, the reaction mixture was concentrated and subjected to flash column chromatography to afford the product.

3. Luminescence Quenching Study

Luminescence spectra were recorded on a FluorSpec F900. An excitation wavelength of 450 nm was used and the emission intensity from 500 nm to 800 nm was measured. One single quartz cuvette was used for all the measurements. Stock solutions of **Ru2** (40 µM) and **1-*p*-CF₃** (twice the concentration of the data point to be measured) were prepared using dry acetonitrile and subsequently degassed using three freeze-pump-thaw cycles and ending on argon. The cuvette was fitted with a septum that was pierced with a needle and introduced to a Schlenk tube. The Schlenk tube was closed and then brought under an argon atmosphere by repeated evacuation and refill cycles, ending on argon. The degassed stock solutions of **Ru2** (1.0 mL) and **1-*p*-CF₃** (1.0 mL) were added sequentially by standard needle and syringe techniques to the cuvette. Shortly before measuring, the Schlenk flask was opened, the needle taken out of the cuvette's septum and the cuvette was placed in the fluorimeter and the emission spectrum was measured. In this manner, five different concentrations of **1-*p*-CF₃** were measured (Figure S2A).

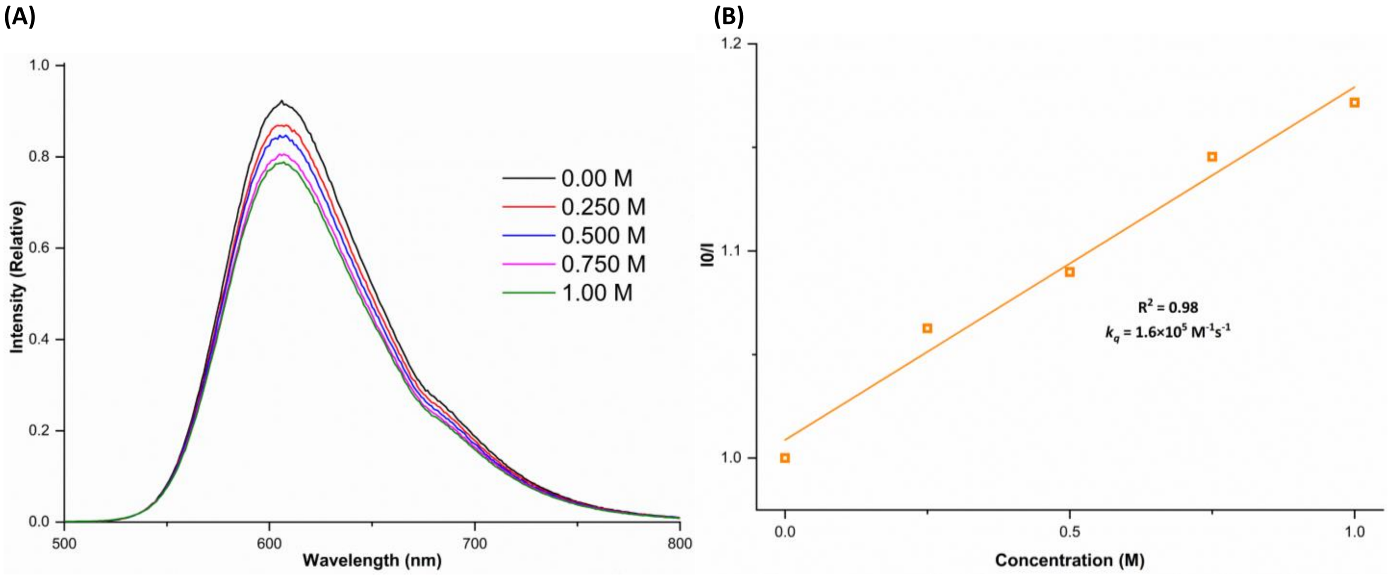

Figure S2. A) Emission quenching of **Ru2** at increasing concentrations of **1-*p*-CF₃** in MeCN. b) Stern-Volmer plot for the quenching of **Ru2** by **1-*p*-CF₃** in MeCN.

The emission intensities at the emission maximum of 620 nm were used to plot the Stern-Volmer plot (Figure S2B). From the slope of the fitted line in Figure S2B the Stern-Volmer constant (K_{sv}) was determined to be 0.170 M^{-1}. The lifetime of the excited state of **Ru2** in deaerated acetonitrile is known (τ_0 = 1100 ns).[7] From this, the rate constant of quenching by **1-*p*-CF₃** was determined to be 1.6 x 10^5 $M^{-1}s^{-1}$ utilizing equation 1.

$$K_{SV} = k_q \tau_0 \quad \text{(Eq. 1)}$$

<u>Discussion of the data:</u>

Azide **1-*p*-CF₃** is a poor quencher of the excited state of **Ru2** as relatively high concentrations of **1-*p*-CF₃** had to be used to observe any significant quenching, which results in a low k_q. Azide **1-*p*-CH₃** was also evaluated in quenching experiments, but no quenching could be detected as it is an even less efficient quencher. The inefficient quenching observed suggests that there is little driving force for the SET to occur as the rate of light-induced electron-transfer is correlated with the reaction free energy, Δ*G*, as reported by Rehm and Weller.[8]

The Δ*G* of our proposed SET was estimated using the simplified Rehm-Weller equation (Eq. 2). The reported values for **Ru2** are $E_{ox,Ru(III)/Ru(II)}$ = + 1.29 V vs SCE and $E_{0,0}$ = 2.12 eV.[9,10] For **1-*p*-CF₃**, $E_{red,azide}$ = − 1.10 V vs SCE was used (determined by cyclic voltammetry in our previous report).[6] Using these values in Eq. 2 it was found that: Δ*G* ≈ + 0.27 eV ≈ + 6 kcal mol^{-1}. The reaction therefore is initiated by an *endergonic* SET. Indeed, inspecting the original Rehm-Weller plot we can expect the rate (k_q) of our electron transfer to correspond with a Δ*G* > 0. Of note, endergonic SET processes are possible when coupled to a fast and irreversible subsequent step to prevent back electron transfer.[11] Our reaction classifies as such a reaction as dinitrogen loss from the azide following SET is irreversible.

$$\Delta G = (E_{ox,Ru(III)/Ru(II)} - E_{red,azide}) - E_{0,0} \quad \text{(Eq. 2)}$$

4. NMR reaction kinetics

To a Schlenk tube, **Ru2** (4.30 mg, 0.020 eq., 5.00 µmol), **1-R** (5.0 eq., 1.25 mmol) and 1,3,5-trimethoxybenzene (typically ~ 3.0 mg) were added. Then 0.50 mL CD_3CN was added and cyclohexene (25 µL, 0.25 mmol, 1.0 eq.) that was passed over a short alumina column was added. The reaction mixture was subjected to three freeze-pump-thaw cycles ending on dinitrogen. The reaction mixture was then transferred using standard syringe and needle techniques to a J Young NMR tube under dinitrogen atmosphere positioned in a long Schlenk tube. The NMR tube was taken out, quickly closed and a ^1H NMR spectrum was recorded. Then the NMR tube was placed in the photoreactor and irradiated. The reaction was monitored periodically by ^1H NMR spectroscopy and the aziridine was quantified at several time intervals using the known quantity of 1,3,5-trimethoxybenzene and the data was used to construct Figure 2A. Stacked and annotated NMR spectra are provided in Figures S3-S5.

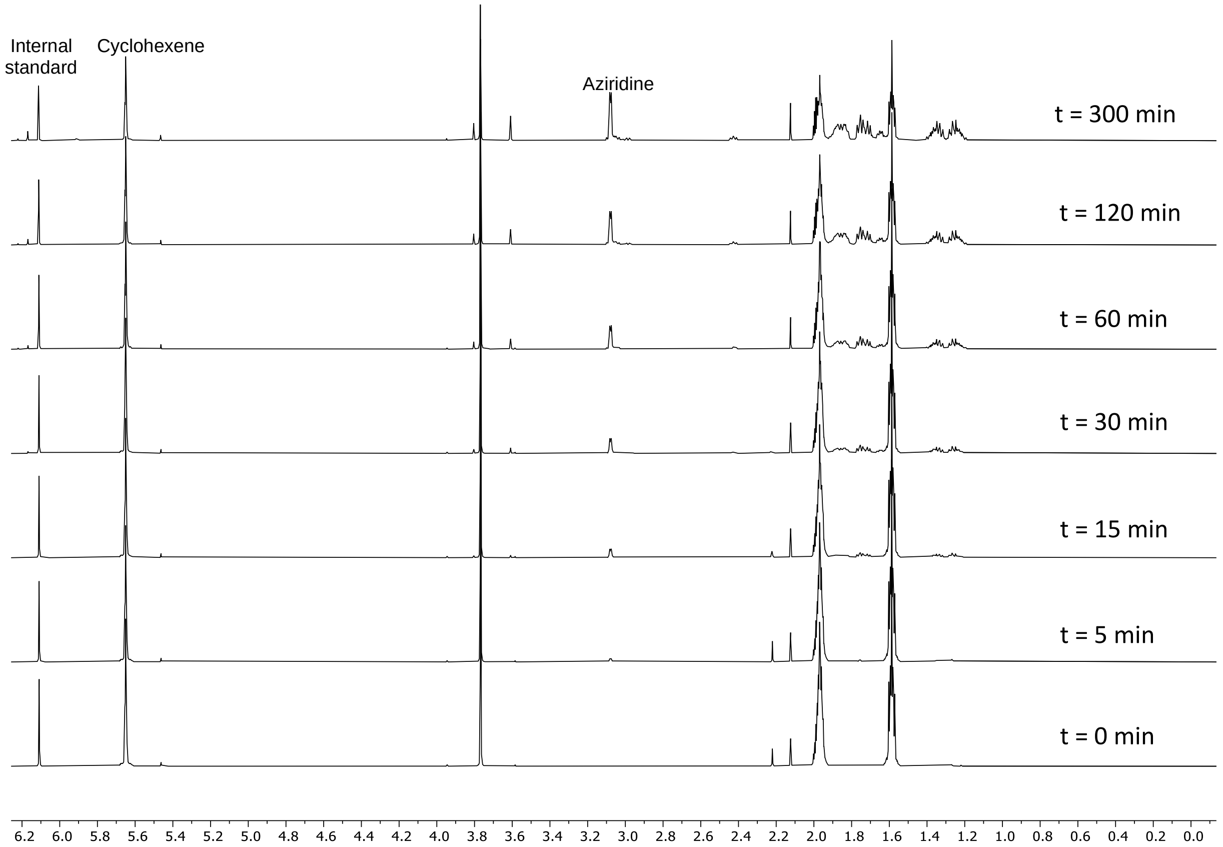

Figure S3. Stacked ^1H NMR spectra of the reaction in Figure 2A with **1-*p*-CF₃**. Reaction conditions: 0.25 mmol cyclohexene, 1.25 mmol **1-*p*-CF_3**, 5.0 µmol **Ru2**, ~3.0 mg 1,3,5-trimethoxybenzene (internal standard) in 0.50 mL CD_3CN. The indicated timepoints refer to cumulative irradiation times.

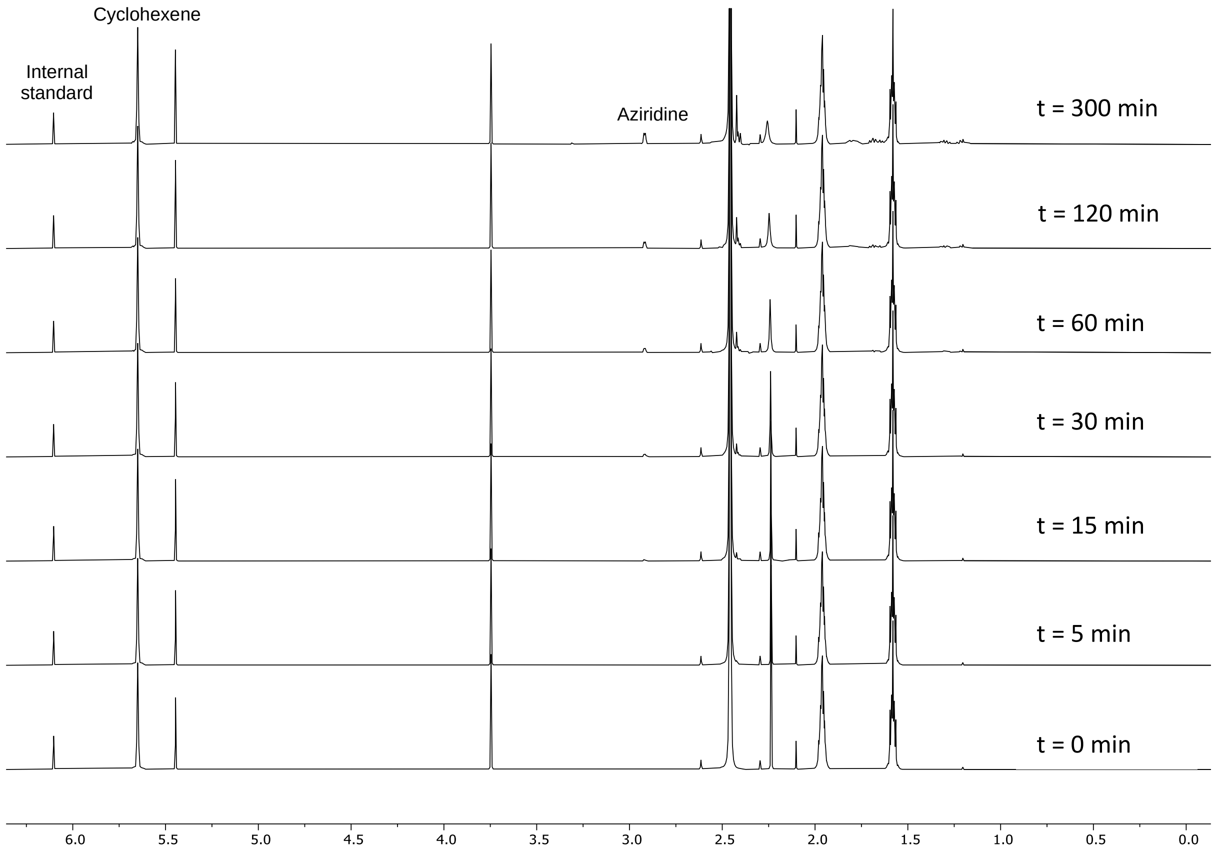

Figure S4. Stacked ^{1}H NMR spectra of the reaction in Figure 2A with **1-*p*-CH₃**. Reaction conditions: 0.25 mmol cyclohexene, 1.25 mmol **1-*p*-CH₃**, 5.0 µmol **Ru2**, ~3.0 mg 1,3,5-trimethoxybenzene (internal standard) in 0.50 mL CD_3CN. The indicated timepoints refer to cumulative irradiation times.

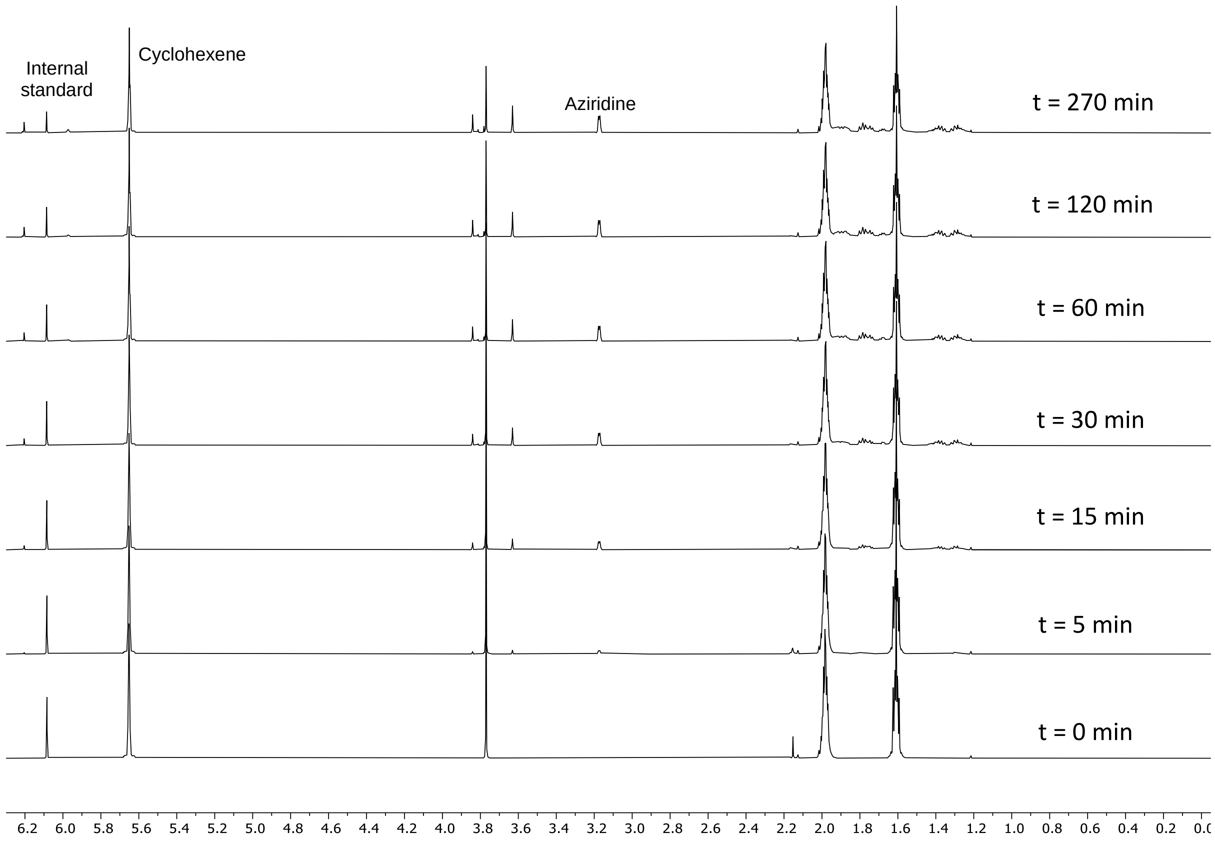

Figure S5. Stacked ^{1}H NMR spectra of the reaction in Figure 2A with **1-*m,m*-(CF₃)₂**. Reaction conditions: 0.25 mmol cyclohexene, 1.25 mmol **1-*m,m*-(CF₃)₂**, 5.0 µmol **Ru2**, ~3.0 mg 1,3,5-trimethoxybenzene (internal standard) in 0.50 mL CD_3CN. The indicated timepoints refer to cumulative irradiation times.

5. Trapping nitrene radical anions

All trapping experiments reported in Figure 2B were performed according to **GP2**. The NMR spectrum obtained in the experiment with **Ru2** is shown in Figure S6 and shows aziridine product and the absence of C-H amination product.

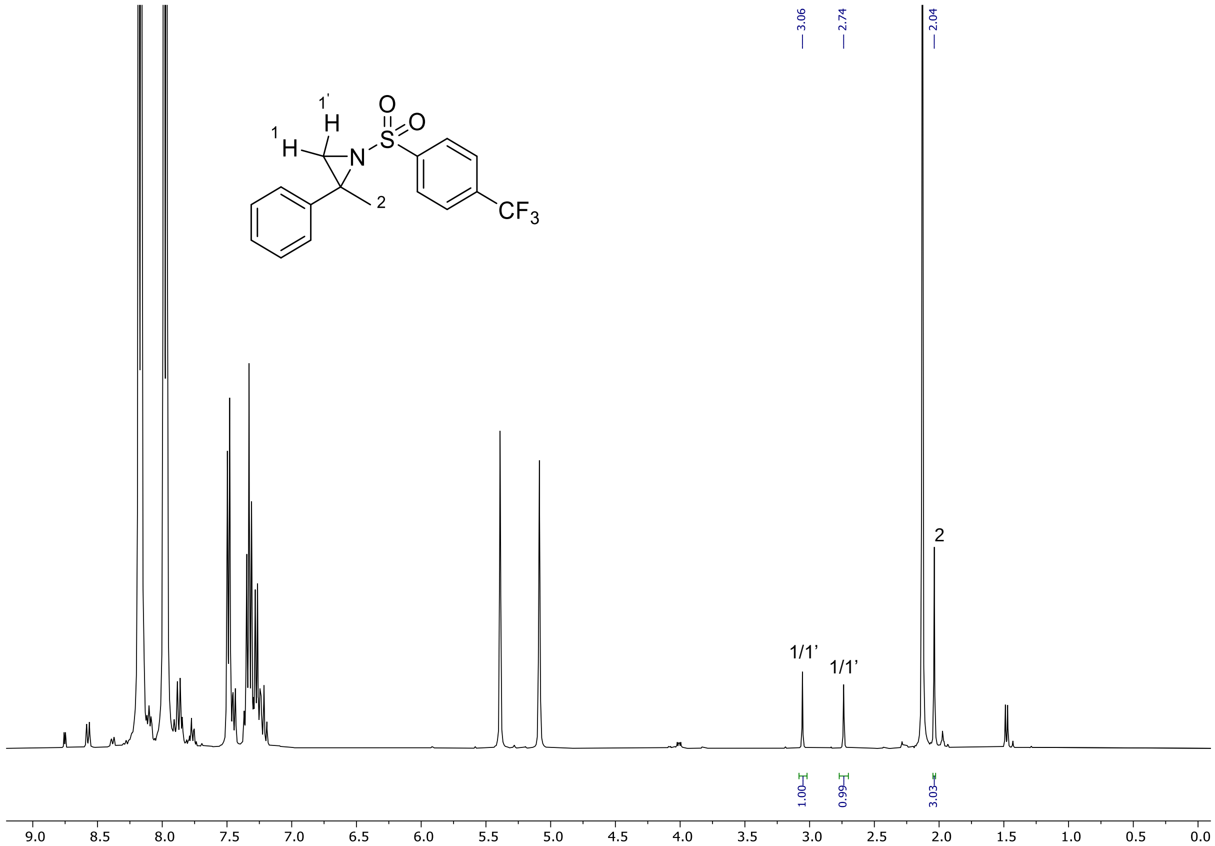

Figure S6. ^{1}H NMR spectrum of the reaction mixture in Figure 2A using **Ru2**, showing aziridine and absence of C-H amination product. Recorded after 50 minutes of irradiation. Reaction conditions: 0.25 mmol α-methylstyrene, 1.25 mmol **1-*p*-CF₃**, 5.0 µmol **Ru2**, 0.25 mL CD_3CN under 450 nm irradiation in a J Young NMR tube under a dinitrogen atmosphere.

6. Reaction monitoring by UV-vis spectroscopy

A GC vial was charged with a stir bar, **Ru2** (4.30 mg, 0.020 eq., 5.00 µmol) and **1-*p*-CF_3** (314 mg, 5.0 eq., 1.25 mmol). The GC vial was entered in a Schlenk tube and was deoxygenated by subjection to three vacuum and dinitrogen cycles, ending on dinitrogen. Then, dried, and degassed MeCN (250 µL) was added to the GC vial using syringe and needle. Cyclohexene (25 µL, 0.25 mmol, 1.0 eq.) passed over a short alumina column was added (for the reaction without cyclohexene this step was skipped), the GC vial was taken out of the Schlenk tube and closed with a screw cap. To prepare a UV-Vis sample, 3.0 mL MeCN was added to the cuvette and a 10 µL sample was taken from the reaction mixture of interest and added. The UV-vis spectrum was measured immediately. Stacked UV-vis spectra for the reaction without cyclohexene and with cyclohexene are shown in Figures 2C and 2D respectively.

7. Substrate Scope

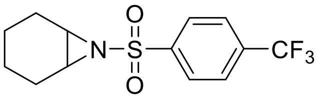

7-((4-(trifluoromethyl)phenyl)sulfonyl)-7-azabicyclo[4.1.0]heptane (**2-*p*-CF$_3$**). Prepared according to **GP2**. Crude purified by flash column chromatography (diethyl ether/pentane; 0.0:1.0 → 1.0:9.0) to give the title compound as a white solid (56 mg, 0.19 mmol, 74%).

^{1}H NMR (400 MHz, CDCl$_3$, H−H COSY, HSQC) δ 8.08 (d, *J* = 8.1 Hz, 2H, Ph), 7.81 (d, *J* = 8.3 Hz, 2H, Ph), 3.12 – 3.03 (m, 2H, N*CH*), 1.89 – 1.72 (m, 4H, *CH$_2$*CHN), 1.47 – 1.32 (m, 2H), 1.31 – 1.16 (m, 2H).

^{13}C{^{1}H}-APT NMR (101 MHz, CDCl$_3$, HSQC) δ 142.8 (m, C$_{q\text{-arom}}$SO$_2$N), 135.0 (q, $^2J_{CF}$ = 32.9 Hz, *C$_{q\text{-arom}}$*CF$_3$), 128.2 (CH$_{arom}$), 126.28 (q, $^3J_{CF}$ = 3.7 Hz), 123.3 (d, $^1J_{CF}$ = 272.9 Hz, CF$_3$), 40.7 (N*CH*), 22.8 (*CH$_2$*CHN), 19.4 (*CH$_2$*CH$_2$CH).

^{19}F{^{1}H}-NMR (376 MHz, CDCl$_3$) δ -63.4 (s, 3F, CF$_3$).

HRMS [M + H]$^+$ calcd. for $C_{13}H_{15}F_3NO_2S^+$ 306.0770; found 306.0773.

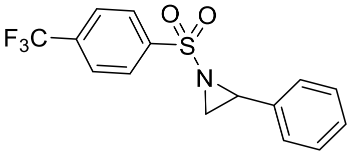

2-phenyl-1-((4-(trifluoromethyl)phenyl)sulfonyl)aziridine (**3**). Prepared according to **GP2**. Crude purified by flash column chromatography (diethyl ether/pentane; 0.0:1.0 → 1.0:9.0) to afford the title compound as a white solid (56 mg, 0.17 mmol, 69%).

^{1}H NMR (400 MHz, CDCl$_3$, H−H COSY, HSQC) δ 8.13 (d, *J* = 8.2 Hz, 2H, SO$_2$Ph), 7.81 (d, *J* = 8.2 Hz, 2H, SO$_2$Ph), 7.37 – 7.27 (m, 3H, Ph), 7.27 – 7.18 (m, 2H, Ph), 3.88 (dd, *J* = 7.2, 4.6 Hz, 1H, *CH*Ph), 3.07 (d, *J* = 7.2 Hz, 1H, CH$_2$), 2.47 (d, *J* = 4.6 Hz, 1H, CH$_2$).

^{13}C{^{1}H}-APT NMR (101 MHz, CDCl$_3$, HSQC) δ 134.6 (*C$_q$*CH), 128.8 (CH$_{arom}$), 128.8 (CH$_{arom}$), 128.6 (CH$_{arom}$), 126.6 (CH$_{arom,sulfonyl}$), 126.5 (q, $^3J_{CF}$ = 3.7 Hz, CH$_{arom,sulfonyl}$), 41.7 (CHN, 36.5 (CH$_2$N).

^{19}F{^{1}H}-NMR (376 MHz, CDCl$_3$) δ -63.5 (s, 3F, CF$_3$).

HRMS [M + H]$^+$ calcd. for $C_{15}H_{13}F_3NO_2S^+$ 328.0614; found 328.0617, [M + MeCN + H]$^+$ calcd. for $C_{17}H_{16}F_3N_2O_2S^+$ 369.0879; found 369.0883.

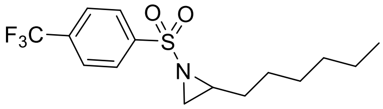

2-hexyl-1-((4-(trifluoromethyl)phenyl)sulfonyl)aziridine (**4**)**.** Prepared by according to **GP2**. Crude purified by flash column chromatography (diethyl ether/pentane; 0.0:1.0 → 1.0:9.0) to afford the title compound as a colorless oil (42 mg, 0.13 mmol, 50%).

^{1}H NMR (400 MHz, CDCl$_3$, H−H COSY, HSQC) δ 8.10 (d, *J* = 8.2 Hz, 2H, Ph), 7.83 (d, *J* = 8.4 Hz, 2H, Ph), 2.83 (tt, *J* = 7.4, 4.8 Hz, 1H, *CH*N), 2.72 (d, *J* = 7.0 Hz, 1H, N*CH$_2$*), 2.14 (d, *J* = 4.7 Hz, 1H, N*CH$_2$*), 1.63 – 1.52 (m, 1H, *CH$_2$*CHN), 1.40 – 1.29 (m, 1H, *CH$_2$*CHN), 1.28 – 1.10 (m, 8H, CH$_2$), 0.85 (t, *J* = 7.0 Hz, 3H, CH$_3$).

^{13}C{^{1}H}-APT NMR (101 MHz, CDCl$_3$, HSQC) δ 141.9 (m, C$_{q\text{-arom}}$SO$_2$N) 135.2 (q, $^2J_{CF}$ = 33.0 Hz, *C$_{q\text{-arom}}$*CF$_3$), 128.5 (CH$_{arom}$), 126.2 (q, $^3J_{CF}$ = 3.7 Hz, CH$_{arom}$), 123.2 (d, $^1J_{CF}$ = 273.0 Hz, CF$_3$), 41.1 (*CH*N), 34.3(N*CH$_2$*), 31.6 (CH$_2$), 31.3 (CH$_2$), 28.7 (CH$_2$), 26.8 (CH$_2$), 22.4 (CH$_2$), 14.0 (CH$_3$).

^{19}F{^{1}H}-NMR (376 MHz, $CDCl_3$) δ -63.5 (s, 3F, CF_3).

HRMS $[M + H]^+$ calcd. for $C_{15}H_{21}F_3NO_2S^+$ 336.1240; found 336.1243.

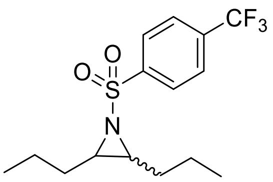

***cis*- and *trans*-2,3-dipropyl-1-((4-(trifluoromethyl)phenyl)sulfonyl)aziridine** (**5**). Prepared according to **GP2** from *cis*-4-octene. The aziridine products proved challenging to separate from the excess of azide. Therefore, it was decided to reduce the excess of azide. The crude was dissolved in THF (10 mL) and then water (10 mL) was added. Triphenylphosphine (360 mg) was added, and the reaction was stirred for 30 minutes. The reaction was diluted with water and the mixture was extracted thrice with diethyl ether. The organic layers were washed with brine, dried with $MgSO_4$, filtered, and concentrated. The crude was purified by flash column chromatography (diethyl ether/pentane; 0.0:1.0 → 1.0:19.0) to afford the title compounds as a colorless oil in a 2:1 ratio of *trans*:*cis* (60 mg, 0.18 mmol, 72%).

^{1}H NMR (400 MHz, $CDCl_3$, H–H COSY, HSQC) δ 8.08 (d, *J* = 7.8 Hz, 2H, Ph), 7.84 – 7.75 (m, 2H, Ph), 2.93 – 2.84 (m, 2H, CH_{cis}), 2.76 – 2.67 (m, 2H, CH_{trans}), 2.00 – 1.71 (m, 2H, $CH_{2,trans}$), 1.70 – 1.58 (m, 2H, CH_2), 1.54 – 1.44 (m, 2H, $CH_{2,cis}$), 1.41 – 1.18 (m, 16H, $CH_{2,cis+trans}$), 0.95 – 0.84 (m, 12H, $CH_{3,cis+trans}$).

^{13}C{^{1}H}-APT NMR (101 MHz, $CDCl_3$, HSQC) δ 144.4 ($C_{q\text{-}arom}SO_2N$), 134.7 (d, $^2J_{CF}$ = 33.1 Hz, $C_{q\text{-}arom}CF_3$), 128.6 ($CH_{arom,cis}$), 128.0 ($CH_{arom,trans}$), 126.1 (q, $^3J_{CF}$ = 4.0 Hz, CH_{arom}), 123.4 (d, $^1J_{CF}$= 272.5 Hz, CF_3), 50.5 (CHN_{trans}), 45.9 (CHN_{cis}), 32.1 (CH_2CHN_{trans}), 28.8 (CH_2CHN_{cis}), 20.9 (CH_3CH_{2trans}), 20.7 (CH_3CH_{2cis}), 13.8 (CH_3).

^{19}F{^{1}H}-NMR (376 MHz, $CDCl_3$) δ -63.3 (s, $3F_{trans}$, CF_3), -63.3 (s, $3F_{cis}$, CF_3).

HRMS $[M + H]^+$ calcd. for $C_{15}H_{21}F_3NO_2S^+$ 336.1240; found 336.1244.

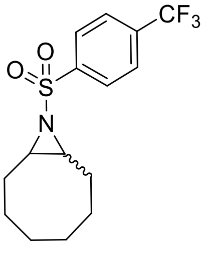

***cis*- and *trans*-9-((4-(trifluoromethyl)phenyl)sulfonyl)-9-azabicyclo[6.1.0]nonane** (**6**). Prepared according to **GP2** from *cis*-cyclooctene. Crude purified by flash column chromatography (diethyl ether/pentane; 0.0:1.0 → 1.0:9.0) to give the title compounds as a white solid (55 mg, 0.16 mmol, 66%, *cis*:*trans* = 5:1).

^{1}H NMR (400 MHz, $CDCl_3$, H-H COSY, HSQC) δ 8.10 (d, *J* = 8.2 Hz, 2H, $Ph_{cis+trans}$), 7.81 (dd, *J* = 7.9, 5.2 Hz, 2H, $Ph_{cis+trans}$), 2.93 – 2.85 (m, 2H, NCH_{cis}), 2.83 – 2.75 (m, 2H, NCH_{trans}), 2.34 – 2.25 (m, 2H, $CH_{2\text{-}trans}$), 2.12 (ddd, *J* = 12.9, 4.4, 2.7 Hz, 2H, $CH_{2\text{-}trans}$), 2.04 (dt, *J* = 13.9, 3.6 Hz, 2H, $NCHCHH_{cis}$), 1.98 (m, 2H, $CH_{2\text{-}trans}$), 1.66 – 1.38 (m, 10H, $CH_{2\text{-}cis+trans}$), 1.31 (dddd, *J* = 15.7, 12.2, 7.8, 2.5 Hz, 4H, $NCHCHH_{cis}$ + $CH_{2\text{-}trans}$), 1.10 – 0.99 (m, 2H, $CH_{2\text{-}trans}$).

^{13}C{^{1}H}-APT NMR (101 MHz, $CDCl_3$, HSQC) δ 142.7 (C_qS), 135.0 (q, $^2J_{CF}$ = 32.9 Hz, $C_{q\text{-}arom}CF_3$), 127.8 ($CH_{arom\text{-}cis}$), 127.8 ($CH_{arom\text{-}trans}$), 126.3 (q, $^3J_{CF}$ = 3.8 Hz, CH_{arom}), 50.36 (NCH_{trans}), 44.7 (NCH_{cis}), 30.5 ($CH_{2\text{-}trans}$), 29.7 ($CH_{2\text{-}trans}$), 28.1 ($CH_{2\text{-}trans}$), 26.4 ($CH_{2\text{-}cis}$), 26.2 ($CH_{2\text{-}cis}$), 25.3 ($NCHCH_{2\text{-}cis}$).

^{19}F{^{1}H}-NMR (376 MHz, $CDCl_3$) -63.1 (s, $3F_{trans}$, CF_3), -63.2 (s, $3F_{cis}$, CF_3).

HRMS $[M + H]^+$ calcd. for $C_{15}H_{19}F_3NO_2S^+$ 334.1083; found 334.1066.

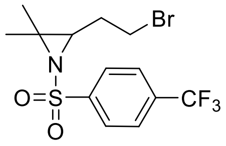

3-(2-bromoethyl)-2,2-dimethyl-1-((4-(trifluoromethyl)phenyl)sulfonyl)aziridine (**7**). Prepared according to **GP2** from 5-bromo-2-methyl-2-ene. Crude purified by flash column chromatography (toluene/pentane; 1.0:4.0 → 1.0:0.0) to afford the title compound as a colorless solid (68 mg, 0.18 mmol, 70%).

^{1}H NMR (400 MHz, $CDCl_3$, H−H COSY, HSQC) δ 8.11 (d, *J* = 8.0 Hz, 2H, Ph), 7.79 (d, *J* = 8.1 Hz, 2H, Ph), 3.28 (ddd, *J* = 10.1, 5.9, 4.9 Hz, 1H, CH_2Br), 3.12 (dd, *J* = 7.6, 5.3 Hz, 1H, CH aziridine), 3.01 (ddd, *J* = 10.1, 9.4, 5.5 Hz, 1H, CH_2Br), 2.12 – 1.98 (m, 1H, $\mathit{CH_2}CH_2Br$), 1.87 (ddt, *J* = 14.9, 7.7, 5.2 Hz, 1H, $C\mathit{H_2}CH_2Br$), 1.77 (s, 3H, CH_3) 1.34 (s, 3H, CH_3).

^{13}C{^{1}H}-APT NMR (101 MHz, $CDCl_3$, HSQC) δ 144.4 (m, $C_{q\text{-}arom}SO_2N$), 134.8 (q, $^{2}J_{CF}$ = 33.3 Hz, $\mathit{C_{q\text{-}arom}}CF_3$), 128.1 ($CH_{arom}$), 126.2 (q, $^{3}J_{CF}$ = 3.7 Hz, CH_{arom}), 123.3 (q, $^{1}J_{CF}$ = 273.7 Hz, CF_3), 51.7 (CH), 30.9 ($\mathit{CH_2}$ CH_2Br), 29.9 (CH_2Br), 21.6 (CH_3), 21.6 (CH_3).

^{19}F{^{1}H}-NMR (376 MHz, $CDCl_3$) δ -63.3 (s, 3F, CF_3).

HRMS $[M + H]^+$ calcd. for $C_{13}H_{16}BrF_3NO_2S^+$ 386.0032 (^{79}Br) and 388.0011 (^{81}Br); found 386.0038 (^{79}Br) and 388.0016 (^{81}Br).

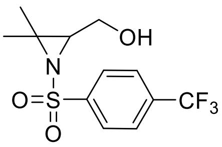

(3,3-dimethyl-1-((4-(trifluoromethyl)phenyl)sulfonyl)aziridin-2-yl)methanol (**8**). Prepared according to **GP2** from 3-methyl-2-buten-1-ol. Crude purified by flash column chromatography (ethyl acetate/pentane; 1.0:9.0 → 1.0:1.5) to afford the title compound as a pale-yellow oil (41 mg, 0.13 mmol, 53%).

^{1}H NMR (400 MHz, $CDCl_3$, H−H COSY, HSQC) δ 8.10 (d, *J* = 8.1 Hz, 2H, Ph), 7.78 (d, *J* = 8.3 Hz, 2H, Ph), 3.77 (dd, *J* = 11.9, 5.0 Hz, 1H, $C\mathit{H_2}OH$), 3.53 (dd, *J* = 11.9, 7.4 Hz, 1H, $C\mathit{H_2}OH$), 3.20 (dd, *J* = 7.4, 5.0 Hz, 1H, NCH), 1.76 (s, 3H, CH_3), 1.34 (s, 3H, CH_3).

^{13}C{^{1}H}-APT NMR (101 MHz, $CDCl_3$, HSQC) δ 128.0 (CH_{arom}), 126.3 (q, $^{3}J_{CF}$ = 3.7 Hz, CH_{arom}), 60.4 (CH_2), 53.4 (CH), 21.7 (CH_3), 21.3 (CH_3).

^{19}F{^{1}H}-NMR (376 MHz, $CDCl_3$) δ -63.4 (s, 3F, CF_3).

HRMS $[M + H]^+$ calcd. for $C_{12}H_{15}F_3NO_3S^+$ 310.0719; found 310.0717.

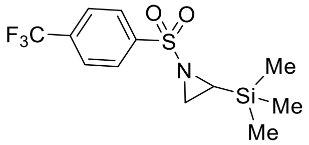

1-((4-(trifluoromethyl)phenyl)sulfonyl)-2-(trimethylsilyl)aziridine (**9**). Prepared according to **GP2** from vinyltrimethylsilane. Crude purified by flash column chromatography (diethyl ether/pentane; 0.0:1.0 → 1.0:9.0) to give the title compound as a colorless oil (28 mg, 0.086 mmol, 35%).

^{1}H NMR (400 MHz, $CDCl_3$, H-H COSY, HSQC) δ 8.09 (dt, *J* = 8.0, 0.8 Hz, 2H, Ph), 7.86 – 7.79 (m, 2H, Ph), 2.74 (d, *J* = 8.6 Hz, 1H, NC*H*H), 2.12 (d, *J* = 5.9 Hz, 1H, NC*H*H), 2.03 (dd, *J* = 8.6, 6.0 Hz, 1H, NC*H*), -0.06 (s, 9H, SiC*H*$_3$).

^{13}C {^{1}H}-APT NMR (101 MHz, $CDCl_3$, HSQC) δ 142.1 (C_qS), 128.8 ($\mathit{C}H_{arom}$), 126.3 (q, $^{3}J_{CF}$ = 3.8 Hz, $\mathit{C}H_{arom}$), 31.2 (N$\mathit{C}H_2$), 30.8 (N*C*H), -3.6 (Si*C*H$_3$).

^{19}F{^{1}H}-NMR (376 MHz, $CDCl_3$) -63.2 (s, 3F, CF_3).

HRMS $[M + H]^+$ calcd. for $C_{12}H_{17}F_3NO_2SSi^+$ 324.0696; found 324.0668.

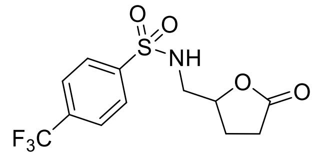

N-((5-oxotetrahydrofuran-2-yl)methyl)-4-(trifluoromethyl)benzenesulfonamide (**10**). Prepared according to **GP2** from 4-pentenoic acid. Crude purified by flash column chromatography (diethyl ether/pentane; 1.0:4.0 → 9.0:1.0; then with ethyl acetate/diethyl ether; 1.0:4.0) to afford the title compound as a colorless oil (38 mg, 0.12 mmol, 47%).

^{1}H NMR (400 MHz, $CDCl_3$, H–H COSY, HSQC) δ 8.01 (d, *J* = 8.2 Hz, 2H, CH_{arom}), 7.80 (d, *J* = 8.2 Hz, 2H, CH_{arom}), 5.80 (t, *J* = 6.6 Hz, 1H, NH), 4.70 – 4.60 (m, 1H, CHO), 3.34 (ddd, *J* = 13.9, 6.7, 3.2 Hz, 1H, CHN), 3.12 (dt, *J* = 14.0, 6.1 Hz, 1H, CHN), 2.63 – 2.56 (m, 2H, C_q=OCH_2), 2.39 – 2.28 (m, 1H, CHC*H_2*), 2.13 (dtd, *J* = 13.1, 9.3, 7.5 Hz, 1H, CHC*H_2*).

^{13}C{^{1}H}-APT NMR (101 MHz, $CDCl_3$, HSQC) δ 177.1 (C_q=O), 143.5 ($C_{q\text{-}arom}SO_2$), 134.7 (q, $^2J_{CF}$ = 33.1 Hz, $C_{q\text{-}arom}CF_3$), 127.6 (CH_{arom}), 126.6 (q, $^3J_{CF}$ = 3.7 Hz, CH_{arom}), 123.3 (d, $^1J_{CF}$ = 273.0 Hz, CF_3), 79.0 (CHO), 46.6 (CH_2N), 28.5 (C_qCH_2), 24.3 (CH*C*H_2).

^{19}F{^{1}H}-NMR (376 MHz, $CDCl_3$) δ -63.4 (s, 3F, CF_3).

HRMS $[M + H]^+$ calcd. for $C_{12}H_{13}F_3NO_4S^+$ 324.0512; found 324.0510. $[M + NH_4]^+$ calcd. for $C_{12}H_{16}F_3N_2O_4S^+$ 341.0777; found 341.0775. $[M + Na]^+$ calcd. for $C_{12}H_{12}F_3NNaO_4S^+$ 346.0331; found 346.0328.

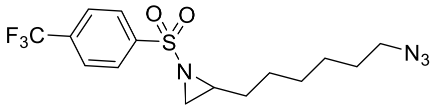

2-(6-azidohexyl)-1-((4-(trifluoromethyl)phenyl)sulfonyl)aziridine (**11**). Prepared according to **GP2** from 8-azidooct-1-ene. Crude purified by flash column chromatography (diethyl ether/pentane; 0.0:1.0 → 1.0:9.0) to give the title compound as a colorless oil (39 mg, 0.10 mmol, 42%).

^{1}H NMR (400 MHz, $CDCl_3$, H-H COSY, HSQC) δ 8.10 (d, *J* = 8.2 Hz, 2H, Ph), 7.83 (d, *J* = 8.3 Hz, 2H, Ph), 3.24 (t, *J* = 6.9 Hz, 2H, C*H_2*N_3), 2.84 (tt, *J* = 7.3, 4.7 Hz, 1H, NC*H*), 2.71 (d, *J* = 7.0 Hz, 1H, NC*H_2*), 2.13 (d, *J* = 4.7 Hz, 1H. NC*H_2*), 1.68 – 1.57 (m, 1H, NCHCH*H*), 1.53 (tt, *J* = 9.1, 6.9, 4.9 Hz, 2H, C*H_2*CH_2N_3), 1.41 – 1.33 (m, 1H, NCHCH*H*), 1.29 (dq, *J* = 6.7, 3.3, 2.7 Hz, 6H, C*H_2*).

^{13}C {^{1}H}-APT NMR (101 MHz, $CDCl_3$, HSQC) δ 142.0 (*C_q*S), 135.8 - 134.8 (m, $C_{q\text{-}arom}CF_3$), 128.6 (CH_{arom}), 126.3 (q, $^3J_{CF}$ = 3.7 Hz) 51.4 (*C*H_2N_3), 40.9 (*C*HN), 34.5 (*C*H_2N), 31.2 (*C*$H_2CH_2N_3$), 28.7 (*C*H_2), 28.6 (*C*H_2), 26.8 (*C*H_2), 26.6 (*C*H_2).

^{19}F{^{1}H}-NMR (376 MHz, $CDCl_3$) -63.2 (s, 3F, CF_3).

HRMS $[M + H - N_2]^+$ calcd. for $C_{15}H_{20}F_3N_2O_2S^+$ 349.1192; found 349.1158.

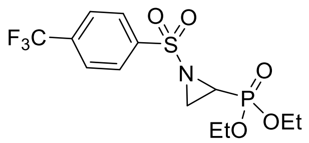

diethyl (1-((4-(trifluoromethyl)phenyl)sulfonyl)aziridin-2-yl)phosphonate (**12**). Prepared according to **GP2** with 5 mol% **Ru2** using diethyl vinylphosphonate as substrate. Crude purified by flash column chromatography (ethyl acetate/pentane; 5.0:5.0 → 1.0:0.0) to give the title compound as a colorless oil (21 mg, 0.054 mmol, 22%).

^{1}H NMR (400 MHz, $CDCl_3$, H-H COSY, HSQC) δ 8.15 – 8.10 (m, 2H, Ph), 7.88 – 7.83 (m, 2H, Ph), 4.18 – 3.96 (m, 4H, OCH_2), 2.94 (dddd, J = 15.3, 7.6, 4.7, 0.9 Hz, 1H, NC*H*), 2.87 – 2.80 (m, 1H, NC*H*H), 2.59 (ddd, J = 9.2, 4.7, 0.9 Hz, 1H, NC*H*H), 1.32 – 1.19 (m, 6H, CH_3).

^{13}C {^{1}H}-APT NMR (101 MHz, $CDCl_3$, HSQC) δ 140.9 (C_qS), 135.9 ($C_{q\text{-arom}}CF_3$), 129.0 (C_{arom}), 126.5 (q, $^3J_{CF}$ = 3.8 Hz), 63.5 (d, $^2J_{CP}$ = 48.9 Hz, OCH_2), 63.5 (d, $^2J_{CP}$ = 48.8 Hz, OCH_2), 32.0 (d, $^2J_{CP}$ = 207.2 Hz, NCH_2), 30.9 (d, $^1J_{CP}$ = 4.3 Hz, NCH), 16.4 (t, $^3J_{CP}$ = 6.5 Hz, CH_3).

^{19}F{^{1}H}-NMR (376 MHz, $CDCl_3$) -63.3 (s, 3F, CF_3).

^{31}P{^{1}H}-NMR (162 MHz, $CDCl_3$) δ 16.2 (s, 1P).

HRMS $[M + H]^+$ calcd. for $C_{13}H_{18}F_3NO_5PS^+$ 388.0590; found 388.0551.

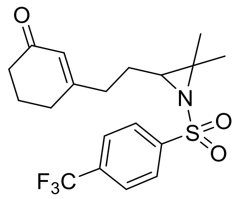

3-(2-(3,3-dimethyl-1-((4-(trifluoromethyl)phenyl)sulfonyl)aziridin-2-yl)ethyl)cyclohex-2-en-1-one (**13**). Prepared according to **GP2** from 3-(4-methylpent-3-en-1-yl)cyclohex-2-en-1-one. Crude purified by flash column chromatography (diethyl ether/pentane; 1.0:9.0 → 4.0:1.0) to afford the title compound as a colorless oil (55 mg, 0.14 mmol, 55%).

^{1}H NMR (400 MHz, $CDCl_3$, H–H COSY, HSQC) δ 8.09 (d, *J* = 8.2 Hz, 2H, Ph), 7.81 (d, *J* = 8.2 Hz, 2H, Ph), 5.76 (t, *J* = 1.5 Hz, 1H, CH enone), 2.93 (dd, *J* = 8.0, 5.4 Hz, 1H, CHN), 2.34 (dd, *J* = 7.4, 6.0 Hz, 2H, endocyclic $CH_2C_{q,alkene}$), 2.19 – 2.09 (m, 3H, CH_2C=O + $CH_2C_{q,alkene}$ exocyclic), 2.03 – 1.91 (m, 3H, CH_2*CH_2*CH_2 + $CH_2C_{q,alkene}$ exocyclic), 1.79 – 1.69 (m, 4H, CH_3 + *CH_2*CHN), 1.62 – 1.46 (m, 1H, *CH_2*CHN), 1.33 (s, 3H, CH_3).

^{13}C{^{1}H}-APT NMR (101 MHz, $CDCl_3$, HSQC) δ 199.6 (C_q=O), 163.9 (C_q enone), 144.5 ($C_{q\text{-arom}}SO_2$), 134.8 (d, $^2J_{CF}$ = 33.3 Hz, $C_{q\text{-arom}}CF_3$), 128.0 (CH_{arom}), 126.2 (q, $^3J_{CF}$ = 3.8 Hz, CH_{arom}), 125.8 (CH enone), 123.3 (q, $^1J_{CF}$ = 273.0 Hz), 53.1 (C_qN), 52.4 (CHN), 37.3 (endocyclic *CH_2*$C_{q,alkene}$), 35.3 (exocyclic *CH_2*$C_{q,alkene}$), 29.8 (*CH_2*C_q=O), 25.4 (*CH_2*CHN), 22.6 (CH_2*CH_2*CH_2), 21.7 (CH_3), 21.1 (CH_3).

^{19}F{^{1}H}-NMR (376 MHz, $CDCl_3$) δ -63.3 (s, 3F, CF_3).

HRMS $[M + H]^+$ calcd. for $C_{19}H_{23}F_3NO_3S^+$ 402.1345; found 402.1348, $[M + Na]^+$ calcd. for $C_{19}H_{22}F_3NNaO_3S^+$ 424.1165; found 424.1167.

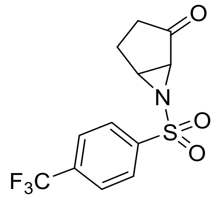

6-((4-(trifluoromethyl)phenyl)sulfonyl)-6-azabicyclo[3.1.0]hexan-2-one (**14**). Prepared according to **GP2**. Crude purified by flash column chromatography (diethyl ether/pentane; 0.5:9.5 → 3.0:7.0) to give the title compound as colorless oil (22 mg, 0.072 mmol, 29%).

^{1}H NMR (400 MHz, $CDCl_3$, H-H COSY, HSQC) δ 8.14 – 8.06 (m, 2H, Ph), 7.85 (d, J = 8.1 Hz, 2H, Ph), 3.92 – 3.85 (m, 1H, NC*H*CH_2), 3.36 (dd, J = 4.6, 0.8 Hz, 1H, COCH), 2.38 – 2.08 (m, 4H, CH_2).

^{13}C{^{1}H}-APT NMR (101 MHz, $CDCl_3$, HSQC) δ 206.3 (C=O), 128.6 (CH_{arom}), 126.7 (q, $^3J_{CF}$ = 3.7 Hz, CH_{arom}), 45.9 (COCHN), 44.8 (NCHCH_2), 31.8 (COCH_2), 23.3 (NCHCH_2).

^{19}F{^{1}H}-NMR (376 MHz, $CDCl_3$) -63.3 (s, 3F, CF_3)

HRMS $[M + H]^+$ calcd. for $[C_{12}H_{11}F_3NO_3S^+]$ 306.0406; found 306.0408.

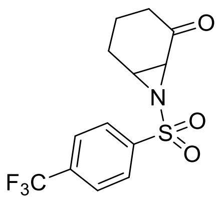

7-((4-(trifluoromethyl)phenyl)sulfonyl)-7-azabicyclo[4.1.0]heptan-2-one (**15**). Prepared according to GP2. Crude purified by flash column chromatography (diethyl ether/pentane; 0.5:9.5 → 3.0:7.0) to give the title compound as colorless oil (26 mg, 0.081 mmol, 33%).

^{1}H NMR (400 MHz, $CDCl_3$, H-H COSY, HSQC) δ 8.11 – 8.06 (m, 2H, Ph), 7.84 (d, J = 8.3 Hz, 2H, Ph), 3.57 (ddt, J = 6.7, 2.9, 1.5 Hz, 1H, CHNC*H*), 3.25 (d, J = 6.6 Hz, 1H, COC*H*N), 2.49 – 2.39 (m, 1H, COCH*H*), 2.28 – 2.18 (m, 1H, NCHCH*H*), 2.12-2.00 (m, 1H, COCH*H*), 1.97 – 1.80 (m, 2H, $CH_2CH\textit{H}CH_2$, NCHCH*H*), 1.77 – 1.65 (m, 1H, $CH_2CH\textit{H}CH_2$).

^{13}C{^{1}H}-APT NMR (101 MHz, $CDCl_3$, HSQC) 200.8 (C=O), 141.3 ($C_{q\text{-arom}}$S), 135.9 ($C_{q\text{-arom}}CF_3$), 128.6 (CH_{arom}), 126.7 (q, $^3J_{CF}$ = 3.8 Hz, CH_{arom}), 44.4 (CO*C*HN), 41.3 (N*C*HCH_2), 37.3 (*C*H_2CO), 21.9 (NCH*C*H_2), 16.9 (CH_2*C*H_2CH_2).

^{19}F{^{1}H}-NMR (376 MHz, $CDCl_3$) -63.3 (s, 3F, CF_3).

HRMS $[M + H]^+$ calcd. for $[C_{13}H_{13}F_3NO_3S]^+$ 320.0563; found 320.0564.

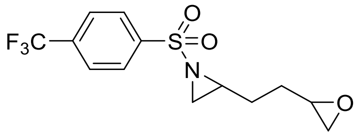

2-(2-(oxiran-2-yl)ethyl)-1-((4-(trifluoromethyl)phenyl)sulfonyl)aziridine (**16**). Prepared according to **GP2**. Crude purified by flash column chromatography (diethyl ether/pentane; 0.5:9.5 → 2.0:8.0) to give the title compound as colorless oil as a 1:1 mixture of diastereomers (53 mg, 0.16 mmol, 66%).

^{1}H NMR (400 MHz, $CDCl_3$, H-H COSY, HSQC) δ 8.10 (dd, J = 8.6, 2.3 Hz, 4H, Ph), 7.83 (d, J = 8.2 Hz, 4H, Ph), 2.93 (ddp, J = 10.0, 6.7, 3.3 Hz, 4H, CH_2N/CH_2O), 2.79 – 2.69 (m, 4H, CH_2N/CH_2O), 2.48 (dt, J = 4.8, 2.2 Hz, 1H, CHO/CHN), 2.41 (dd, J = 4.9, 2.7 Hz, 1H, CHO/CHN), 2.16 (d, J = 4.6 Hz, 2H, CHO/CHN), 1.91 – 1.63 (m, 4H, CH_2), 1.60 – 1.45 (m, 3H, CH_2), 1.37 (ddt, J = 13.9, 9.1, 6.5 Hz, 1H, CH_2).

^{13}C{^{1}H}-APT NMR (101 MHz, $CDCl_3$, HSQC) δ 128.7 (CH_{arom}), 128.6 (CH_{arom}), 126.3 (m, CH_{arom}), 51.6 (CHO), 51.3 (CHO), 47.1 (CH_2O), 47.0 (CH_2O), 40.4 (CHN), 40.0 (CHN), 34.8 (CH_2N), 34.6 (CH_2N), 29.9 (CH_2), 29.5 (CH_2), 28.3 (CH_2), 27.8 (CH_2).

^{19}F{^{1}H}-NMR (376 MHz, $CDCl_3$) -63.2 (s, 6F CF_3, both diastereomers).

HRMS $[M + Na]^+$ calcd. for $[C_{13}H_{14}F_3NO_3SNa^+]$ 344.0539; found 344.0536.

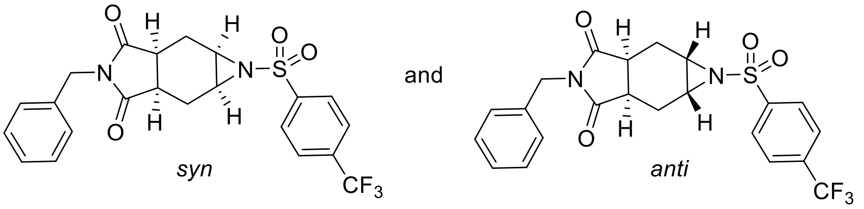

4-benzyl-1-((4-(trifluoromethyl)phenyl)sulfonyl)hexahydroazirino[2,3-f]isoindole-3,5(1H,4H)-dione (*syn* and *anti*, **17**). Prepared according to **GP2** from 2-benzyl-3a,4,7,7a-tetrahydro-1H-isoindole-1,3(2H)-dione. Crude purified by flash column chromatography (diethyl ether/pentane; 1.0:4.0 → 1.0:0.0; then with ethyl acetate/diethyl ether; 9.0:1.0 → 1.0:0.0) to give first the *syn*-diastereomer (55 mg, 0.12 mmol 47%) and then the *anti*-diastereomer (38 mg, 82 µmol, 33%) as colorless solids. The relative stereochemistry of the products was corroborated with NOESY experiments. For the *syn* diastereomer NOE was observed between CHN and CHC=O while for the *anti*-diastereomer this was not observed.

<u>*Syn*:</u> **^{1}H NMR** (400 MHz, $CDCl_3$, H−H COSY, H−H NOESY, HSQC) δ 8.06 (d, *J* = 8.1 Hz, 2H, SO_2Ph), 7.83 (d, *J* = 8.3 Hz, 2H, SO_2Ph), 7.33 – 7.28 (m, 5H, Ph), 4.60 (s, 2H, (CH_2Ph), 3.24 – 3.19 (m, 2H, CHN), 2.87 (tt, *J* = 9.2, 4.6 Hz, 2H, CHC=O), 2.55 – 2.45 (m, 2H, CH_2CHN), 1.90 – 1.79 (m, 2H, CH_2CHN).

^{13}C{^{1}H}-APT NMR (101 MHz, $CDCl_3$, HSQC) δ 178.5 (C_q=O), 142.0 ($C_{q\text{-}arom}SO_2N$), 135.6 (C_qPh), 135.5 (q, $^{2}J_{CF}$ = 33.6 Hz, $C_{q\text{-}arom}CF_3$), 128.8 (CH_{arom}), 128.7 (CH_{arom}), 128.2 (CH_{arom}), 128.2 (CH_{arom}), 126.6 (q, $^{3}J_{CF}$ = 3.7 Hz, CH_{arom}), 123.2 (q, $^{1}J_{CF}$ = 273.1 Hz, CF_3), 42.4 (*C*H_2Ph), 38.0 (CHN), 35.4 (*C*HC_q=O), 21.7 (*C*H_2CHN).

^{19}F{^{1}H}-NMR (376 MHz, $CDCl_3$) δ -63.4 (s, 3F, CF_3).

HRMS $[M + H]^+$ calcd. for $C_{22}H_{20}F_3N_2O_4S^+$ 465.1090; found 465.1094.

<u>*Anti*:</u> **^{1}H NMR** (400 MHz, $CDCl_3$, H−H COSY, H−H NOESY, HSQC) δ 7.77 (d, *J* = 8.6 Hz, 2H, SO_2Ph), 7.72 (d, *J* = 8.4 Hz, 2H, SO_2Ph), 7.35 – 7.20 (m, 5H, Ph), 4.31 (s, 2H, (CH_2Ph), 3.20 – 3.15 (m, 2H, CHN), 2.82 – 2.73 (m, 2H, CHC=O), 2.68 (dt, *J* = 15.5, 1.8 Hz, 2H, CH_2CHN), 2.10 (ddd, *J* = 15.4, 5.5, 2.5 Hz, 2H, CH_2CHN).

^{13}C{^{1}H}-APT NMR (101 MHz, $CDCl_3$, HSQC) δ 179.2 (C_q=O), 141.8 ($C_{q\text{-}arom}SO_2N$), 135.7 (C_qPh), 135.3 (q, $^{2}J_{CF}$ = 33.2 Hz, $C_{q\text{-}arom}CF_3$), 128.8 (CH_{arom}), 128.1 (CH_{arom}), 127.9 (CH_{arom}), 126.4 (q, $^{3}J_{CF}$ = 3.8 Hz, CH_{arom}), 123.2 (q, $^{1}J_{CF}$ = 273.0 Hz, CF_3), 42.6 (*C*H_2Ph), 39.4 (CHN), 34.8 (*C*HC_q=O), 20.7 (*C*H_2CHN).

^{19}F{^{1}H}-NMR (376 MHz, $CDCl_3$) δ -63.3 (s, 3F, CF_3).

HRMS $[M + Na]^+$ calcd. for $C_{22}H_{19}F_3N_2NaO_4S^+$ 487.0910; found 487.0908.

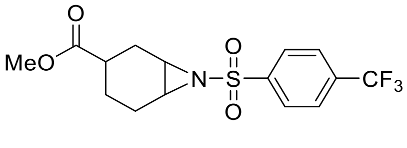

Methyl 7-((4-(trifluoromethyl)phenyl)sulfonyl)-7-azabicyclo[4.1.0]heptane-3-carboxylate (**18**). Prepared according to **GP2**. Crude purified by flash column chromatography (diethyl ether/pentane; 0.5:9.5 → 2.0:8.0) to give the title compound as colorless oil as a 1:1.5 (syn:anti) mixture (33 mg, 0.09 mmol, 36%).

^{1}H NMR (400 MHz, $CDCl_3$, H-H COSY, HSQC, NOESY) δ 8.11 – 8.05 (m, 4H, Ph-syn, Ph-anti), 7.83 (dt, J = 8.3, 0.7 Hz, 4H, Ph-syn, Ph-anti), 3.66 (s, 1H, OMe-syn), 3.64 (s, 1H, OMe-anti), 3.20 (ddd, J = 7.1, 3.9, 1.6 Hz, 1H, NCH-syn), 3.15 (ddd, J = 7.2, 3.3, 1.4 Hz, 1H, NCH-anti), 3.13 – 3.08 (m, 2H, NCH-syn, NCH-anti), 2.52 – 2.44 (m, 1H, CHCO-anti), 2.29 – 2.17 (m, 2H, CHCO-anti, CHH-anti), 2.15 – 2.05 (m, 2H, 2x CH-syn), 2.04 – 1.95 (m, 3H, CH-syn, 2x CH-anti), 1.84 – 1.73 (m, 3H, CH-syn, 2x CH-anti), 1.72 – 1.66 (m, 1H, CH-syn), 1.55 (tdd, J = 11.3, 4.6, 2.1 Hz, 1H, CH-anti), 1.46 – 1.39 (m, 1H, CH-syn).

$^{13}C\{^1H\}$-APT NMR (101 MHz, $CDCl_3$, HSQC) δ 175.3 (C=O-syn), 174.8 (C=O-anti), 128.3 (CH_{arom}-syn), 128.2 (CH_{arom}-anti), 126.4 (m, CH_{arom}-syn, CH_{arom}-anti), 52.1 (OMe-syn), 52.0 (OMe-anti), 40.7 (CH-syn), 40.1 (CH_2-anti), 39.7 (CH-syn), 39.2 (CH_2-anti), 37.7 (CH_2-anti), 35.9 (CH-syn), 25.5 (CH_2-syn), 24.7 (CH_2-anti), 23.0 (CH_2-syn), 22.8 (CH_2-anti), 21.4 (CH_2-syn), 21.3 (CH_2-anti).

$^{19}F\{^1H\}$-NMR (376 MHz, $CDCl_3$) -63.2 (s, 6F, CF_3, *syn+anti*)

HRMS $[M + H]^+$ calcd. for $C_{15}H_{18}F_3NO_4S^+$ 364.0825; found 364.0823.

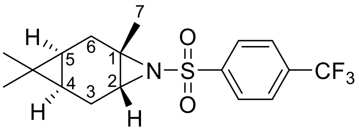

(1S,3S,5R,7R)-3,8,8-trimethyl-4-((4-(trifluoromethyl)phenyl)sulfonyl)-4-azatricyclo[5.1.0.03,5]octane (**19**). Prepared according to **GP2** from (+)-3-carene. Crude purified by flash column chromatography (diethyl ether/pentane; 0:1 → 1:9) to afford the title compound as a colorless solid (58 mg, 0.16 mmol, 65%). The relative stereochemistry of the product was corroborated with a NOESY experiment as NOE was observed between CHN and one of the CH_3 groups of the cyclopropyl rings.

^1H NMR (400 MHz, $CDCl_3$, H–H COSY, H–H NOESY, HSQC) δ 8.11 – 8.04 (m, 2H, Ph), 7.78 (d, J = 8.4 Hz, 2H, Ph), 3.00 (t, J = 2.4 Hz, 1H, H-2), 2.31 (dd, J = 16.0, 9.4 Hz, 1H, H-6), 2.02 (ddd, J = 16.2, 9.5, 2.1 Hz, 1H, H-3), 1.68 (s, 3H, CH_3-7), 1.47 (dt, J = 16.1, 3.1 Hz, 1H, H-3), 1.29 (dd, J = 16.0, 3.3 Hz, 1H, H-6), 0.97 (s, 3H, CH_3), 0.69 (s, 3H, CH_3), 0.52 (td, J = 9.3, 3.3 Hz, 1H, H-5), 0.32 (td, J = 9.4, 3.3 Hz, 1H, H-4).

$^{13}C\{^1H\}$-APT NMR (101 MHz, $CDCl_3$, HSQC) δ 145.5 ($C_{q\text{-}arom}SO_2$), 134.4 (q, $^2J_{CF}$ = 32.3 Hz, $C_{q\text{-}arom}CF_3$), 127.5 (CH_{arom}), 126.2 (q, $^3J_{CF}$ = 3.0 Hz, CH_{arom}), 123.44 (d, $^1J_{CF}$ = 273.0 Hz, CF_3), 49.9 (C-1), 47.0 (C-2), 27.7 (CH_3), 25.3 (C-6), 20.0 (C-7), 17.8 (C-3), 16.6 (C_q, cyclopropyl), 16.1 (C-5), 15.2 (CH_3), 13.6 (C-4).

$^{19}F\{^1H\}$-NMR (376 MHz, $CDCl_3$) δ -63.3 (s, 3F, CF_3).

HRMS $[M + H]^+$ calcd. for $C_{17}H_{21}F_3NO_2S^+$ 360.1240; found 360.1244.

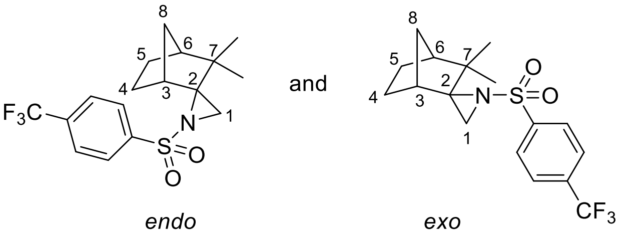

endo *exo*

3',3'-dimethyl-1-((4-(trifluoromethyl)phenyl)sulfonyl)spiro[aziridine-2,2'-bicyclo[2.2.1]heptane] (**20**). Prepared according to **GP2** from (±)-camphene. The aziridine products proved challenging to separate from the excess of azide. Therefore, it was decided to reduce the excess of azide. The crude was dissolved in THF (10 mL) and then water (10 mL) was added. Triphenylphosphine (360 mg) was added, and the reaction was stirred for 30 minutes. The reaction was diluted with water and the mixture was extracted with diethyl ether (3x). The organic layers were washed with brine, dried with $MgSO_4$, filtered, and concentrated. The crude product was purified by flash column chromatography (diethyl ether/pentane; 1.0:49 → 1.0:4.0) to give the title compounds as a colorless solid and as a mixture of *endo*- and *exo*-diastereomers in a 1.7:1.0 ratio (56 mg, 0.16 mmol, 62%). It could not be determined which was the major or minor diastereomer.

^1H NMR (400 MHz, $CDCl_3$, H–H COSY, H–H NOESY, HSQC) δ 8.12 – 8.05 (m, 4H, CH_{arom} *endo* + *exo*), 7.82 – 7.76 (m, 4H, CH_{arom} *endo* + *exo*), 2.80 (dd, J = 4.9, 1.6 Hz, 1H, H-3, minor), 2.74 (s, 1H, H-1 minor), 2.63 (dd, J = 4.8, 1.6 Hz, 1H, H-3 major), 2.54 (s, 1H, H-1 major), 2.25 (s, 1H, H-1 minor), 2.23 (s, 1H, H-1 major), 2.16 – 2.06 (m, 2H), 2.05 – 2.02 (m, 1H,

H-6 minor), 2.00 (d, J = 1.8 Hz, 1H, H-6 major), 1.90 – 1.80 (m, 1H), 1.79 – 1.64 (m, 1H), 1.51 – 1.30 (m, 3H), 0.99 (s, 3H, CH_3 minor), 0.93 (s, 3H. CH_3 major), 0.84 (s, 3H, CH_3 major), 0.81 (s, 3H, CH_3 minor).

^{13}C{^{1}H}-APT NMR (101 MHz, $CDCl_3$, HSQC) δ 128.4 (CH_{arom} major), 128.1 (CH_{arom} minor), 126.1 (q, $^3J_{CF}$ = 3.8 Hz, CH_{arom} major + minor), 69.3 (C-2 major), 67.3 (C-2 minor), 48.8 (C-6 minor), 48.3 (C-6 major), 44.5 (C-3 major), 42.3 (C-3 minor), 40.8 (C-7 minor), 39.1 (C-7 major), 38.8 (C-1 major), 37.0 (C-1 minor), 36.8 (C-8 major), 36.8 (C-8 minor), 27.3, 26.8 (CH_3 major), 26.3 (CH_3 minor), 25.3, 24.6, 24.0 (CH_3 major), 23.9, 23.4 (CH_3 minor).

^{19}F{^{1}H}-NMR (376 MHz, $CDCl_3$) δ -63.4 (s, 3F, CF_3), -63.4 (s, 3F, CF_3).

HRMS $[M + H]^+$ calcd. for $C_{17}H_{21}F_3NO_2S^+$ 360.1240; found 360.1239.

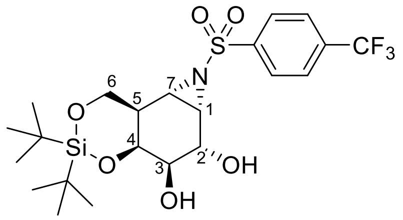

Galactose-cyclophellitol aziridine (**21**). Prepared according to **GP2** from the corresponding alkene. Crude product was purified by flash column chromatography (ethyl acetate/pentane; (1:9 → 1:1) to afford the title compound as a white solid (89 mg, 0.17 mmol, 68%).

^{1}H NMR (500 MHz, $CDCl_3$, H−H COSY, H−H NOESY, HSQC) δ 8.11 (d, J = 8.2 Hz, 2H, Ph), 7.80 (d, J = 8.2 Hz, 2H, Ph), 4.42 – 4.35 (m, 2H, H-4, H-6), 4.21 – 4.14 (m, 2H, H-2, H-6), 3.42 (dd, J = 6.9, 3.8 Hz, 1H, H-1), 3.31 (d, J = 7.5 Hz, 1H, H-3), 3.27 (dt, J = 7.0, 1.3 Hz, 1H, H-7), 2.50 (s, 2H, OH), 2.04 – 1.99 (m, 1H, H-5), 1.05 (s, 9H, CH_3), 1.00 (s, 9H, CH_3).

^{13}C{^{1}H}-APT NMR (126 MHz, $CDCl_3$, HSQC) δ 141.5 ($C_{q\text{-}arom}SO_2$), 135.5 (q, J = 33.3 Hz, $C_{q\text{-}arom}CF_3$), 128.6 (CH_{arom}), 126.4 (q, J = 3.7 Hz, CH_{arom}), 123.2 (d, J = 273.4 Hz, CF_3), 75.4 (C-4), 74.0 (C-3), 68.1 (C-2), 66.7 (C-6), 46.7 (C-1), 43.8 (C-7), 39.7 (C-5), 28.2 (CH_3), 27.1 (CH_3), 23.4 (C_q-tert-butyl), 20.4 (C_q-tert-butyl).

^{19}F{^{1}H}-NMR (376 MHz, $CDCl_3$) δ -63.2 (s, 3F).

HRMS $[M + H]^+$ calcd. for $C_{22}H_{33}F_3NO_6SSi^+$ 524.1745; found 524.1751.

8. Substrate Scope – Low yielding reactions or challenging purifications

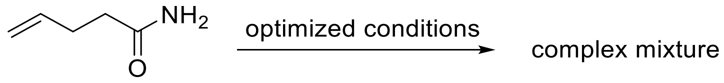

Preparation according to **GP2** from 4-pentenamide. The crude ^{1}H NMR spectrum indicated conversion of the substrate alkene but also of several other side products. Attempted purifications by column chromatography gave NMR spectra (Figures S7-S11) which indicated the presence of several compounds.

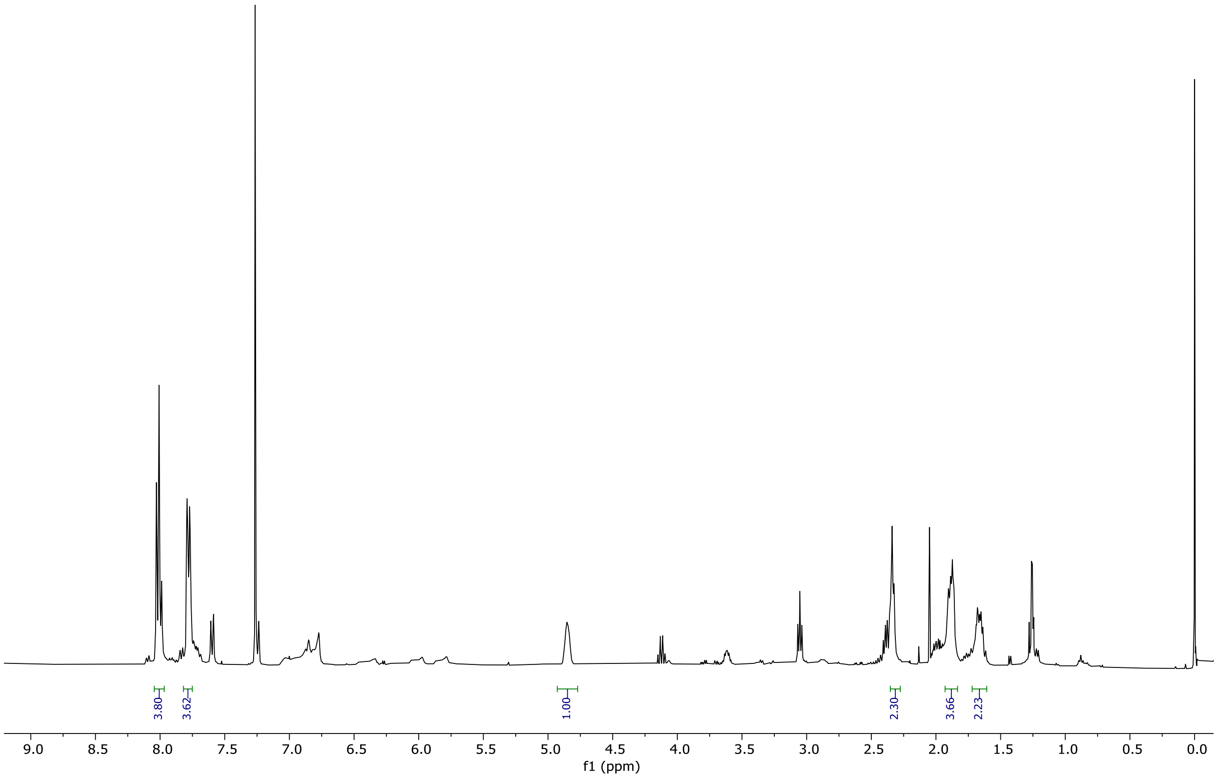

Figure S7. ^{1}H NMR spectrum (400 MHz, $CDCl_3$) of the attempted photocatalytic aziridination reaction on 4-pentenamide after column chromatography. The spectrum shows the presence of multiple products.

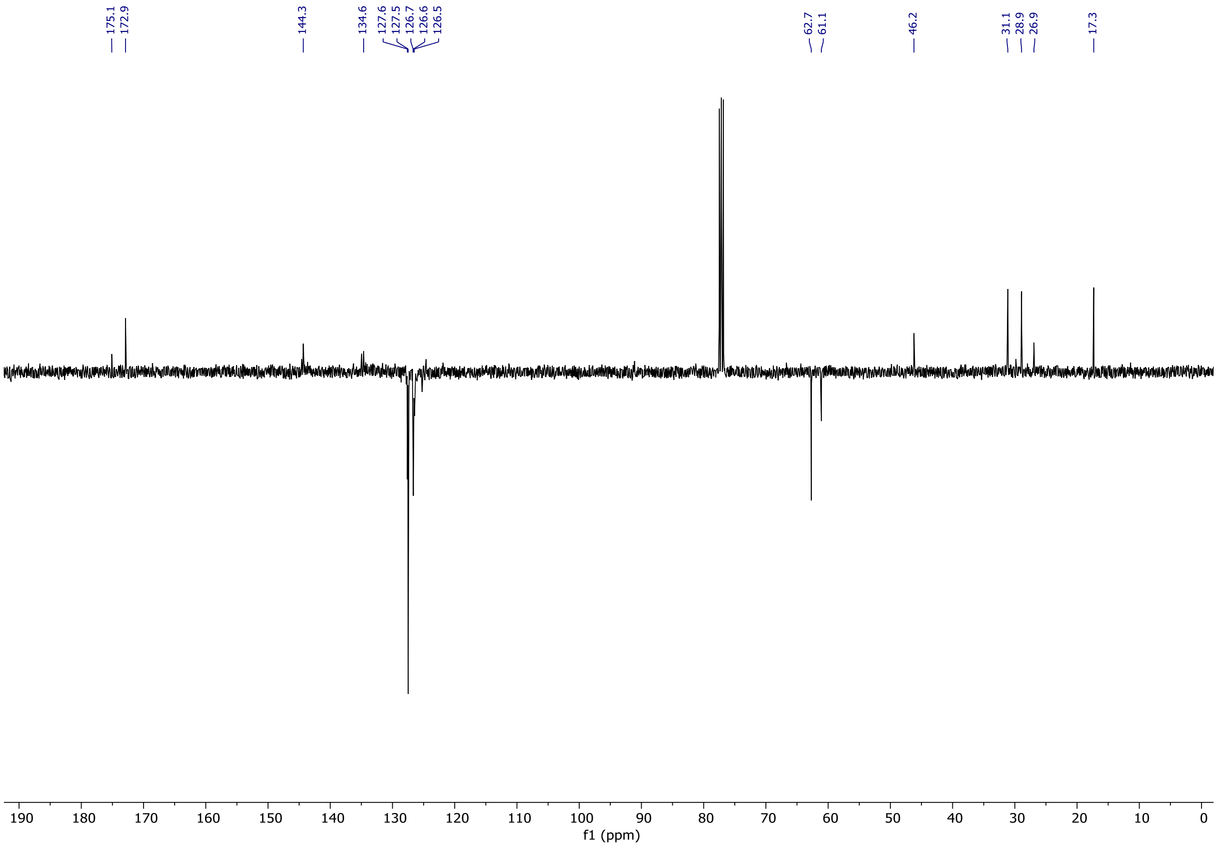

Figure S8. $^{13}C\{^{1}H\}$-APT NMR spectrum (101 MHz, $CDCl_3$) of the attempted photocatalytic aziridination reaction on 4-pentenamide after column chromatography. The spectrum shows the presence of multiple products.

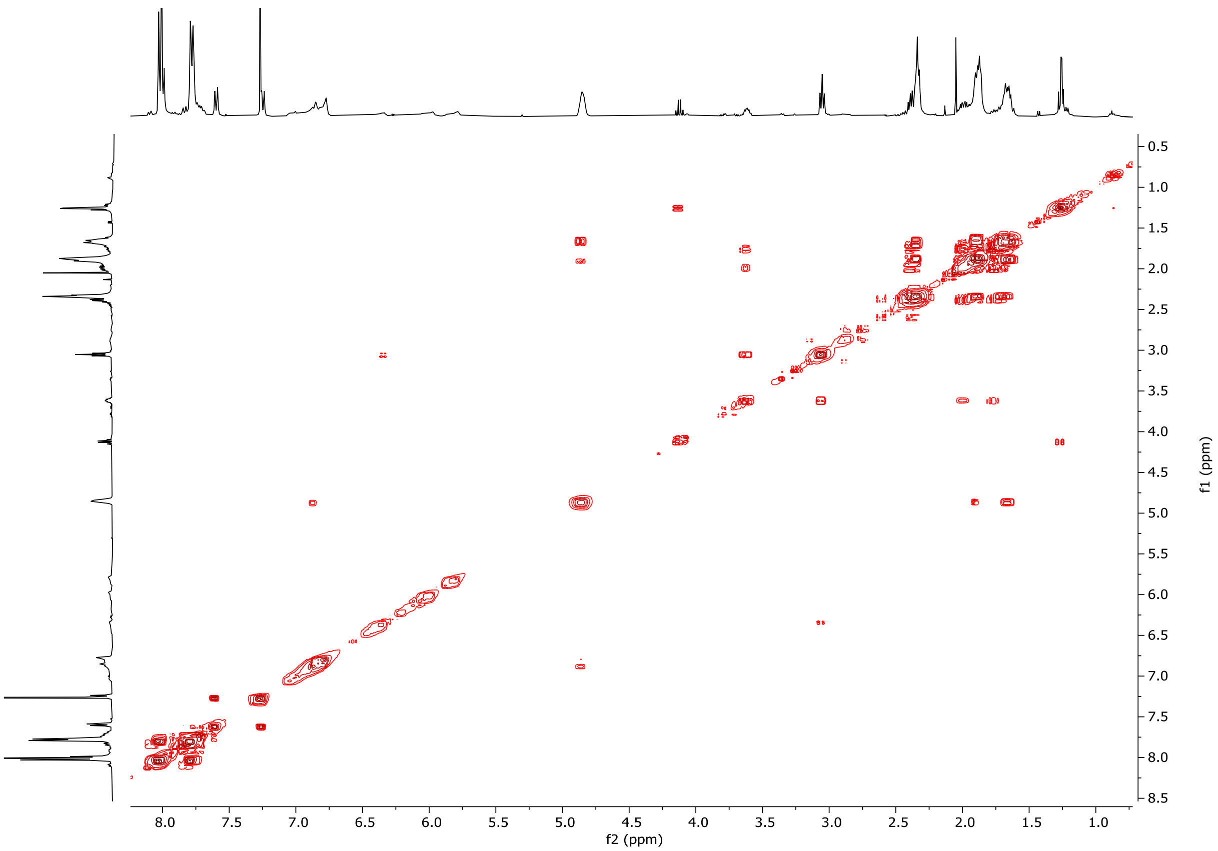

Figure S9. H–H COSY NMR spectrum in $CDCl_3$ of the attempted photocatalytic aziridination reaction on 4-pentenamide after column chromatography. The spectrum shows the presence of multiple products.

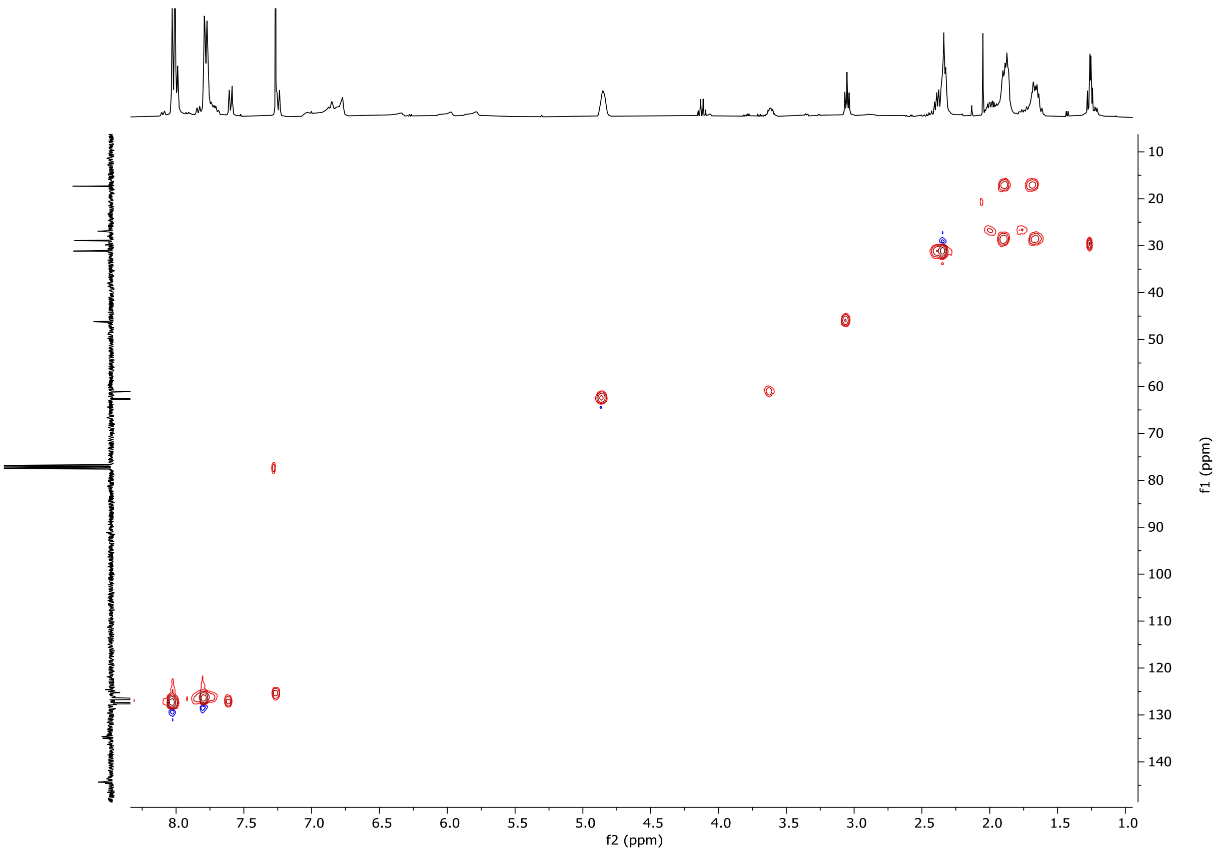

Figure S10. HSQC NMR spectrum in $CDCl_3$ of the attempted photocatalytic aziridination reaction on 4-pentenamide after column chromatography. The spectrum shows the presence of multiple products.

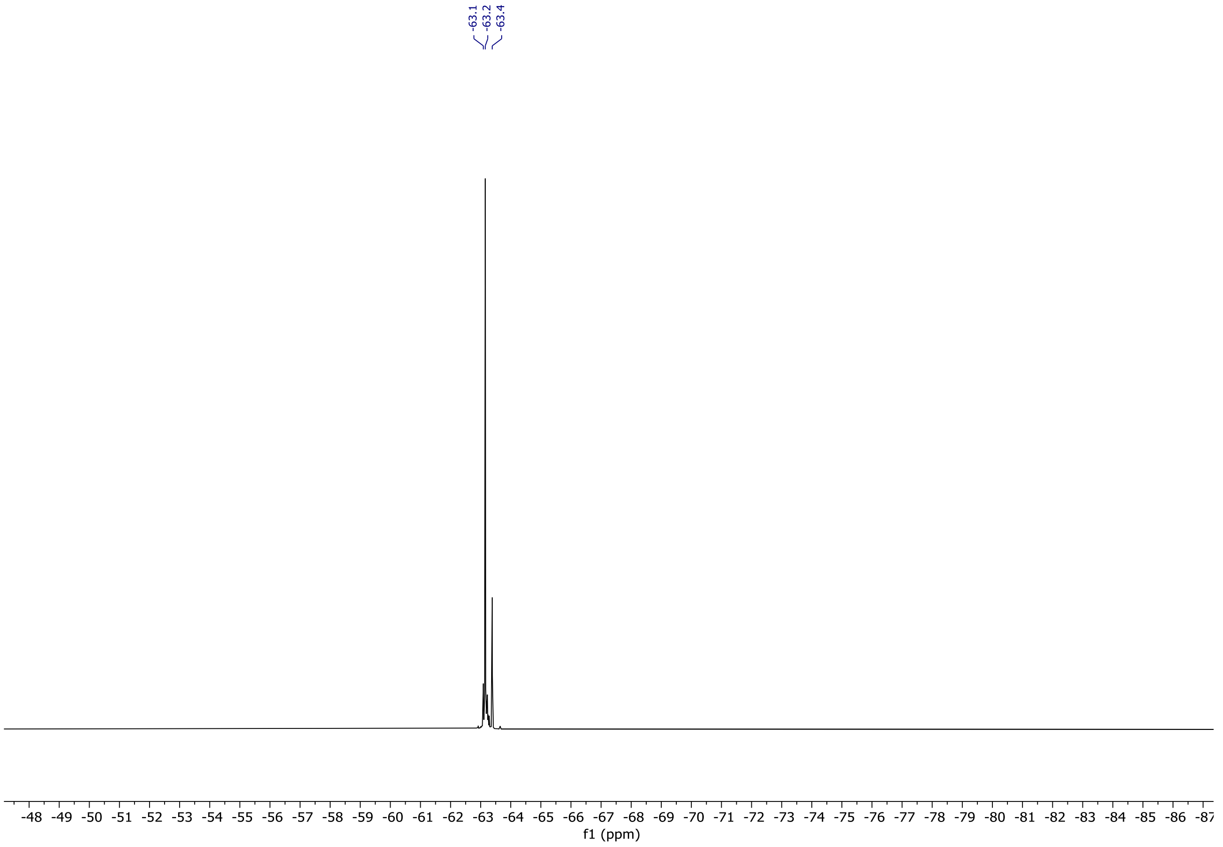

Figure S11. $^{19}F\{^{1}H\}$-NMR spectrum (376 MHz, $CDCl_3$) of the attempted photocatalytic aziridination reaction on 4-pentenamide after column chromatography. The spectrum shows the presence of multiple products.

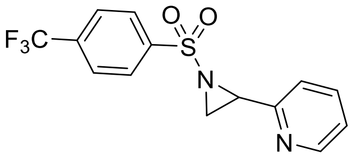

2-(1-((4-(trifluoromethyl)phenyl)sulfonyl)aziridin-2-yl)pyridine (**S1**). Prepared according **GP2** from 2-vinylpyridine. Crude purified by column chromatography (ethyl acetate/pentane; 2.0:8.0 → 5.0:5.0) to give the title compound as a brown oil with impurities (15 mg, 0.046 mmol, ~18%). Despite the impurities the structure could be assigned. The NMR spectra are displayed in Figures S12-S16.

^{1}H NMR (400 MHz, $CDCl_3$, H-H COSY, HSQC) δ 8.57 (ddd, J = 4.9, 1.8, 0.9 Hz, 1H, H_{arom}), 8.15 (dp, *J* = 7.8, 0.8 Hz, 2H, H_{arom}), 7.88 – 7.76 (m, 4H, H_{arom}), 7.69 (td, *J* = 7.7, 1.8 Hz, 1H, H_{arom}), 7.31 (dt, *J* = 7.9, 1.1 Hz, 1H, H_{arom}), 7.26 (ddd, *J* = 7.6, 4.9, 1.2 Hz, 1H, H_{arom}), 4.03 (dd, *J* = 7.2, 4.5 Hz, 1H, NC*H*), 3.09 (d, *J* = 7.2 Hz, 1H, NCH*H*), 2.80 (d, *J* = 4.5 Hz, 1H, NCH*H*).

^{13}C {^{1}H}-APT NMR (101 MHz, $CDCl_3$, HSQC) δ 149.90 (CH_{arom}), 137.05 (CH_{arom}), 128.71 (CH_{arom}), 127.14 (CH_{arom}), 126.41 (q, $^3J_{CF}$ = 3.8 Hz, CH_{arom}) , 123.69 (CH_{arom}), 122.10 (CH_{arom}), 41.79 (N*C*H), 35.46 (NCH_2).

^{19}F{^{1}H}-NMR (376 MHz, $CDCl_3$) -63.2 (s, 3F, CF_3).

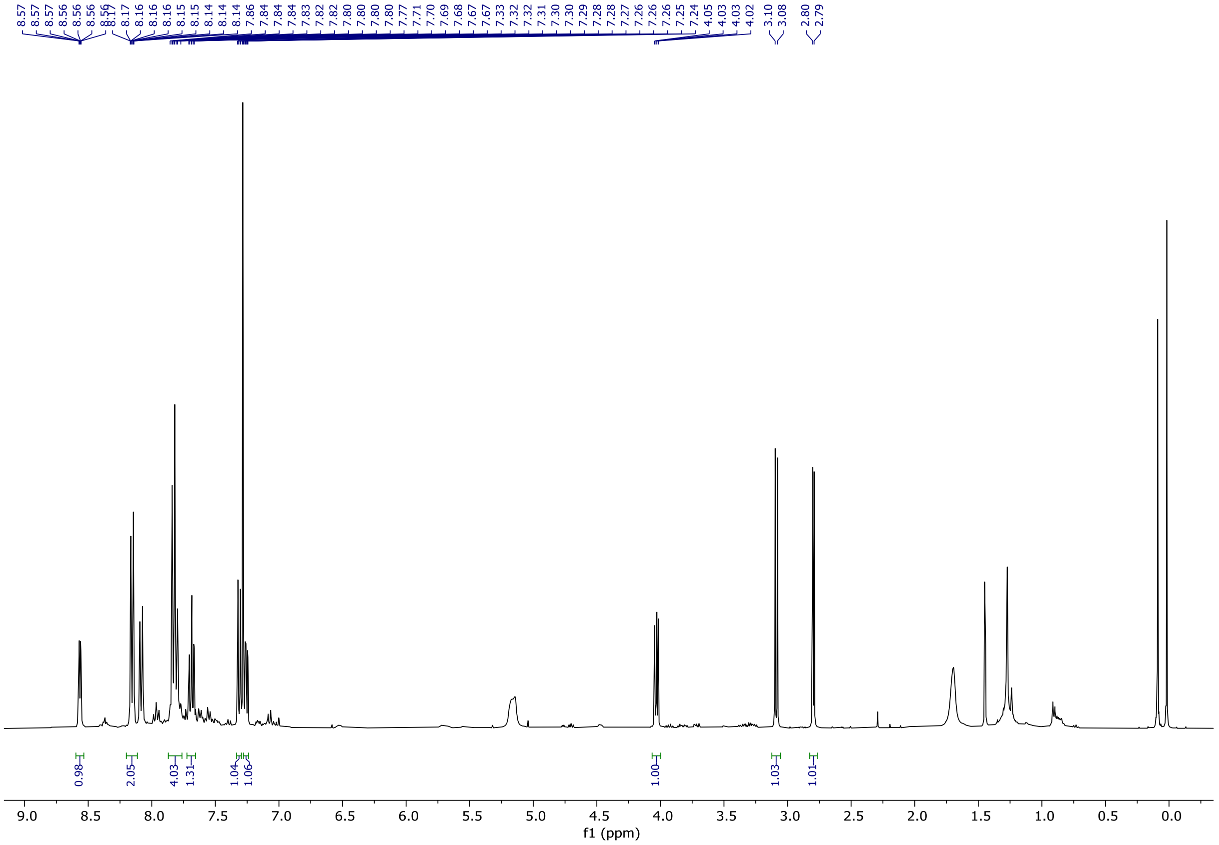

Figure S12. ^{1}H NMR spectrum (400 MHz, $CDCl_3$) of **S1** after column chromatography. The spectrum shows the presence of some impurities.

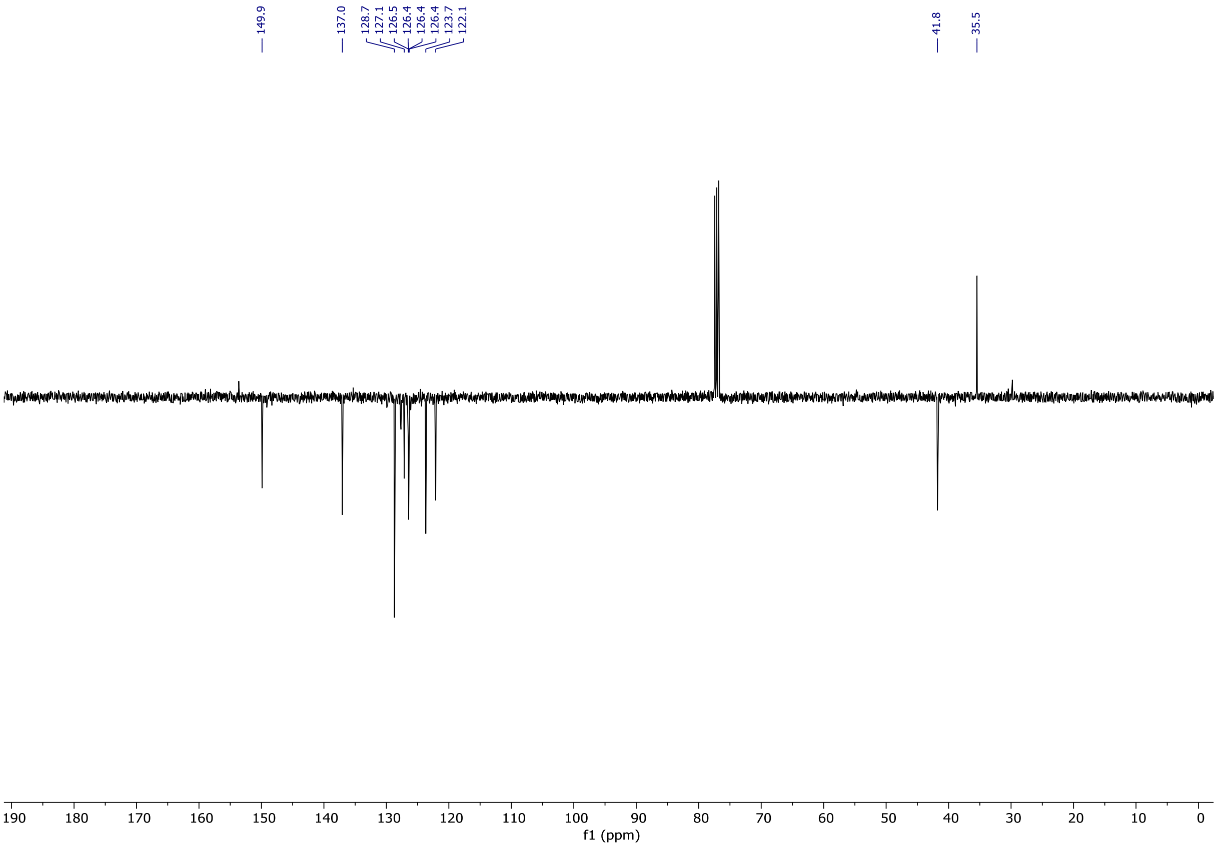

Figure S13. $^{13}C\{^1H\}$-APT NMR spectrum (101 MHz, $CDCl_3$) of **S1** after column chromatography. The spectrum shows the presence of some impurities.

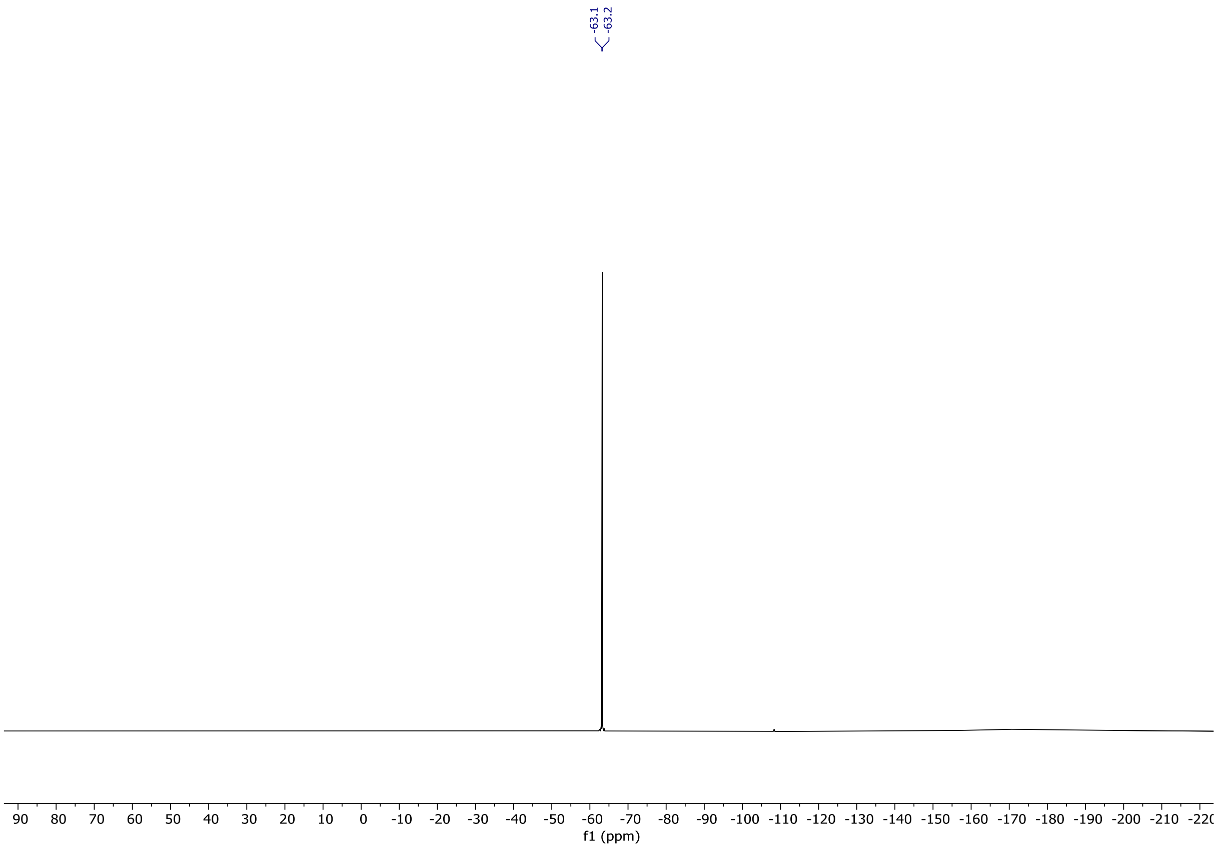

Figure S14. $^{19}F\{^1H\}$-NMR spectrum (376 MHz, $CDCl_3$) of **S1** after column chromatography. The spectrum shows the presence of some impurities.

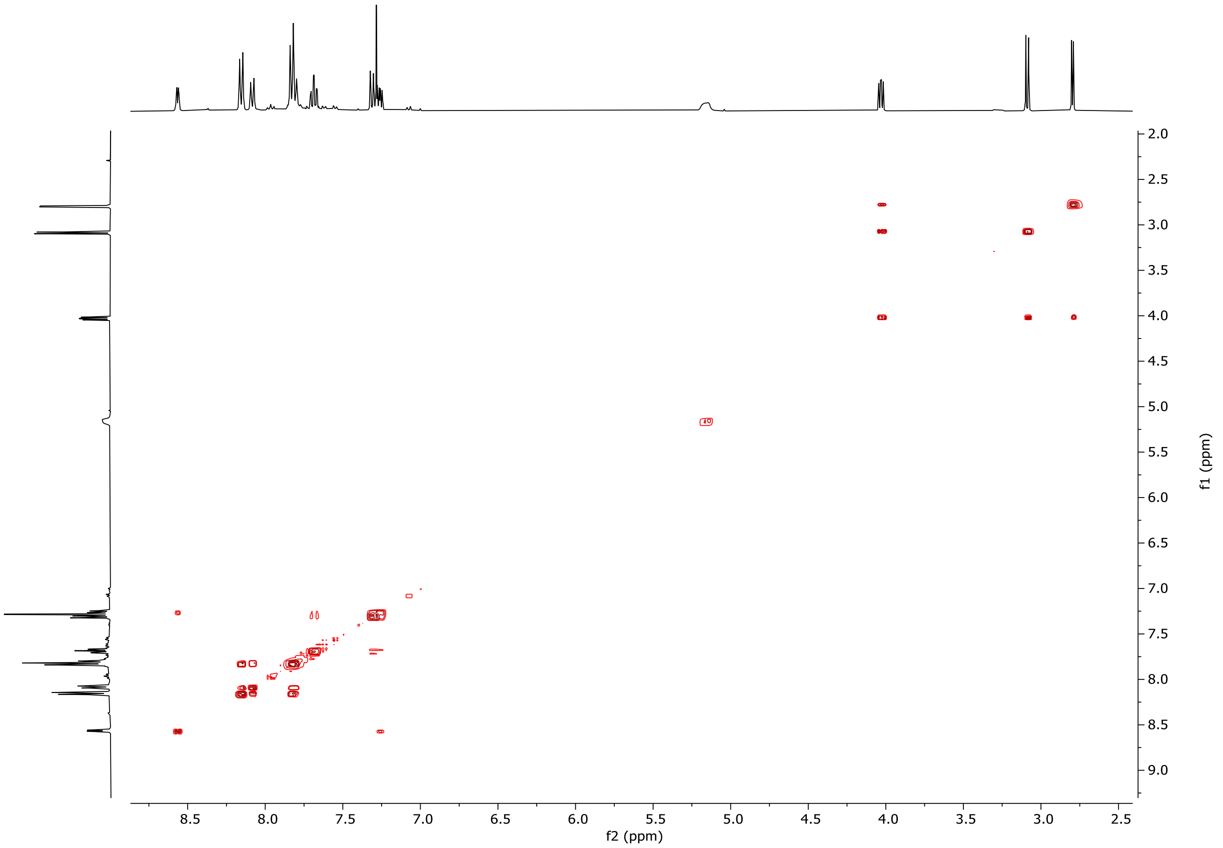

Figure S15. H–H COSY NMR spectrum in $CDCl_3$ of **S1** after column chromatography. The spectrum shows the presence of some impurities.

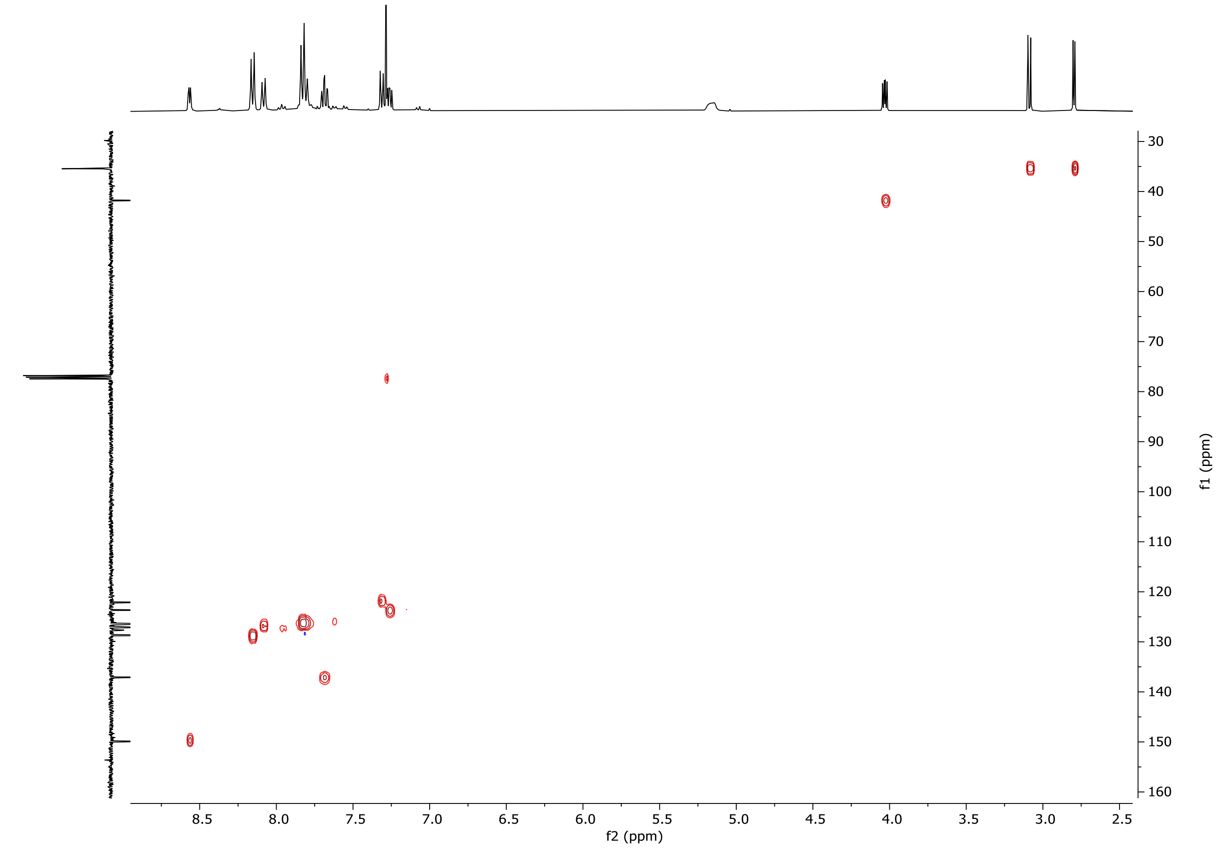

Figure S16. HSQC NMR spectrum in $CDCl_3$ of **S1** after column chromatography. The spectrum shows the presence of some impurities.

9. Scale-up experiment

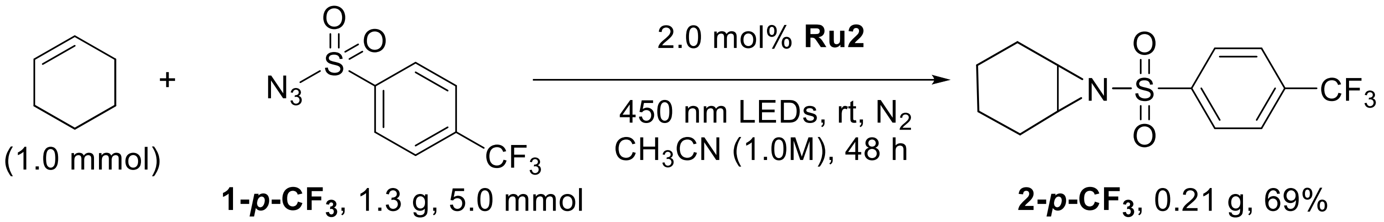

In similar fashion to GP1, a Schlenk tube was charged with a PTFE stir bar, **Ru2** (17 mg, 0.020 mmol, 0.020 eq.) and **1-*p*-CF₃** (1.3 g, 5.0 mmol, 5.0 eq.). Then, dry MeCN (1.0 mL) was added, followed by cyclohexene (0.10 mL, 1.0 mmol, 1.0 eq.) that had been passed over a short alumina column. The reaction mixture was subjected to three freeze-pump-thaw cycles ending on dinitrogen. The Schlenk tube was then positioned in the photoreactor and irradiated for 48 hours under constant stirring. The reaction mixture was concentrated and the crude product was purified by flash column chromatography (diethyl ether/pentane; 0.0:1.0 → 1.0:9.0) to give the title compound as a white solid (0.21 g, 0.70 mmol, 69%). Spectral data matched the data described in section 7 for compound **2-*p*-CF₃**.

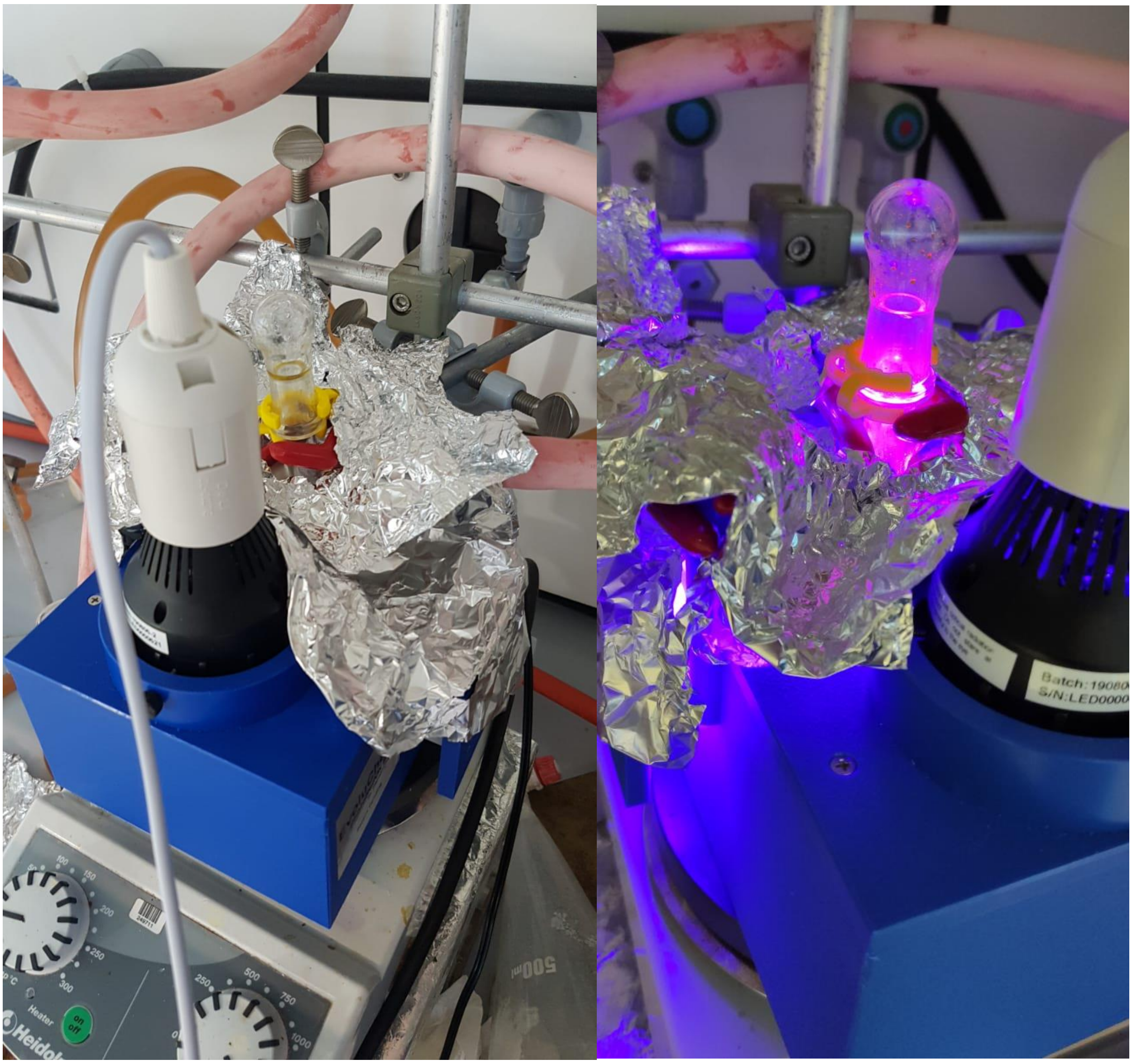

Figure S17. Setup used for the scale-up experiment. **Left**: The Schlenk tube inserted in the PhotoRedOx box™ prior to shining light. **Right**: Reaction whilst irradiating with blue light. The pink color originates from emission of **Ru2** following excitation.

10. References

(1) Kumar, P.; Jiang, T.; Li, S.; Zainul, O.; Laughlin, S. T. Caged Cyclopropenes for Controlling Bioorthogonal Reactivity. *Org. Biomol. Chem.* **2018**, *16* (22), 4081–4085.

(2) Hsu, C.-S.; Gonçalves, C. R.; Tona, V.; Pons, A.; Kaiser, M.; Maulide, N. Leveraging Electron-Deficient Iminium Intermediates in a General Synthesis of Valuable Amines. *Angewandte Chemie International Edition* **2022**, *61* (20), e202115435.

(3) Guevel, A.-C.; Hart, D. J. Synthesis of Carbocycles via Intramolecular Conjugate Additions: Total Syntheses of Axane Sesquiterpenoids. *J. Org. Chem.* **1996**, *61* (2), 473–479.

(4) Song, T.; Arseniyadis, S.; Cossy, J. Highly Enantioselective, Base-Free Synthesis of α-Quaternary Succinimides through Catalytic Asymmetric Allylic Alkylation. *Chemistry – A European Journal* **2018**, *24* (32), 8076–8080.

(5) Jaśkowska, J.; Kowalski, P. N-Alkylation of Imides Using Phase Transfer Catalysts under Solvent-Free Conditions. *Journal of Heterocyclic Chemistry* **2008**, *45* (5), 1371–1375.

(6) Dam, D.; Lagerweij, N. R.; Janmaat, K. M.; Kok, K.; Bouwman, E.; Codée, J. D. C. Organic Dye-Sensitized Nitrene Generation: Intermolecular Aziridination of Unactivated Alkenes. *J. Org. Chem.* **2024**, *89* (5), 3251–3258.

(7) Juris, A.; Balzani, V.; Belser, P.; von Zelewsky, A. Characterization of the Excited State Properties of Some New Photosensitizers of the Ruthenium (Polypyridine) Family. *Helvetica Chimica Acta* **1981**, *64* (7), 2175–2182.

(8) Rehm, D.; Weller, A. Kinetics of Fluorescence Quenching by Electron and H-Atom Transfer. *Israel Journal of Chemistry* **1970**, *8* (2), 259–271.

(9) Prier, C. K.; Rankic, D. A.; MacMillan, D. W. C. Visible Light Photoredox Catalysis with Transition Metal Complexes: Applications in Organic Synthesis. *Chem. Rev.* **2013**, *113* (7), 5322–5363.

(10) Vlcek, A. A.; Dodsworth, E. S.; Pietro, W. J.; Lever, A. B. P. Excited State Redox Potentials of Ruthenium Diimine Complexes; Correlations with Ground State Redox Potentials and Ligand Parameters. *Inorg. Chem.* **1995**, *34* (7), 1906–1913.

(11) Marzo, L.; Pagire, S. K.; Reiser, O.; König, B. Visible-Light Photocatalysis: Does It Make a Difference in Organic Synthesis? *Angewandte Chemie International Edition* **2018**, *57* (32), 10034–10072.

11. NMR Spectra

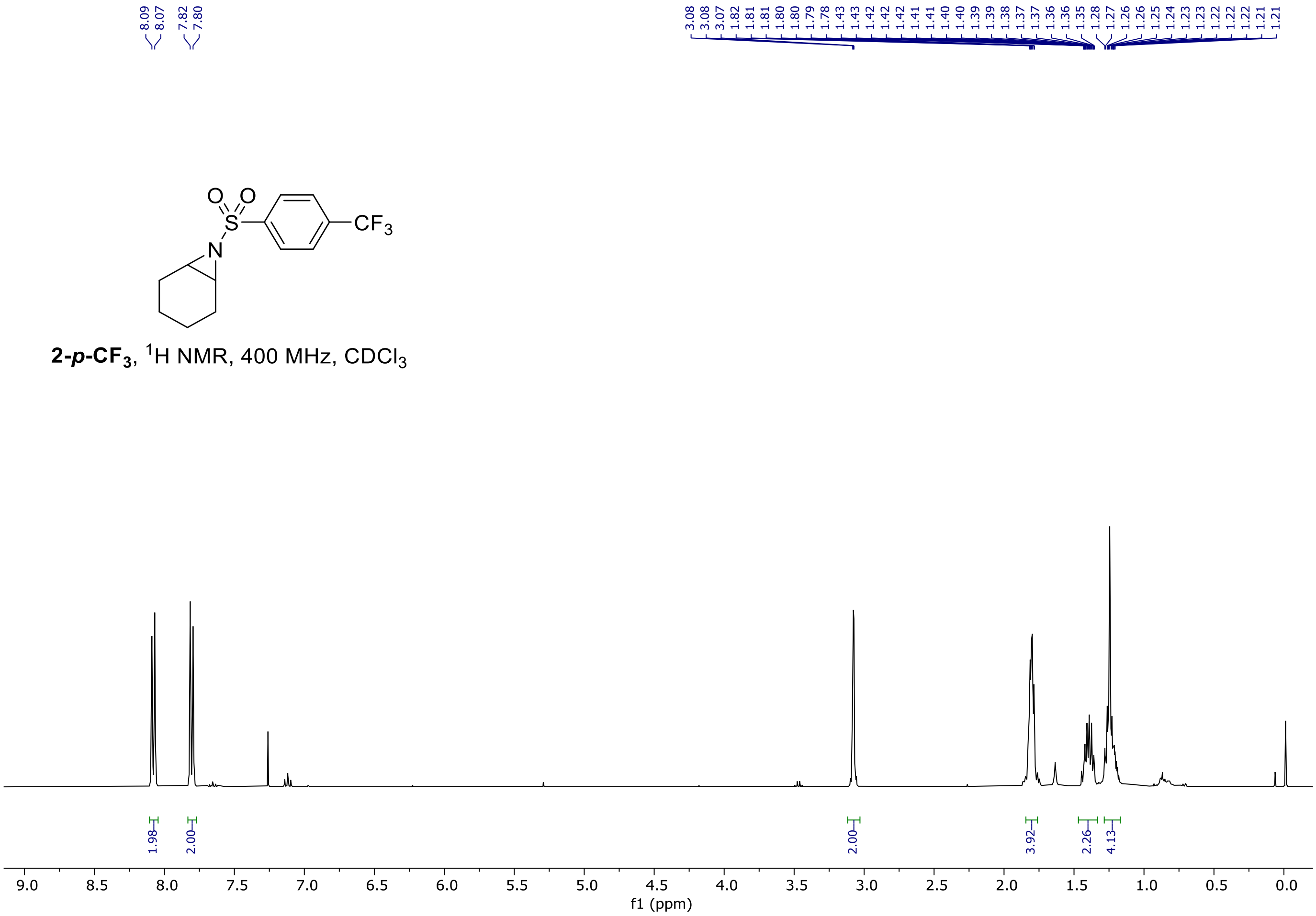

2-*p*-CF$_3$, ^{1}H NMR, 400 MHz, CDCl$_3$

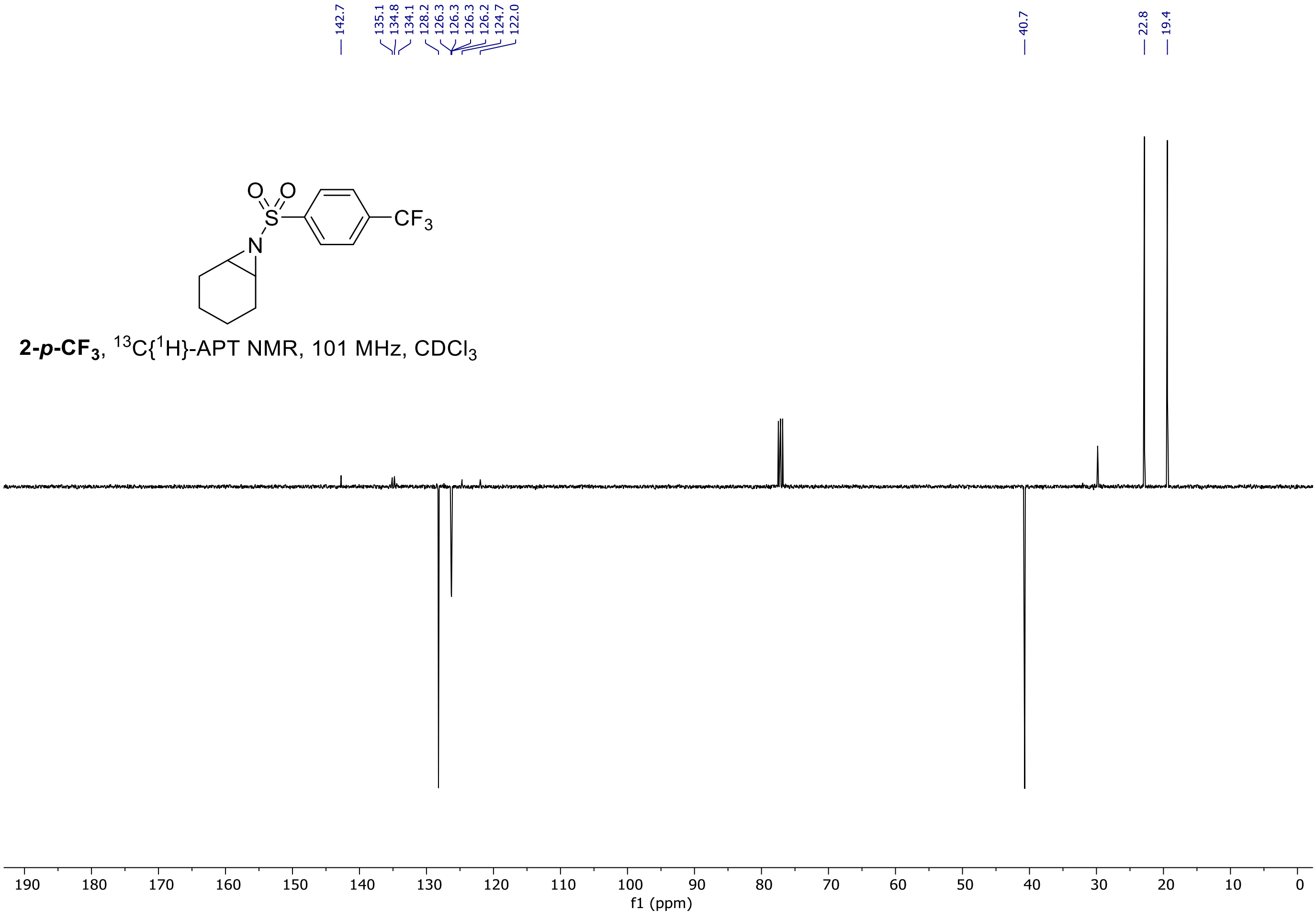

2-*p*-CF$_3$, ^{13}C{^{1}H}-APT NMR, 101 MHz, CDCl$_3$

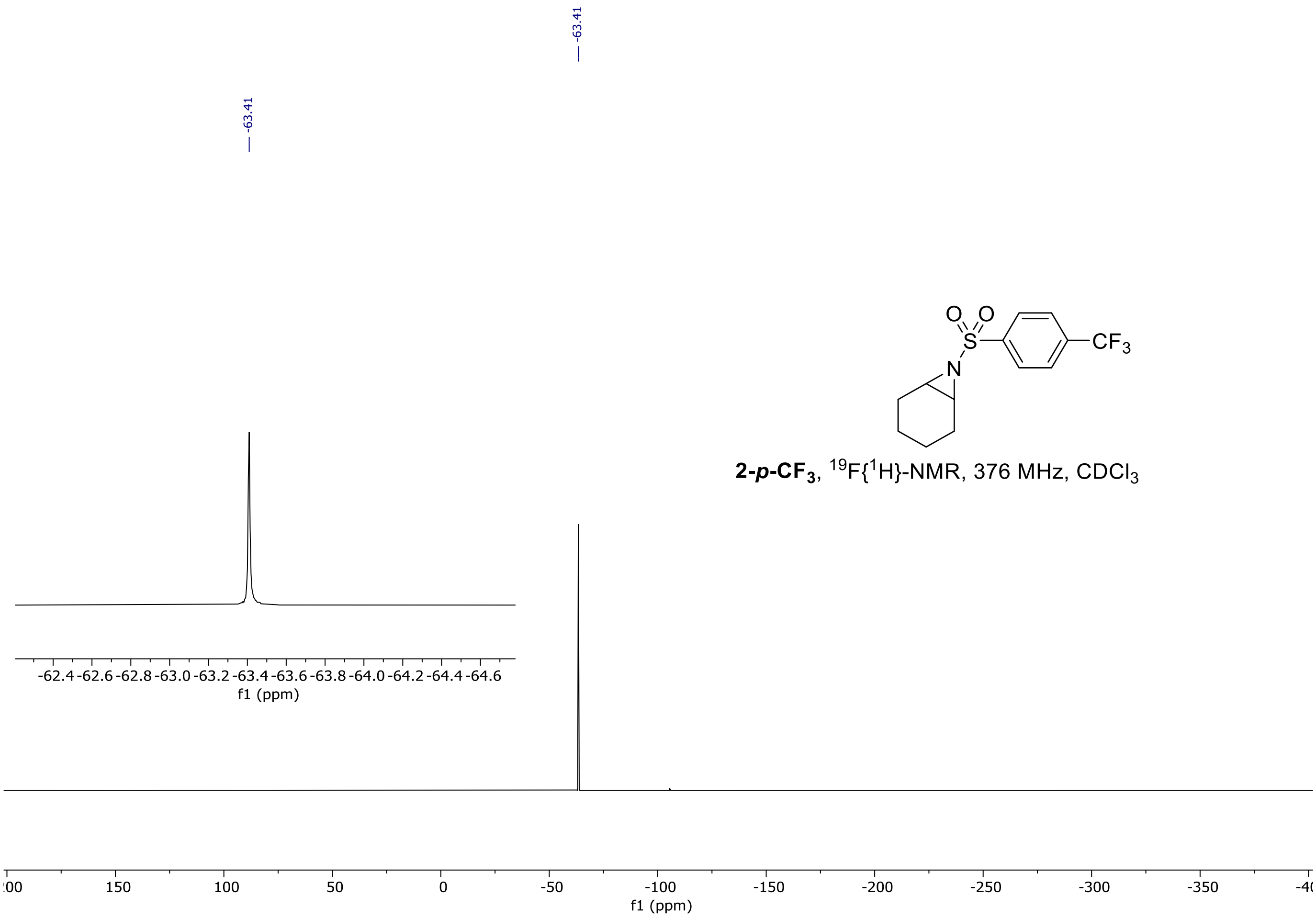
-63.41
-63.41
CF3
2-p-CF3, 19F{1H}-NMR, 376 MHz, CDCl3
-62.4 -62.6 -62.8 -63.0 -63.2 -63.4 -63.6 -63.8 -64.0 -64.2 -64.4 -64.6
f1 (ppm)
150 100 50 0 -50 -100 -150 -200 -250 -300 -350
f1 (ppm)

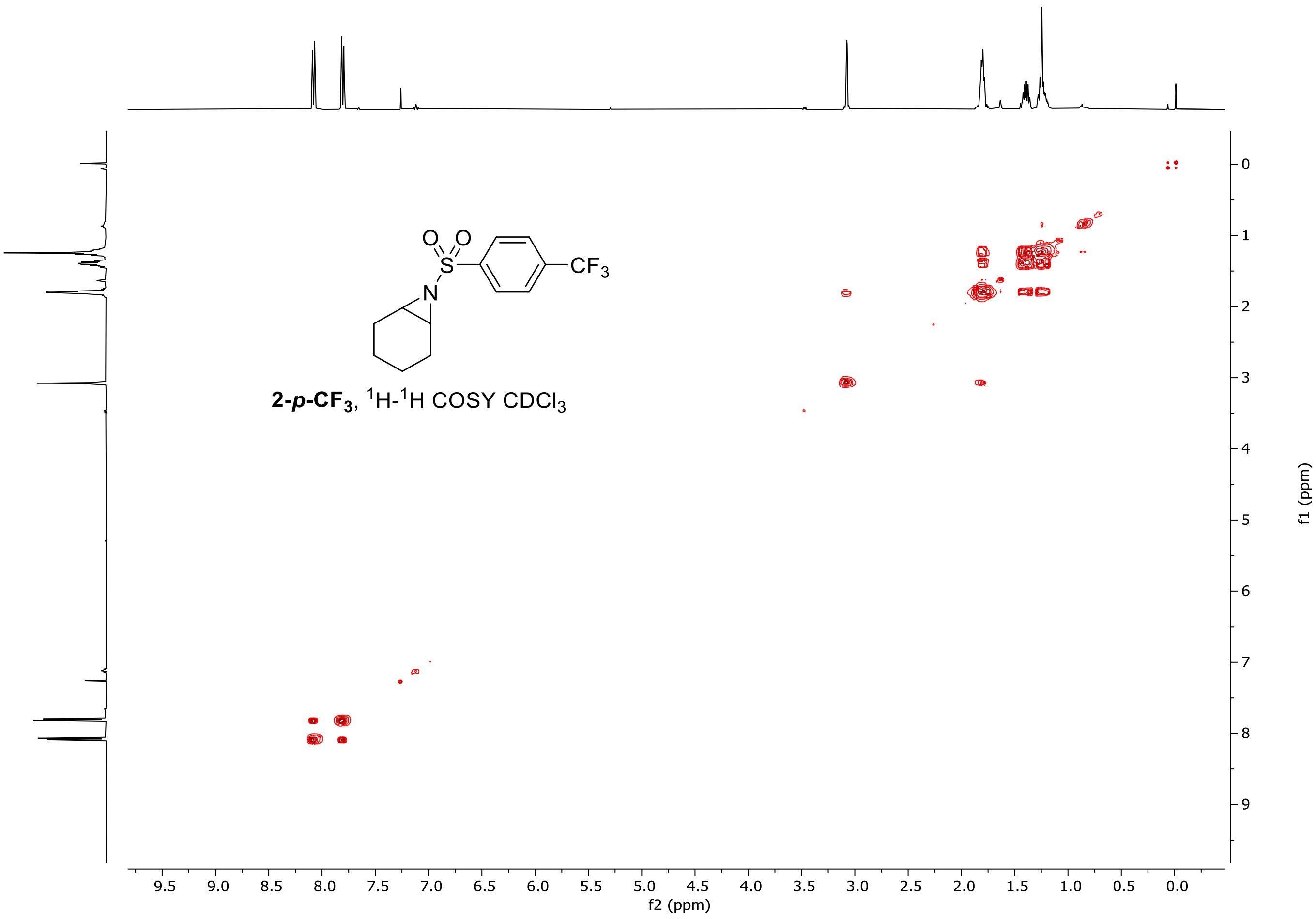
CF3
2-p-CF3, 1H-1H COSY CDCl3
9.5 9.0 8.5 8.0 7.5 7.0 6.5 6.0 5.5 5.0 4.5 4.0 3.5 3.0 2.5 2.0 1.5 1.0 0.5 0.0
f2 (ppm)
0 1 2 3 4 5 6 7 8 9
f1 (ppm)

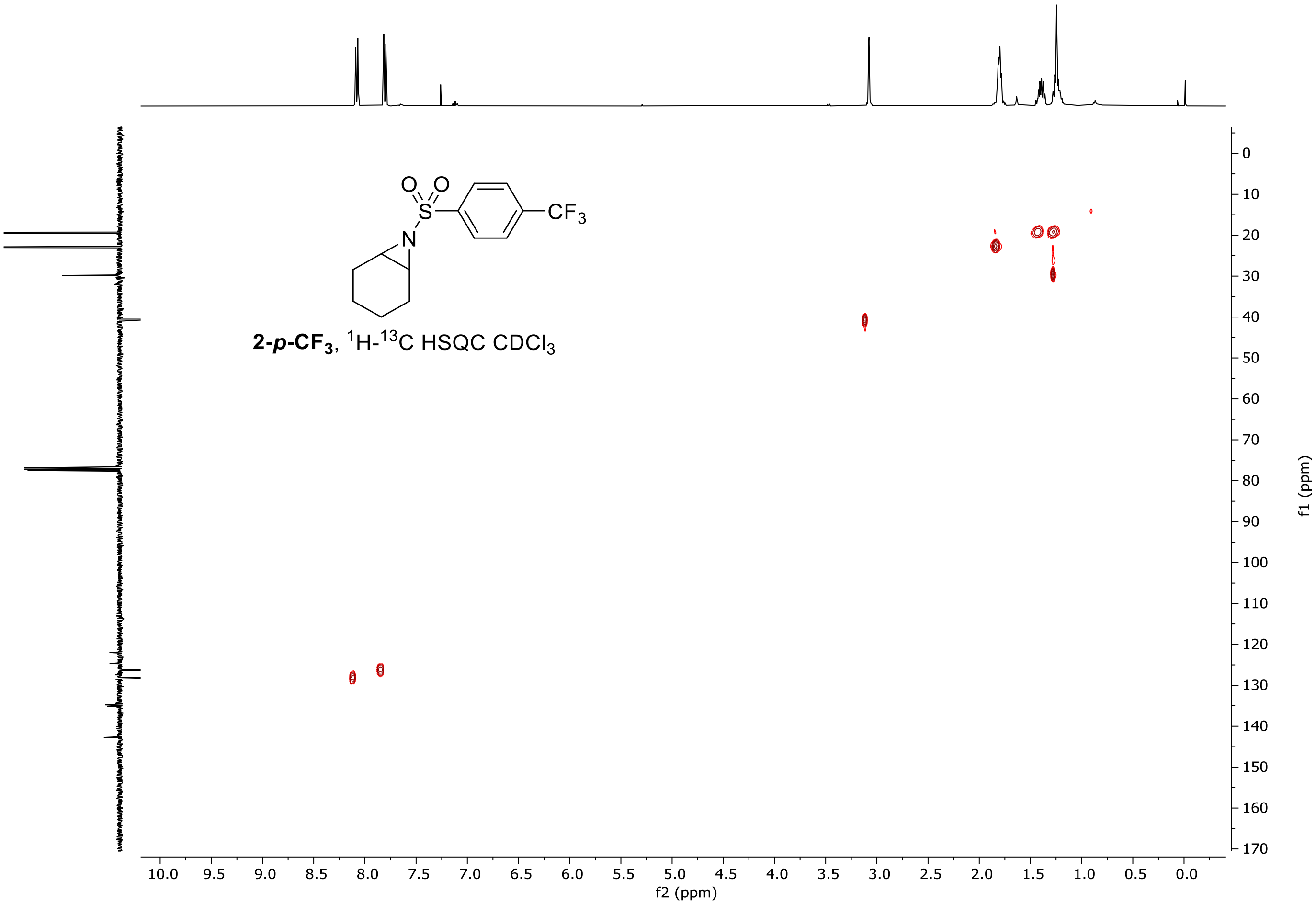
2-*p*-CF3, 1H-13C HSQC CDCl3
CF3
f2 (ppm)
f1 (ppm)

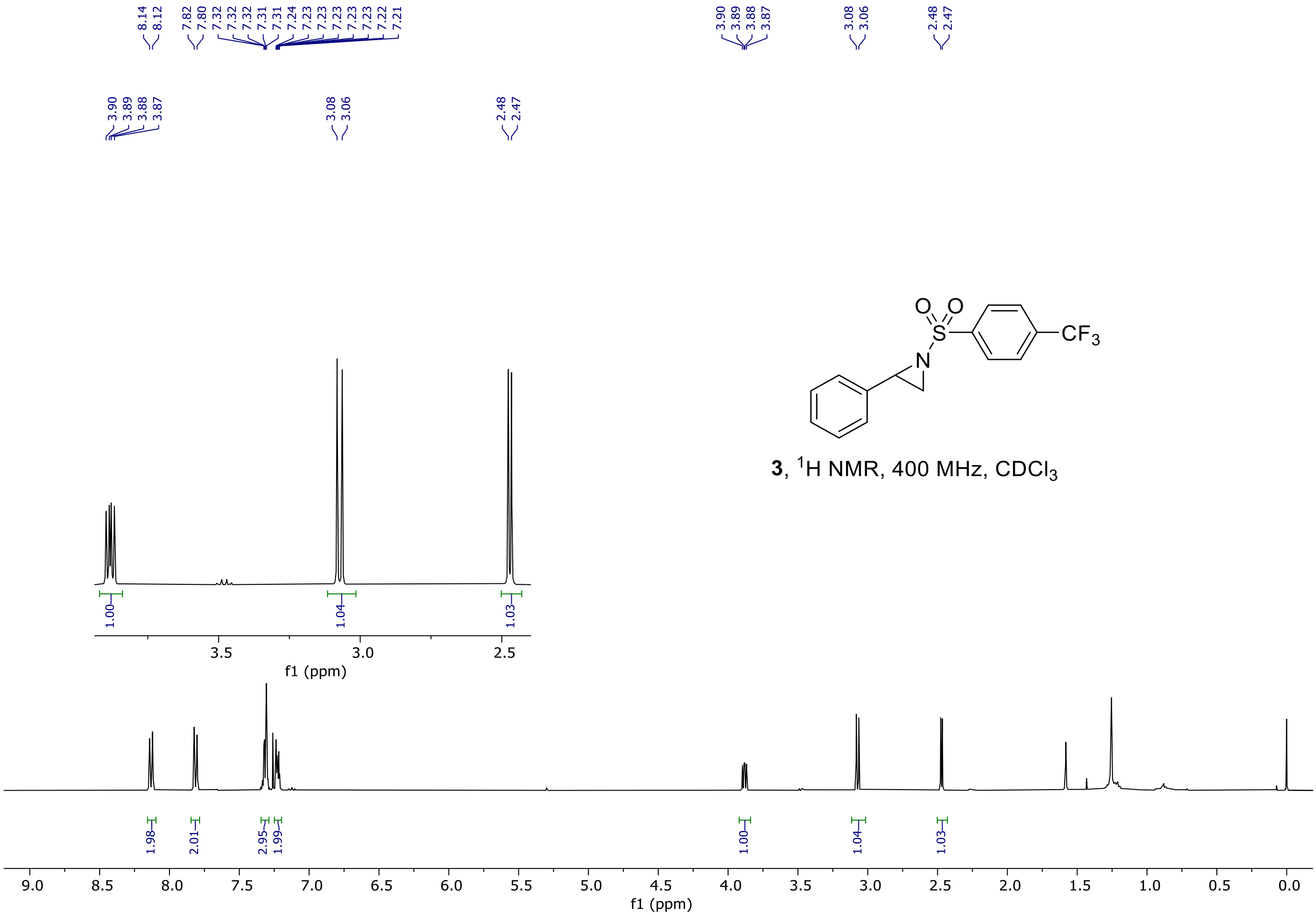
3, 1H NMR, 400 MHz, CDCl3
CF3
8.14 8.12 7.82 7.80 7.32 7.32 7.32 7.31 7.31 7.24 7.23 7.23 7.23 7.23 7.23 7.22 7.21
3.90 3.89 3.88 3.87
3.08 3.06
2.48 2.47
1.98 2.01 2.95 1.99 1.00 1.04 1.03
f1 (ppm)

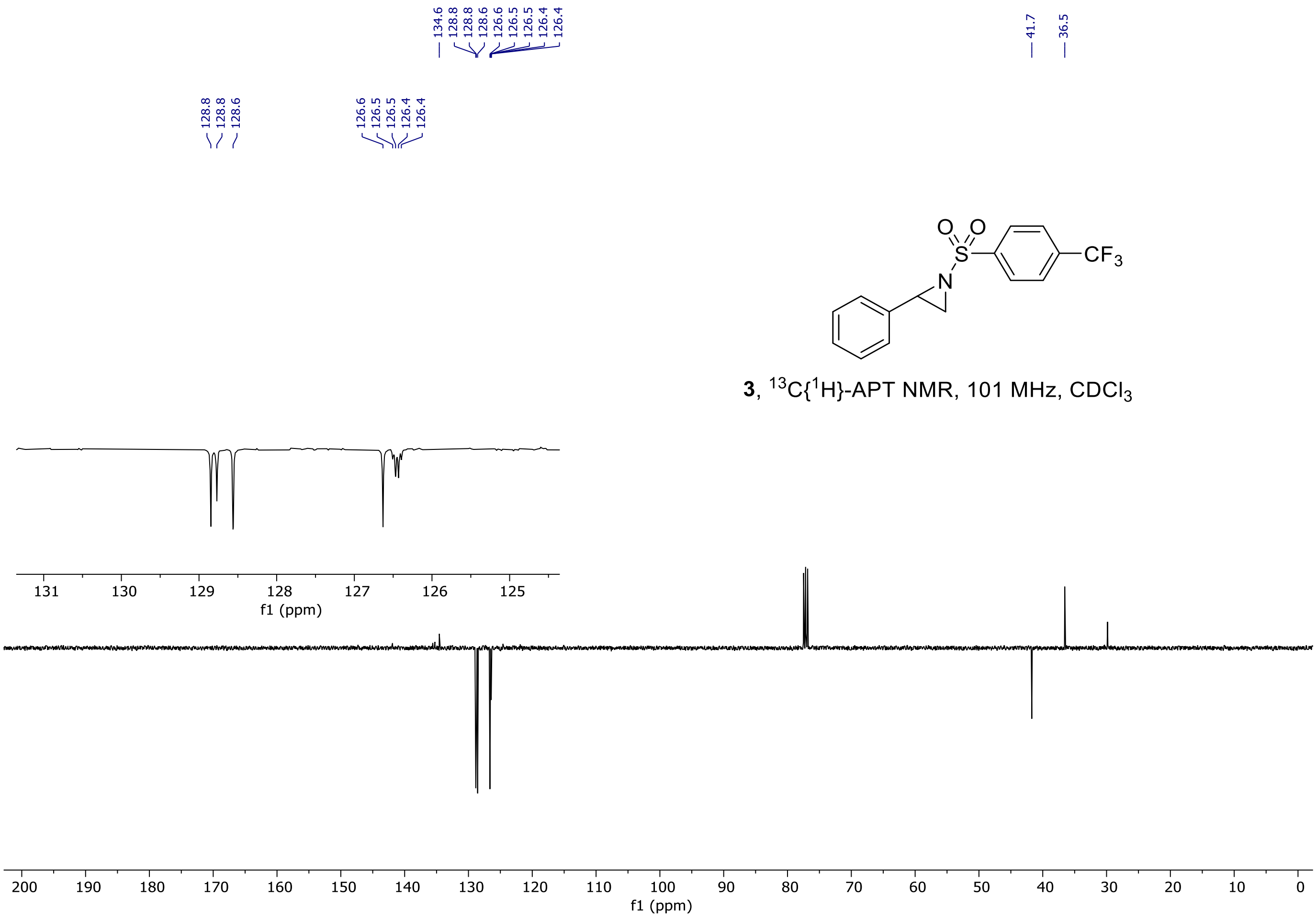
134.6
128.8
128.8
128.6
126.6
126.5
126.5
126.4
126.4
41.7
36.5
3, $^{13}C\{^{1}H\}$-APT NMR, 101 MHz, $CDCl_3$
f1 (ppm)

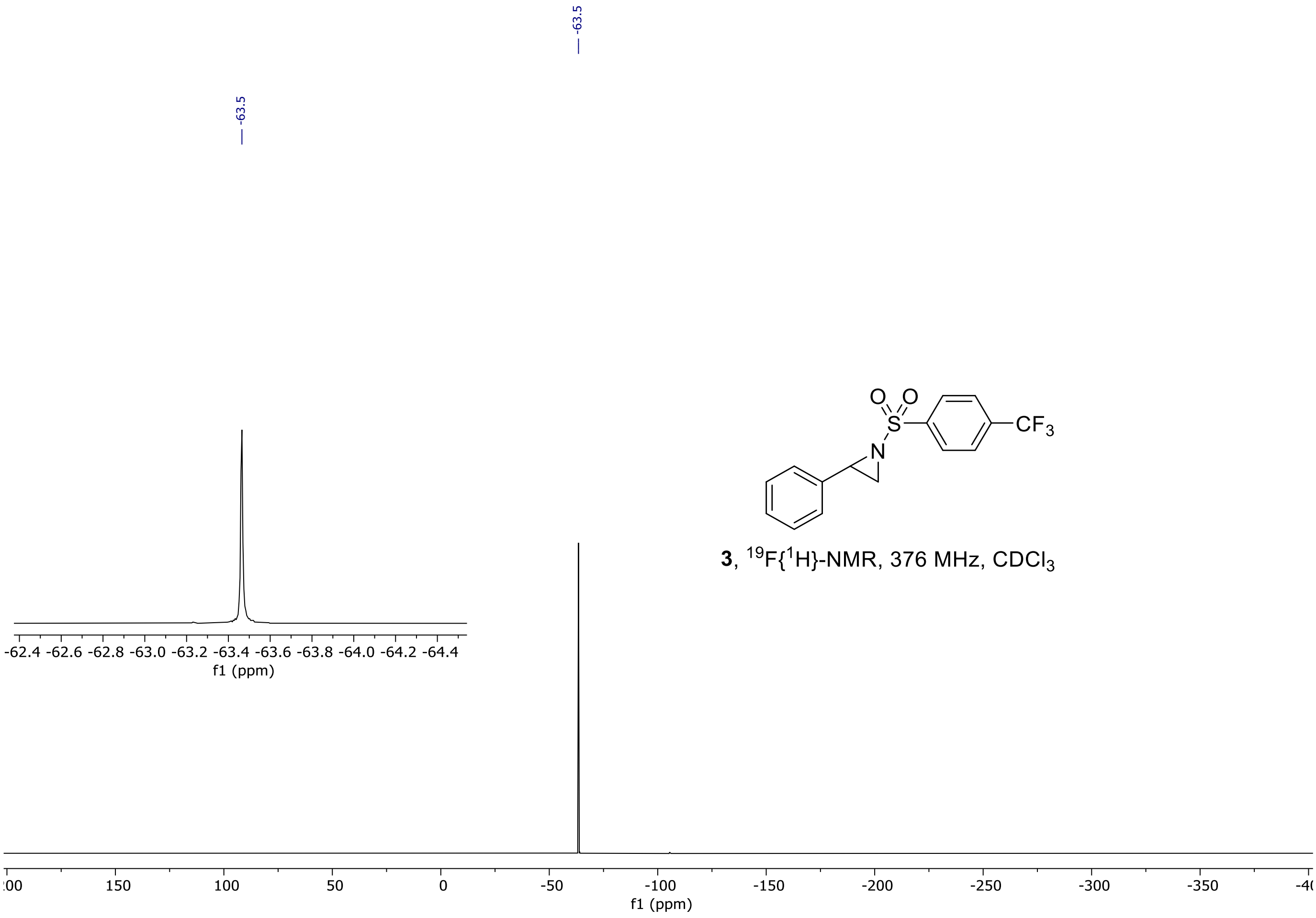
-63.5
3, $^{19}F\{^{1}H\}$-NMR, 376 MHz, $CDCl_3$
f1 (ppm)

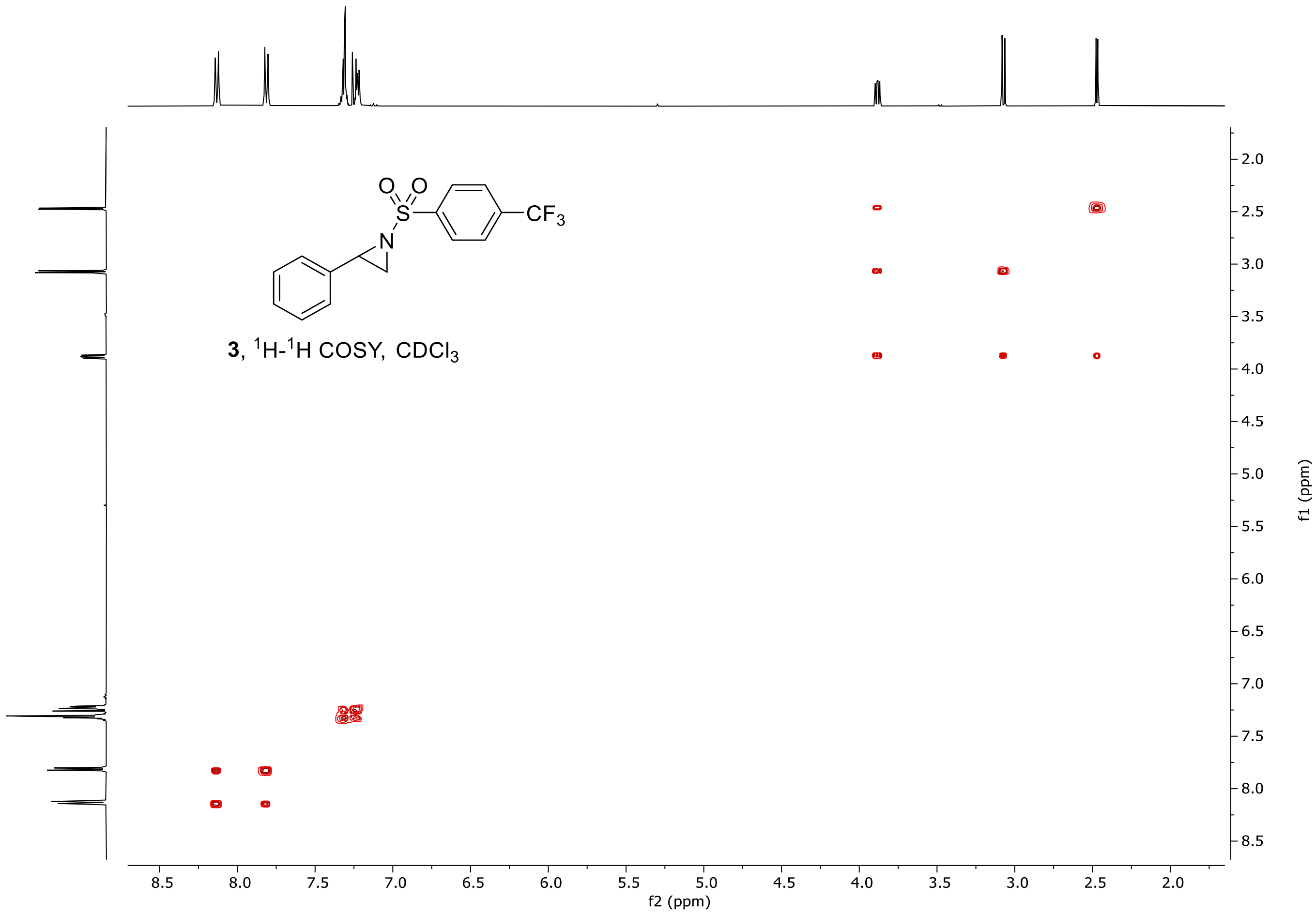

CF3
3, 1H-1H COSY, CDCl3
f2 (ppm)
f1 (ppm)

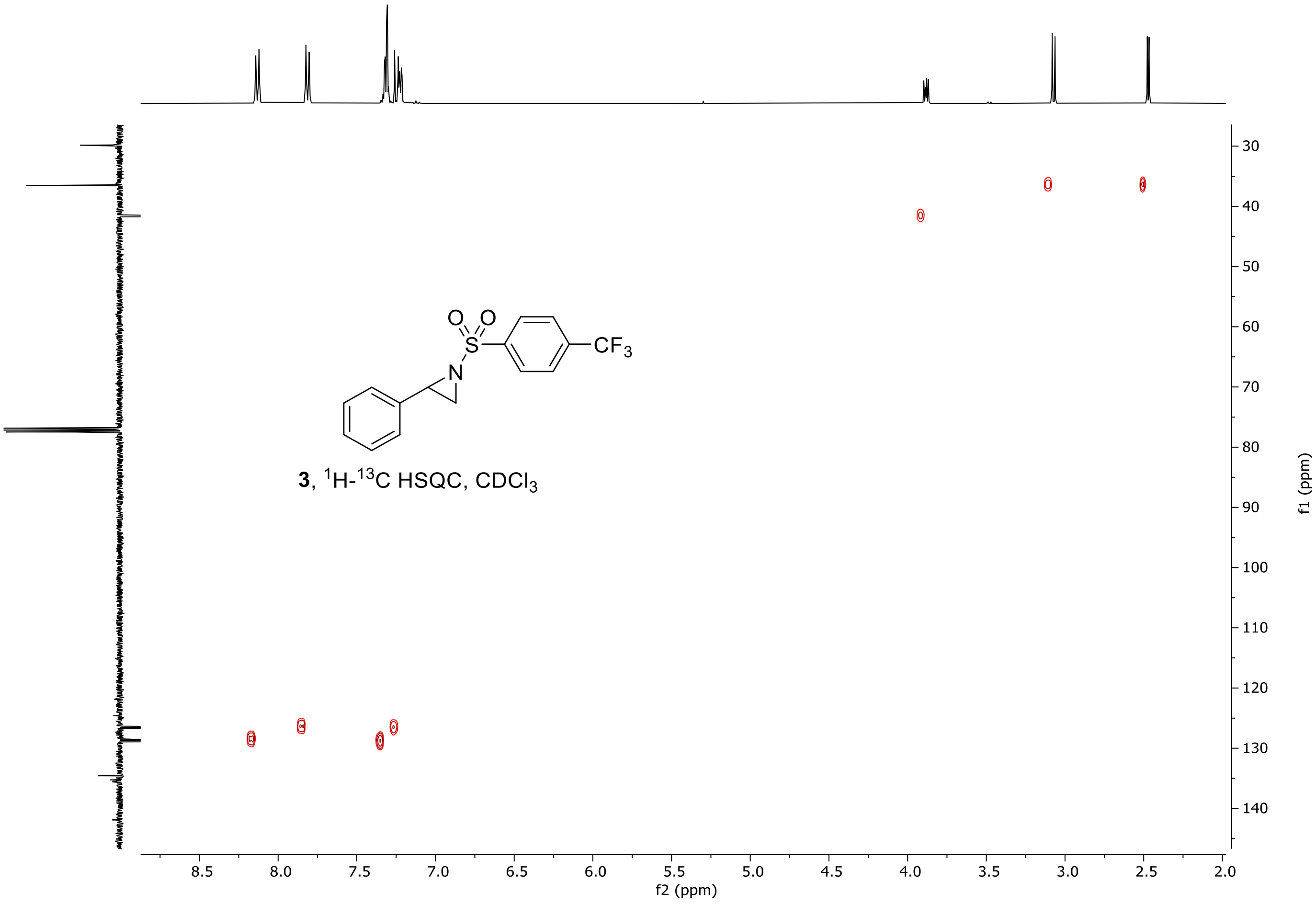

CF3
3, 1H-13C HSQC, CDCl3
f2 (ppm)
f1 (ppm)

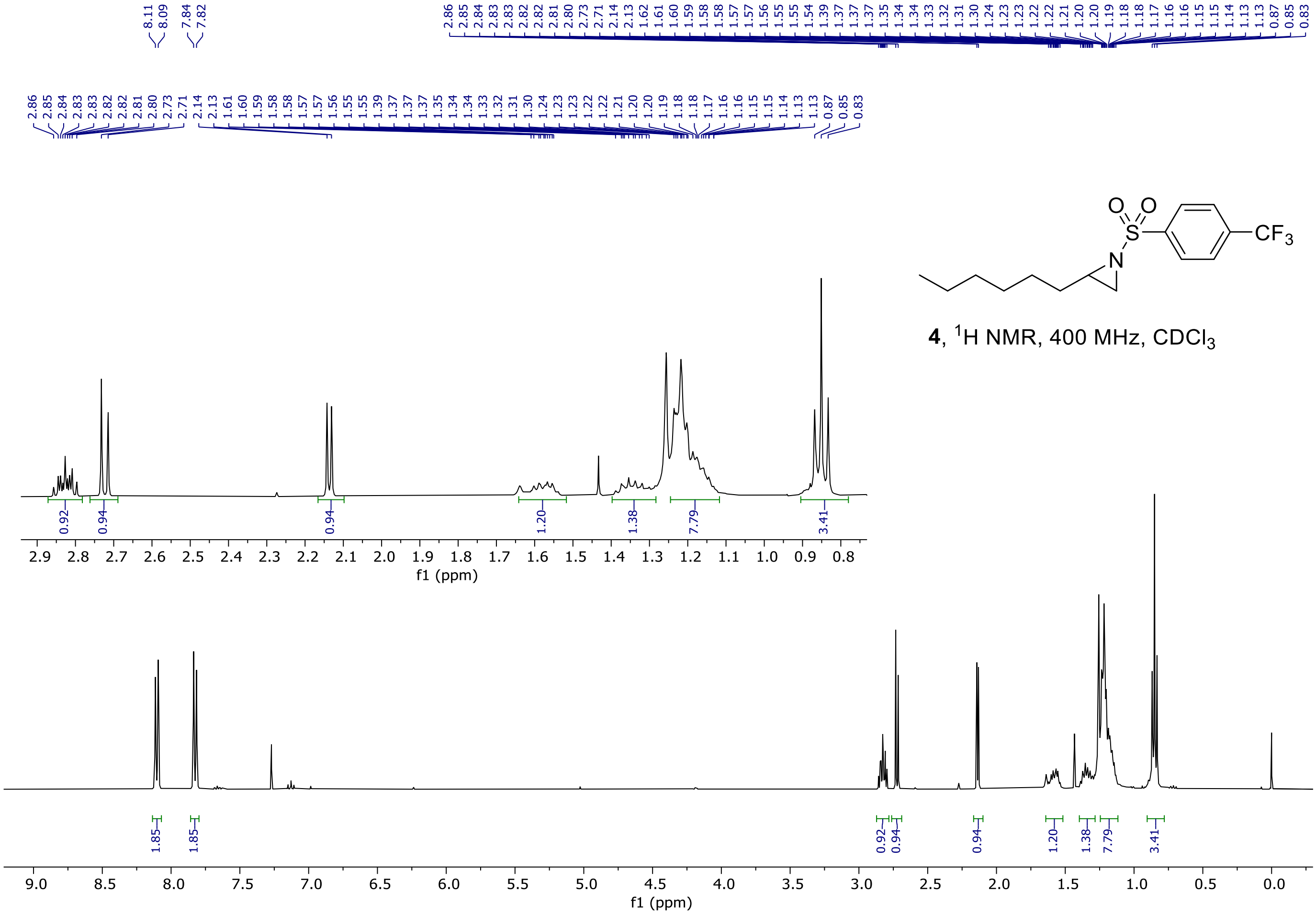
CF3
N
S
O O
4, 1H NMR, 400 MHz, CDCl3
f1 (ppm)

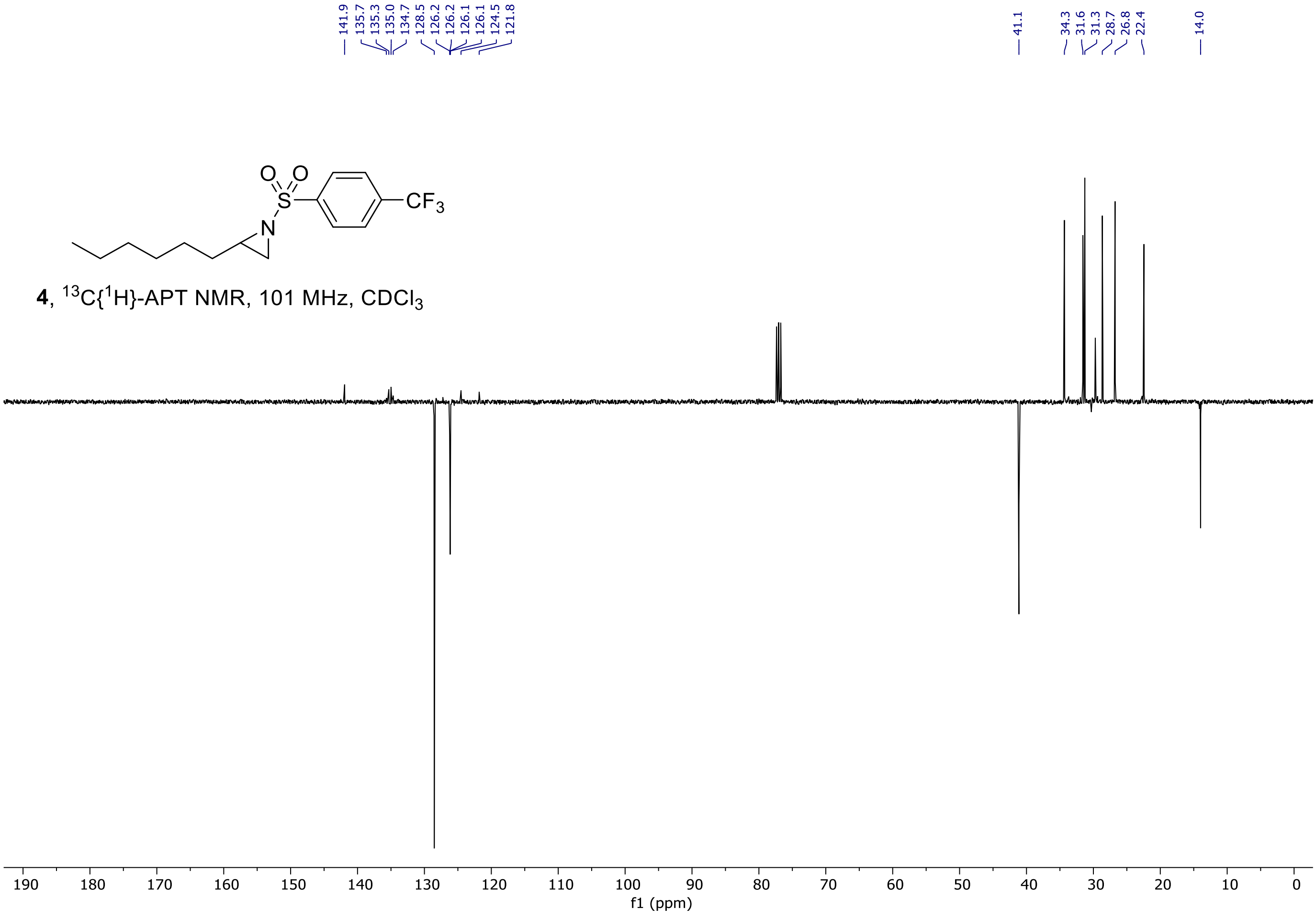
141.9
135.7
135.3
135.0
134.7
128.5
126.2
126.2
126.1
126.1
124.5
121.8
41.1
34.3
31.6
31.3
28.7
26.8
22.4
14.0
CF3
N
S
O O
4, 13C{1H}-APT NMR, 101 MHz, CDCl3
f1 (ppm)

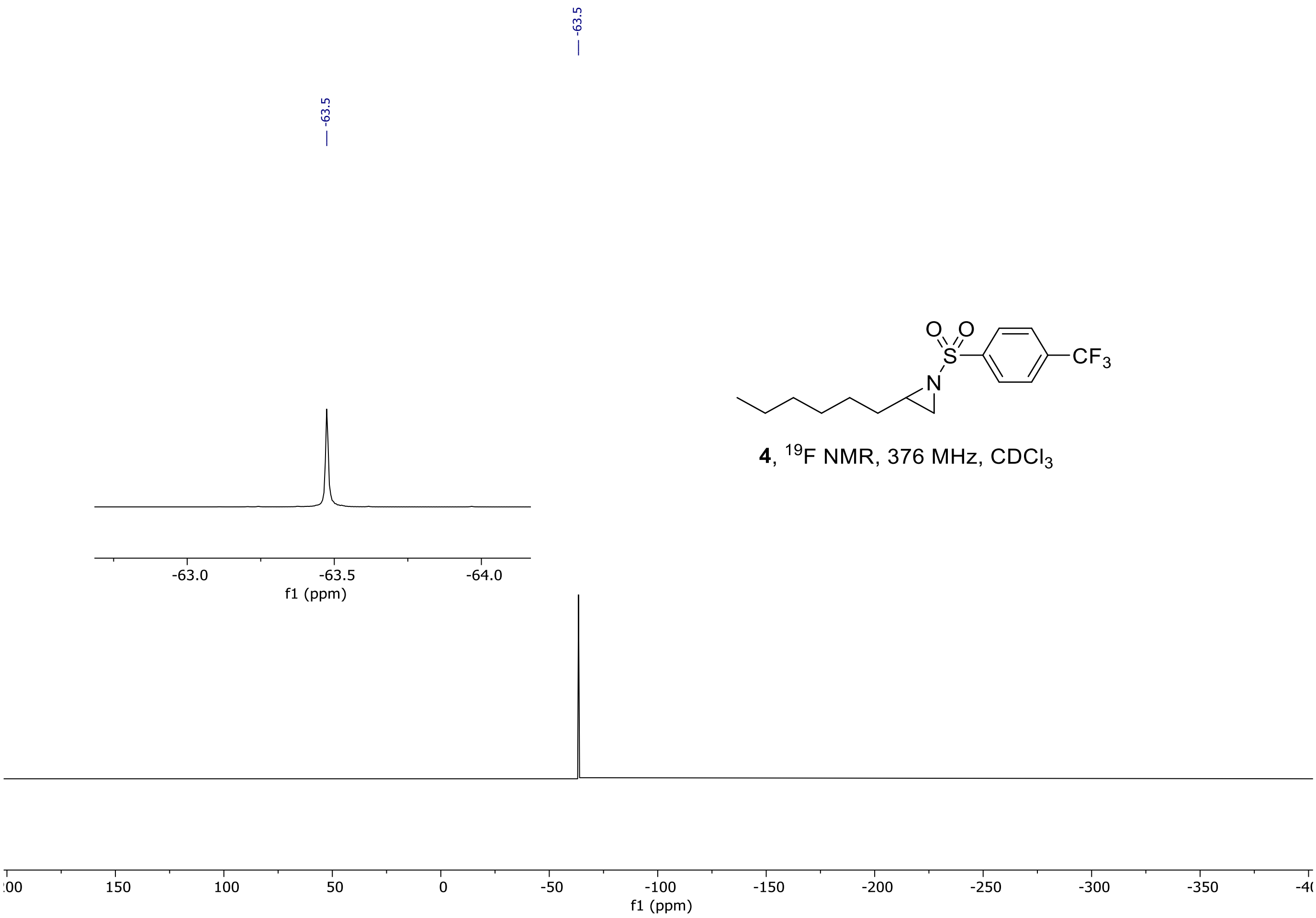

4, ^{19}F NMR, 376 MHz, $CDCl_3$

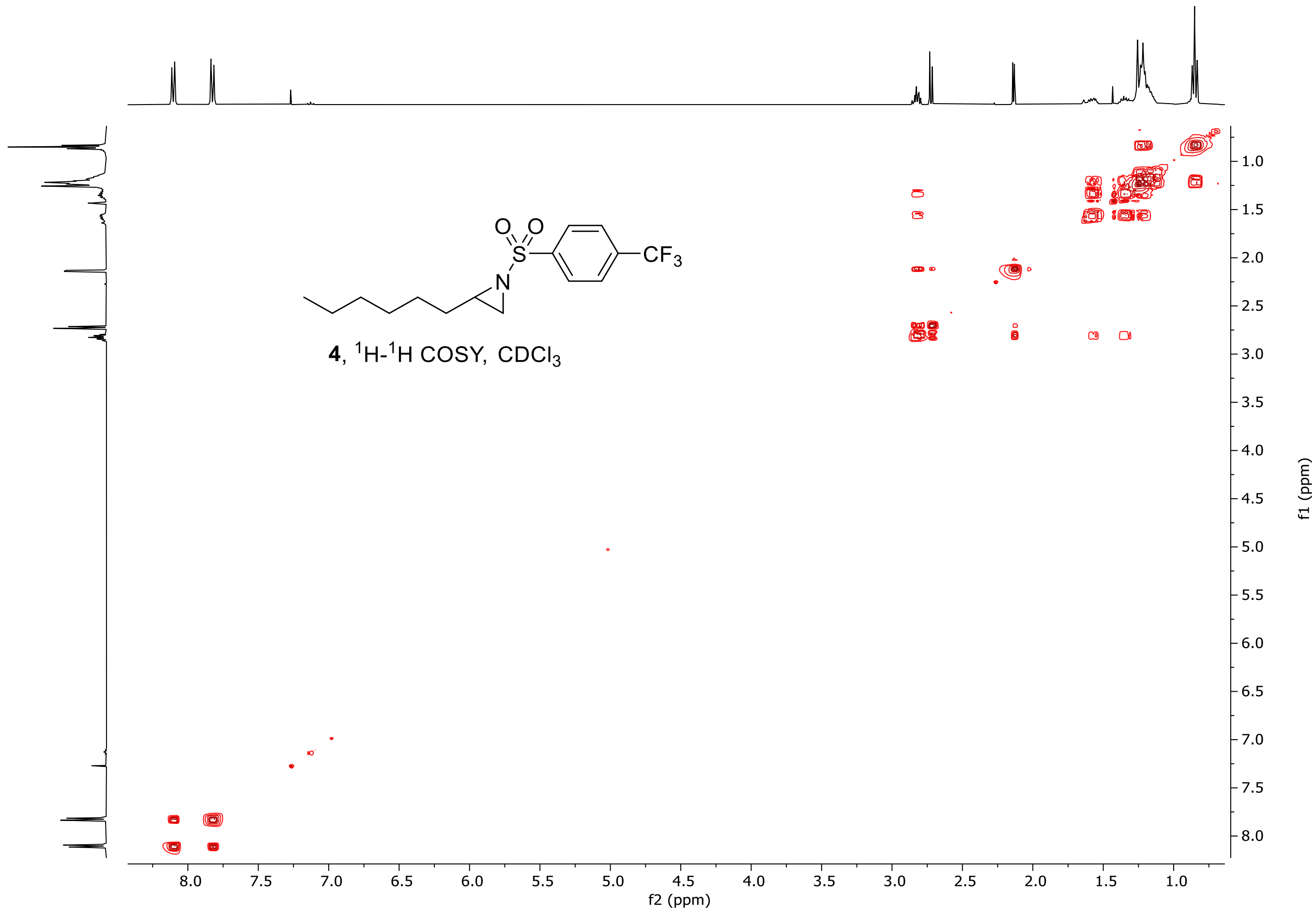

4, ^{1}H-^{1}H COSY, $CDCl_3$

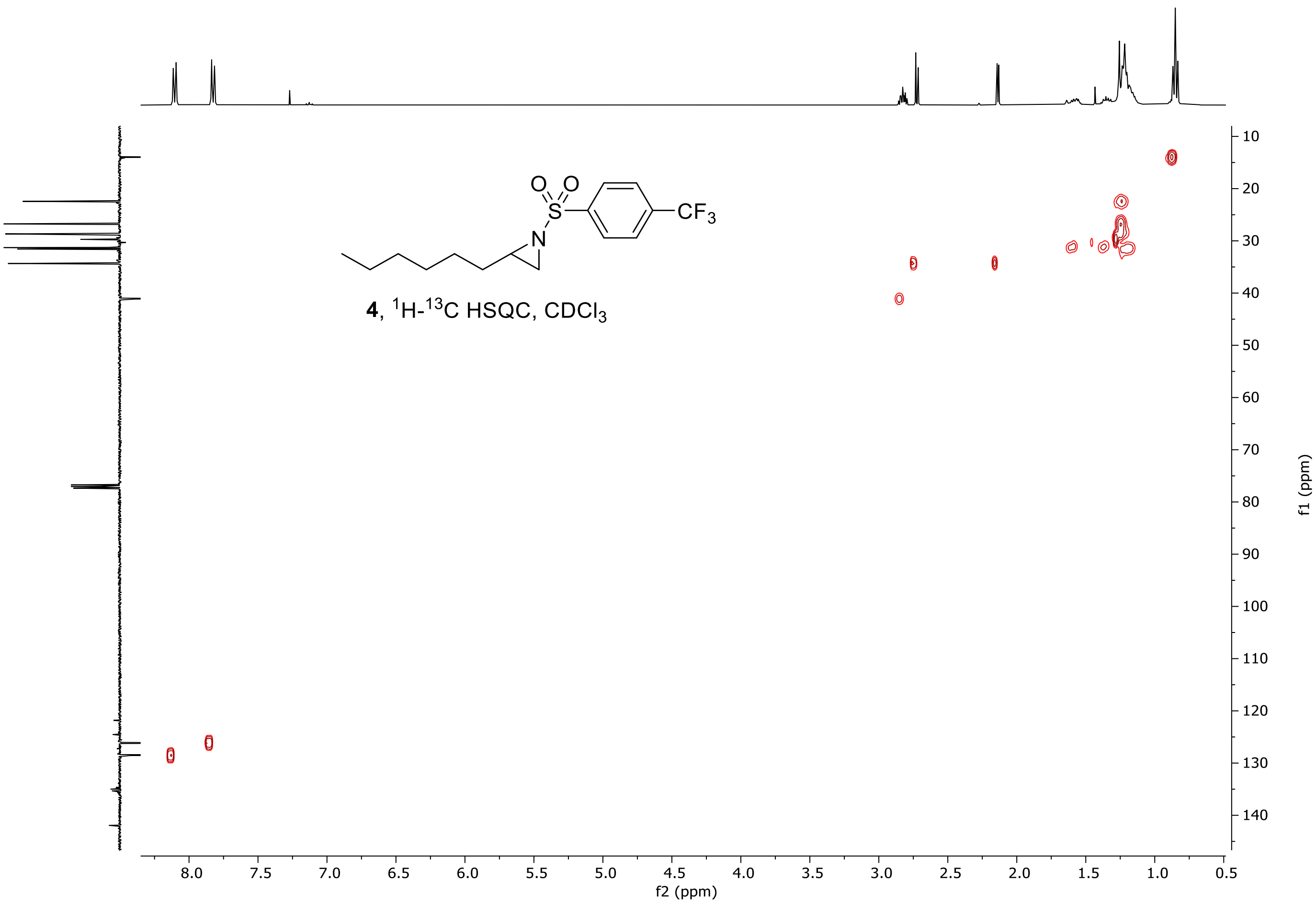

4, ^{1}H-^{13}C HSQC, $CDCl_3$

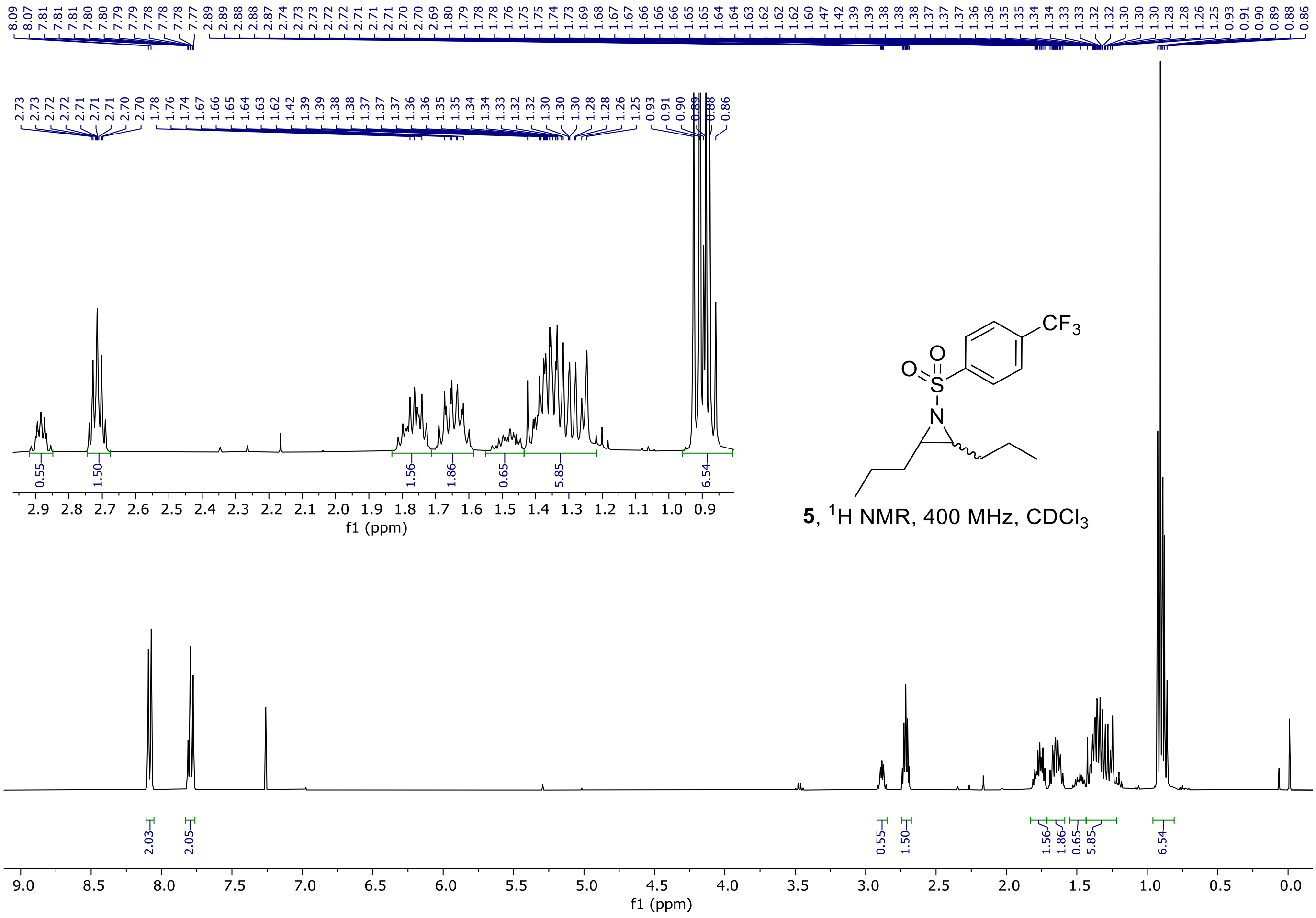

5, ^{1}H NMR, 400 MHz, $CDCl_3$

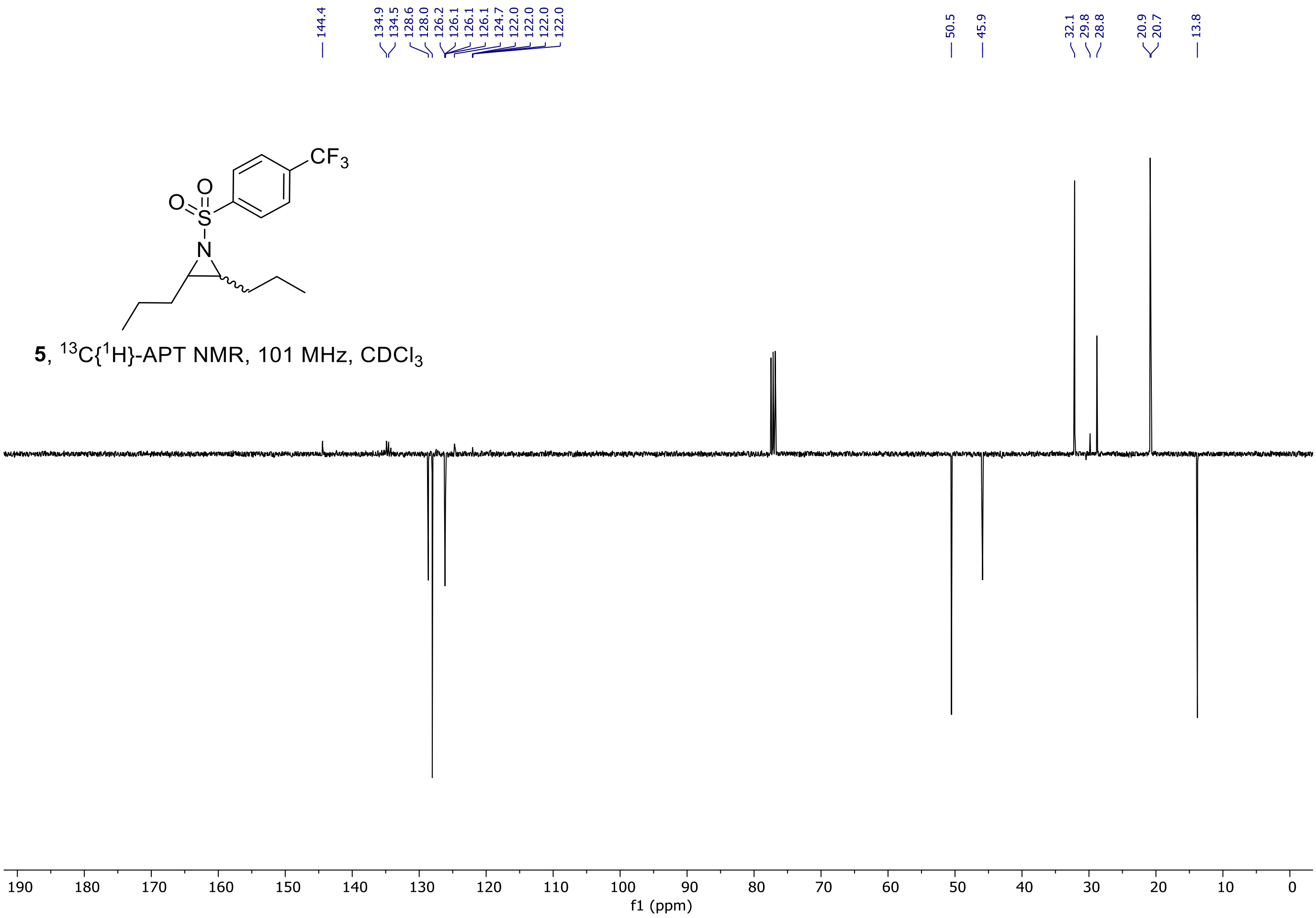
5, $^{13}C\{^1H\}$-APT NMR, 101 MHz, $CDCl_3$
f1 (ppm)

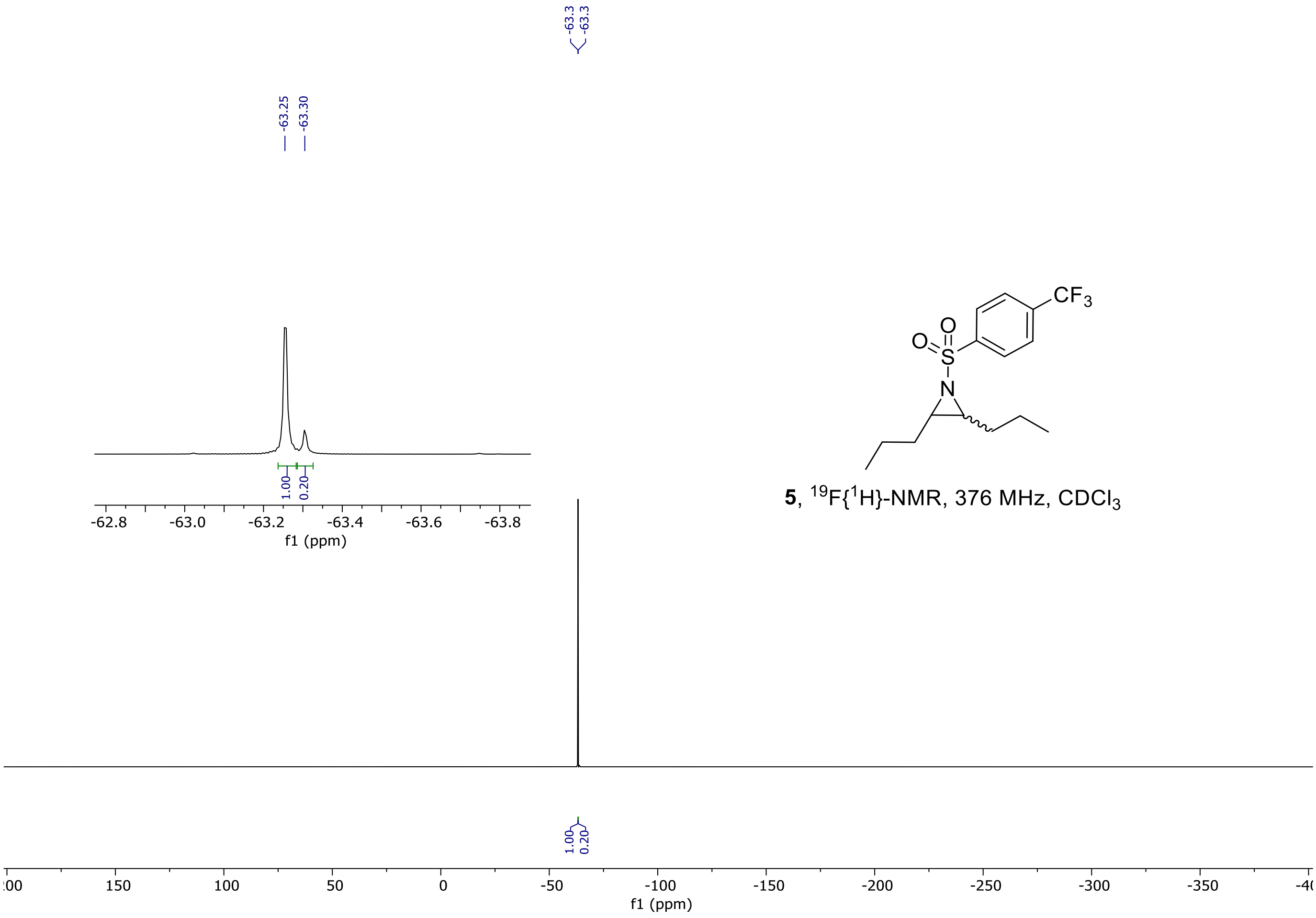
5, $^{19}F\{^1H\}$-NMR, 376 MHz, $CDCl_3$
f1 (ppm)

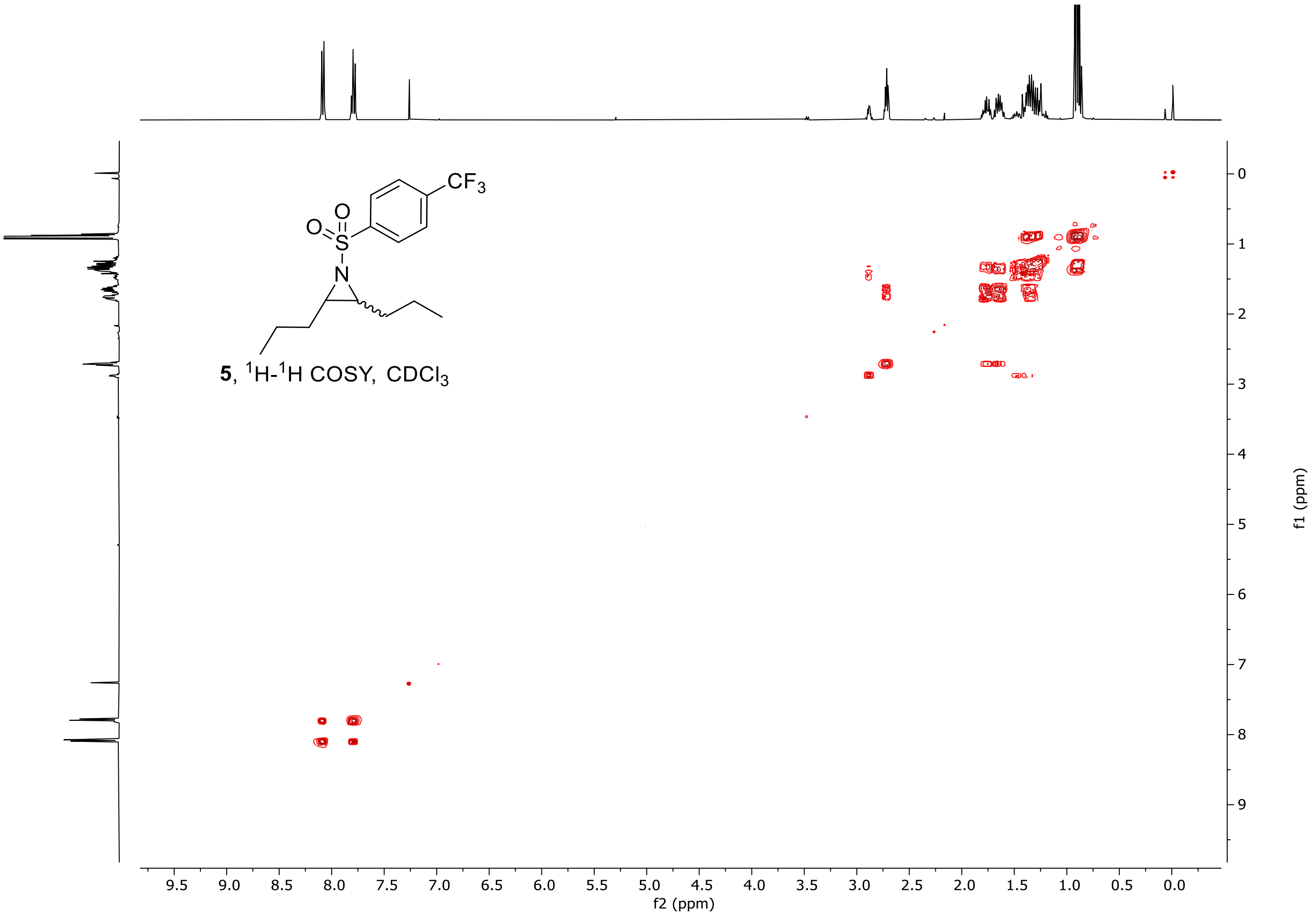
CF3
O
O
S
N
5, 1H-1H COSY, CDCl3
f1 (ppm)
f2 (ppm)

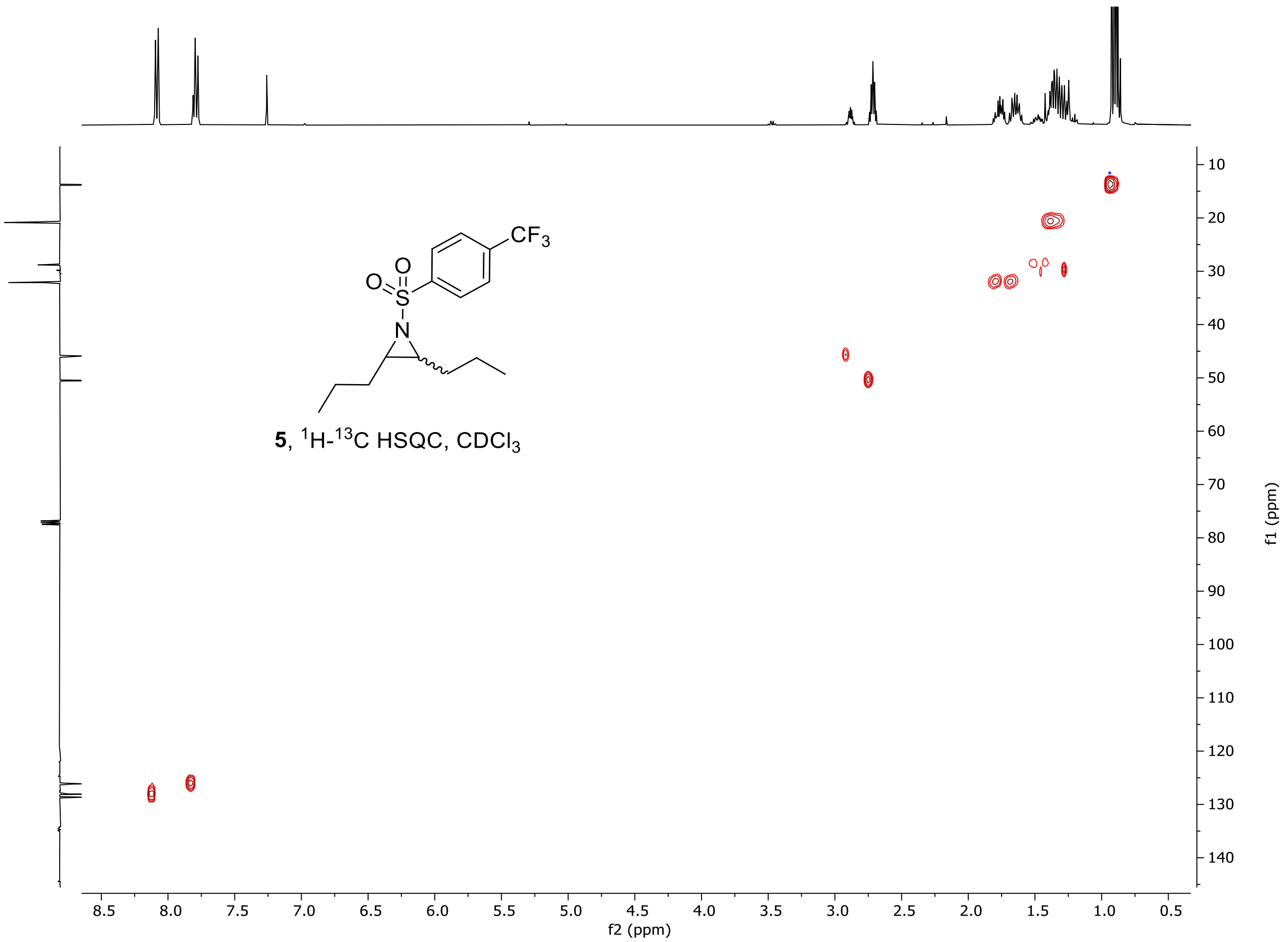
CF3
O
O
S
N
5, 1H-13C HSQC, CDCl3
f1 (ppm)
f2 (ppm)

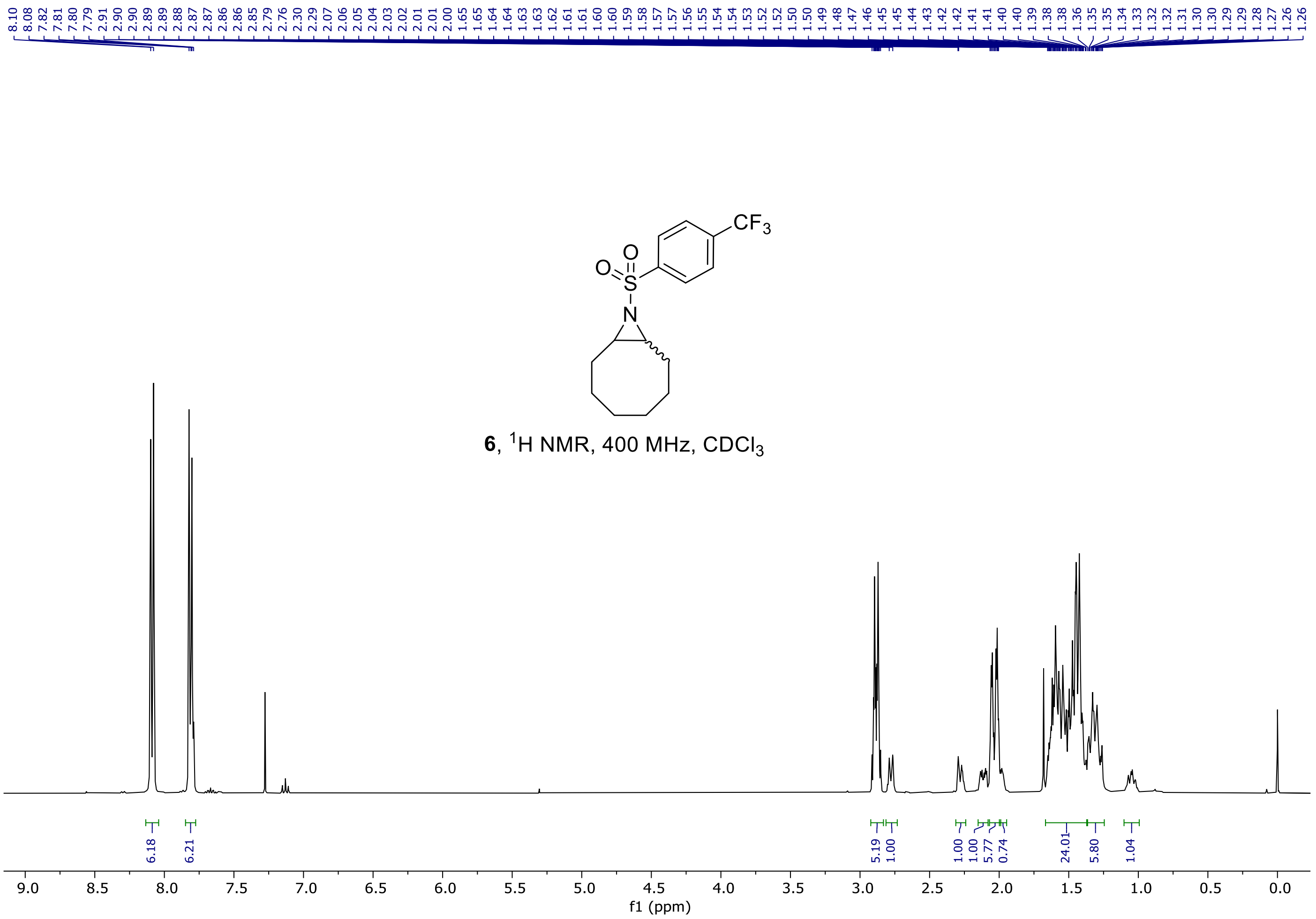
8.10 8.08 7.82 7.81 7.80 7.79 2.91 2.90 2.90 2.89 2.89 2.88 2.87 2.87 2.86 2.86 2.85 2.79 2.76 2.30 2.29 2.07 2.06 2.05 2.04 2.03 2.02 2.01 2.01 2.00 1.65 1.65 1.64 1.64 1.63 1.63 1.62 1.61 1.61 1.60 1.59 1.58 1.57 1.57 1.56 1.55 1.54 1.54 1.53 1.52 1.52 1.50 1.49 1.48 1.47 1.46 1.45 1.45 1.44 1.43 1.42 1.42 1.41 1.40 1.40 1.39 1.38 1.38 1.36 1.35 1.35 1.34 1.33 1.32 1.32 1.31 1.30 1.30 1.29 1.29 1.28 1.27 1.26 1.26
CF3
O
O
S
N
6, 1H NMR, 400 MHz, CDCl3
6.18 6.21 5.19 1.00 1.00 1.00 5.77 0.74 24.01 5.80 1.04
9.0 8.5 8.0 7.5 7.0 6.5 6.0 5.5 5.0 4.5 4.0 3.5 3.0 2.5 2.0 1.5 1.0 0.5 0.0
f1 (ppm)

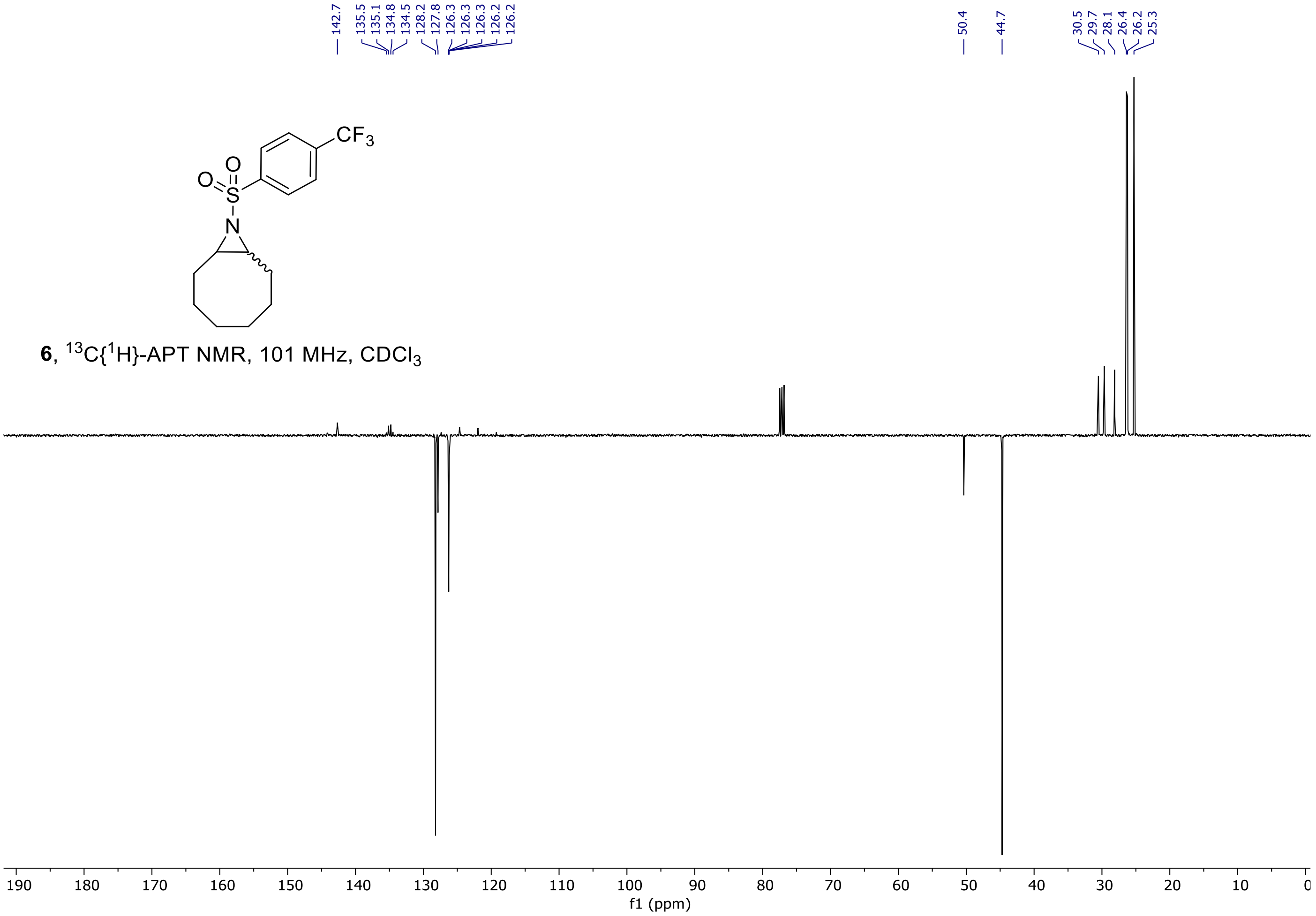
142.7 135.5 135.1 134.8 134.5 128.2 127.8 126.3 126.3 126.3 126.2 126.2
50.4 44.7
30.5 29.7 28.1 26.4 26.2 25.3
CF3
O
O
S
N
6, 13C{1H}-APT NMR, 101 MHz, CDCl3
190 180 170 160 150 140 130 120 110 100 90 80 70 60 50 40 30 20 10 0
f1 (ppm)

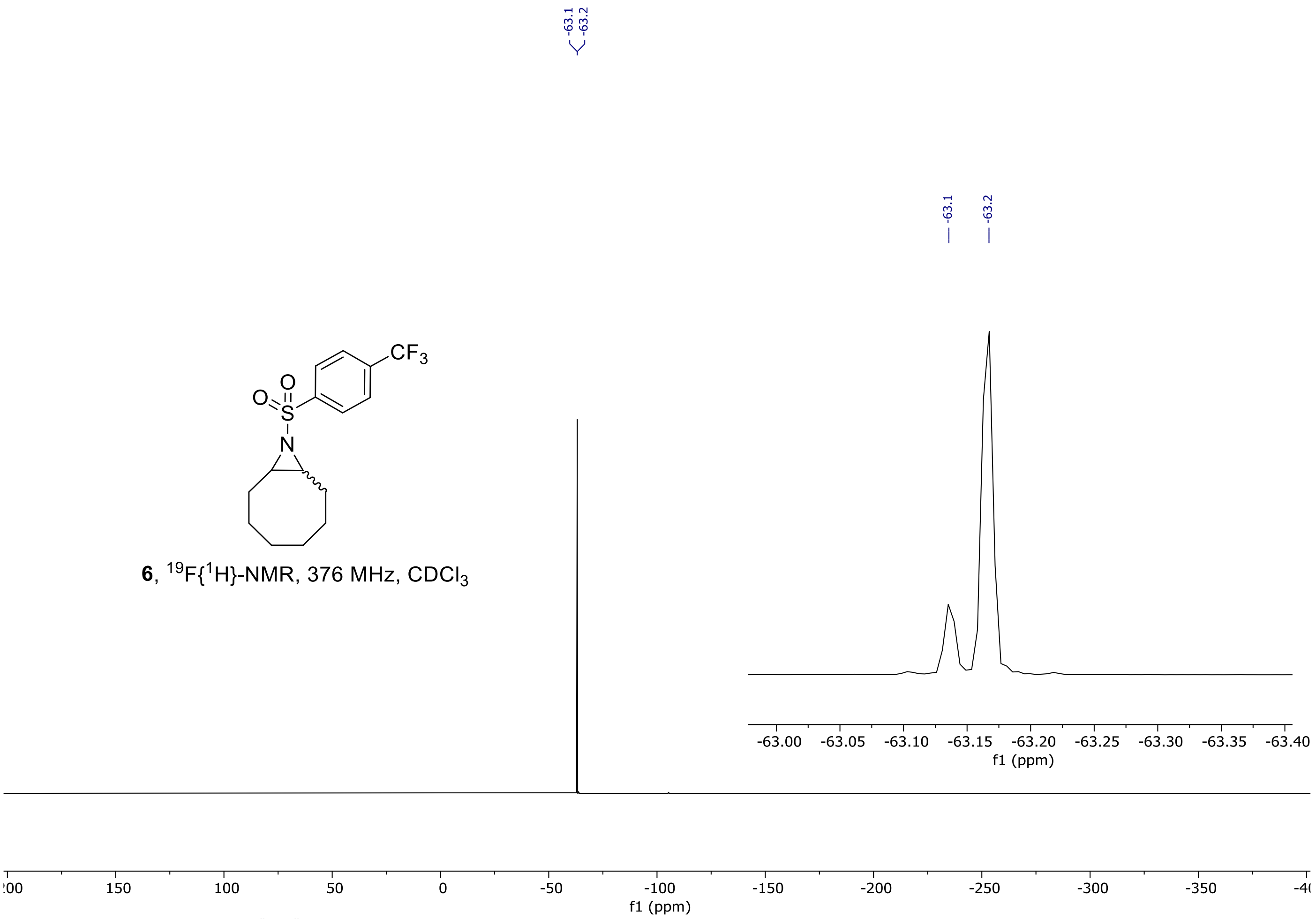
-63.1
-63.2
CF3
6, 19F{1H}-NMR, 376 MHz, CDCl3
f1 (ppm)

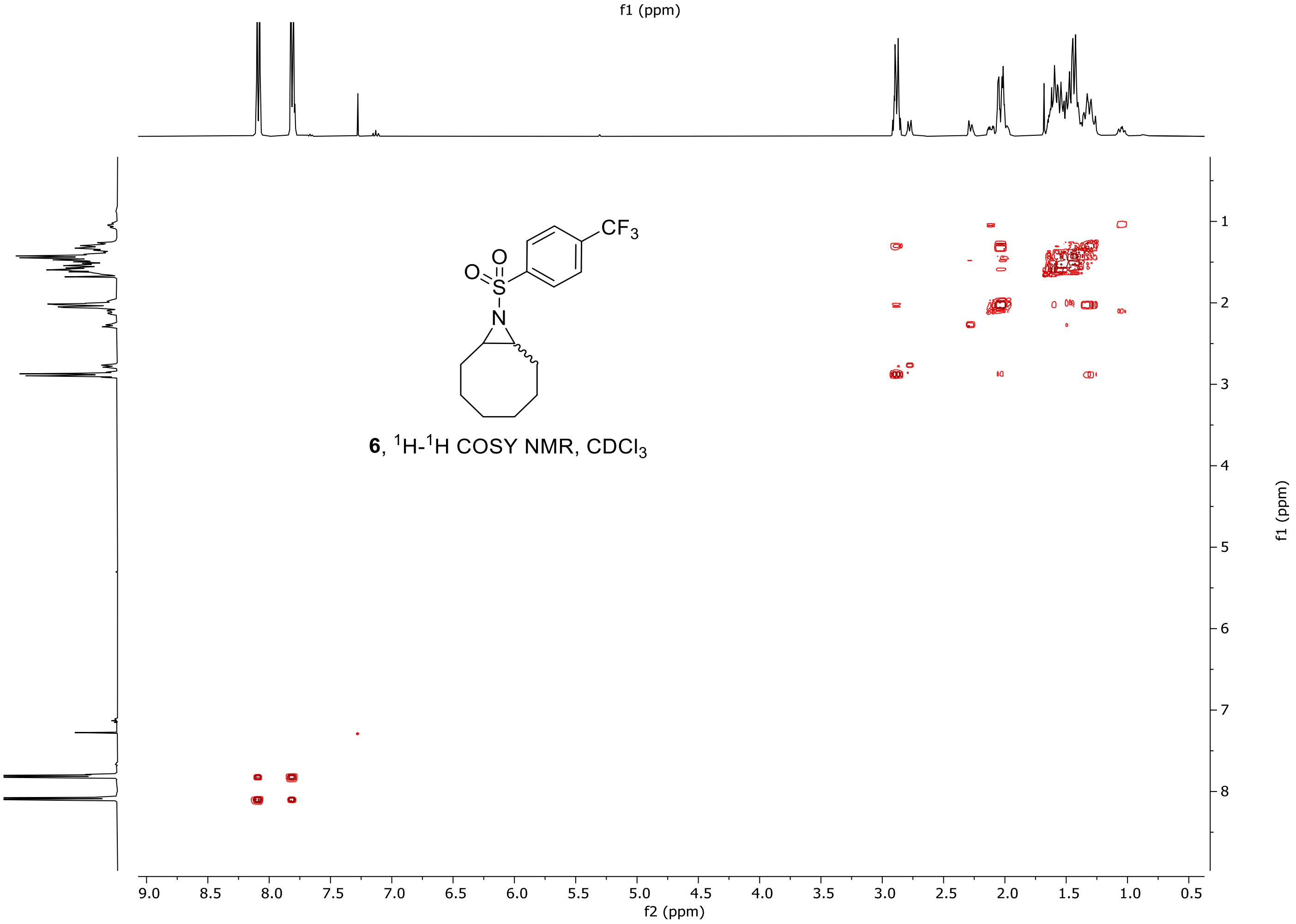
CF3
6, 1H-1H COSY NMR, CDCl3
f2 (ppm)
f1 (ppm)

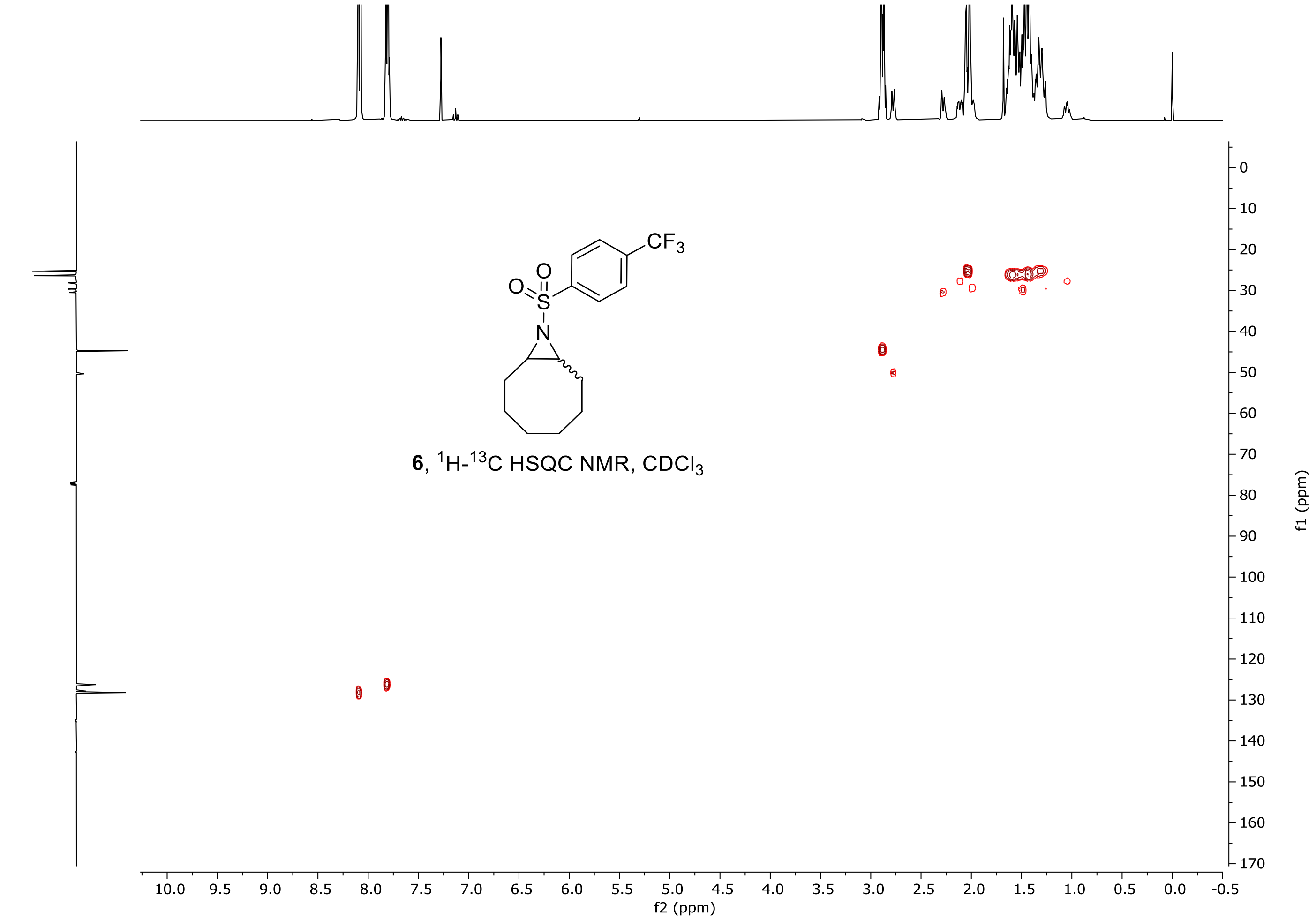
CF3
O
O
S
N
6, 1H-13C HSQC NMR, CDCl3
f2 (ppm)
f1 (ppm)

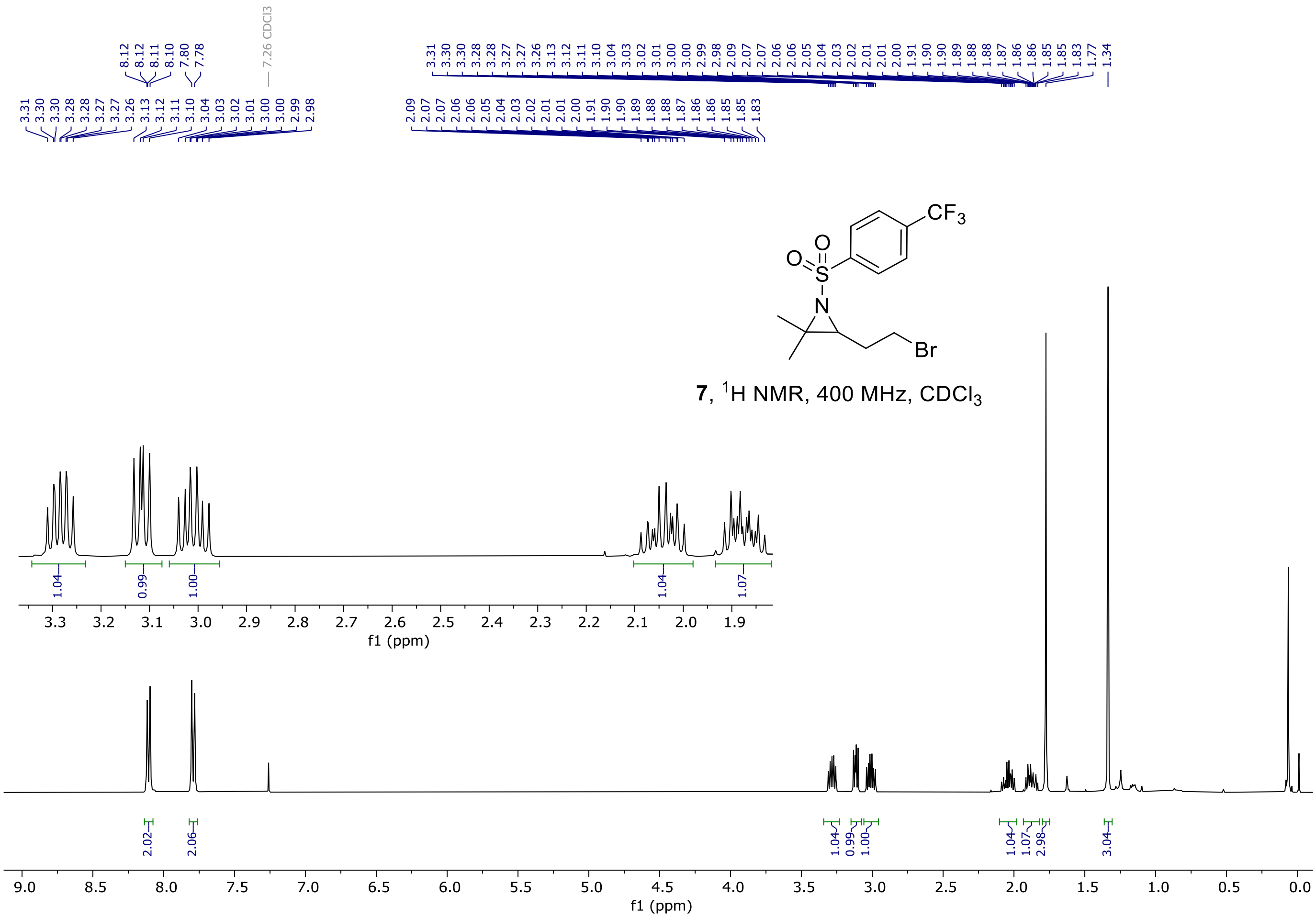
7.26 CDCl3
CF3
O
O
S
N
Br
7, 1H NMR, 400 MHz, CDCl3
f1 (ppm)

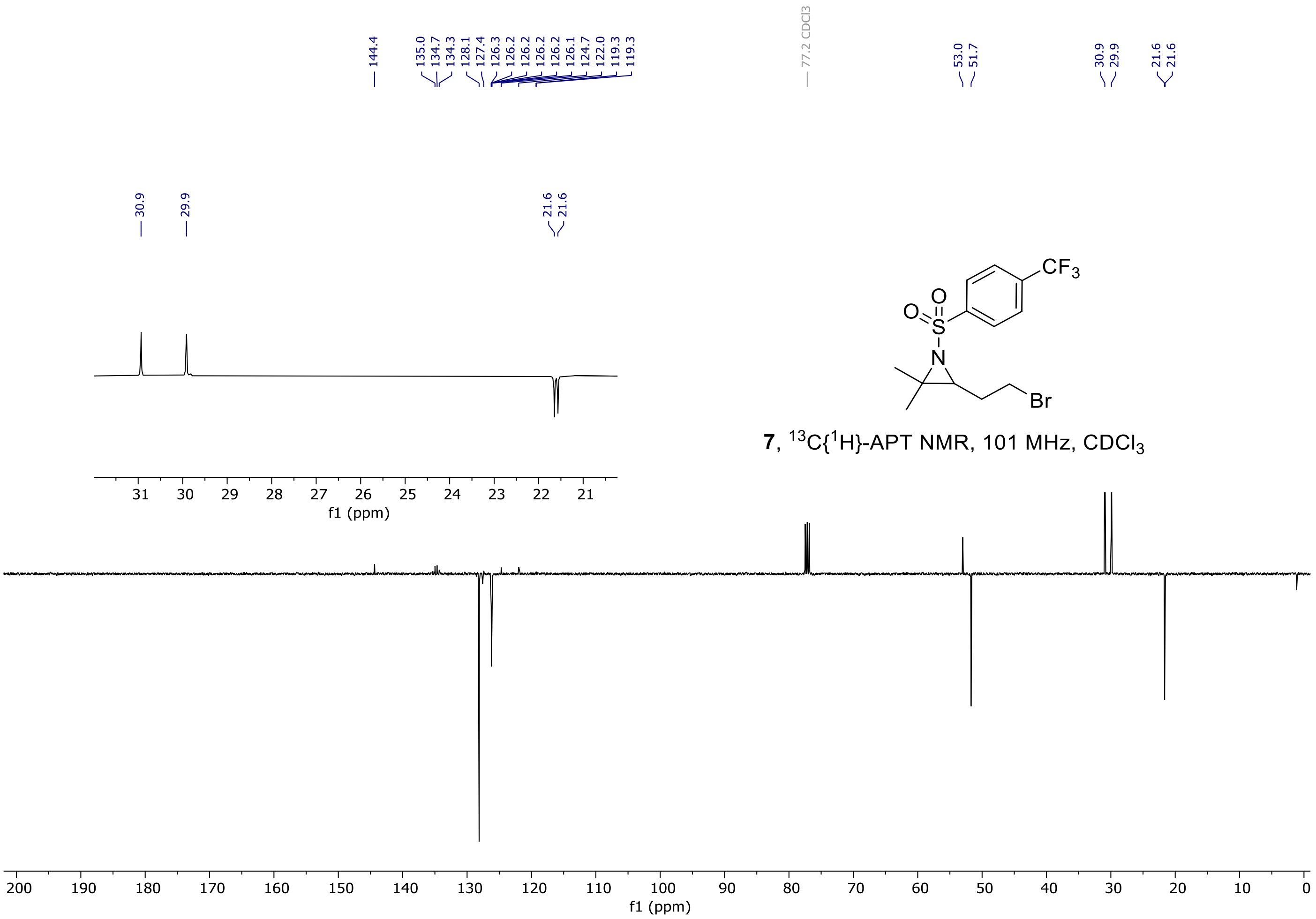

7, $^{13}C\{^{1}H\}$-APT NMR, 101 MHz, $CDCl_3$

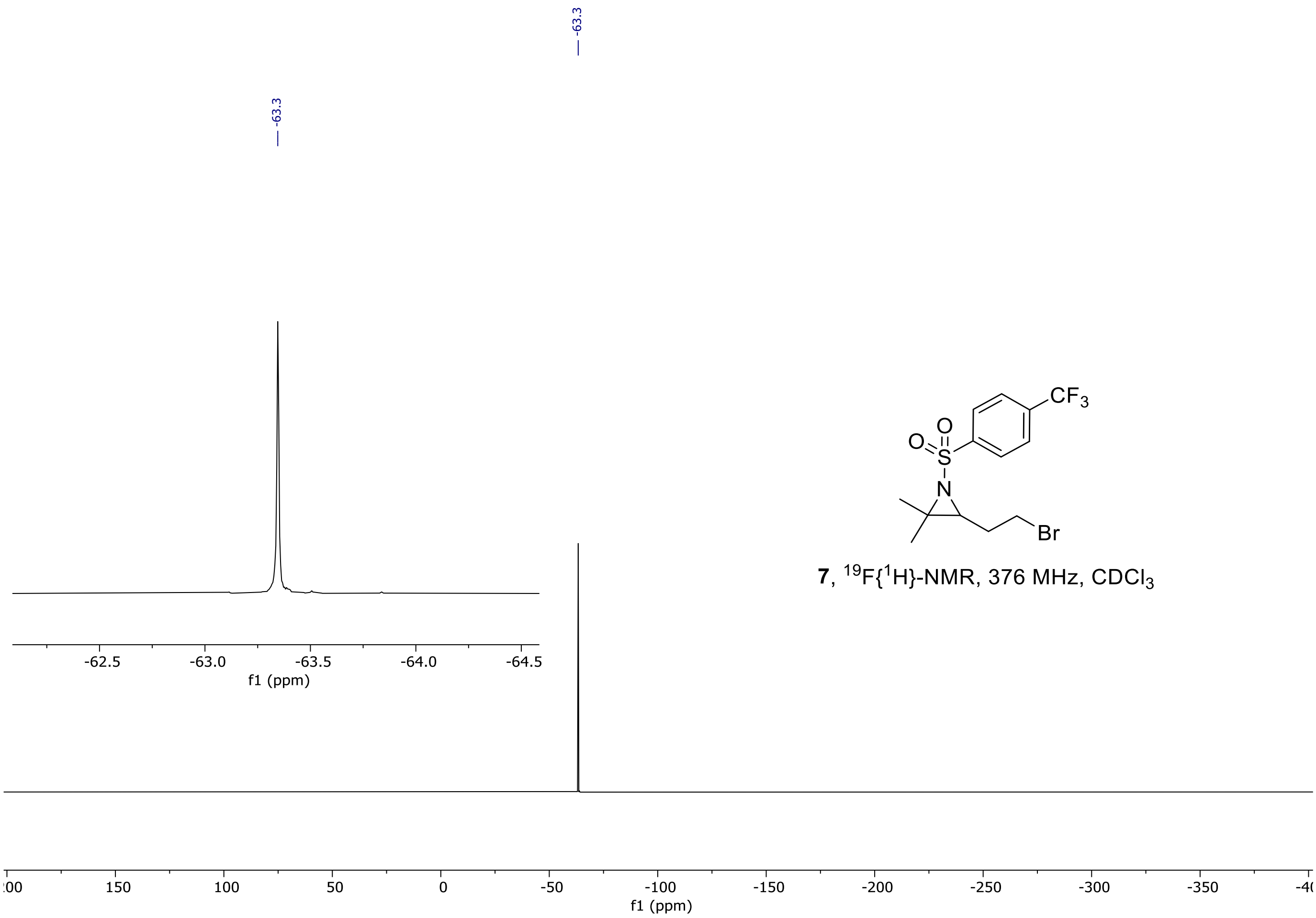

7, $^{19}F\{^{1}H\}$-NMR, 376 MHz, $CDCl_3$

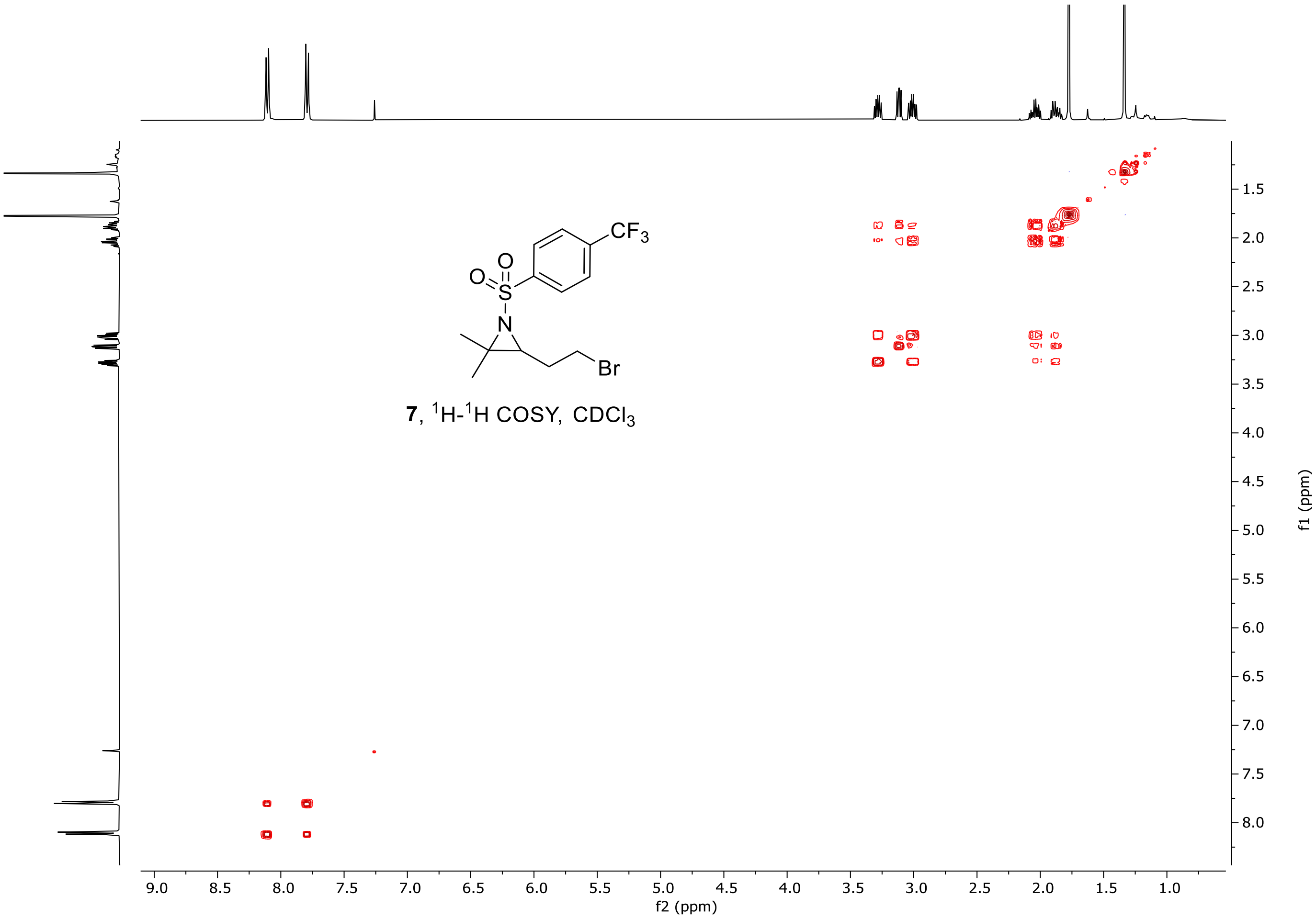
CF3
O
O
S
N
Br
7, 1H-1H COSY, CDCl3
f2 (ppm)
f1 (ppm)

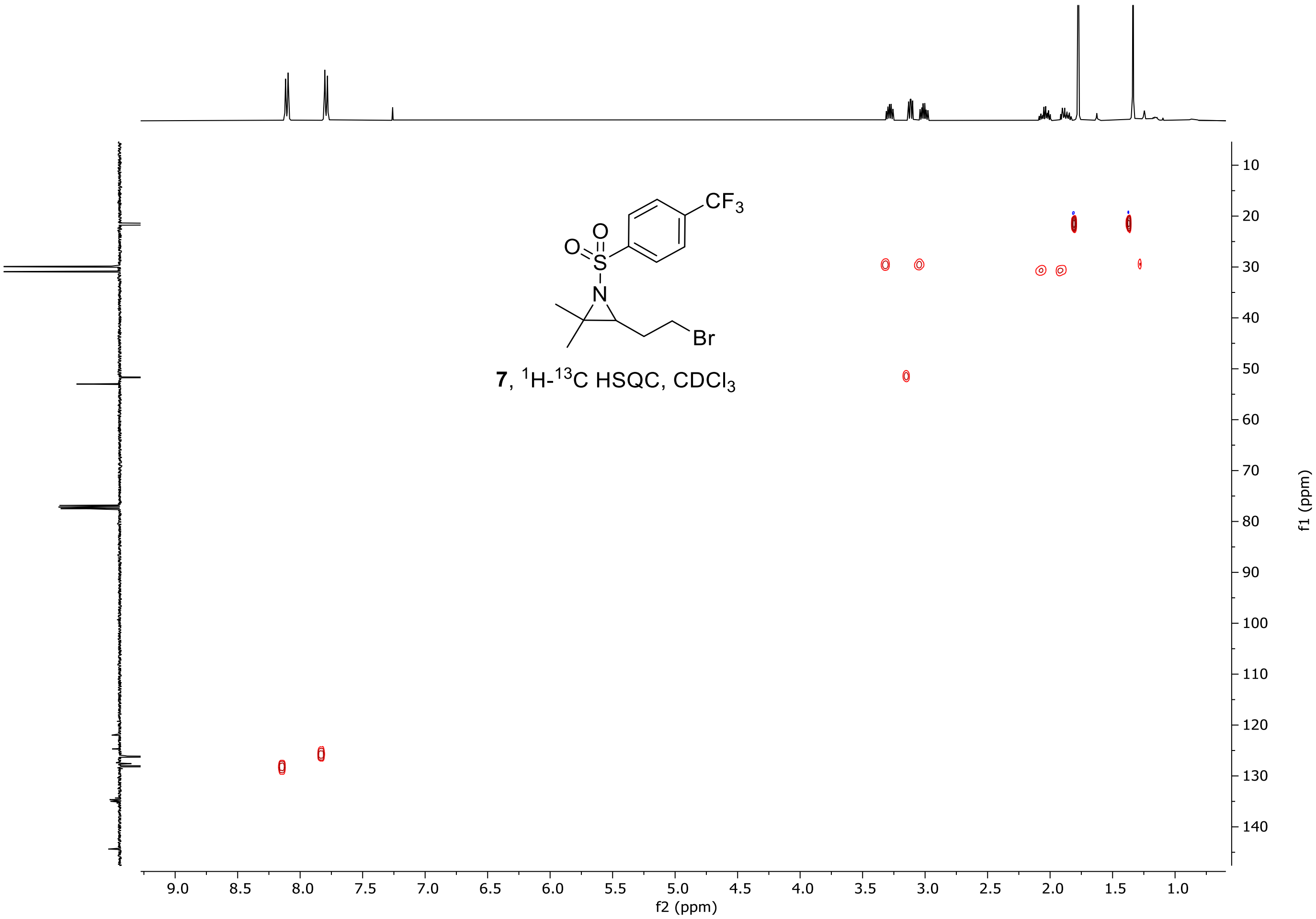
CF3
O
O
S
N
Br
7, 1H-13C HSQC, CDCl3
f2 (ppm)
f1 (ppm)

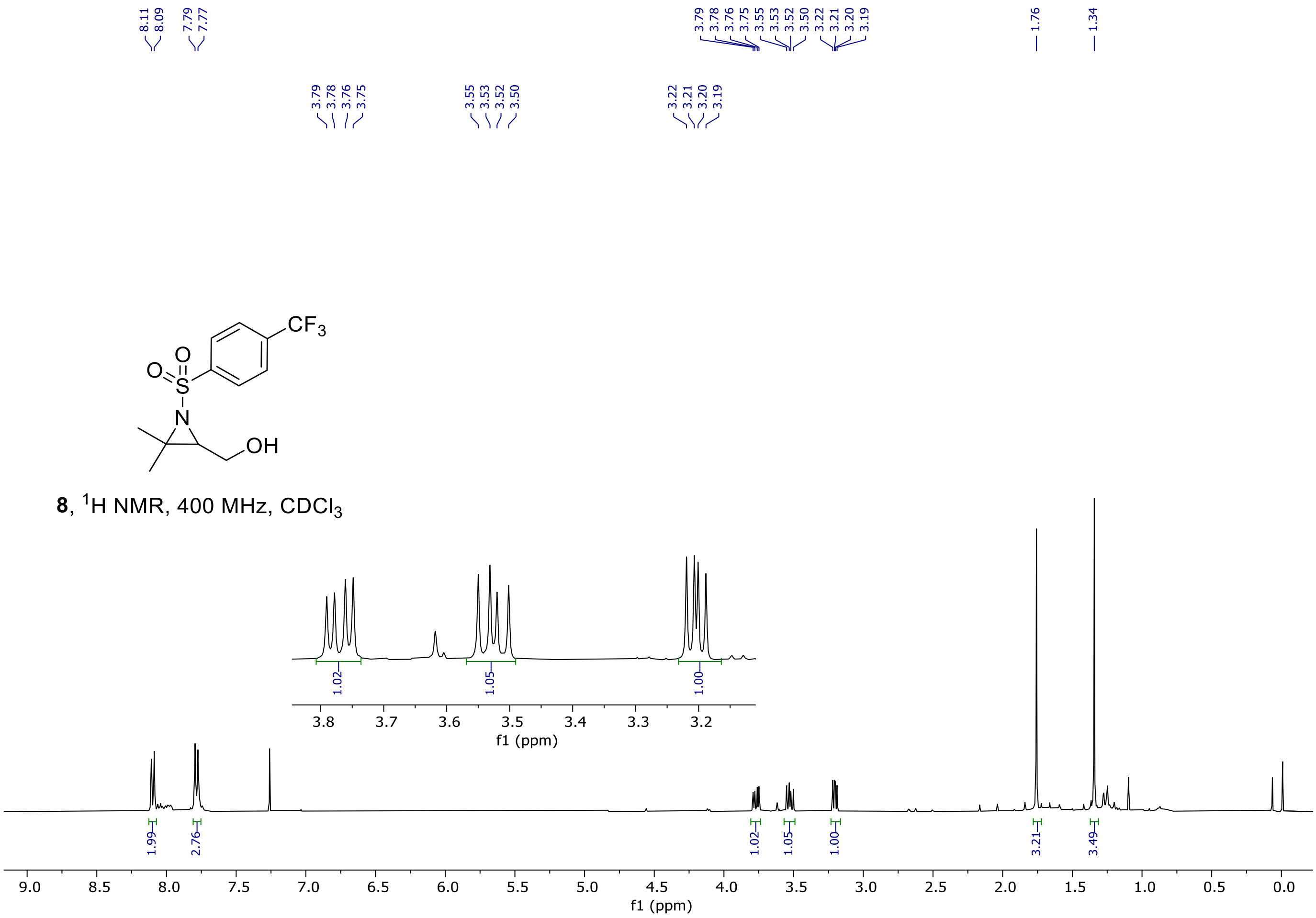

8, ^{1}H NMR, 400 MHz, $CDCl_3$

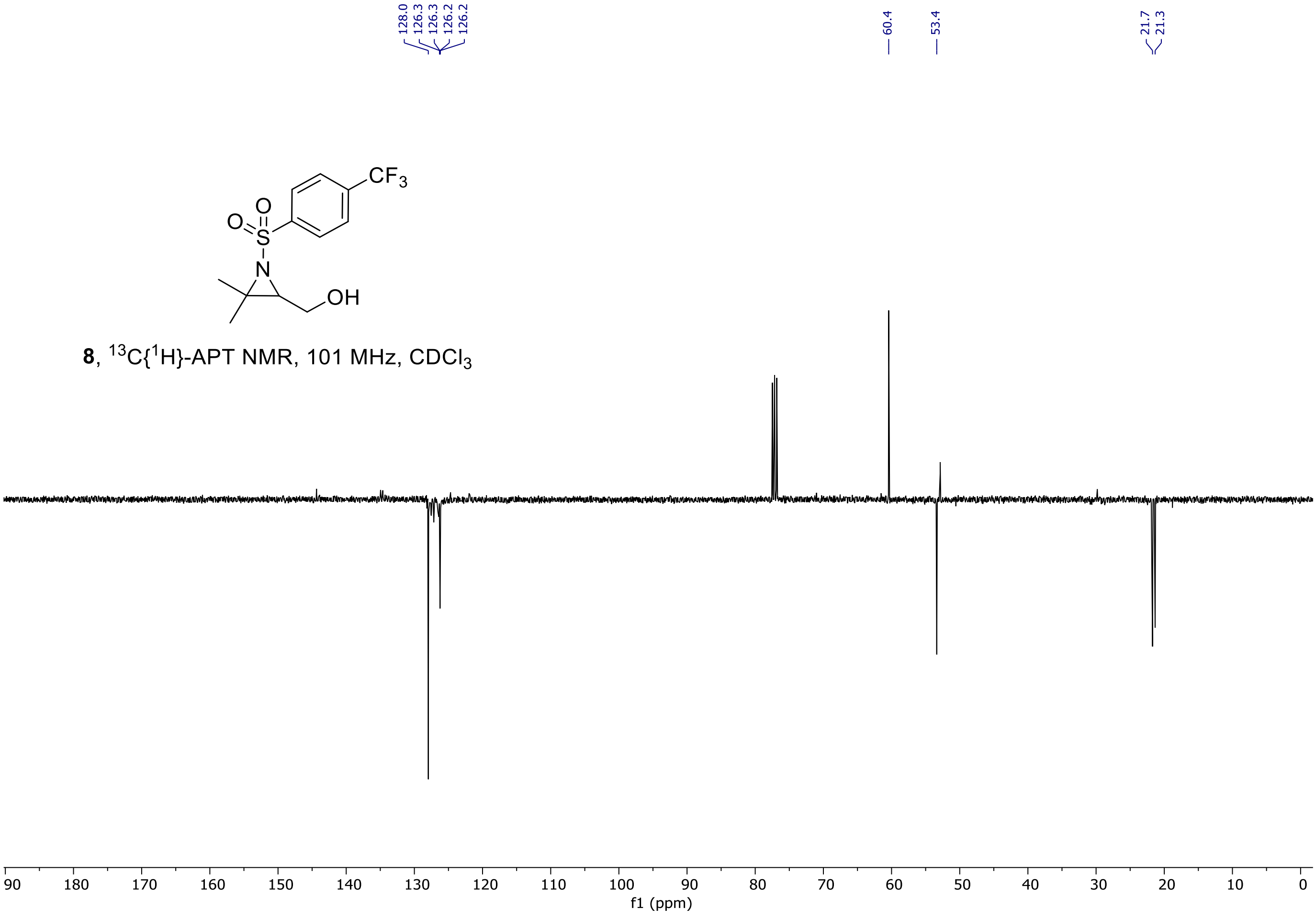

8, $^{13}C\{^{1}H\}$-APT NMR, 101 MHz, $CDCl_3$

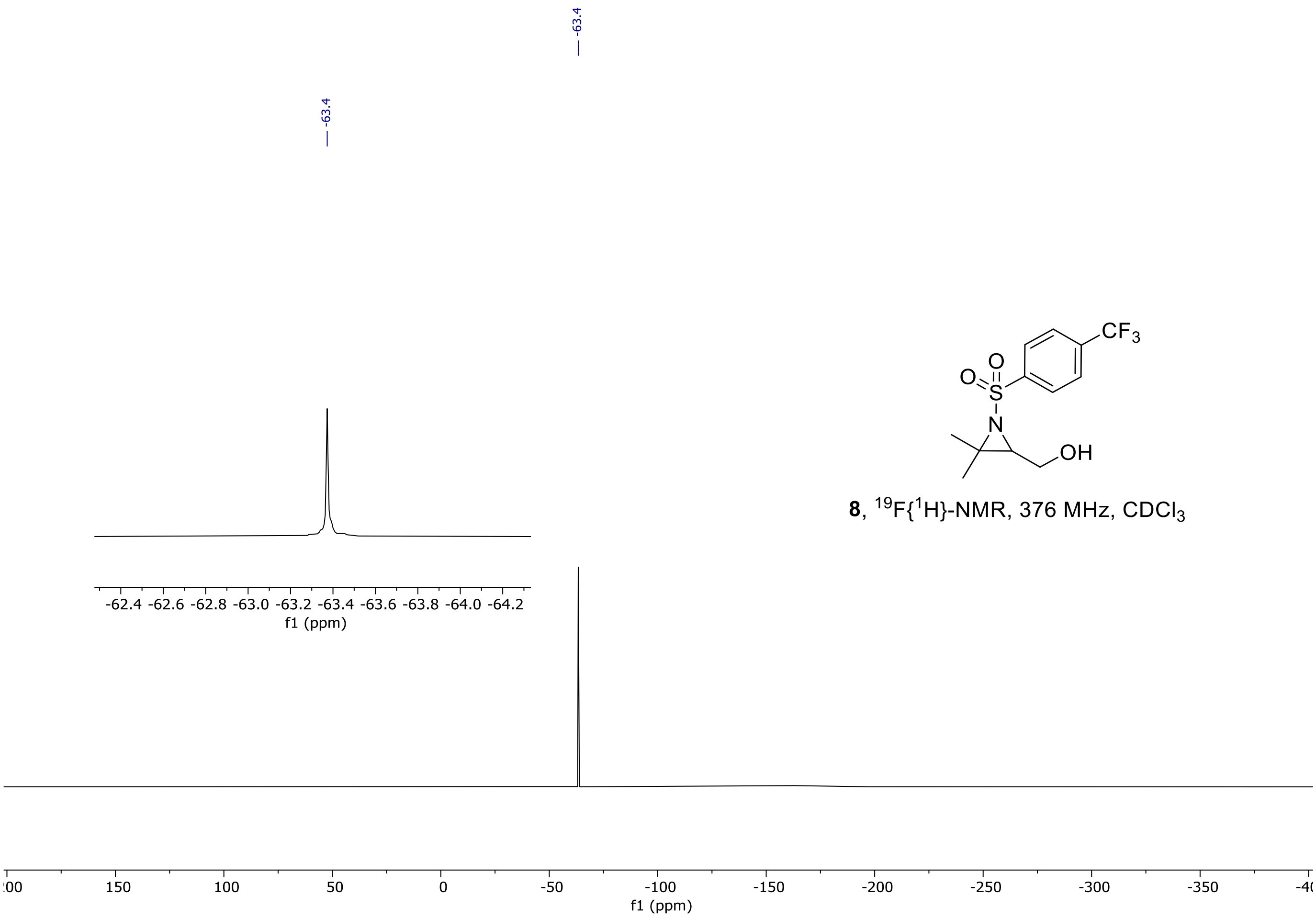
-63.4
-63.4
CF3
O
O
S
N
OH
8, 19F{1H}-NMR, 376 MHz, CDCl3
-62.4 -62.6 -62.8 -63.0 -63.2 -63.4 -63.6 -63.8 -64.0 -64.2
f1 (ppm)
150
100
50
0
-50
-100
-150
-200
-250
-300
-350
f1 (ppm)

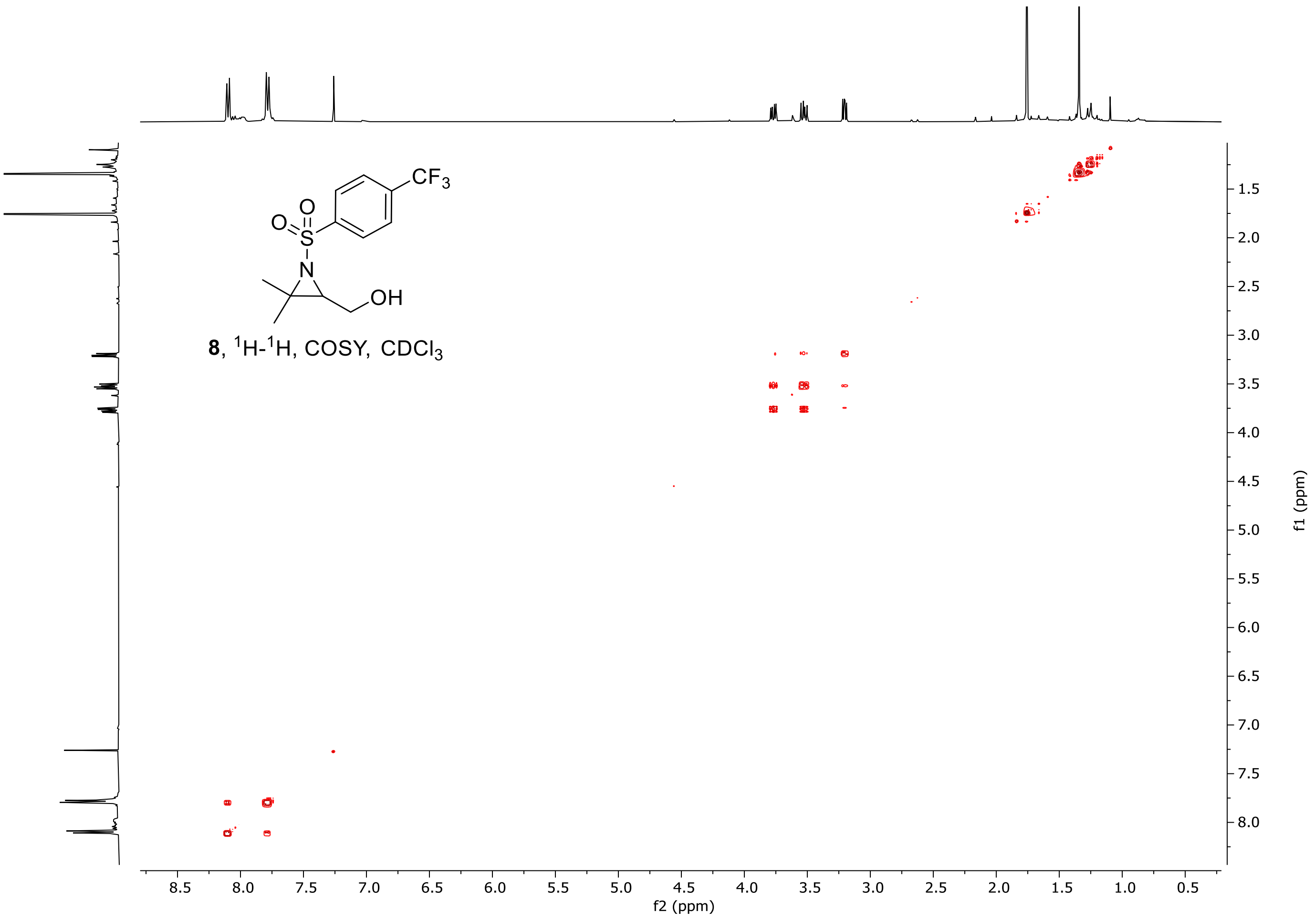
CF3
O
O
S
N
OH
8, 1H-1H, COSY, CDCl3
1.5
2.0
2.5
3.0
3.5
4.0
4.5
5.0
5.5
6.0
6.5
7.0
7.5
8.0
f1 (ppm)
8.5
8.0
7.5
7.0
6.5
6.0
5.5
5.0
4.5
4.0
3.5
3.0
2.5
2.0
1.5
1.0
0.5
f2 (ppm)

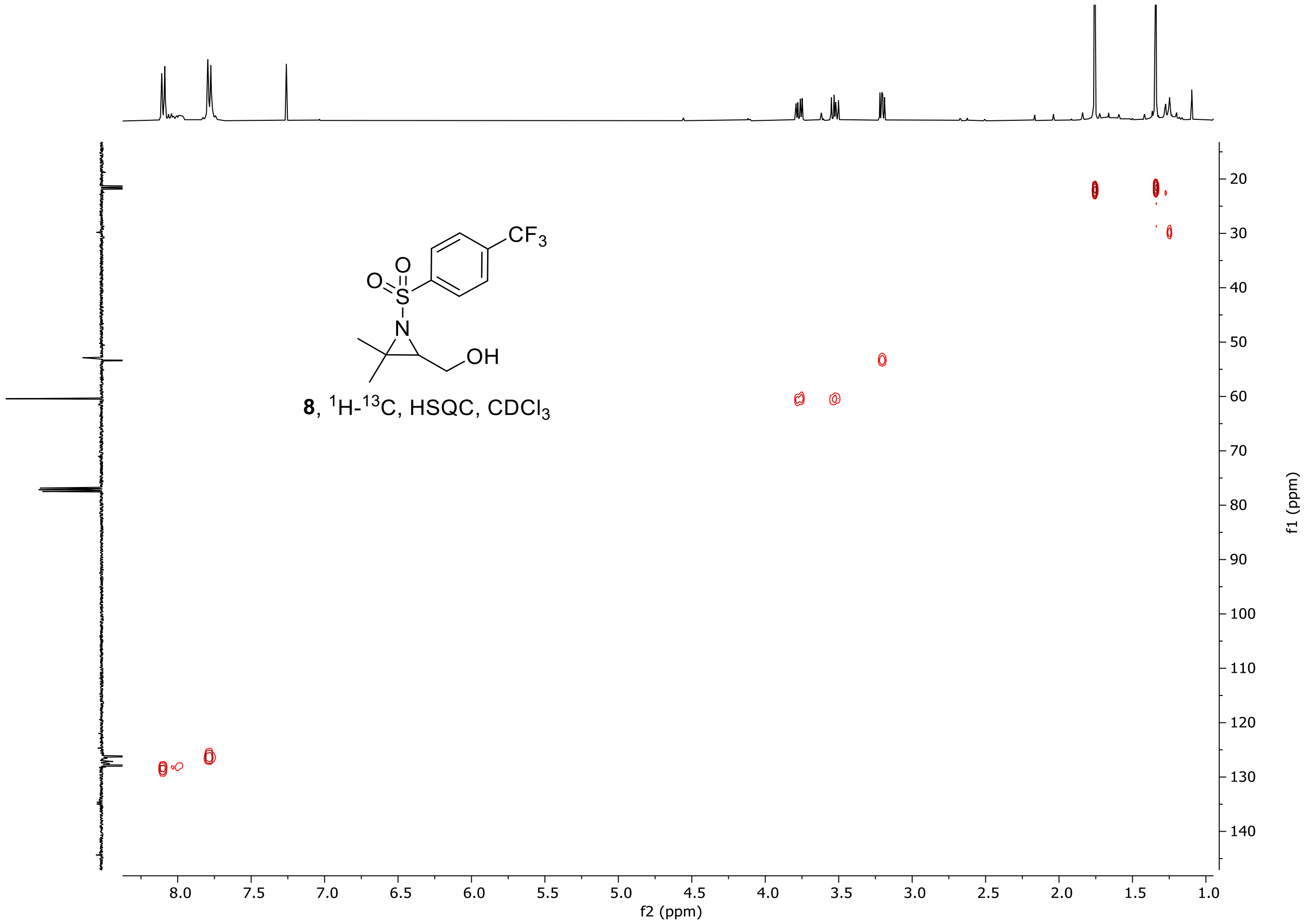
CF3
O
O
S
N
OH
8, 1H-13C, HSQC, CDCl3
f2 (ppm)
f1 (ppm)

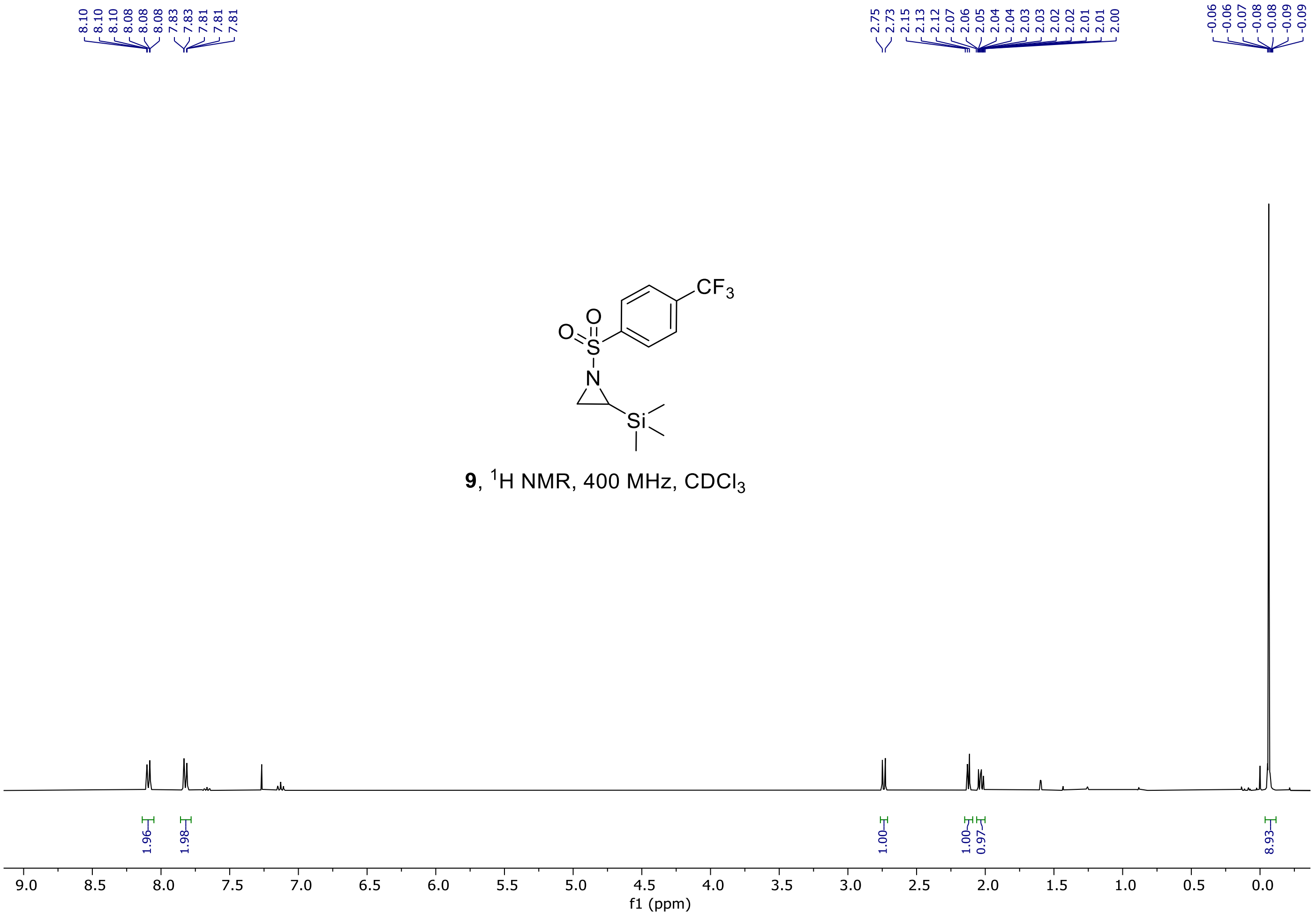
CF3
O
O
S
N
Si
9, 1H NMR, 400 MHz, CDCl3
1.96
1.98
1.00
1.00
0.97
8.93
f1 (ppm)

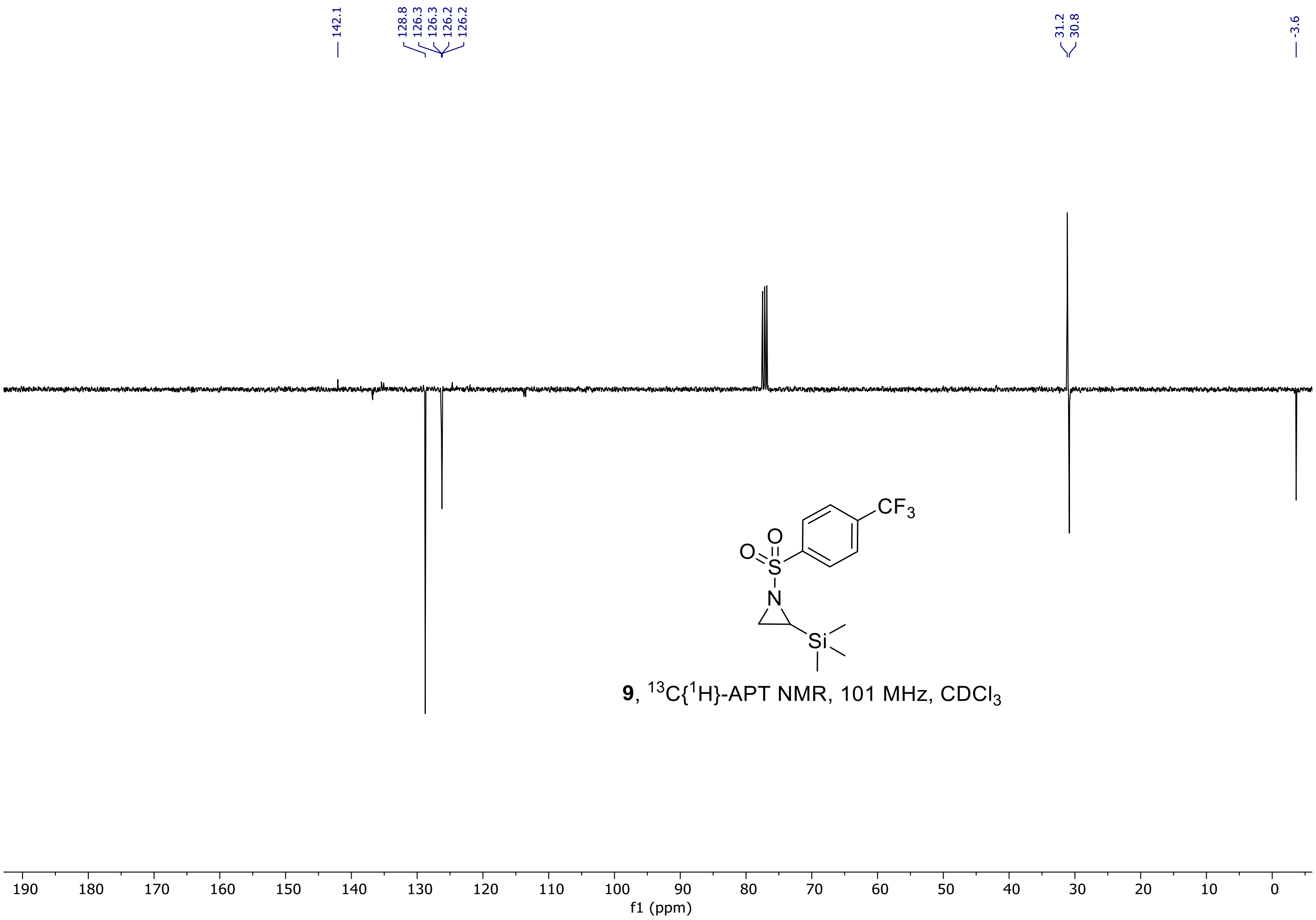
142.1
128.8
126.3
126.3
126.2
126.2
31.2
30.8
-3.6
CF3
O
O
S
N
Si
9, 13C{1H}-APT NMR, 101 MHz, CDCl3
190 180 170 160 150 140 130 120 110 100 90 80 70 60 50 40 30 20 10 0
f1 (ppm)

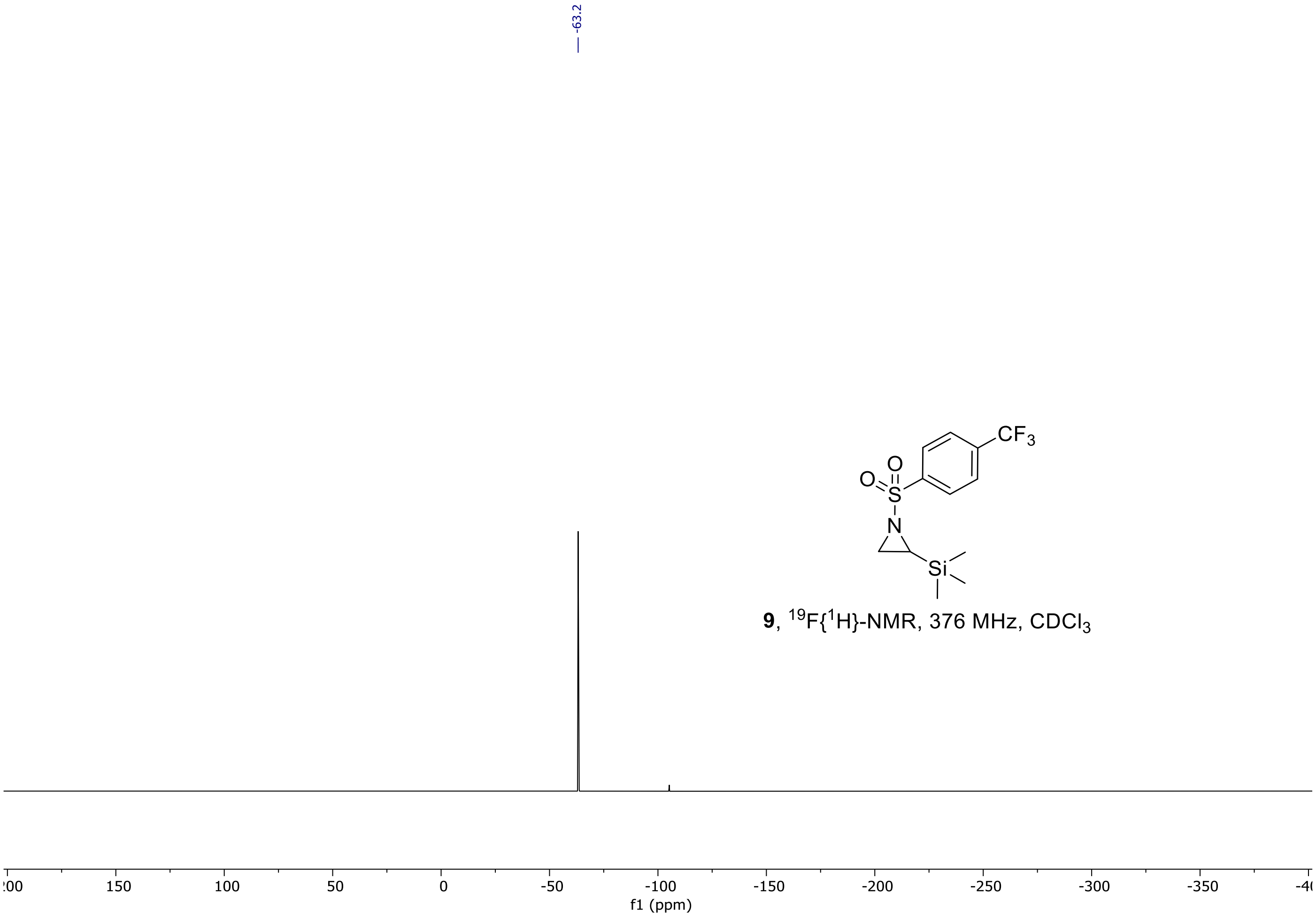
-63.2
CF3
O
O
S
N
Si
9, 19F{1H}-NMR, 376 MHz, CDCl3
150 100 50 0 -50 -100 -150 -200 -250 -300 -350
f1 (ppm)

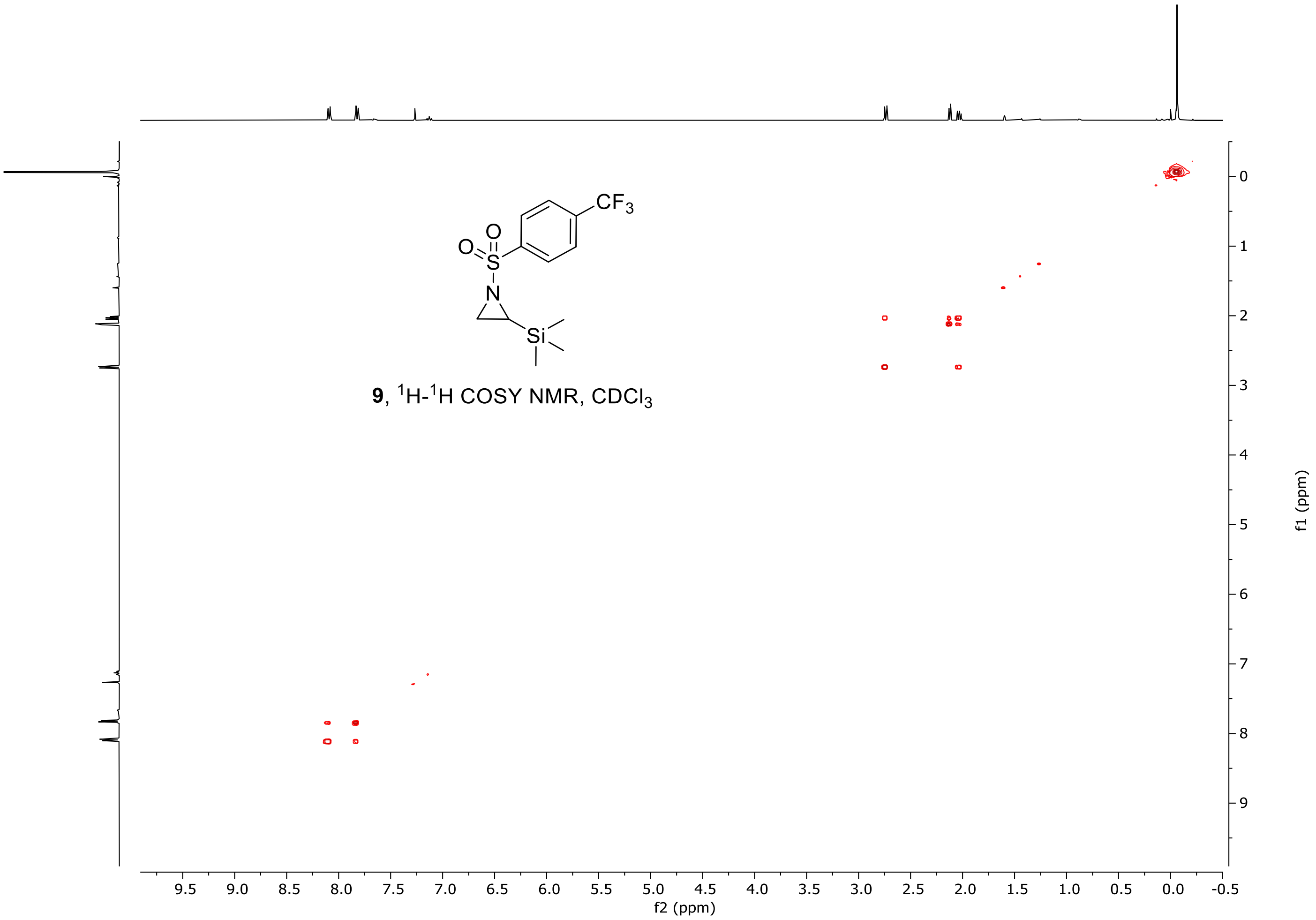
CF3
O
O
S
N
Si
9, 1H-1H COSY NMR, CDCl3
f2 (ppm)
f1 (ppm)

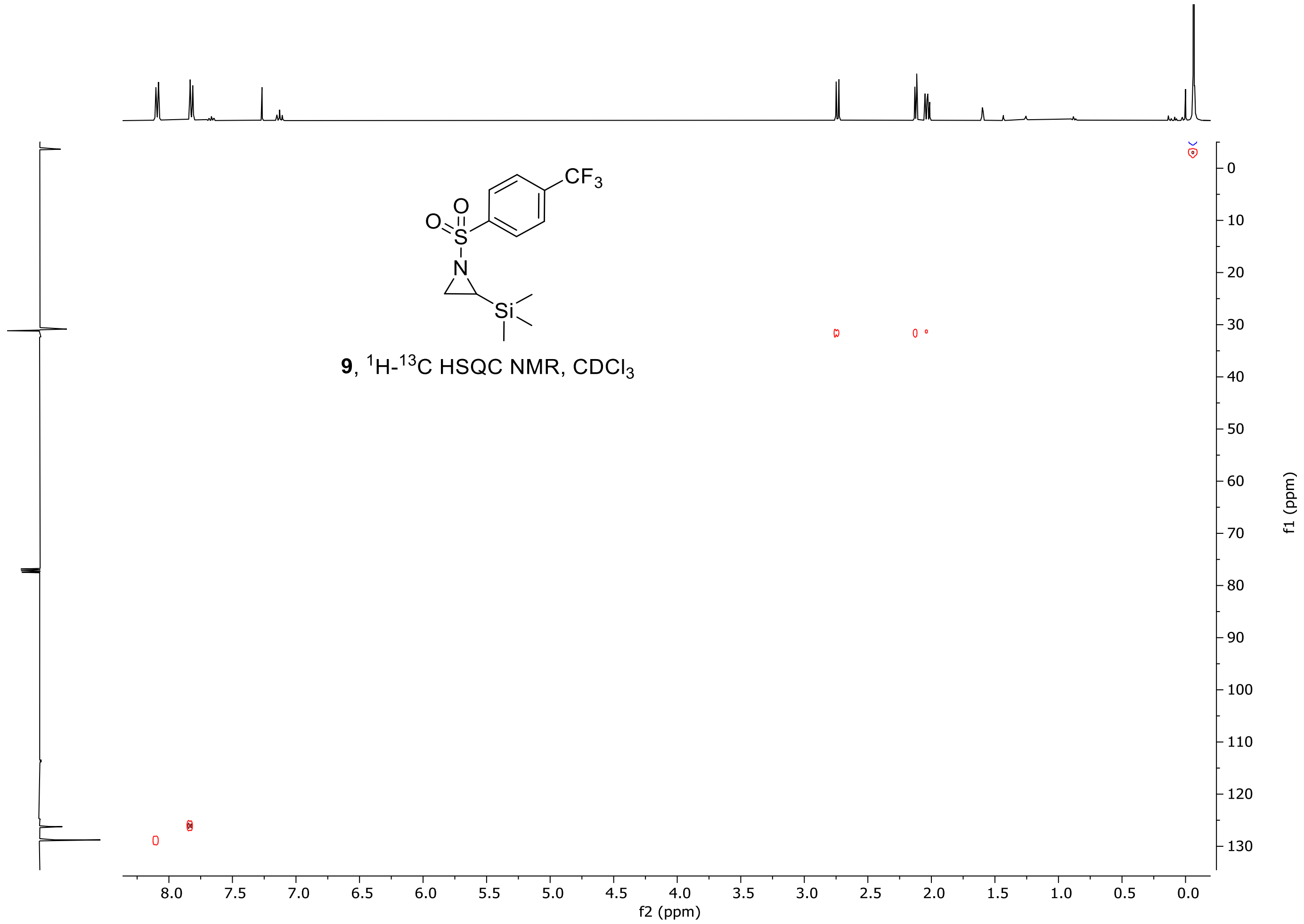
CF3
O
O
S
N
Si
9, 1H-13C HSQC NMR, CDCl3
f2 (ppm)
f1 (ppm)

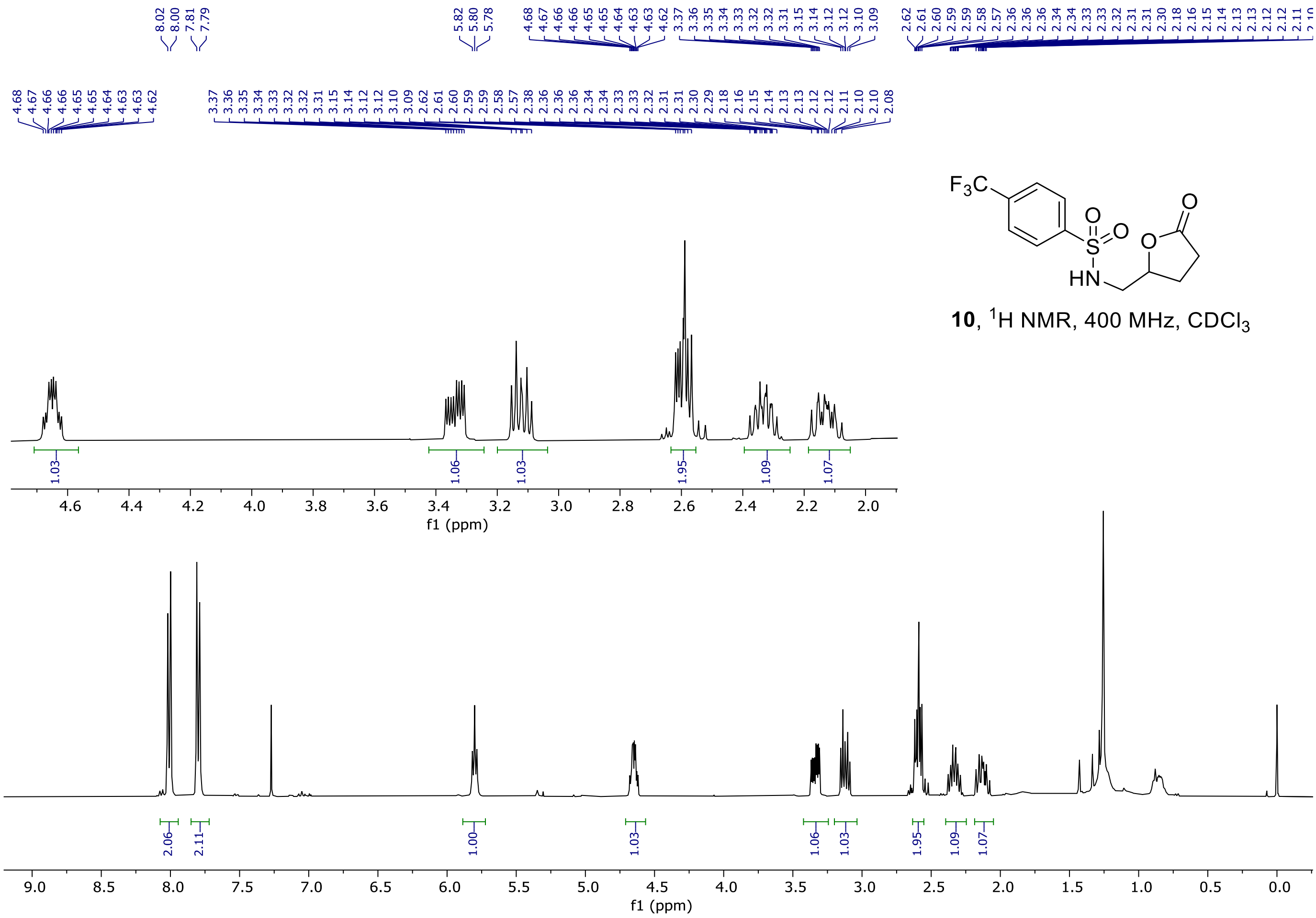

10, ^{1}H NMR, 400 MHz, $CDCl_3$

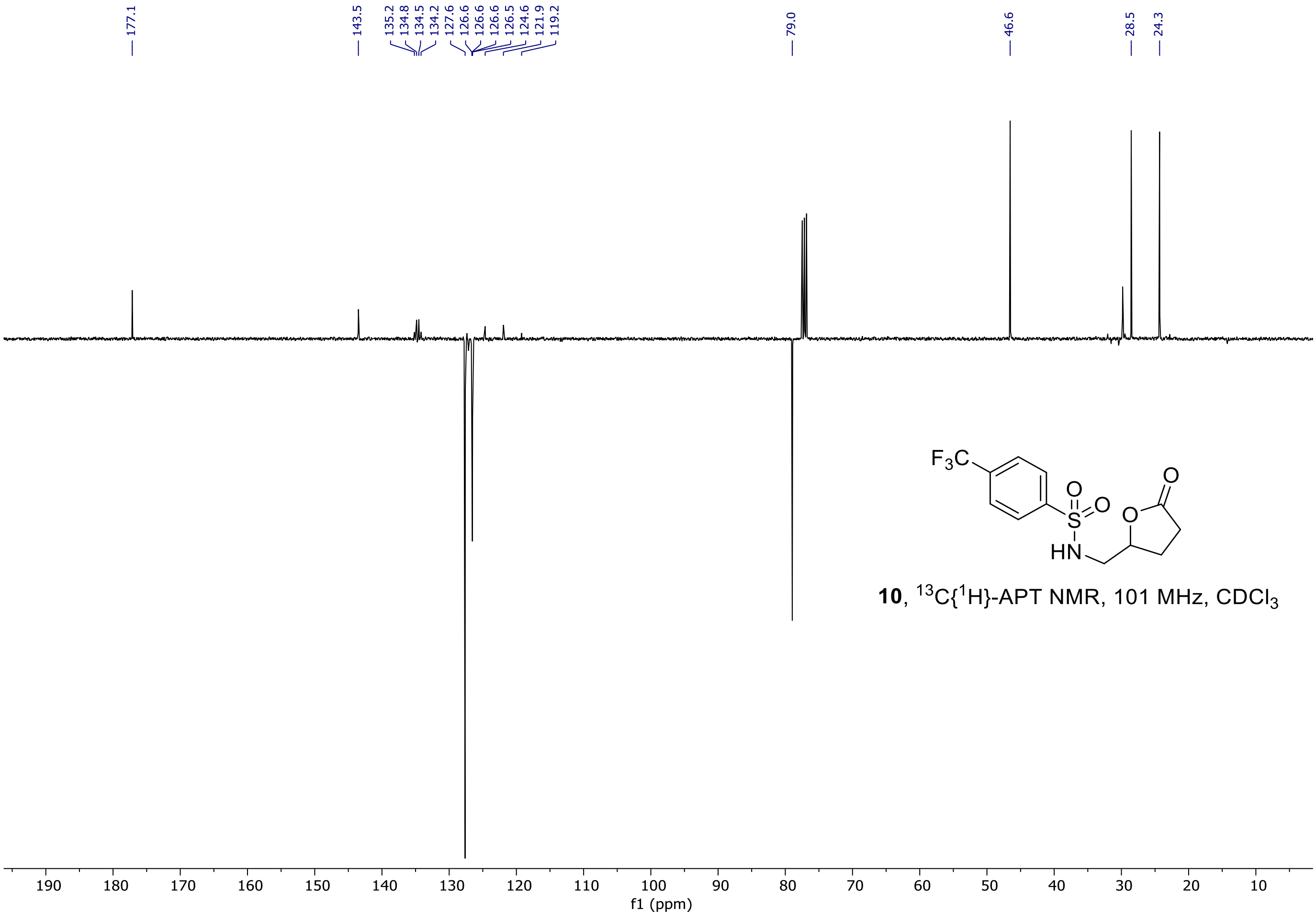

10, $^{13}C\{^{1}H\}$-APT NMR, 101 MHz, $CDCl_3$

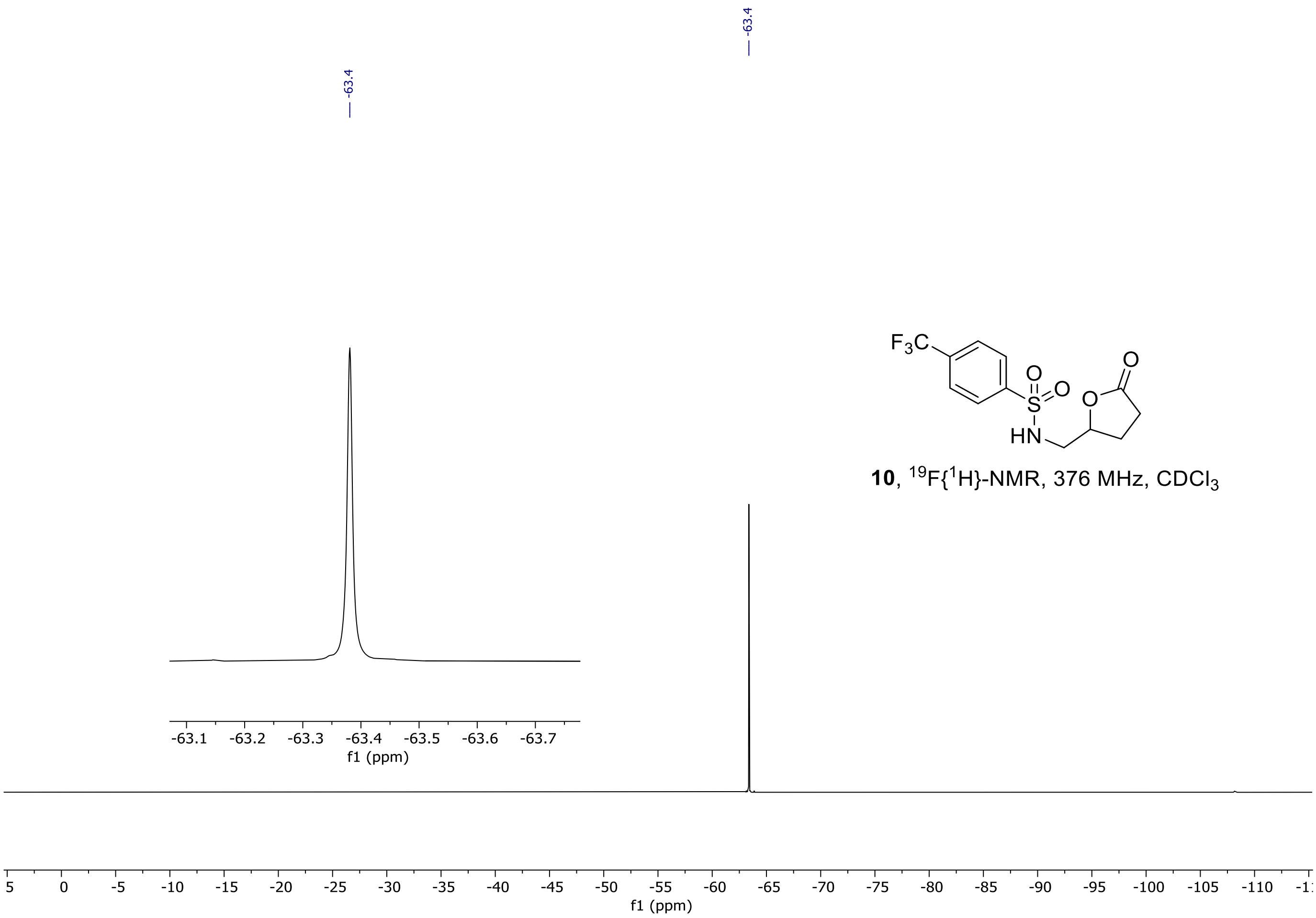
-63.4
-63.4
-63.1 -63.2 -63.3 -63.4 -63.5 -63.6 -63.7
f1 (ppm)
F3C
O
S
O
HN
O
O
10, 19F{1H}-NMR, 376 MHz, CDCl3
5 0 -5 -10 -15 -20 -25 -30 -35 -40 -45 -50 -55 -60 -65 -70 -75 -80 -85 -90 -95 -100 -105 -110
f1 (ppm)

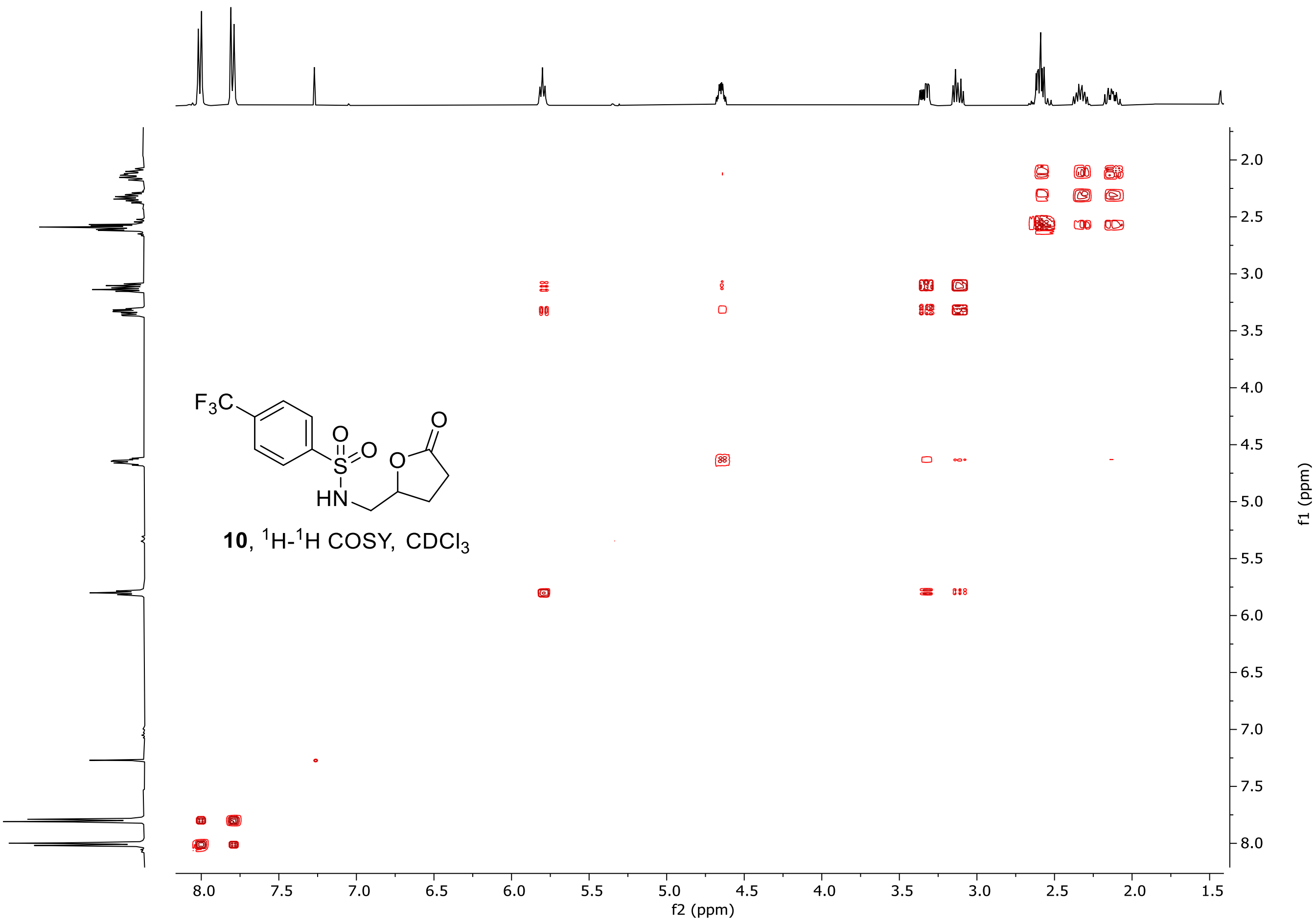
F3C
O
S
O
HN
O
O
10, 1H-1H COSY, CDCl3
8.0 7.5 7.0 6.5 6.0 5.5 5.0 4.5 4.0 3.5 3.0 2.5 2.0 1.5
f2 (ppm)
2.0 2.5 3.0 3.5 4.0 4.5 5.0 5.5 6.0 6.5 7.0 7.5 8.0
f1 (ppm)

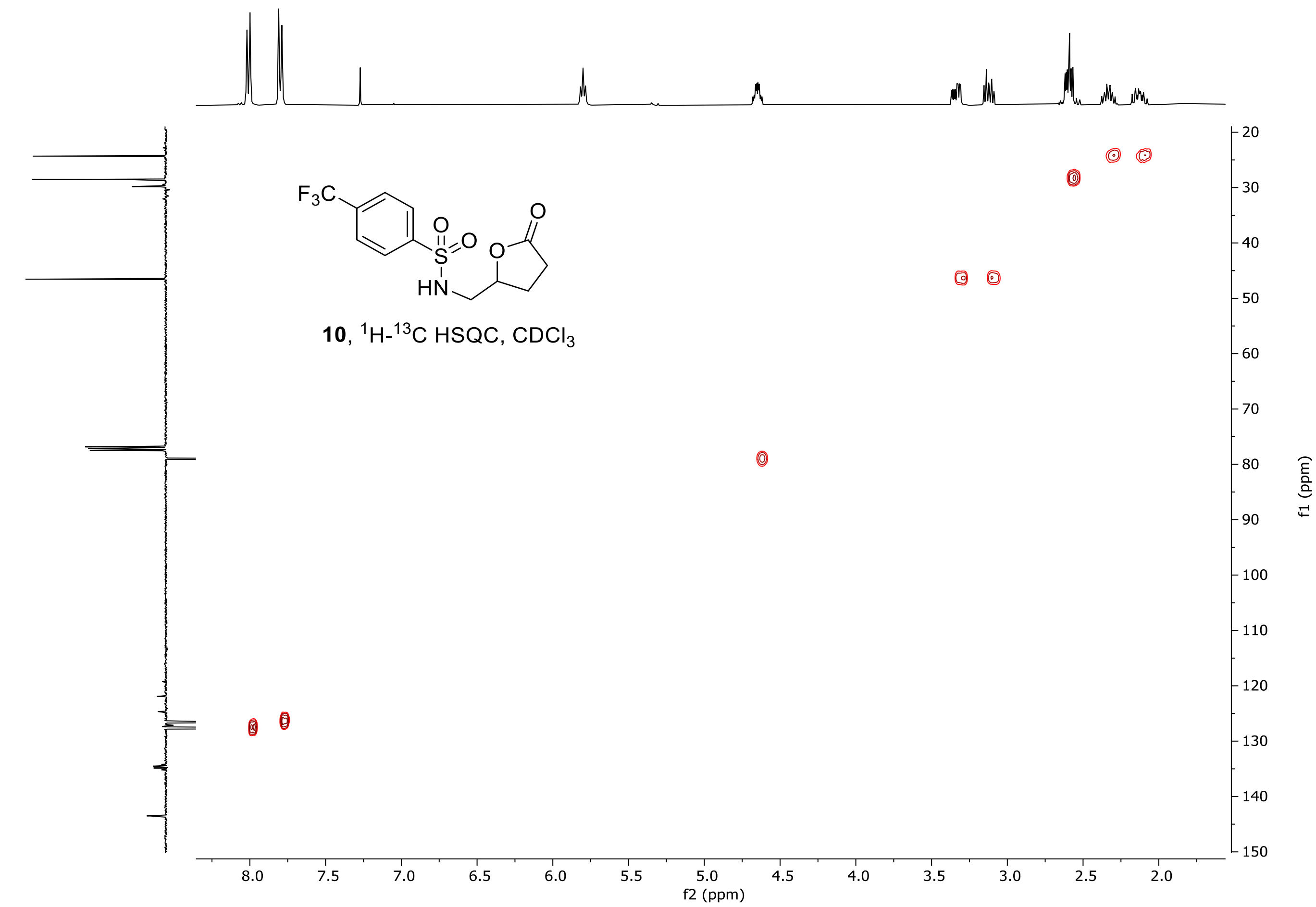

10, ^{1}H-^{13}C HSQC, $CDCl_3$

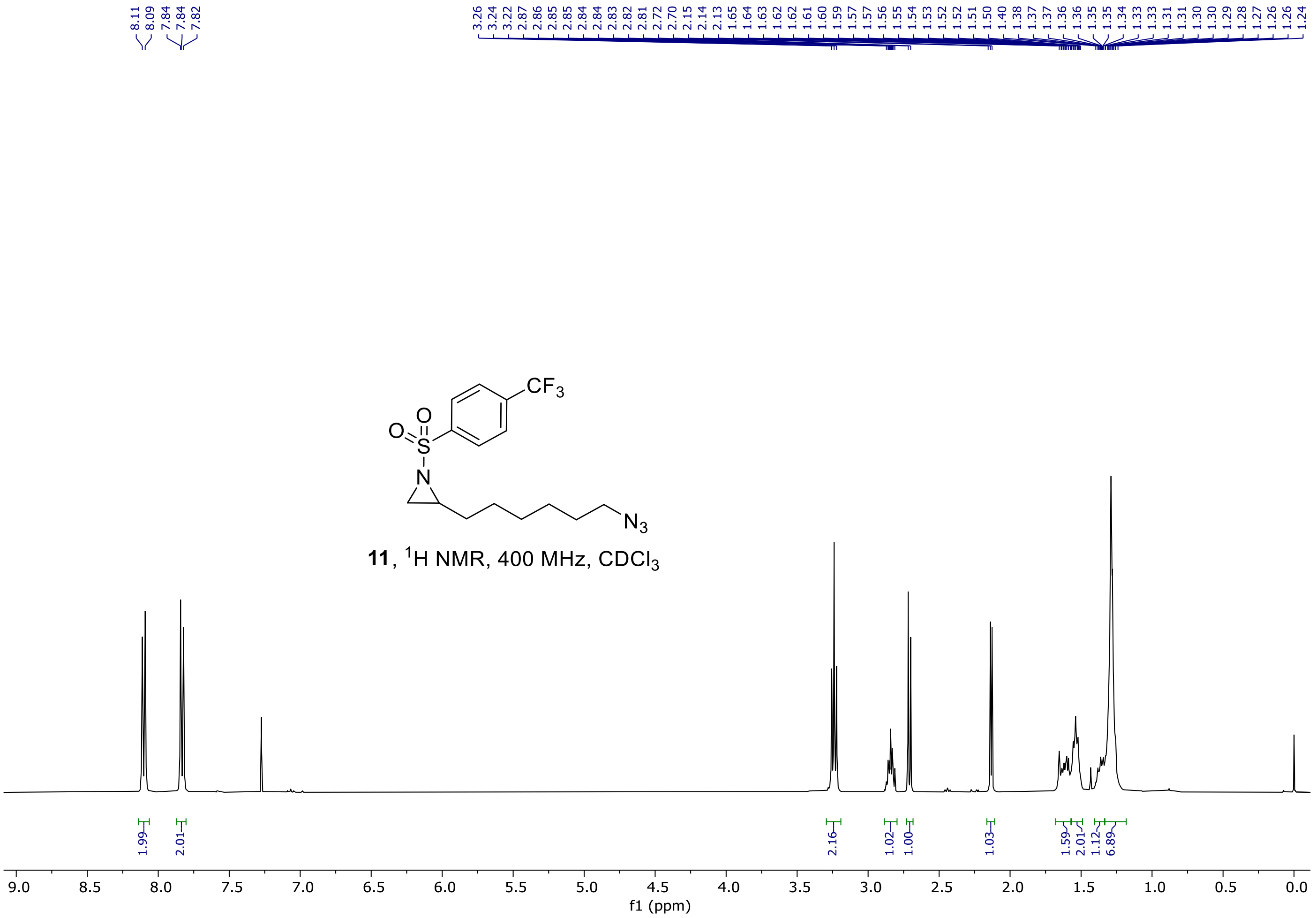

11, ^{1}H NMR, 400 MHz, $CDCl_3$

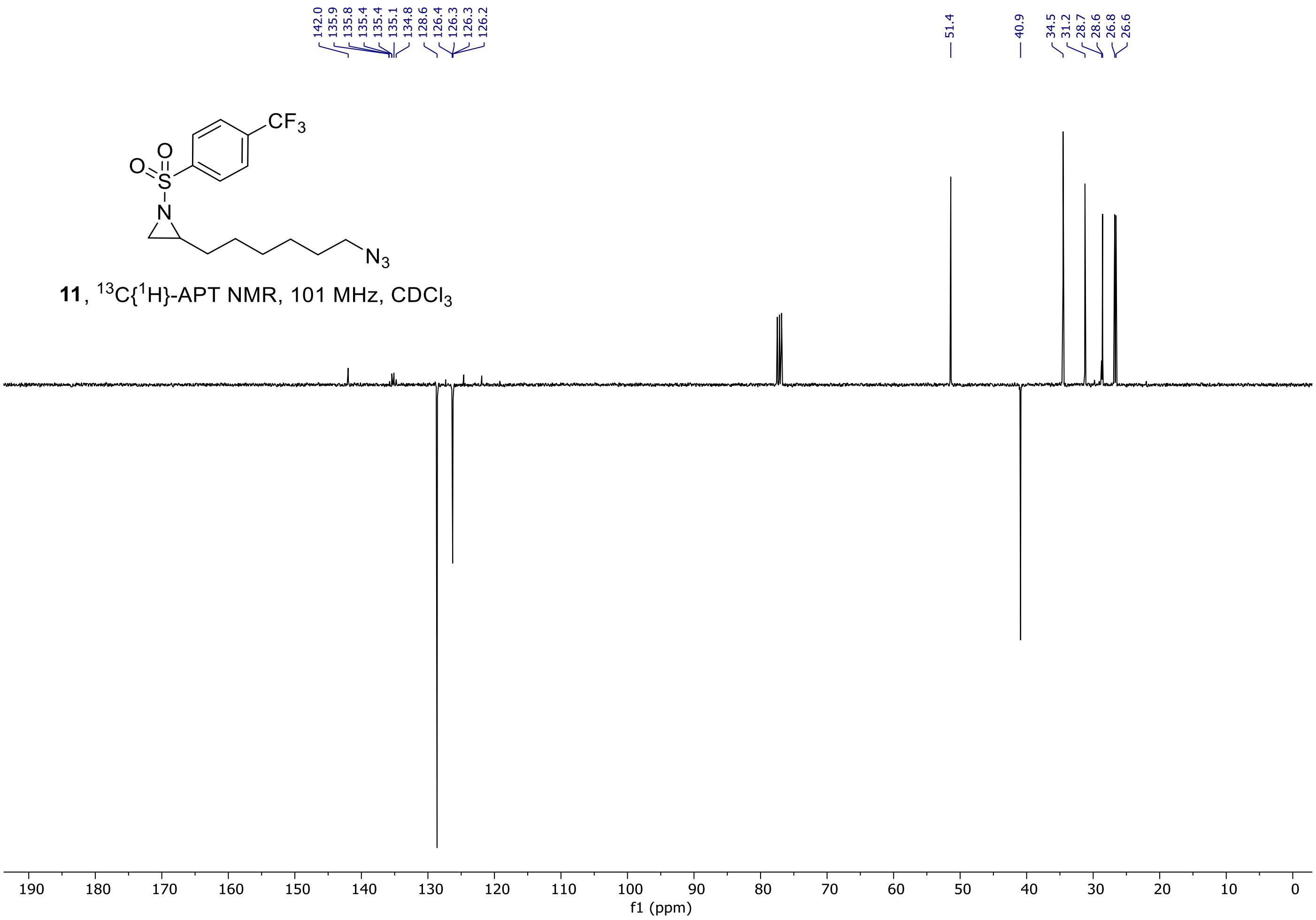

11, $^{13}C\{^{1}H\}$-APT NMR, 101 MHz, $CDCl_3$

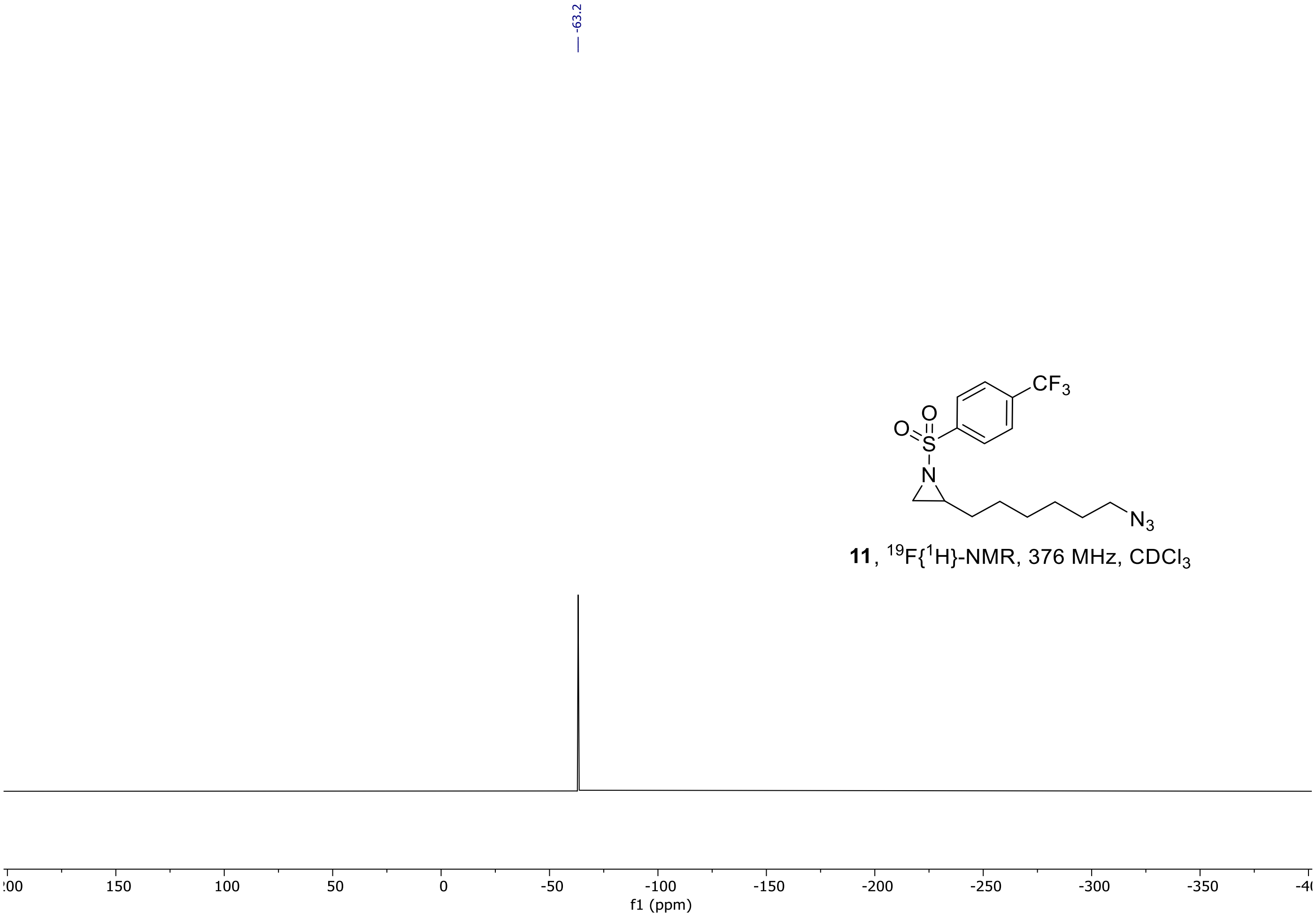

11, $^{19}F\{^{1}H\}$-NMR, 376 MHz, $CDCl_3$

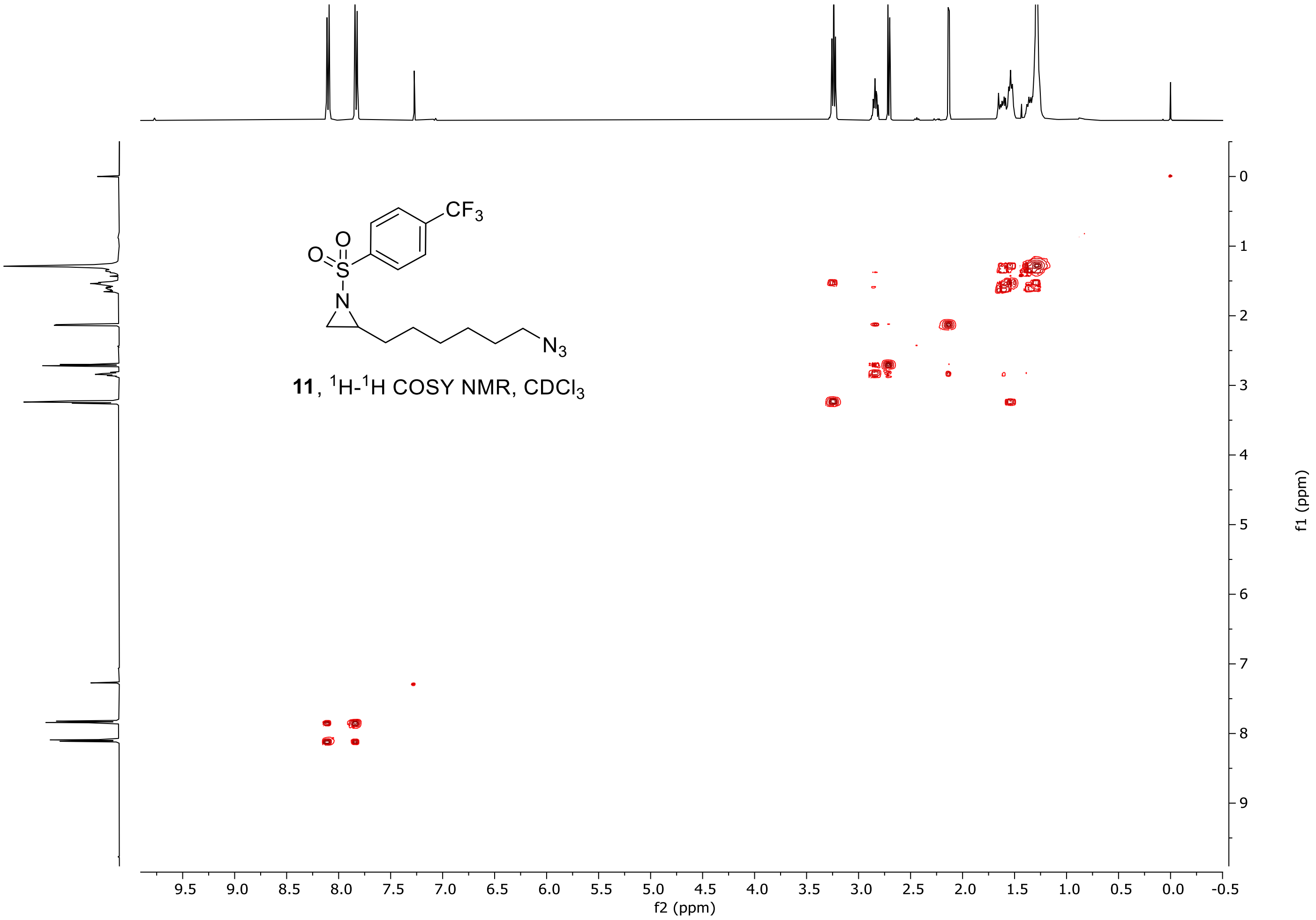
CF3
O
O
S
N
N3
11, 1H-1H COSY NMR, CDCl3
f2 (ppm)
f1 (ppm)

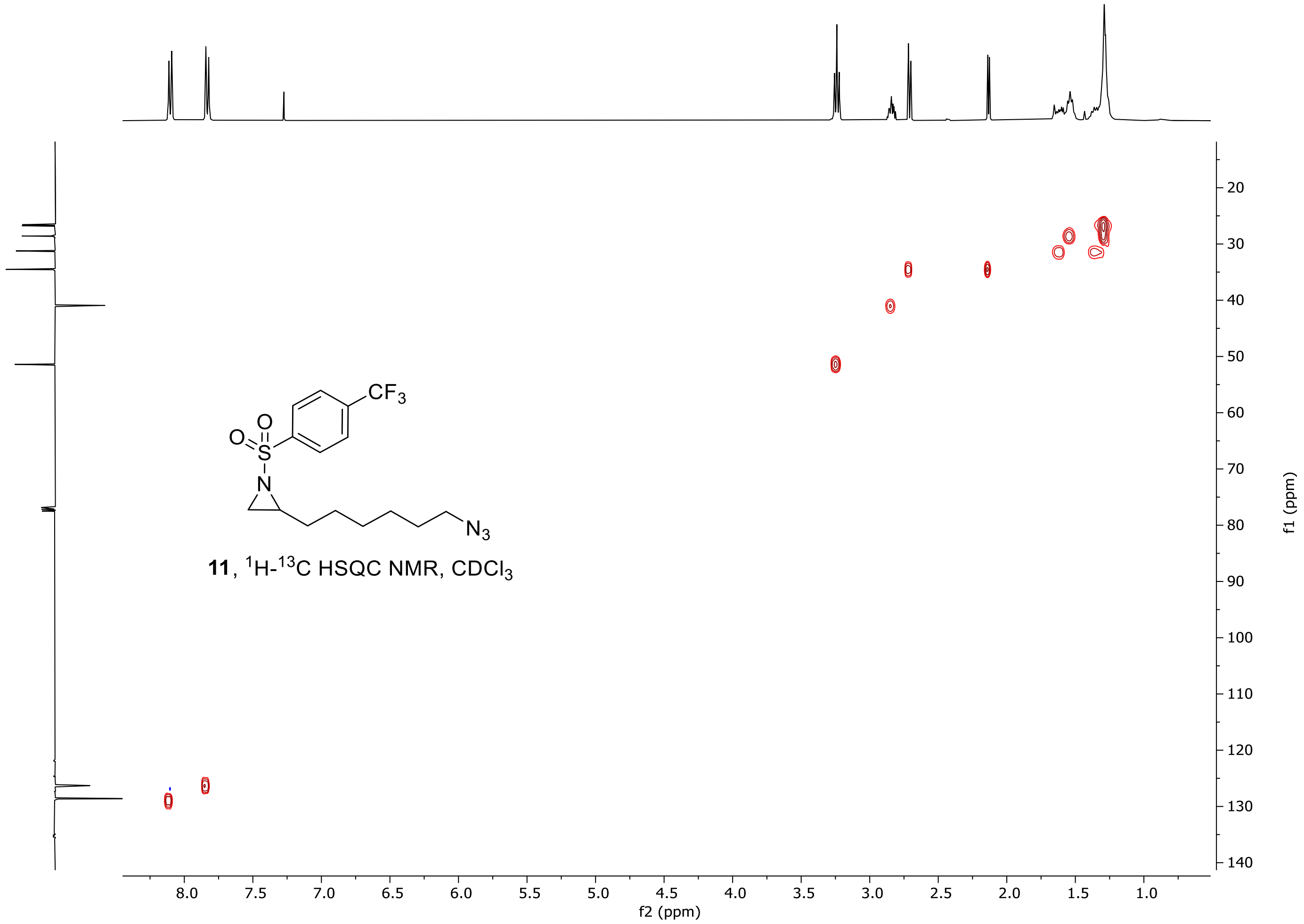
CF3
O
O
S
N
N3
11, 1H-13C HSQC NMR, CDCl3
f2 (ppm)
f1 (ppm)

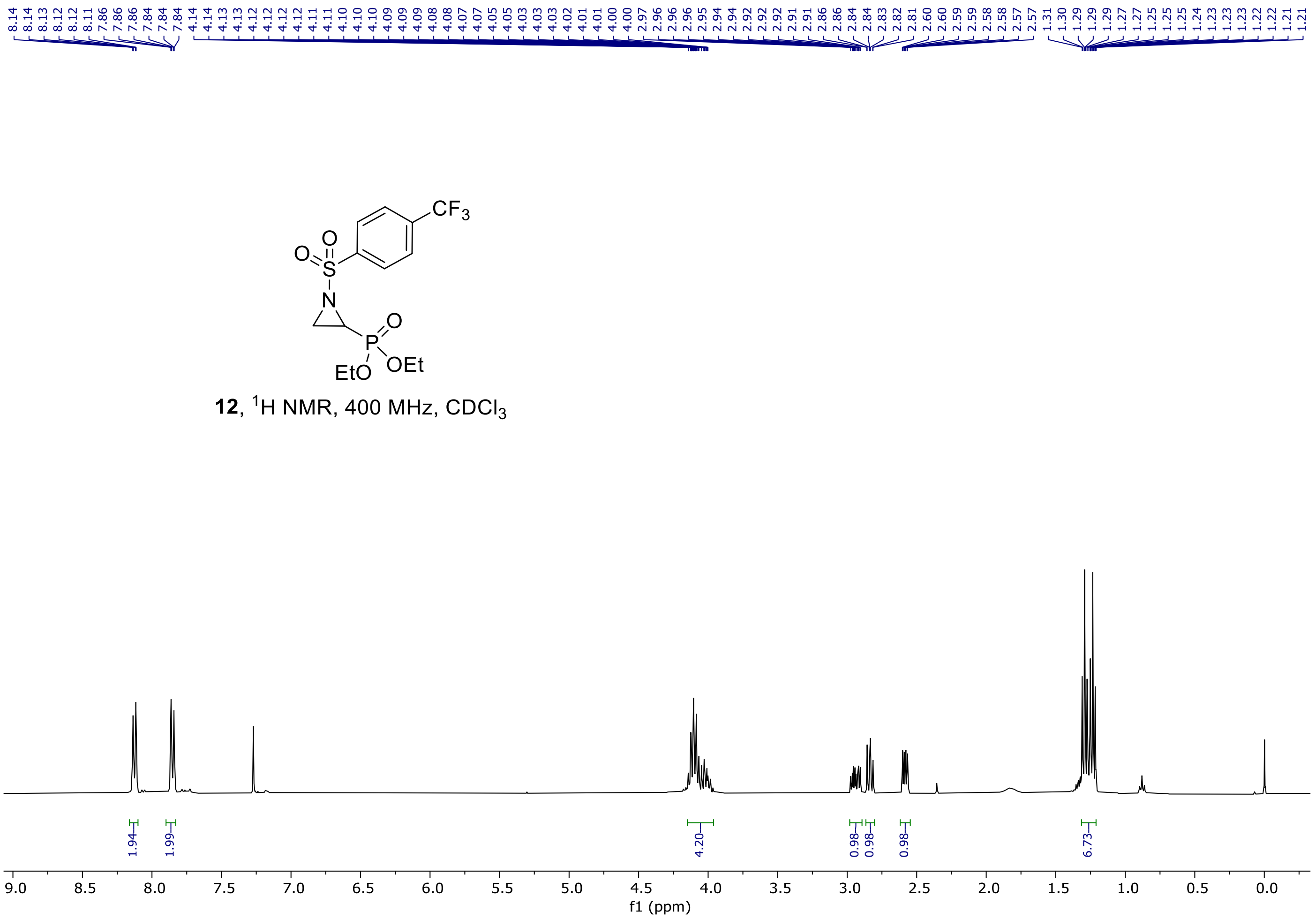

12, ^{1}H NMR, 400 MHz, $CDCl_3$

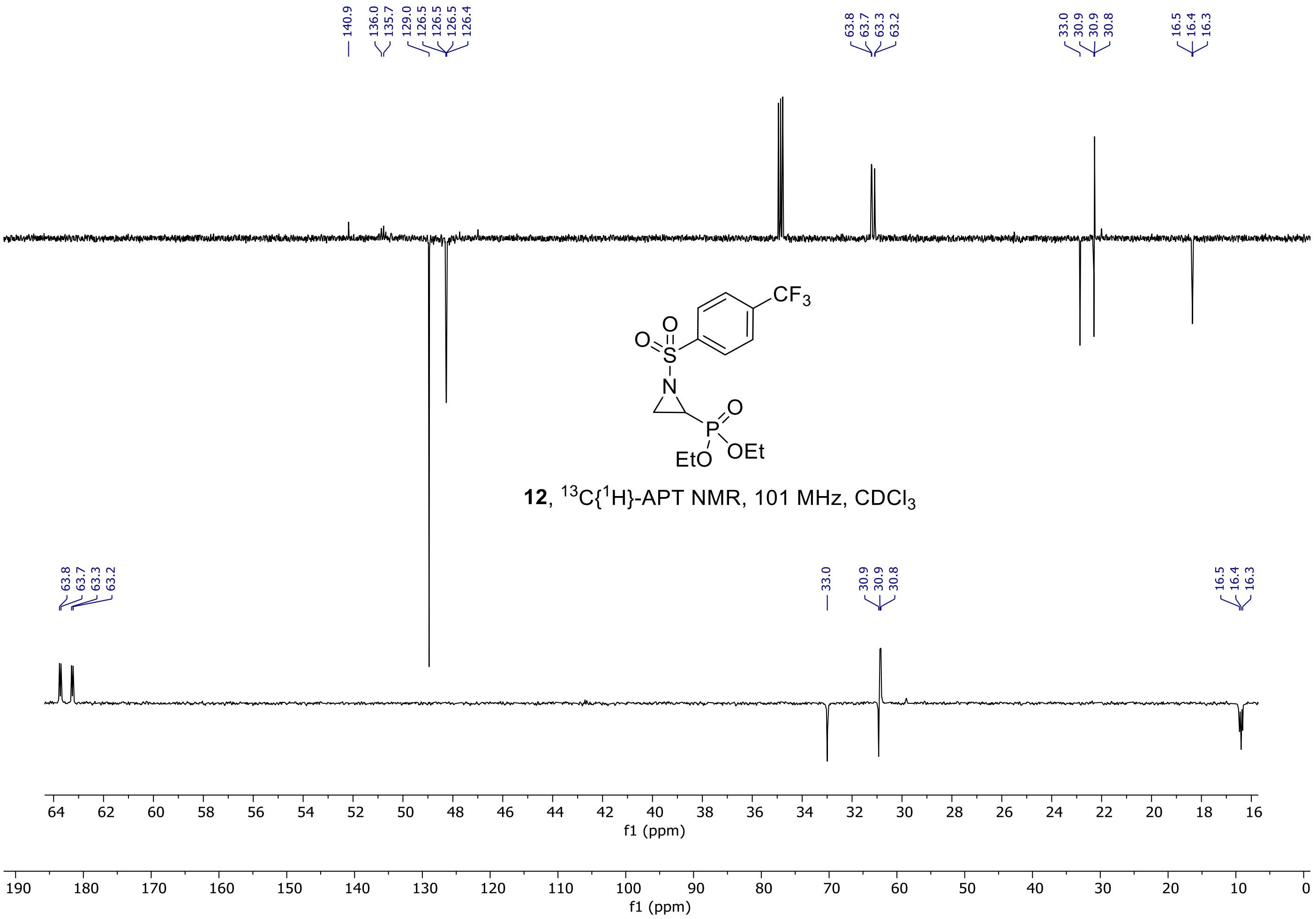

12, $^{13}C\{^{1}H\}$-APT NMR, 101 MHz, $CDCl_3$

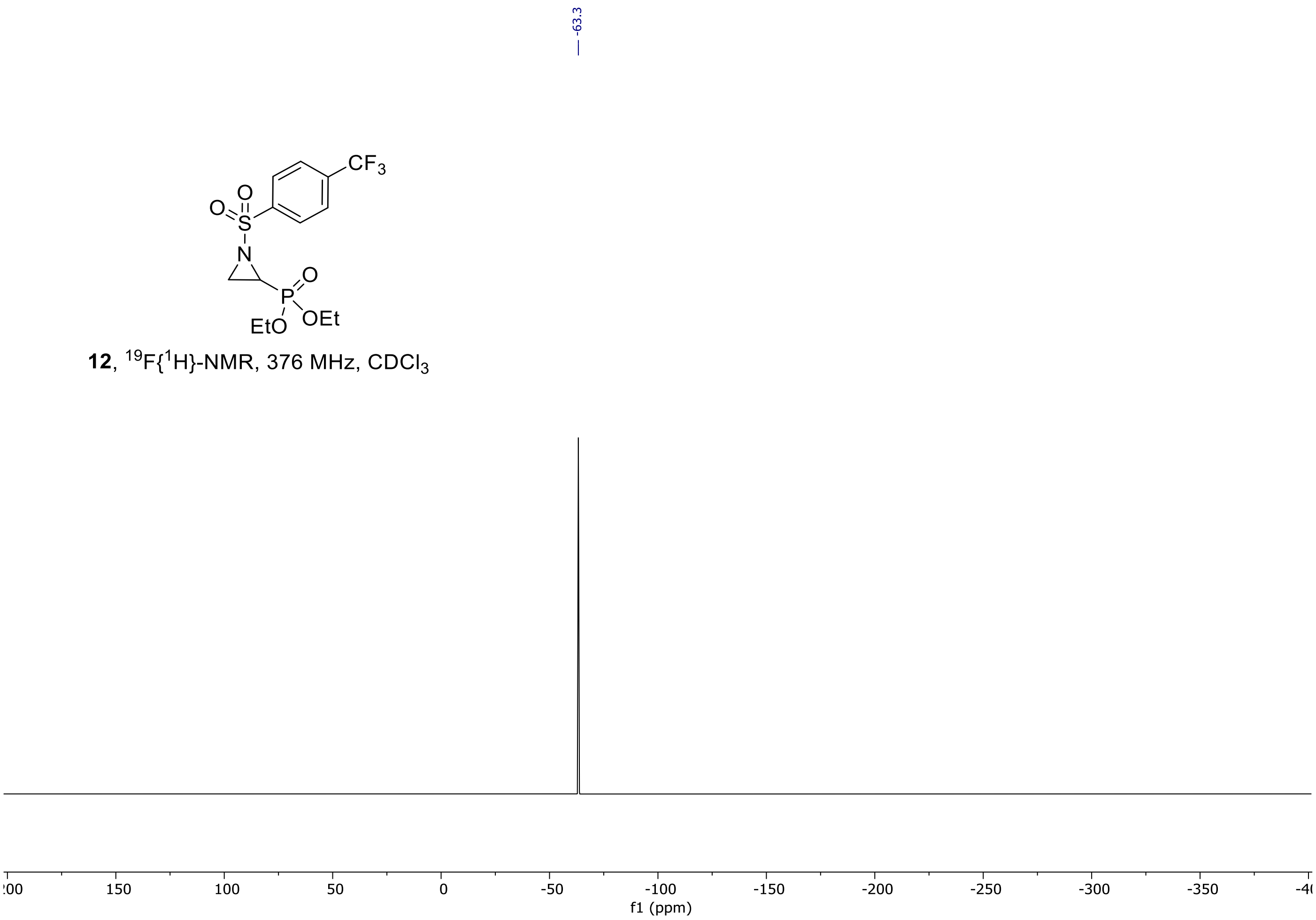
-63.3
CF3
O
O
S
N
P
O
OEt
EtO
12, 19F{1H}-NMR, 376 MHz, CDCl3
f1 (ppm)

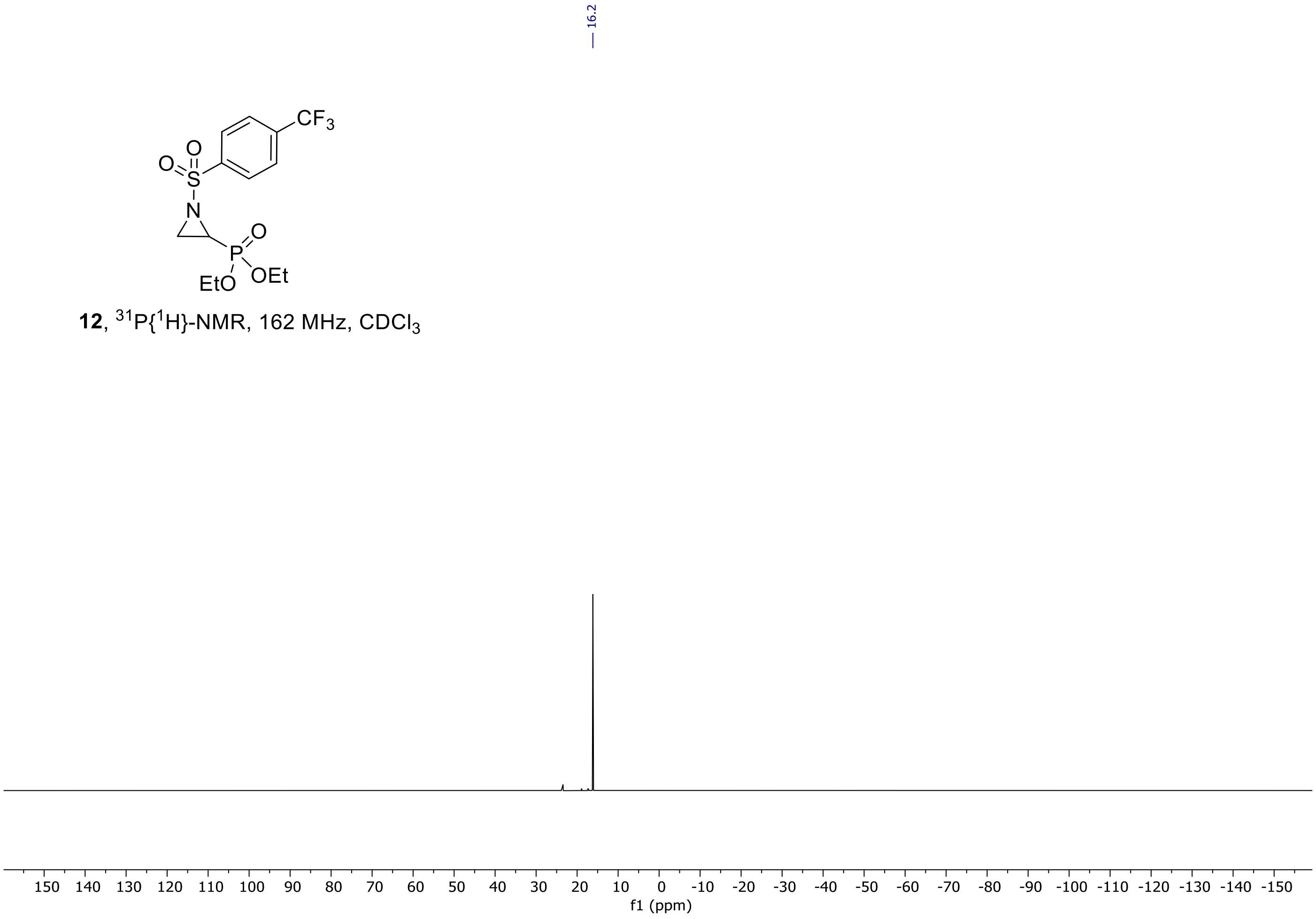
16.2
CF3
O
O
S
N
P
O
OEt
EtO
12, 31P{1H}-NMR, 162 MHz, CDCl3
f1 (ppm)

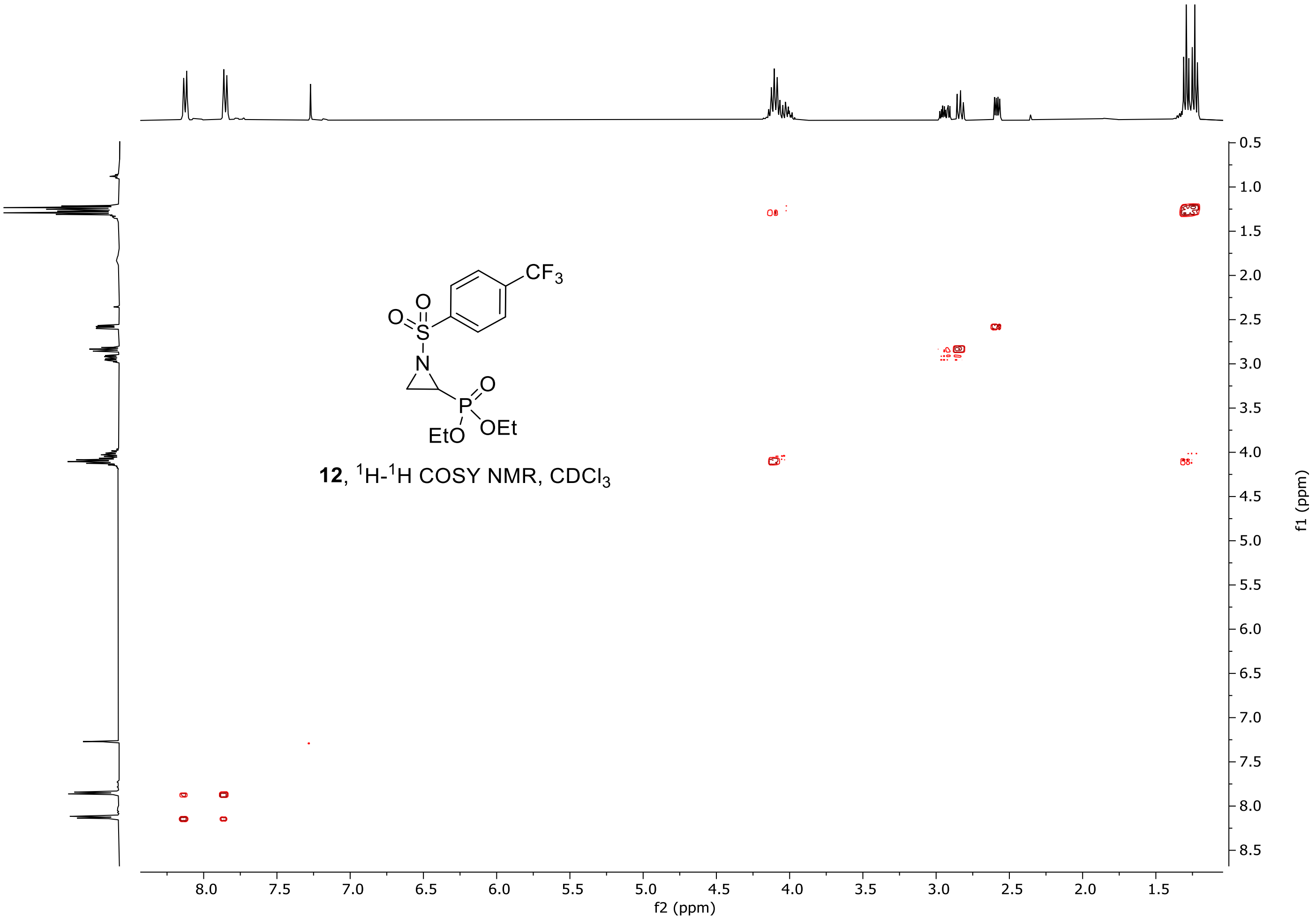
CF3
O
O
S
N
P
O
EtO
OEt
12, 1H-1H COSY NMR, CDCl3
f2 (ppm)
f1 (ppm)

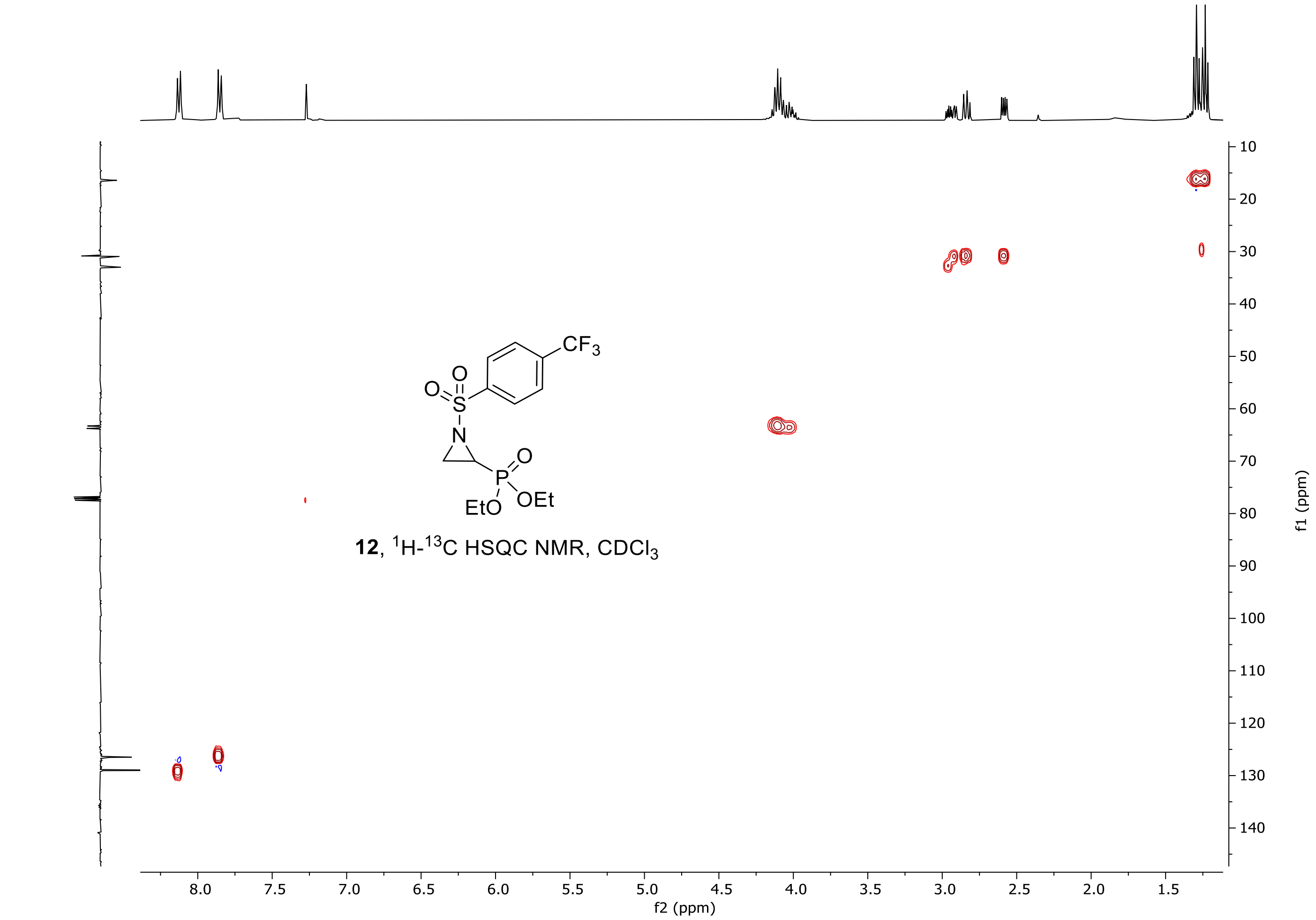
CF3
O
O
S
N
P
O
EtO
OEt
12, 1H-13C HSQC NMR, CDCl3
f2 (ppm)
f1 (ppm)

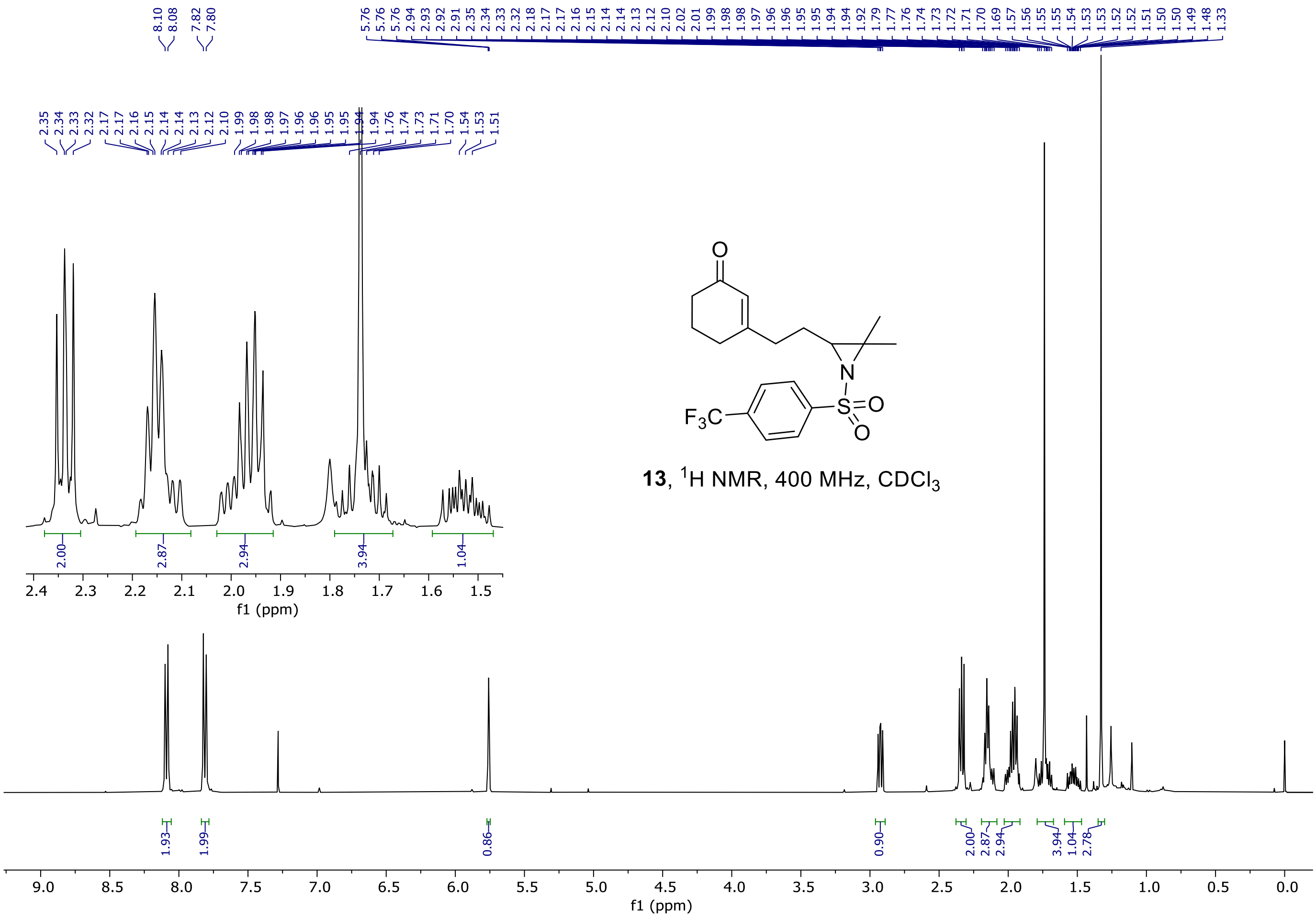
13, ^{1}H NMR, 400 MHz, CDCl$_3$
f1 (ppm)

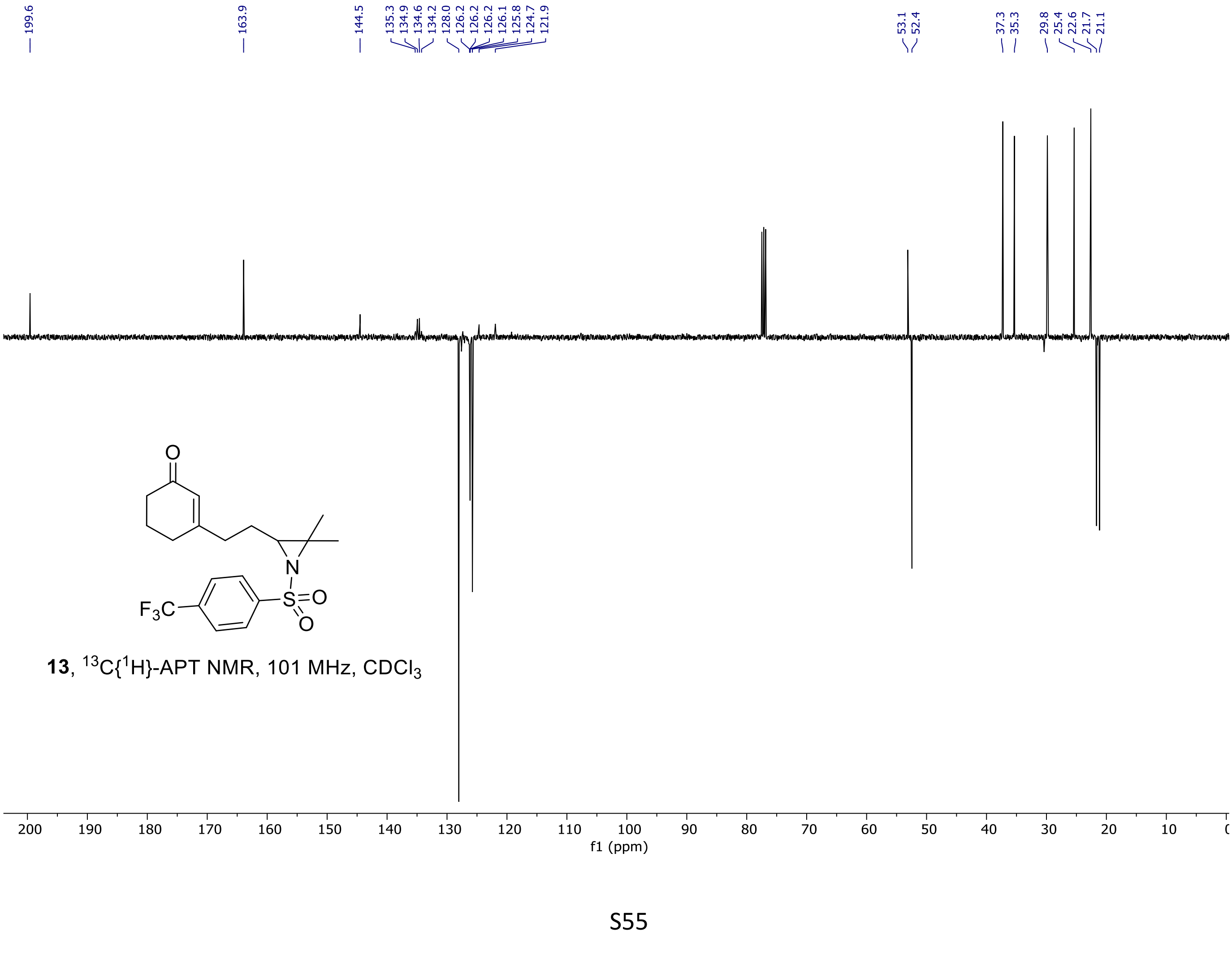
13, ^{13}C{^{1}H}-APT NMR, 101 MHz, CDCl$_3$
f1 (ppm)

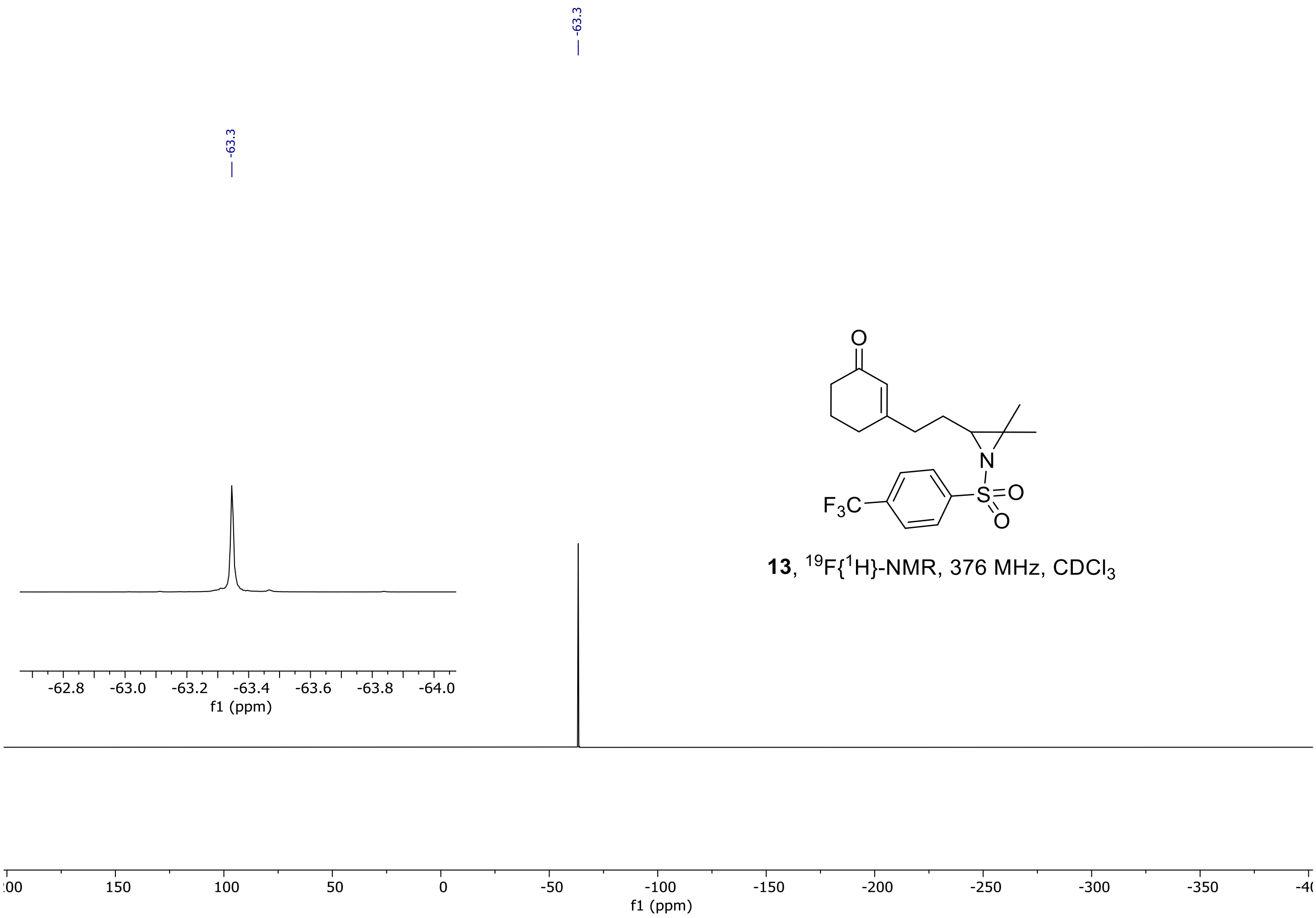

13, $^{19}F\{^{1}H\}$-NMR, 376 MHz, $CDCl_3$

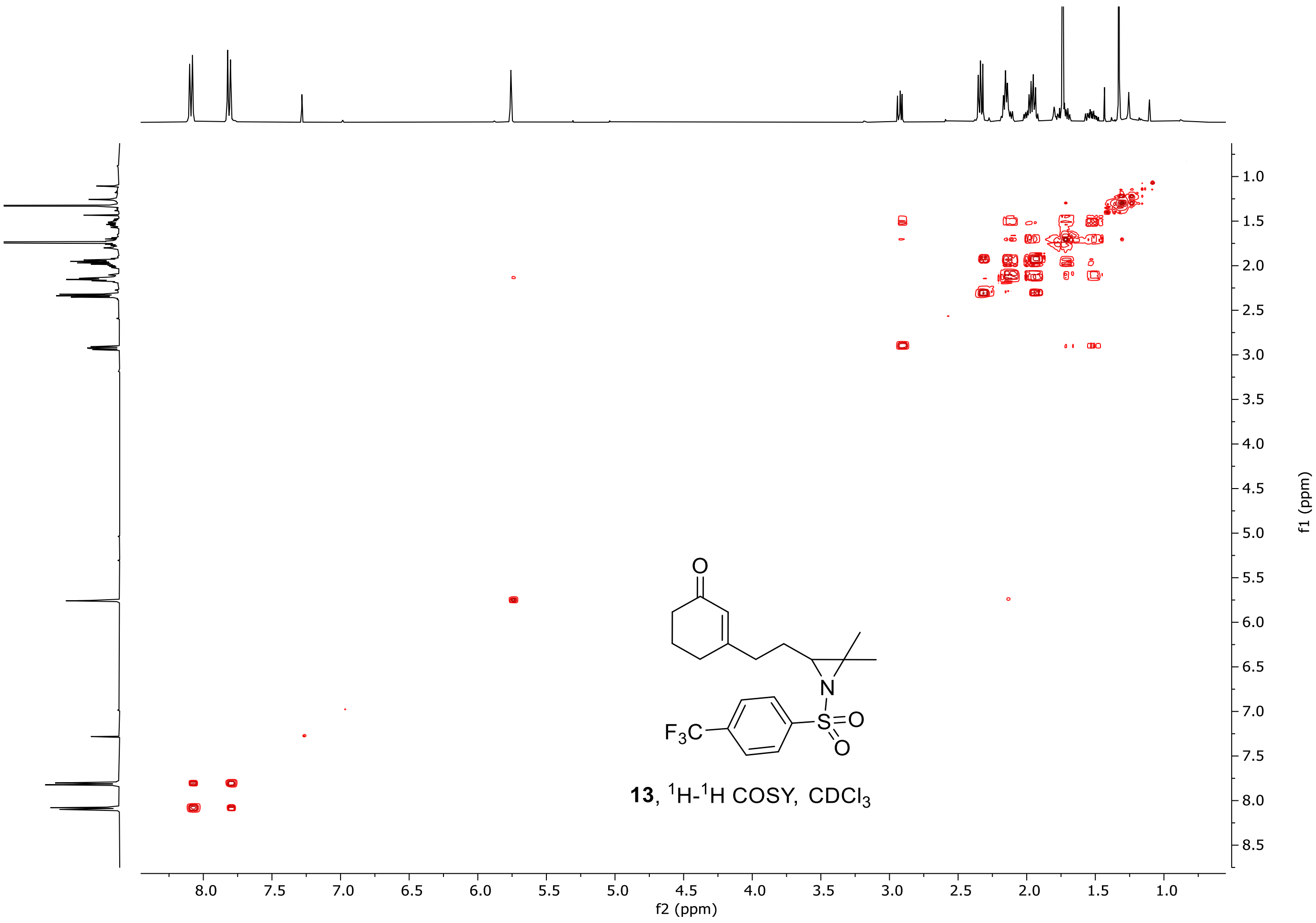

13, ^{1}H-^{1}H COSY, $CDCl_3$

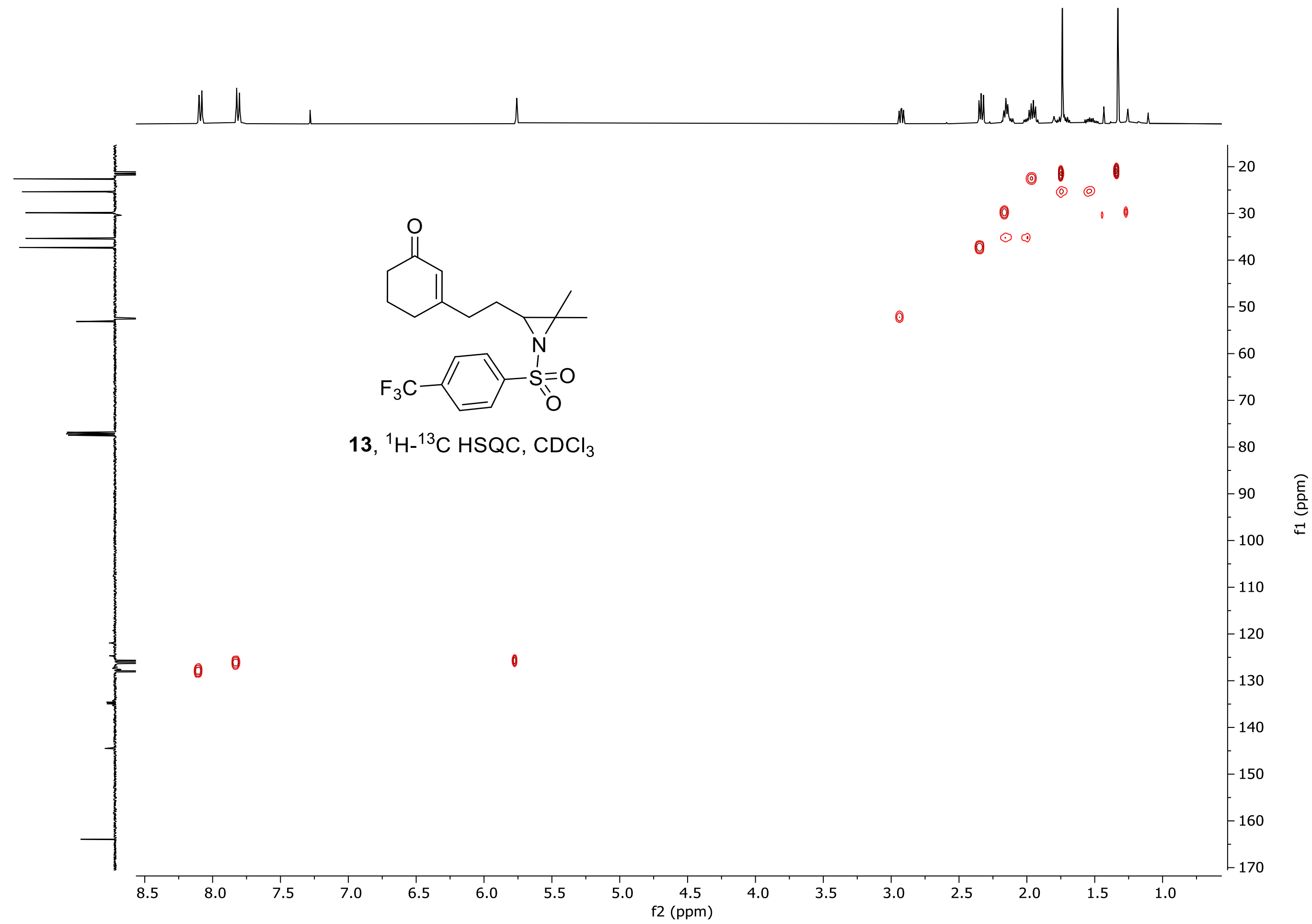
13, ^{1}H-^{13}C HSQC, $CDCl_3$
f2 (ppm)
f1 (ppm)

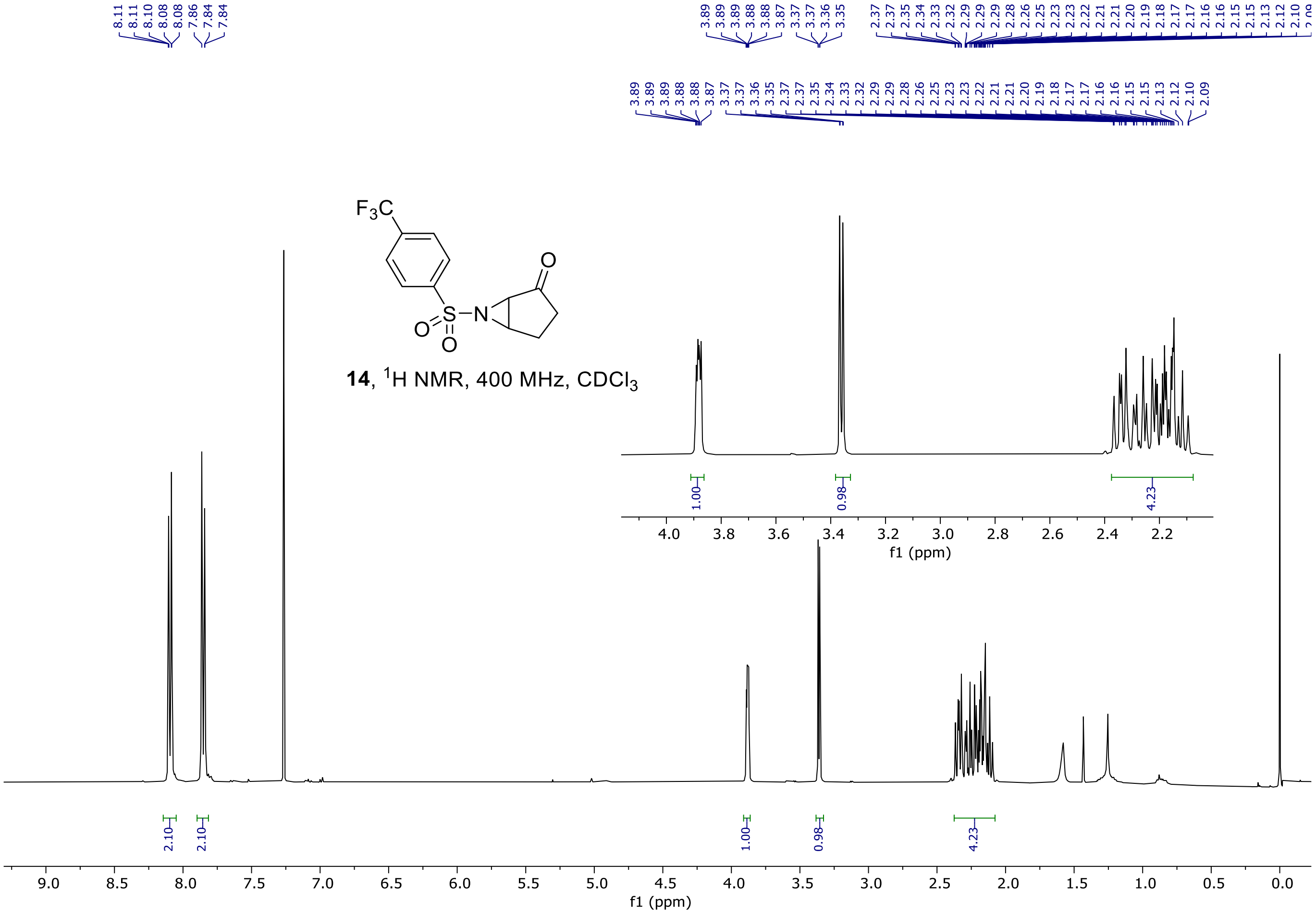
14, ^{1}H NMR, 400 MHz, $CDCl_3$
f1 (ppm)

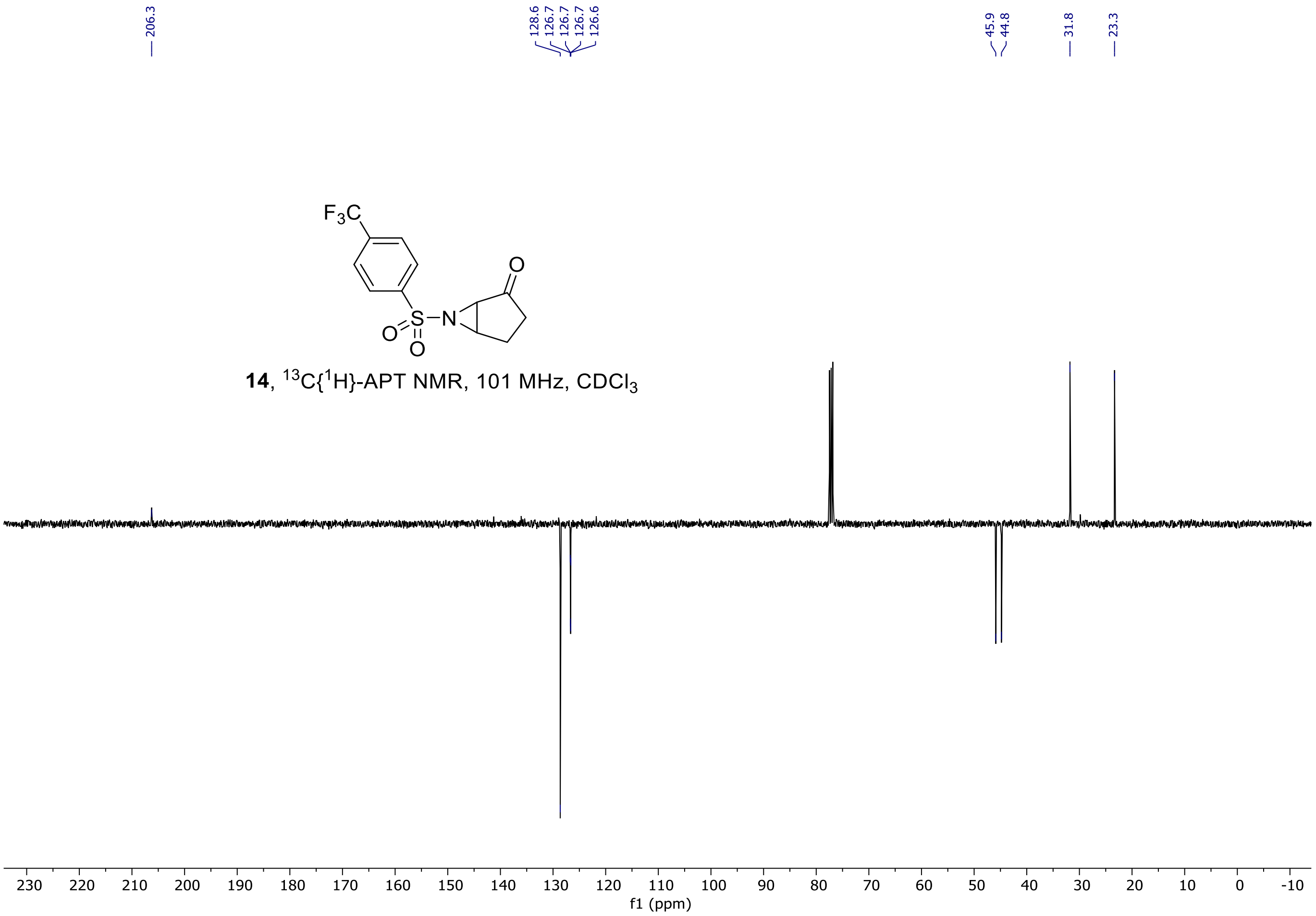
206.3
128.6
126.7
126.7
126.7
126.6
45.9
44.8
31.8
23.3
F3C
O
S
N
O
O
14, 13C{1H}-APT NMR, 101 MHz, CDCl3
230 220 210 200 190 180 170 160 150 140 130 120 110 100 90 80 70 60 50 40 30 20 10 0 -10
f1 (ppm)

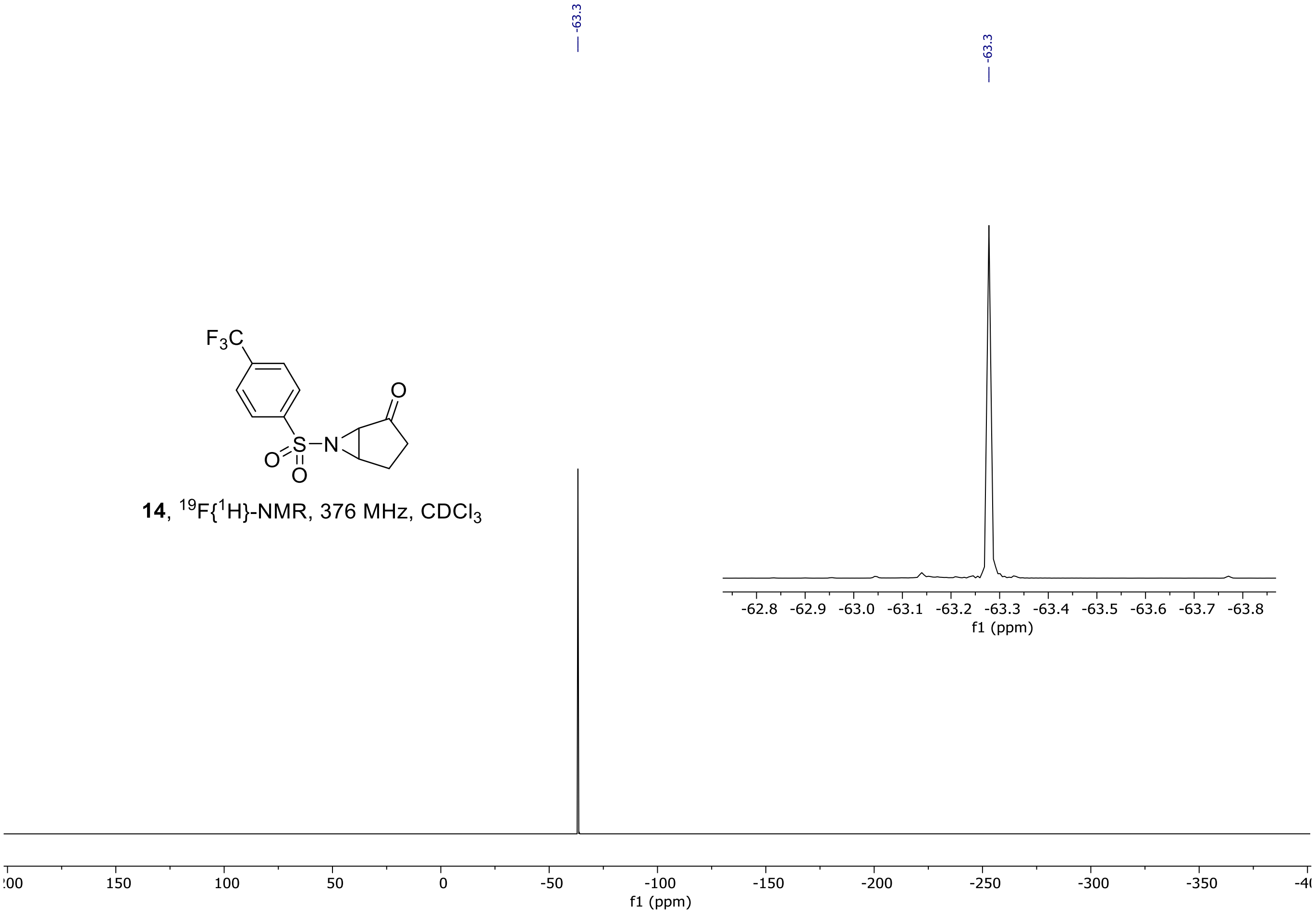
-63.3
-63.3
F3C
O
S
N
O
O
14, 19F{1H}-NMR, 376 MHz, CDCl3
-62.8 -62.9 -63.0 -63.1 -63.2 -63.3 -63.4 -63.5 -63.6 -63.7 -63.8
f1 (ppm)
150 100 50 0 -50 -100 -150 -200 -250 -300 -350
f1 (ppm)

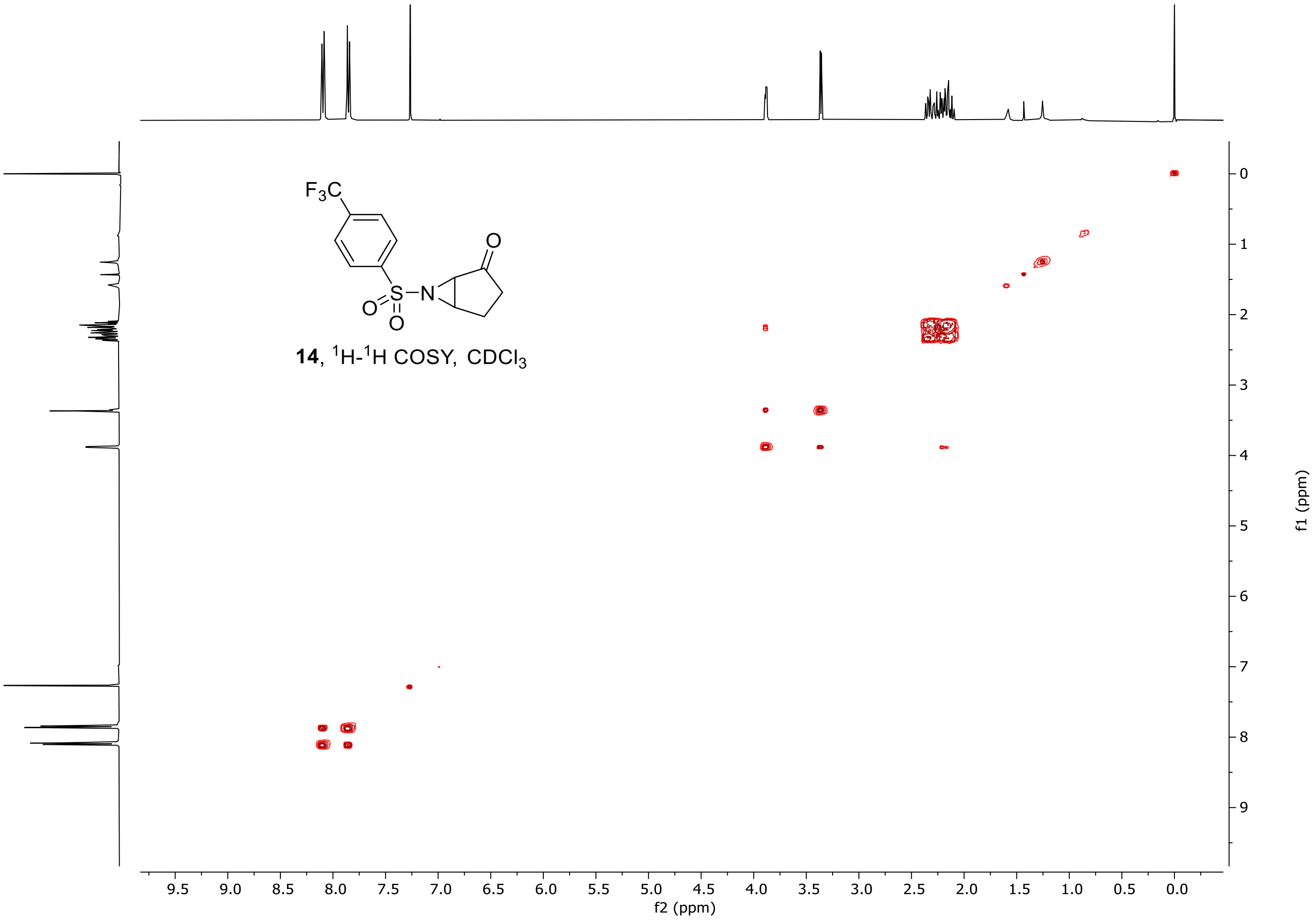
F3C
O
O=S=O
N
14, 1H-1H COSY, CDCl3
f1 (ppm)
f2 (ppm)

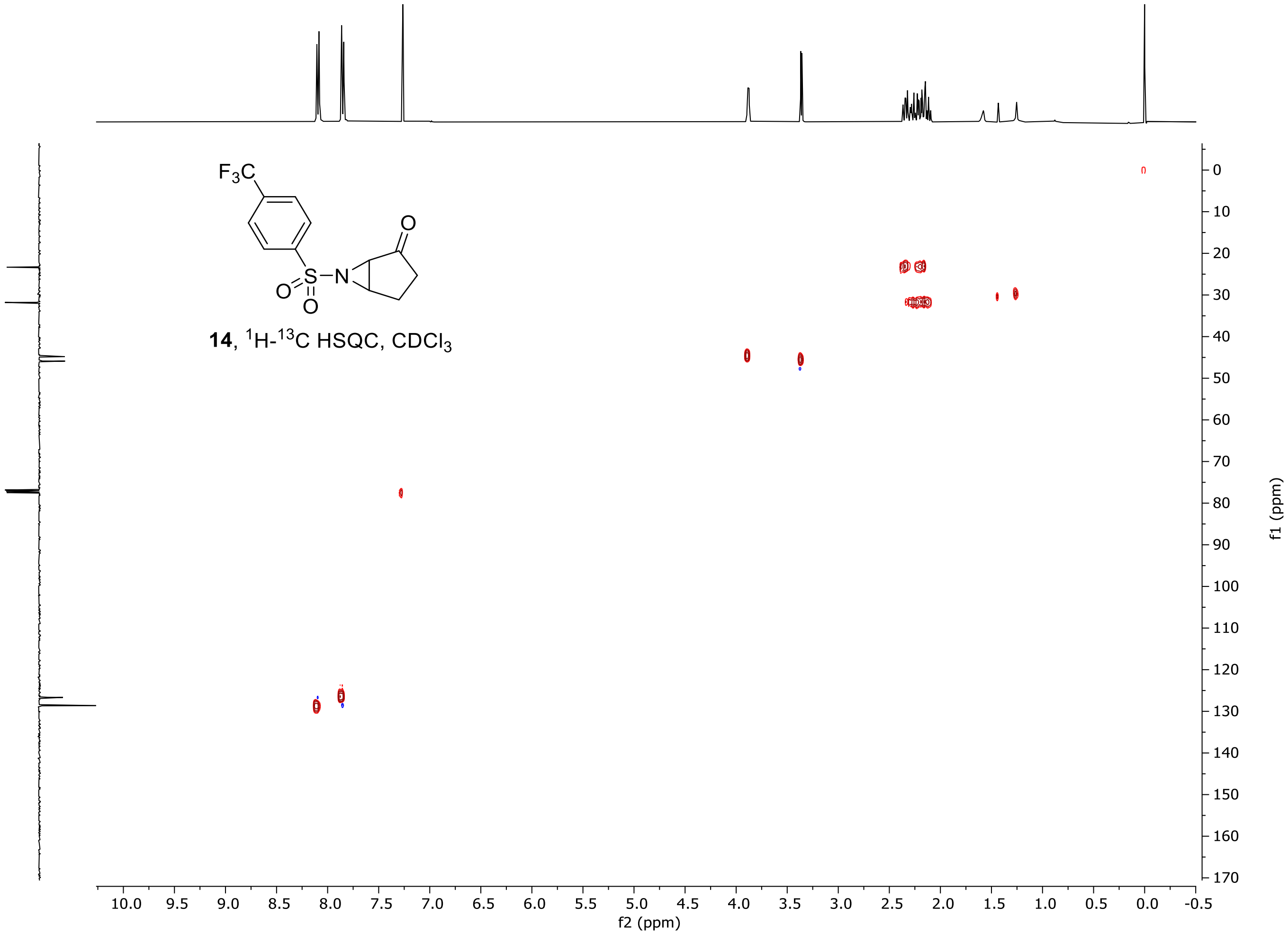
F3C
O
O=S=O
N
14, 1H-13C HSQC, CDCl3
f1 (ppm)
f2 (ppm)

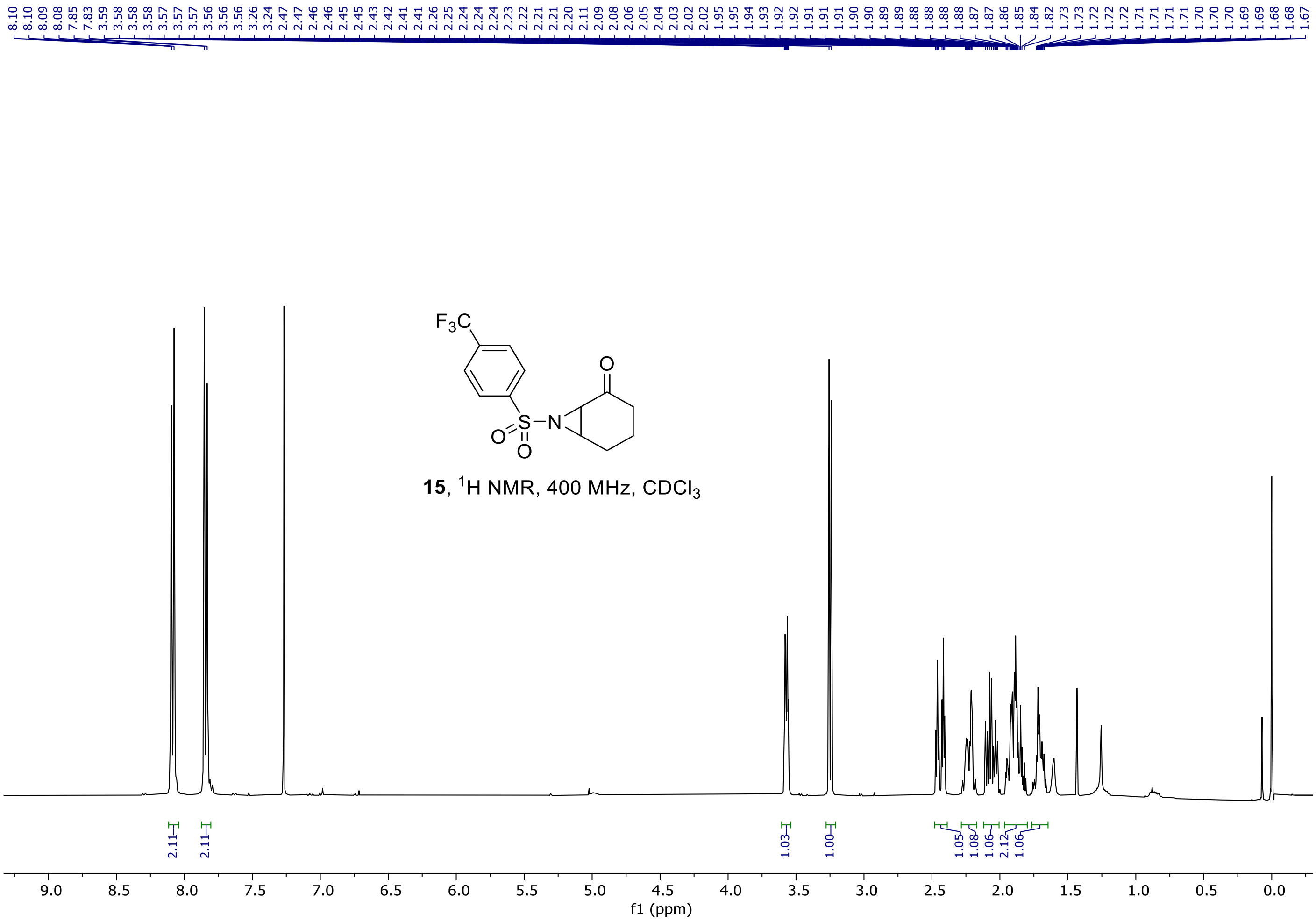
8.10 8.10 8.09 8.08 7.85 7.83 3.59 3.58 3.58 3.58 3.57 3.57 3.57 3.56 3.56 3.56 3.26 3.24 2.47 2.47 2.46 2.45 2.43 2.42 2.41 2.41 2.26 2.25 2.24 2.24 2.24 2.23 2.22 2.21 2.21 2.20 2.11 2.09 2.08 2.06 2.05 2.04 2.03 2.02 2.02 1.95 1.95 1.94 1.93 1.92 1.92 1.91 1.91 1.91 1.90 1.89 1.89 1.88 1.88 1.88 1.87 1.87 1.86 1.85 1.84 1.82 1.73 1.73 1.72 1.72 1.71 1.71 1.71 1.70 1.70 1.69 1.69 1.68 1.67
F3C
O
S
N
O
O
15, 1H NMR, 400 MHz, CDCl3
2.11 2.11 1.03 1.00 1.05 1.08 1.06 2.12 1.06
9.0 8.5 8.0 7.5 7.0 6.5 6.0 5.5 5.0 4.5 4.0 3.5 3.0 2.5 2.0 1.5 1.0 0.5 0.0
f1 (ppm)

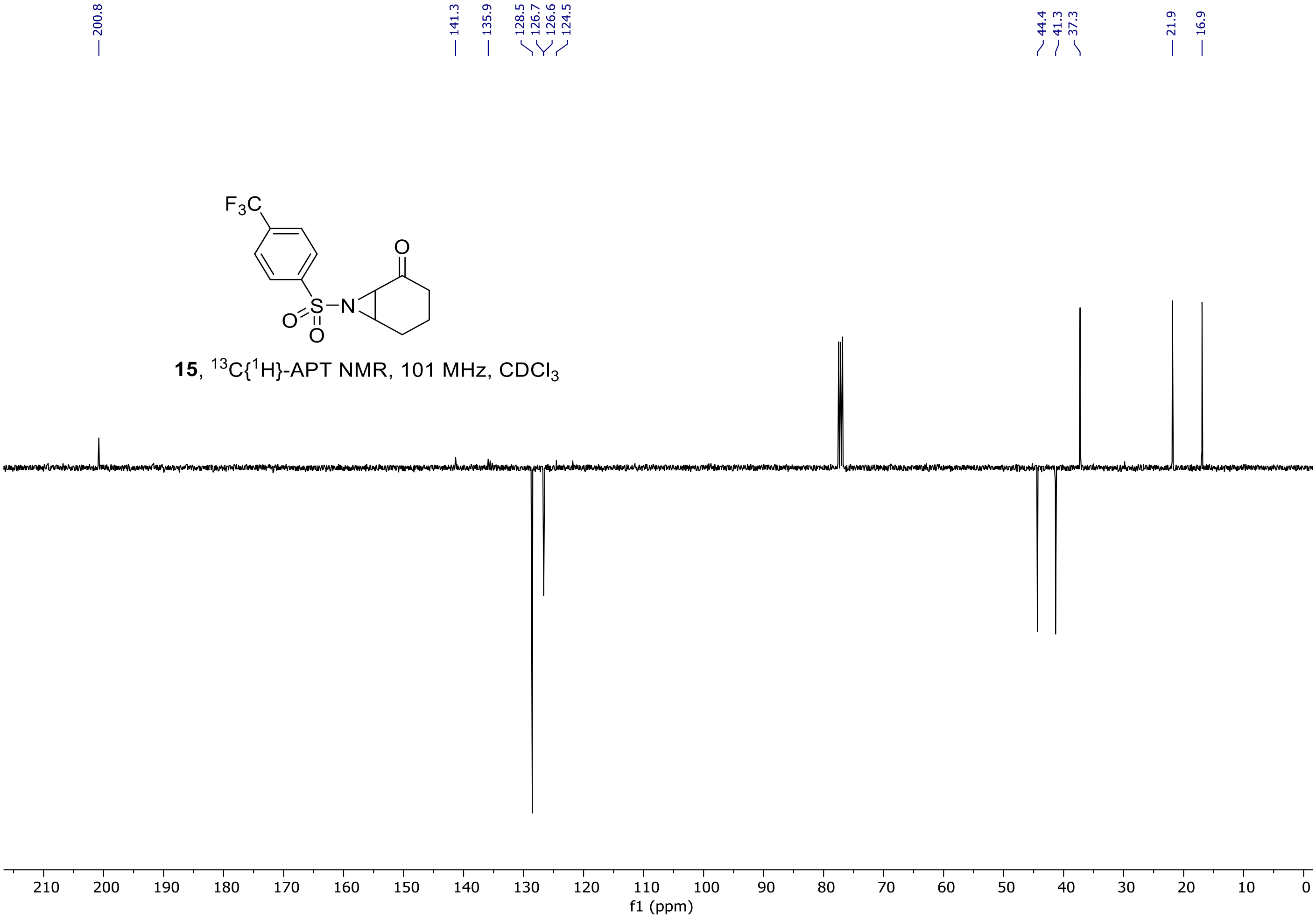
200.8 141.3 135.9 128.5 126.7 126.6 124.5 44.4 41.3 37.3 21.9 16.9
F3C
O
S
N
O
O
15, 13C{1H}-APT NMR, 101 MHz, CDCl3
210 200 190 180 170 160 150 140 130 120 110 100 90 80 70 60 50 40 30 20 10 0
f1 (ppm)

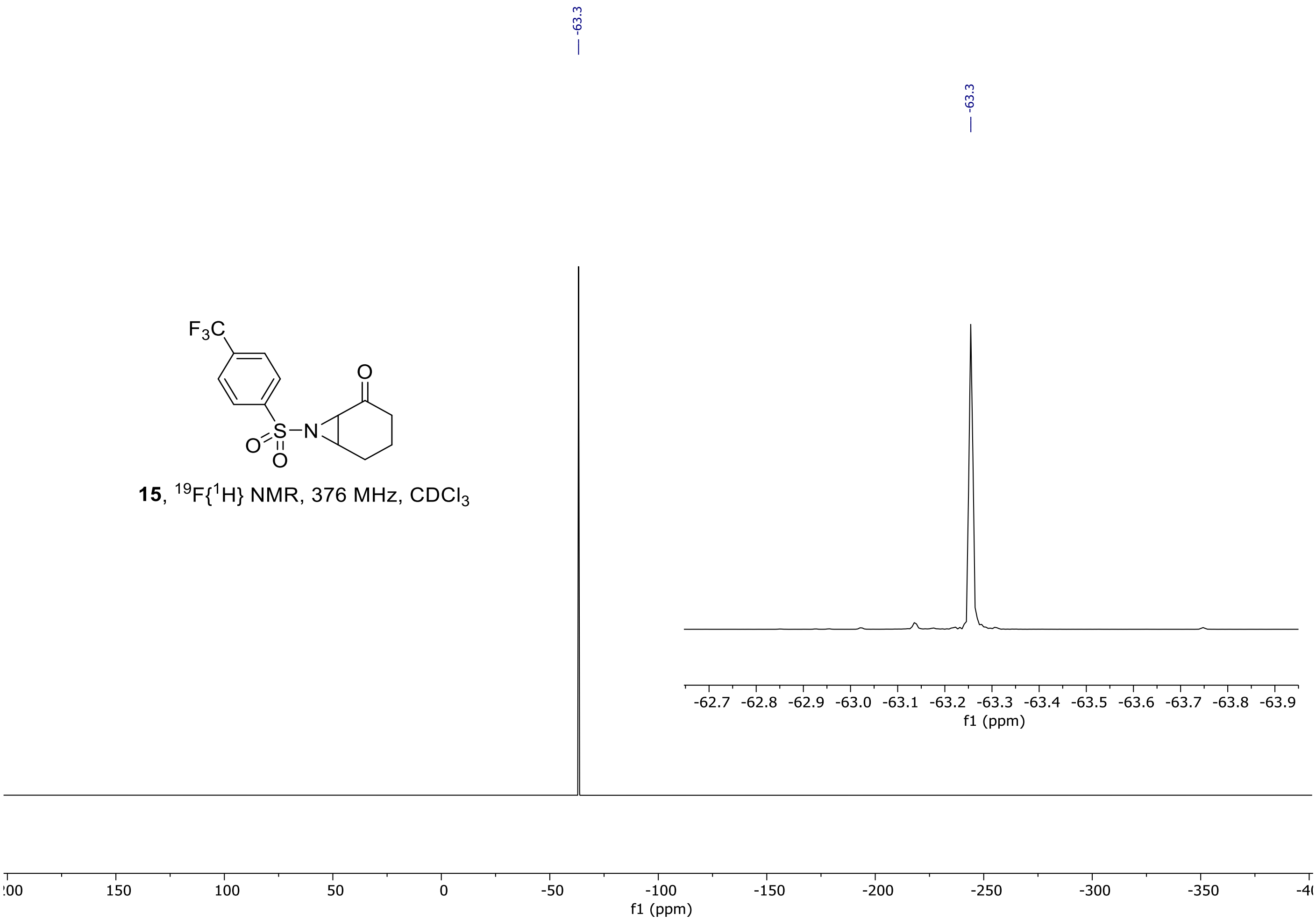
-63.3
-63.3
F3C
15, 19F{1H} NMR, 376 MHz, CDCl3
f1 (ppm)
f1 (ppm)

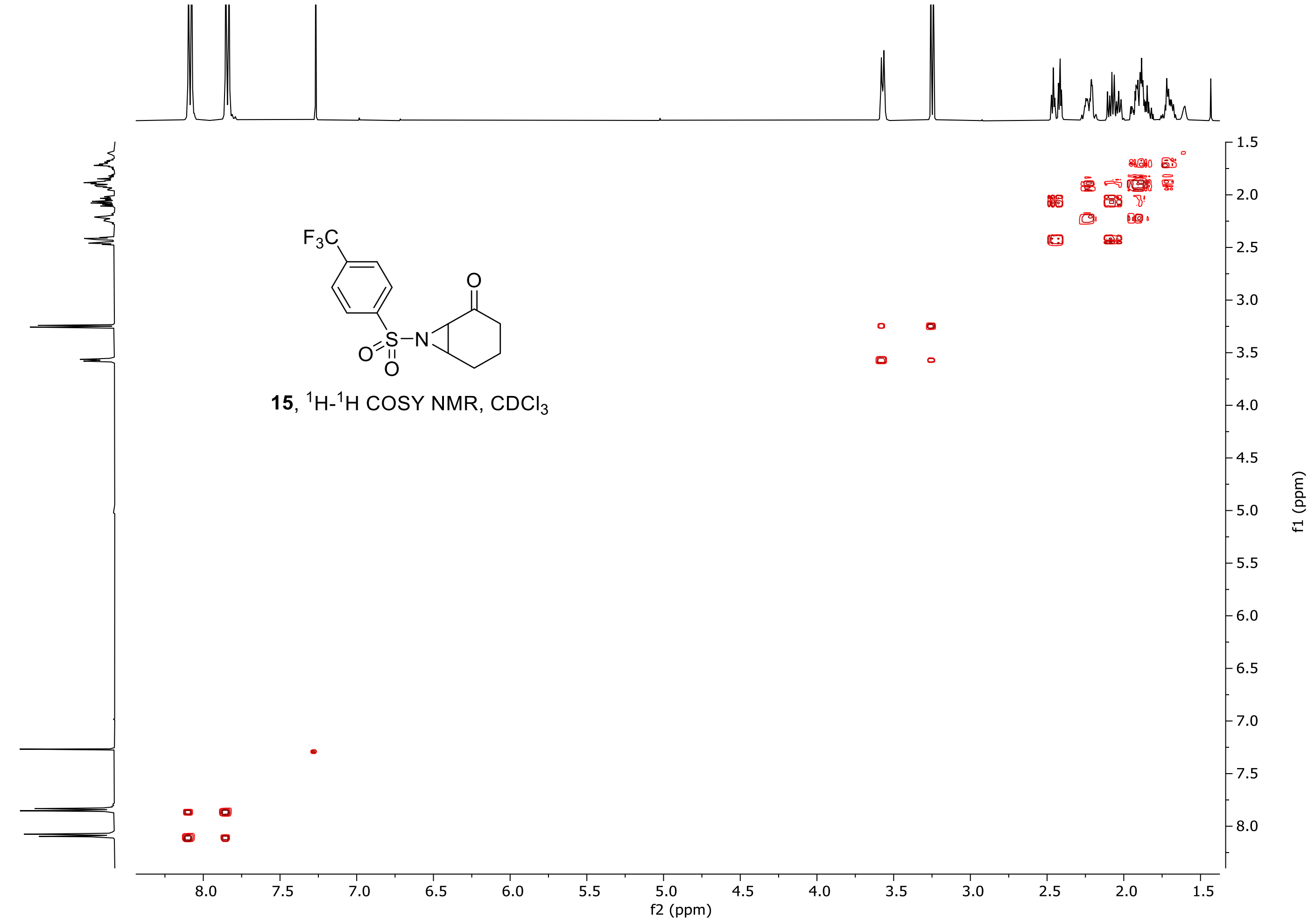
F3C
15, 1H-1H COSY NMR, CDCl3
f2 (ppm)
f1 (ppm)

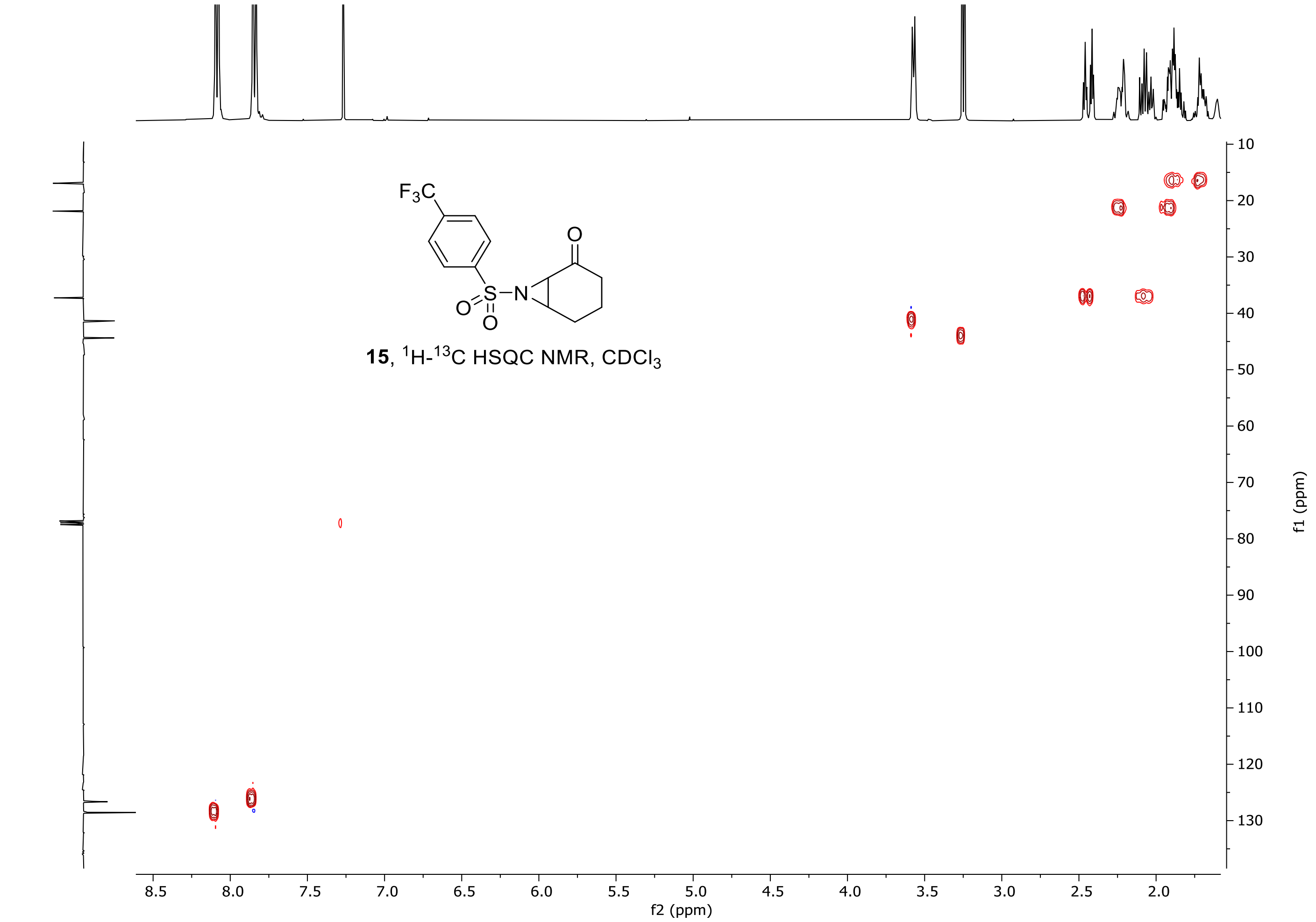

15, ^{1}H-^{13}C HSQC NMR, $CDCl_3$

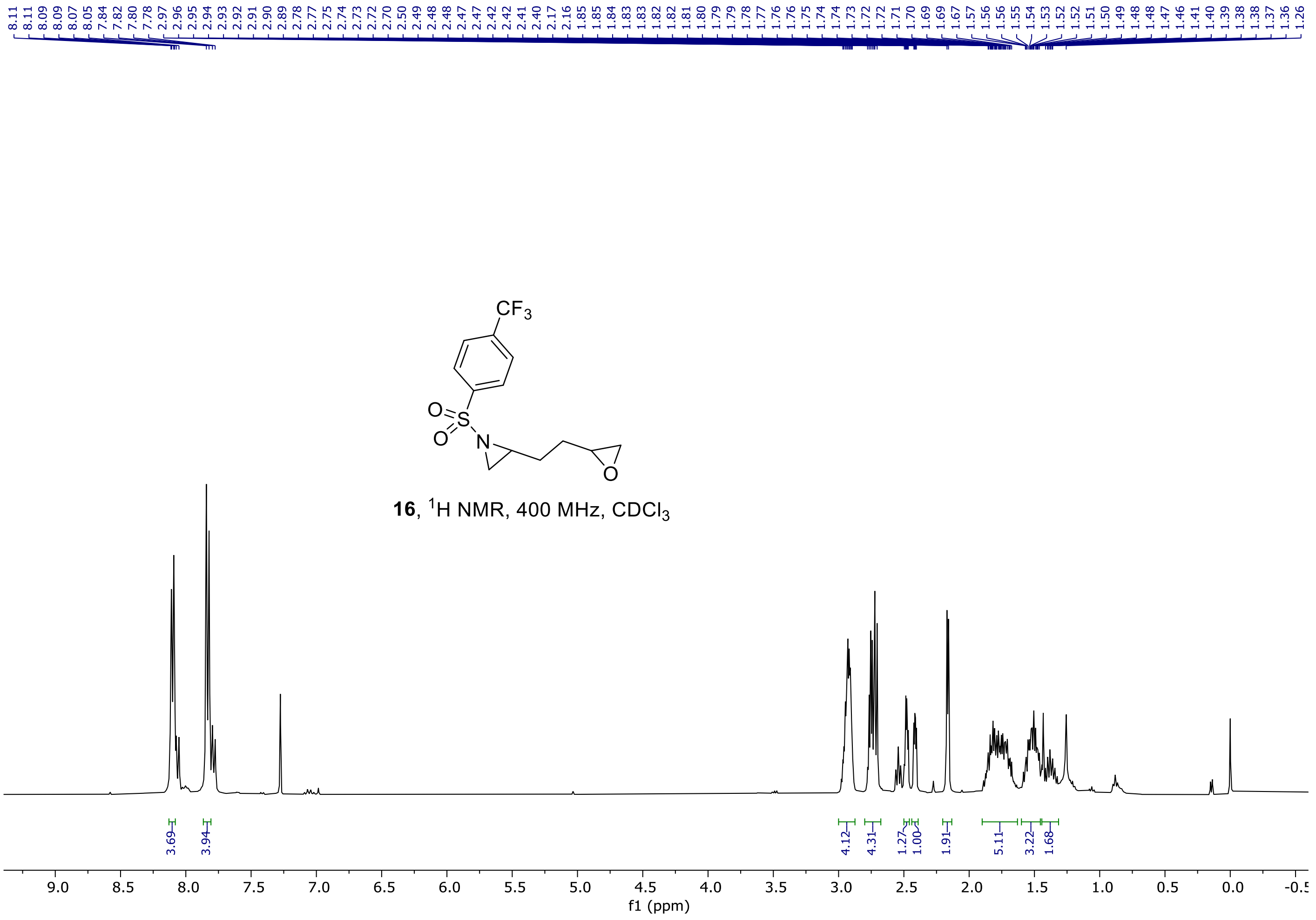

16, ^{1}H NMR, 400 MHz, $CDCl_3$

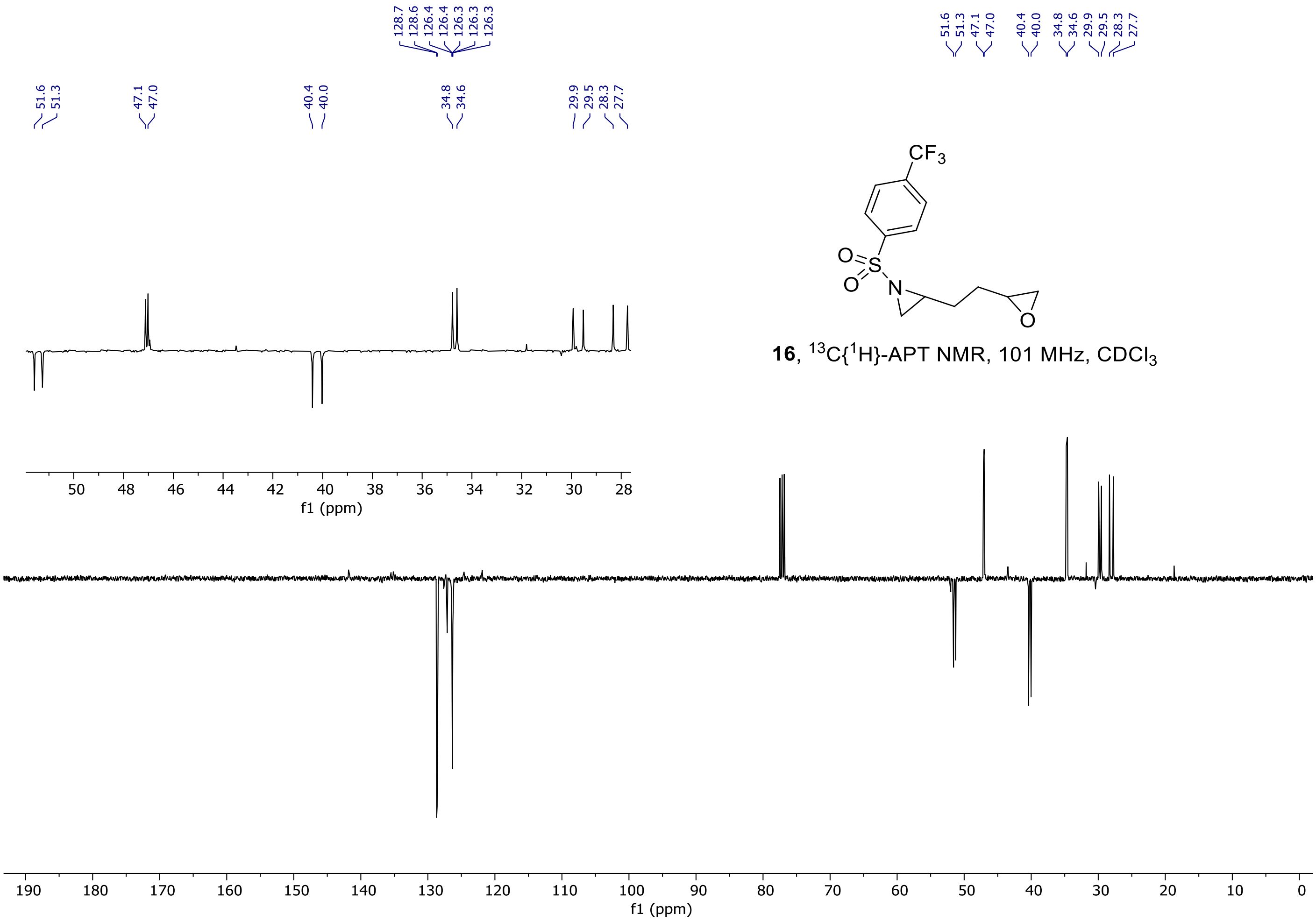

16, $^{13}C\{^{1}H\}$-APT NMR, 101 MHz, $CDCl_3$

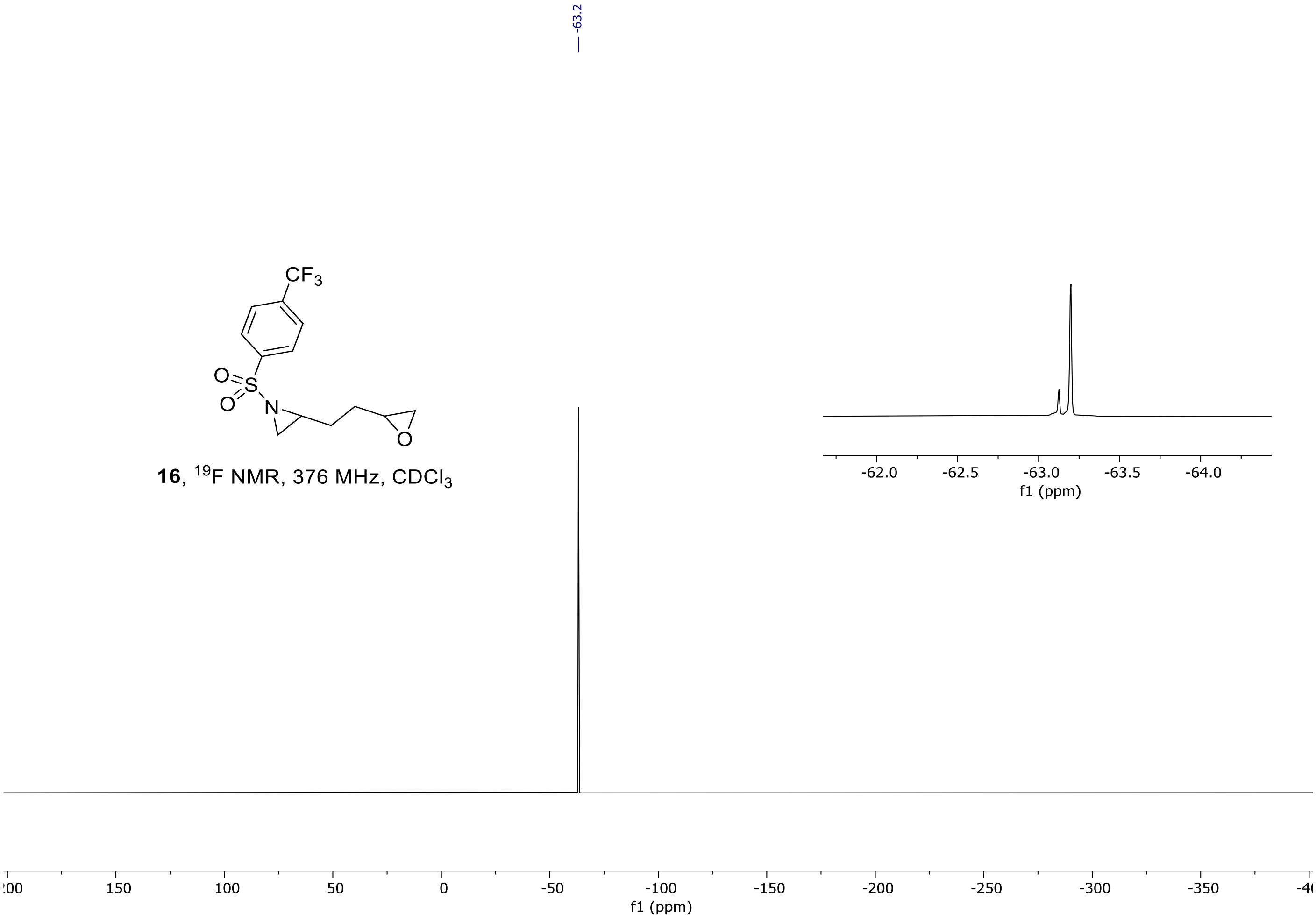

16, ^{19}F NMR, 376 MHz, $CDCl_3$

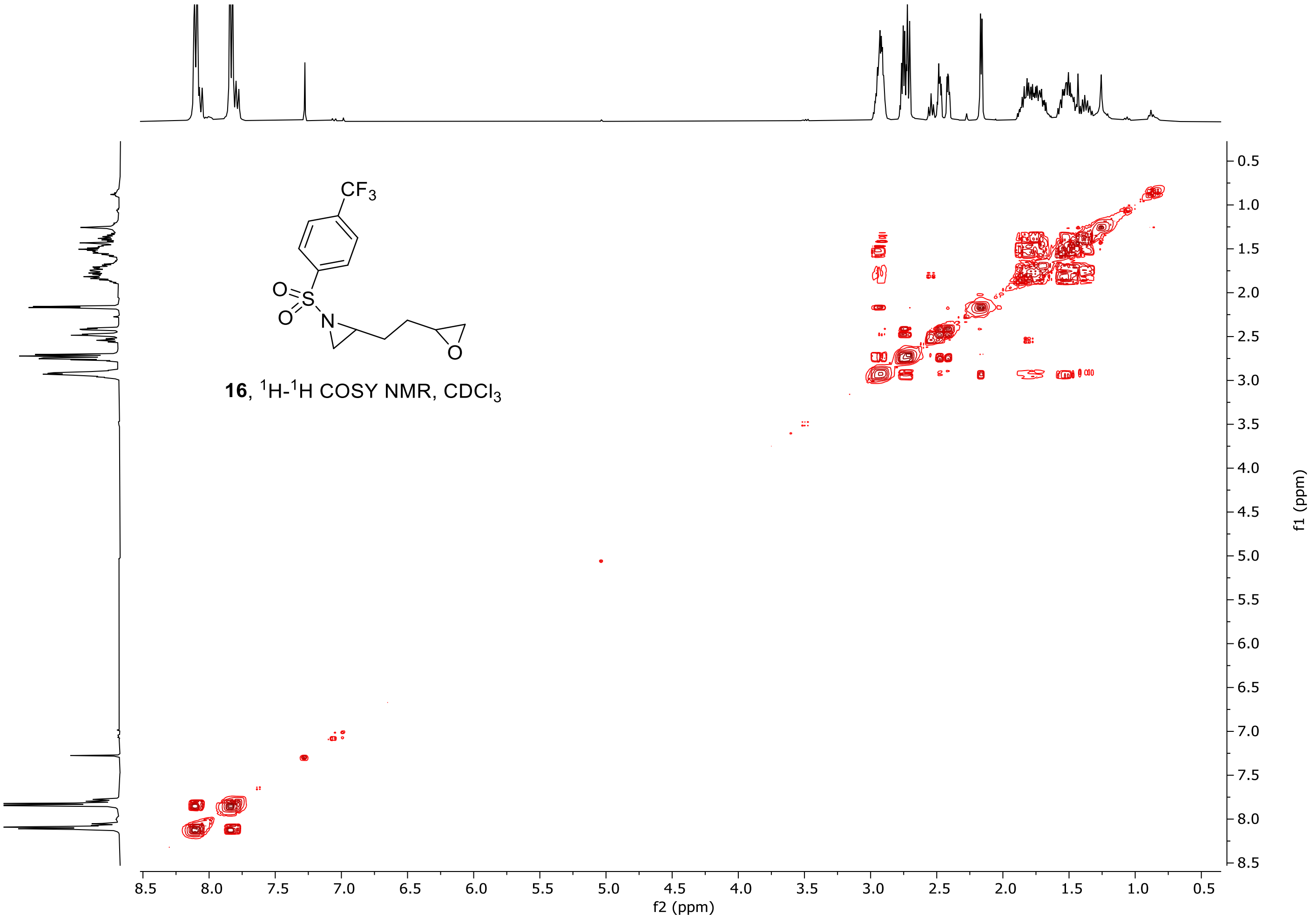

16, ^{1}H-^{1}H COSY NMR, $CDCl_3$

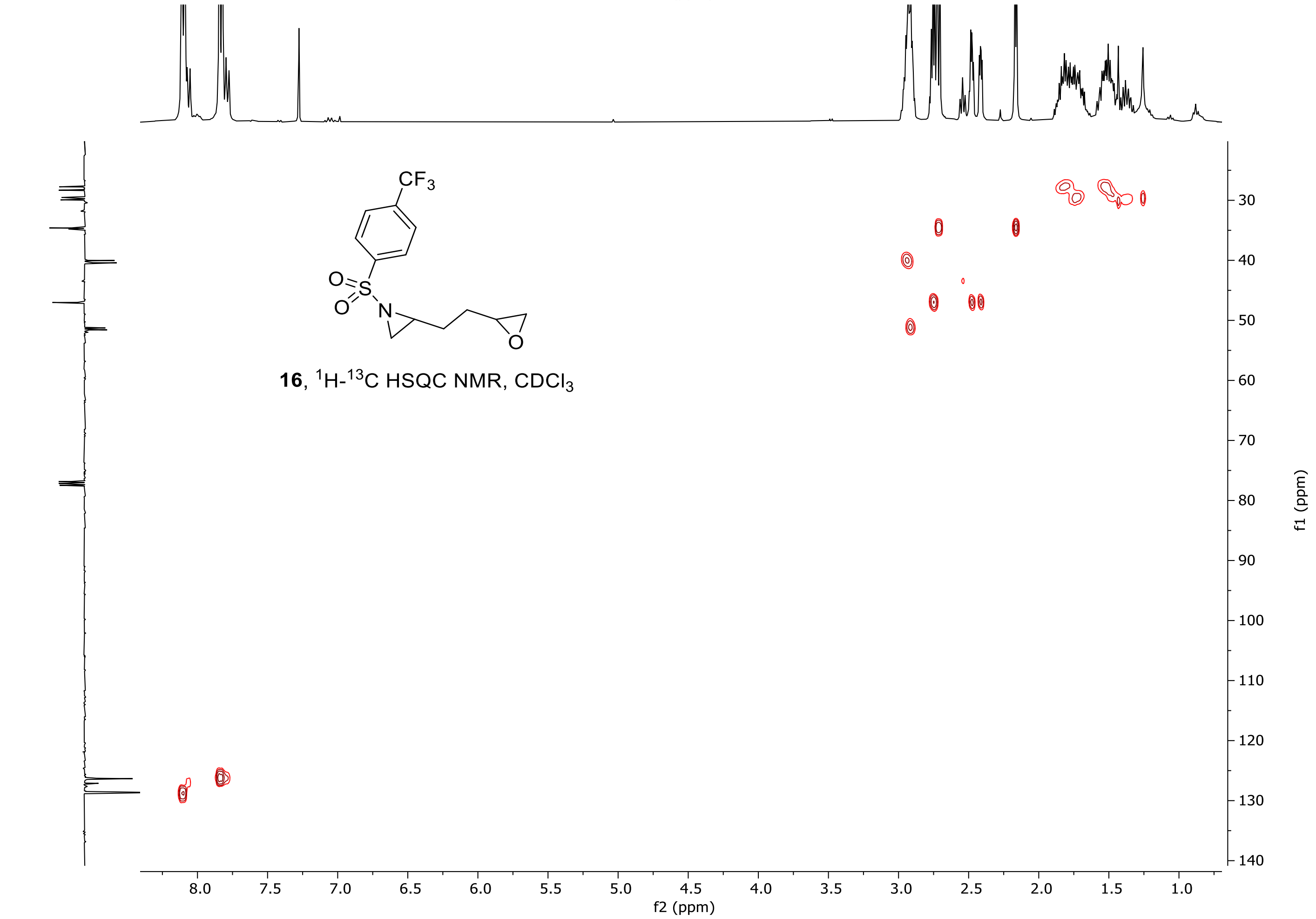

16, ^{1}H-^{13}C HSQC NMR, $CDCl_3$

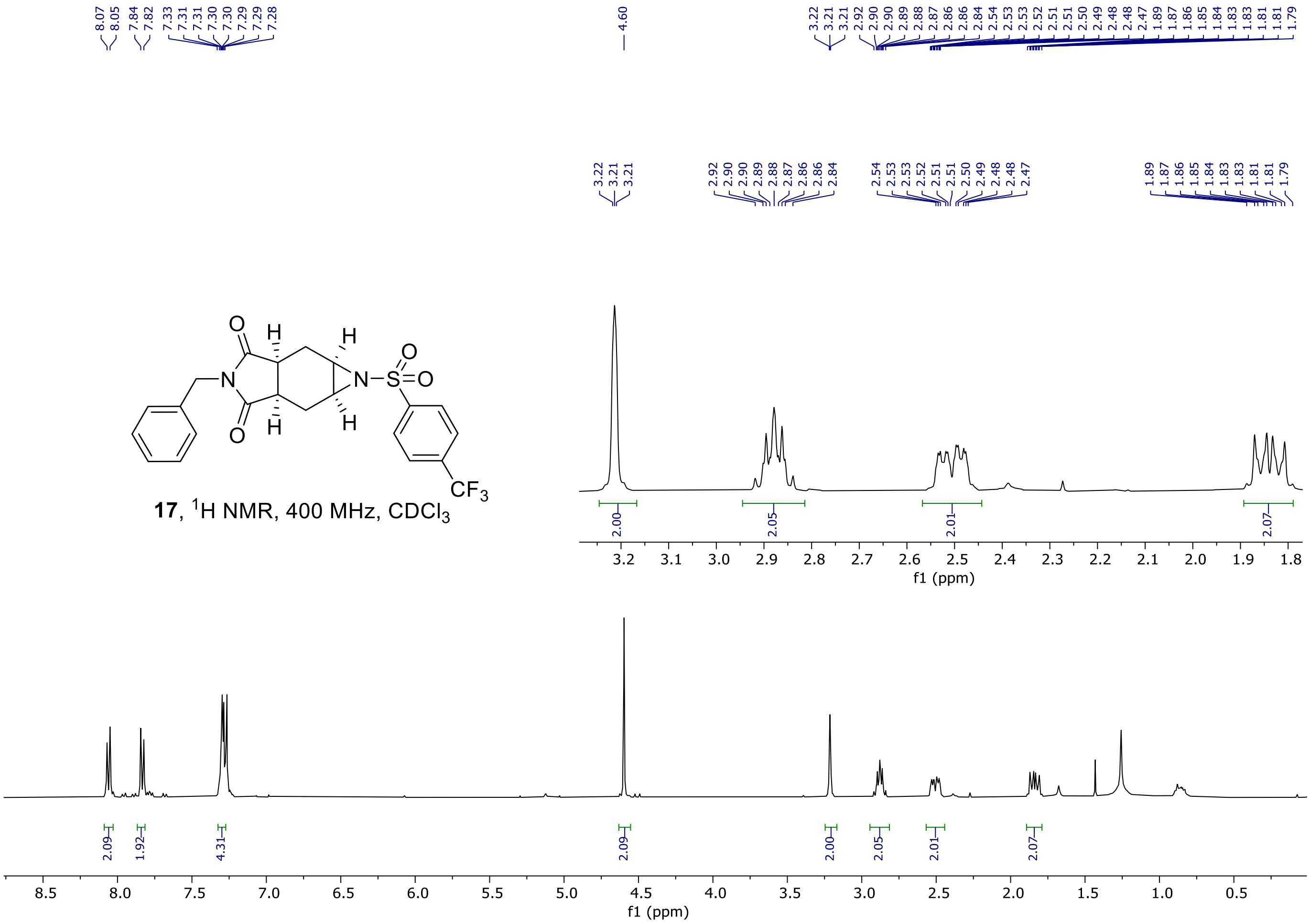

17, ^{1}H NMR, 400 MHz, $CDCl_3$

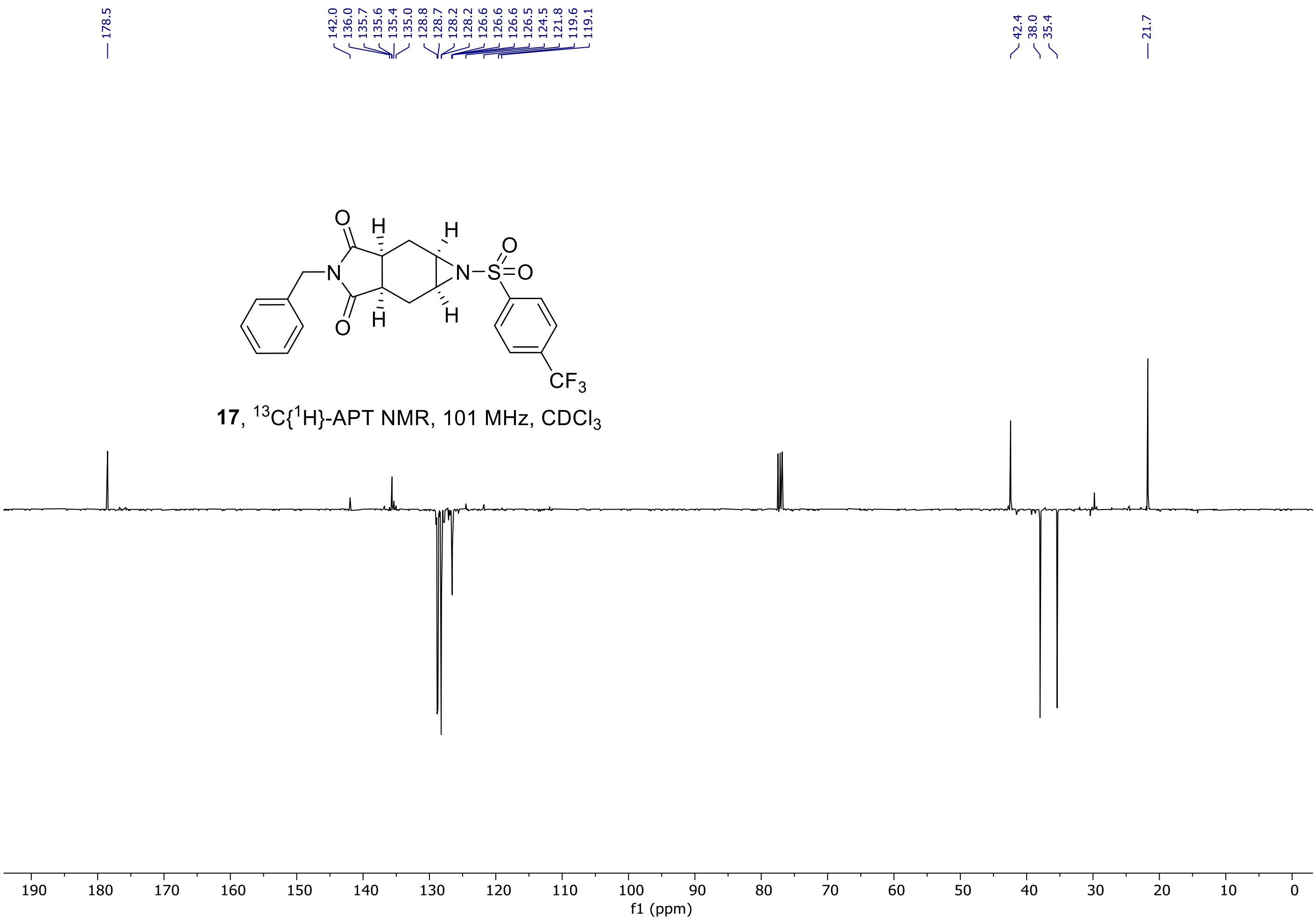

17, $^{13}C\{^{1}H\}$-APT NMR, 101 MHz, $CDCl_3$

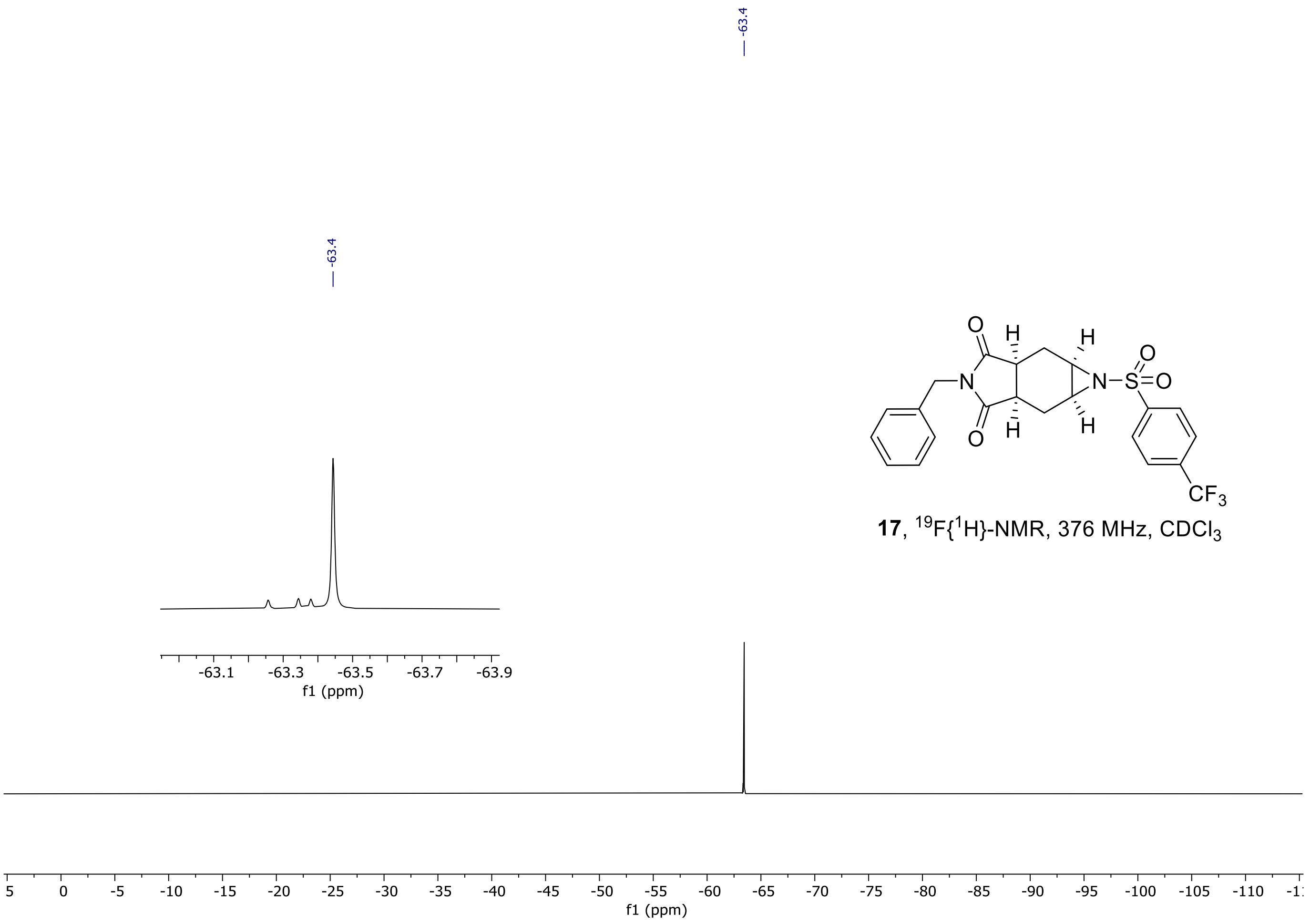

17, $^{19}F\{^{1}H\}$-NMR, 376 MHz, $CDCl_3$

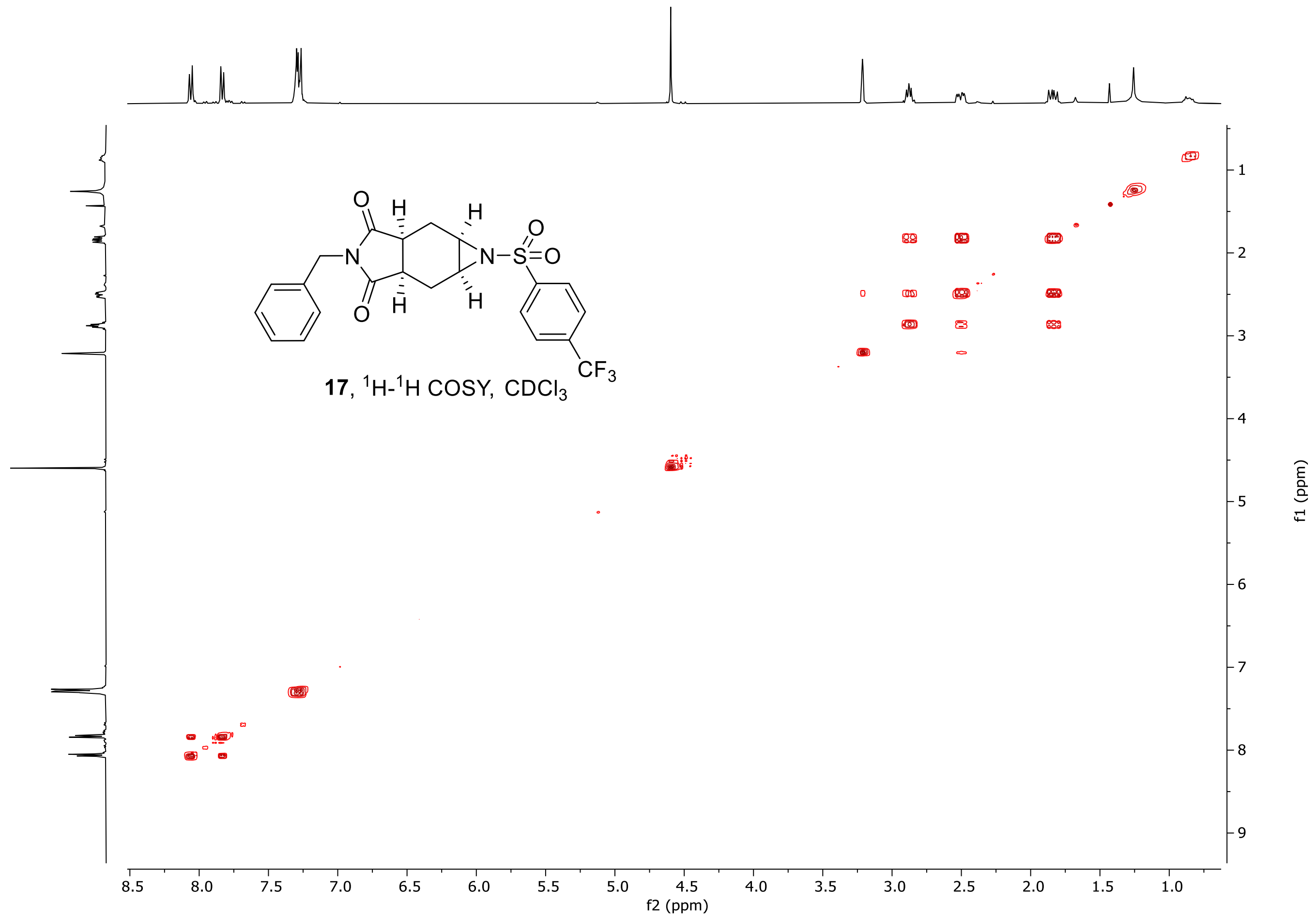

17, ^{1}H-^{1}H COSY, $CDCl_3$

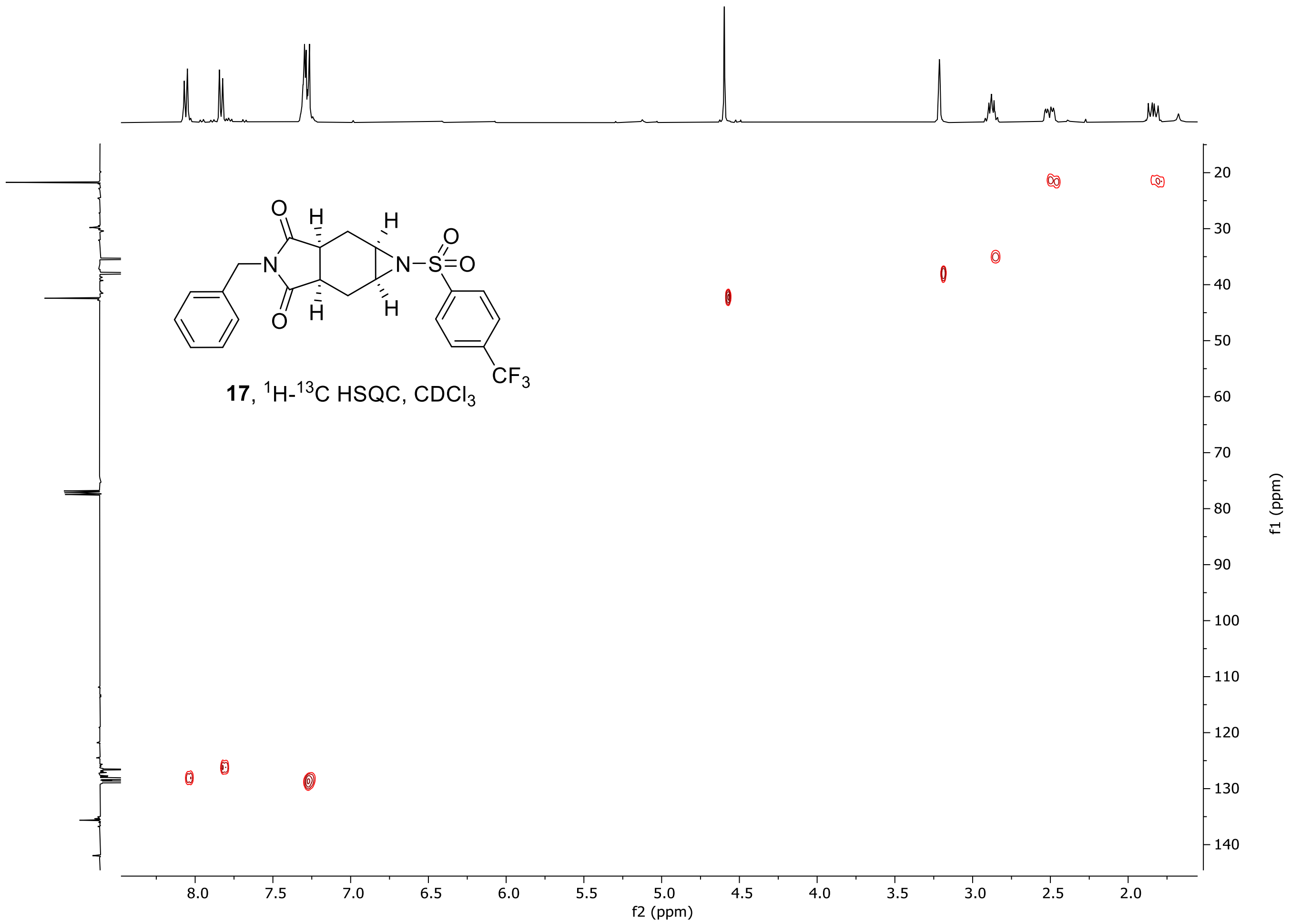
17, ^{1}H-^{13}C HSQC, CDCl$_3$
f2 (ppm)
f1 (ppm)

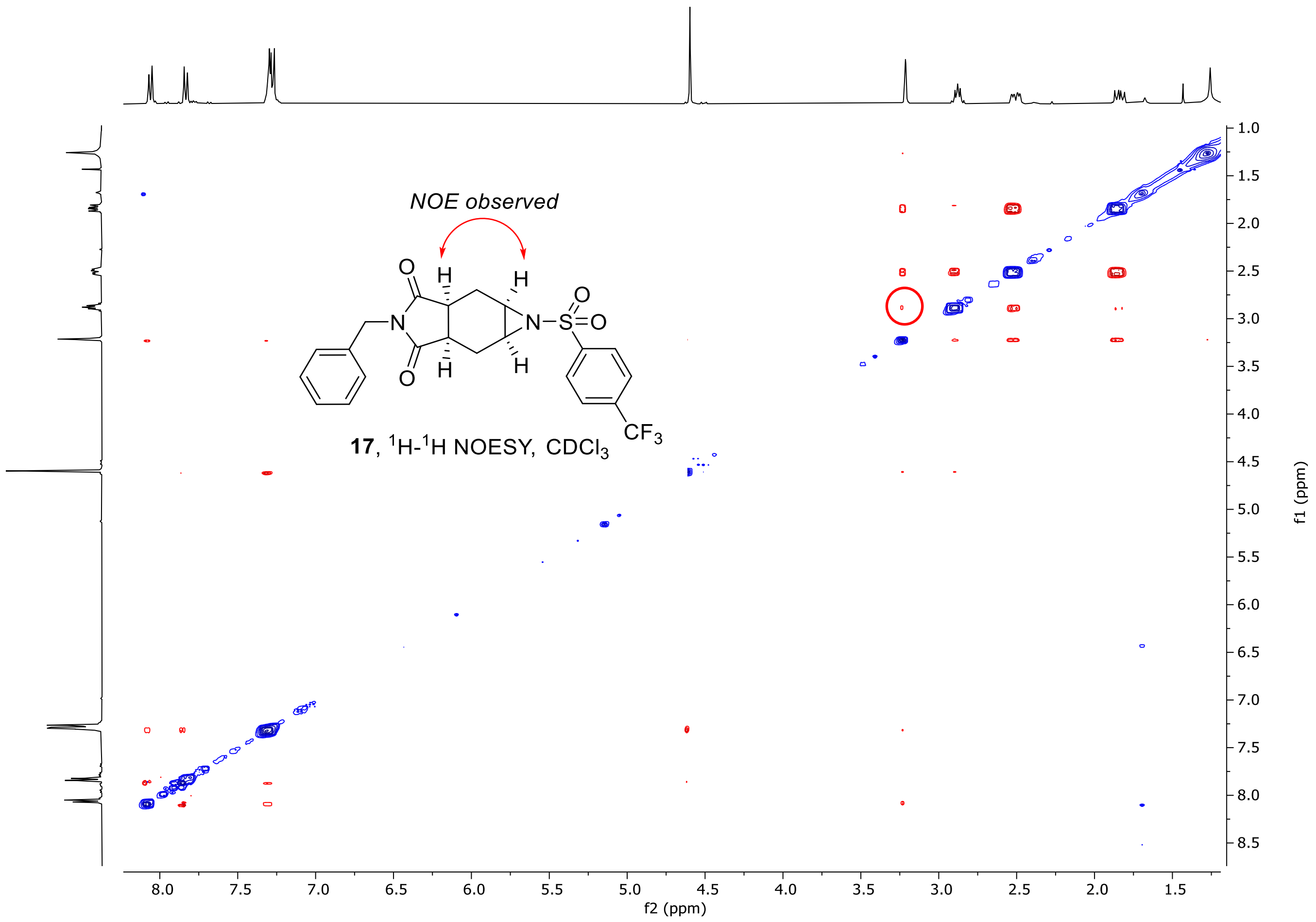
NOE observed
17, ^{1}H-^{1}H NOESY, CDCl$_3$
f2 (ppm)
f1 (ppm)

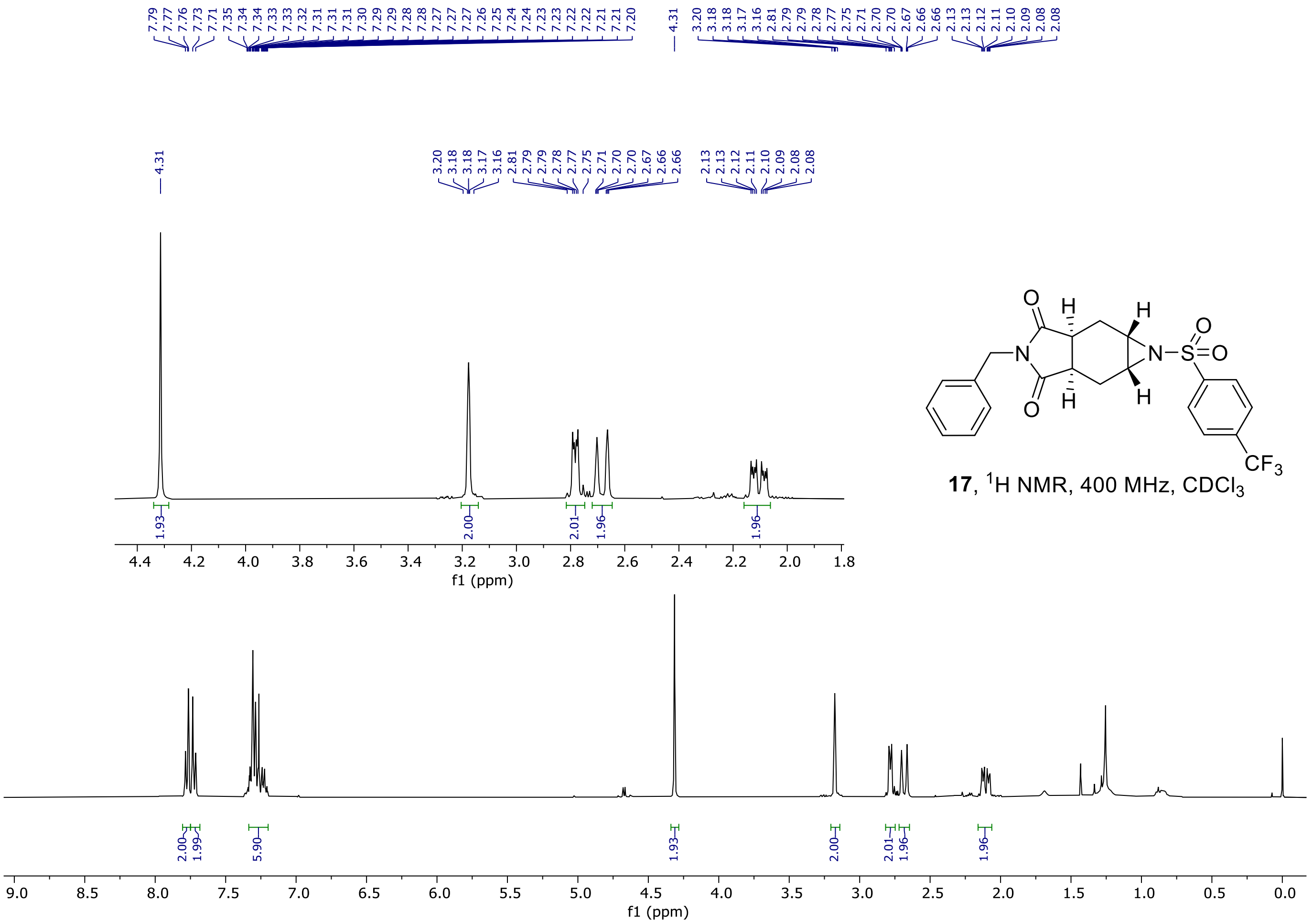

17, ^{1}H NMR, 400 MHz, $CDCl_3$

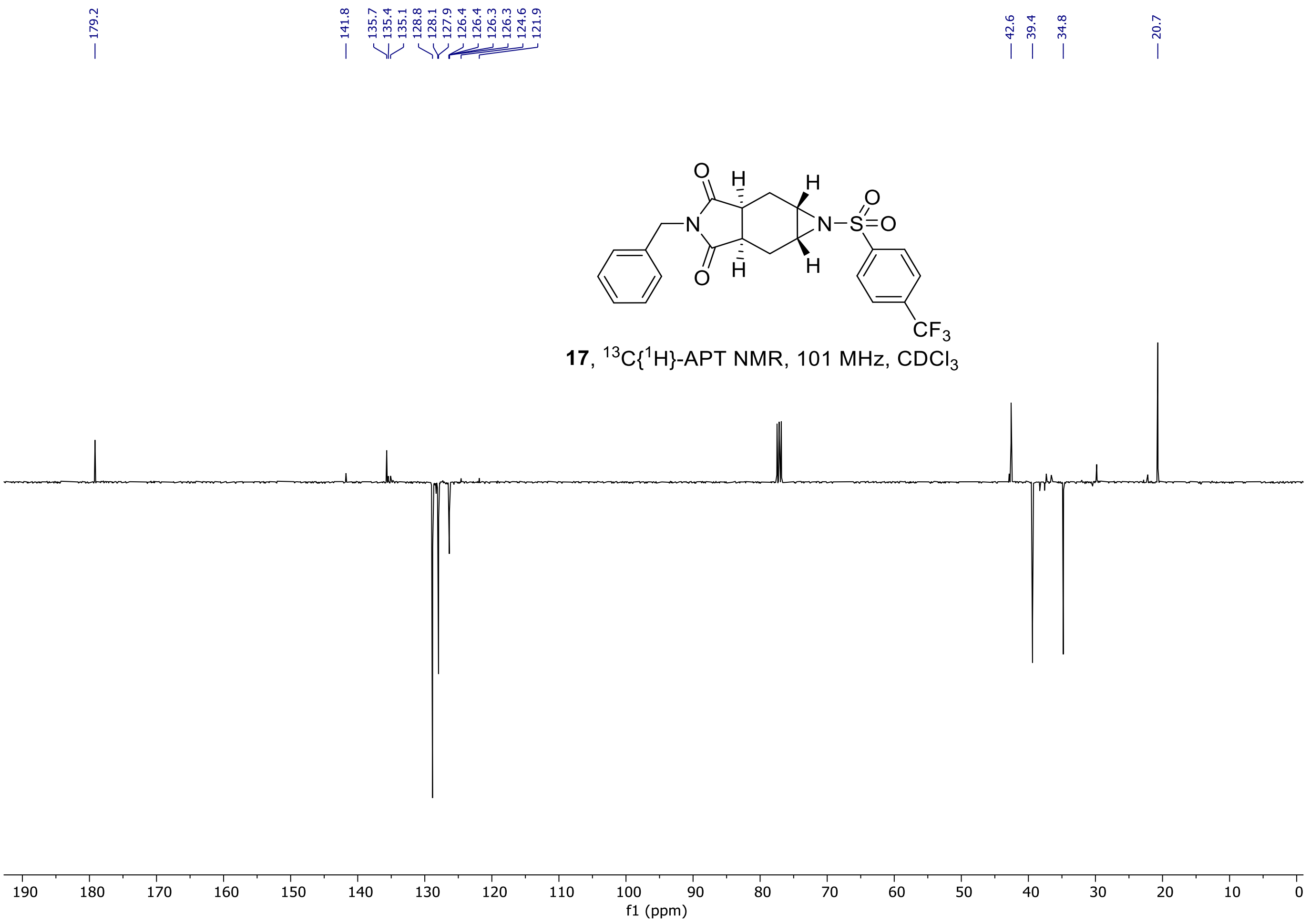

17, ^{13}C{^{1}H}-APT NMR, 101 MHz, $CDCl_3$

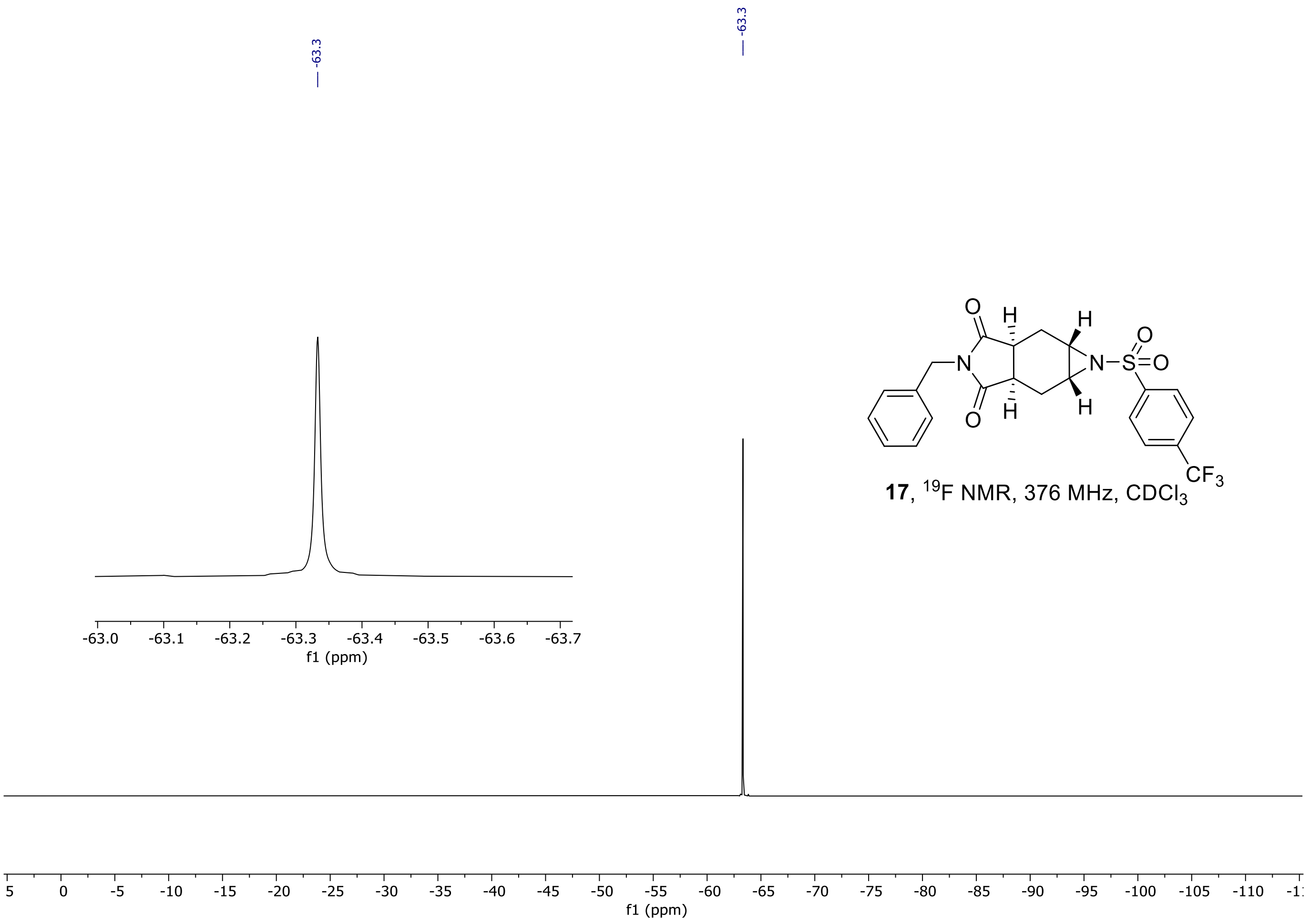

17, ^{19}F NMR, 376 MHz, $CDCl_3$

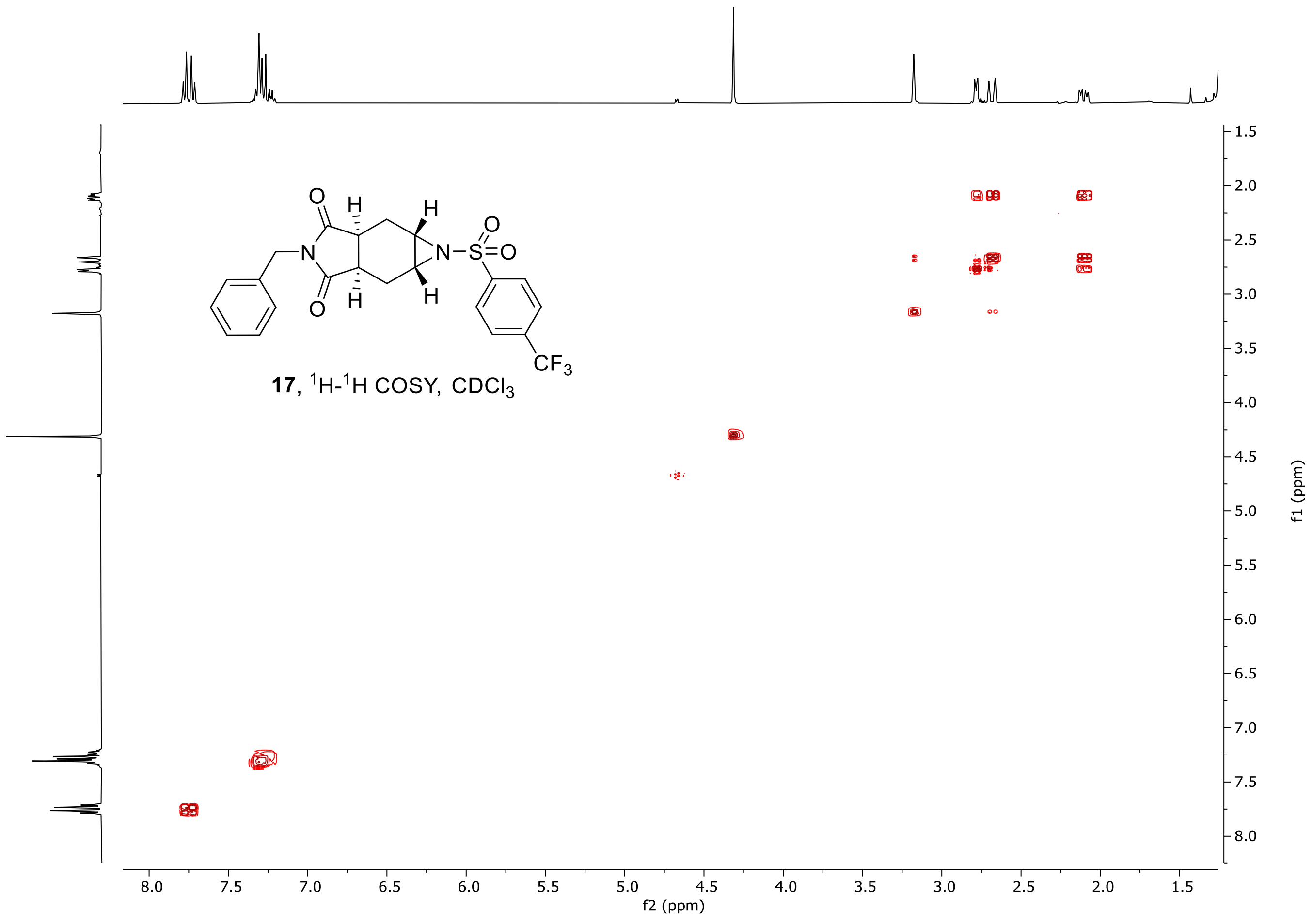

17, ^{1}H-^{1}H COSY, $CDCl_3$

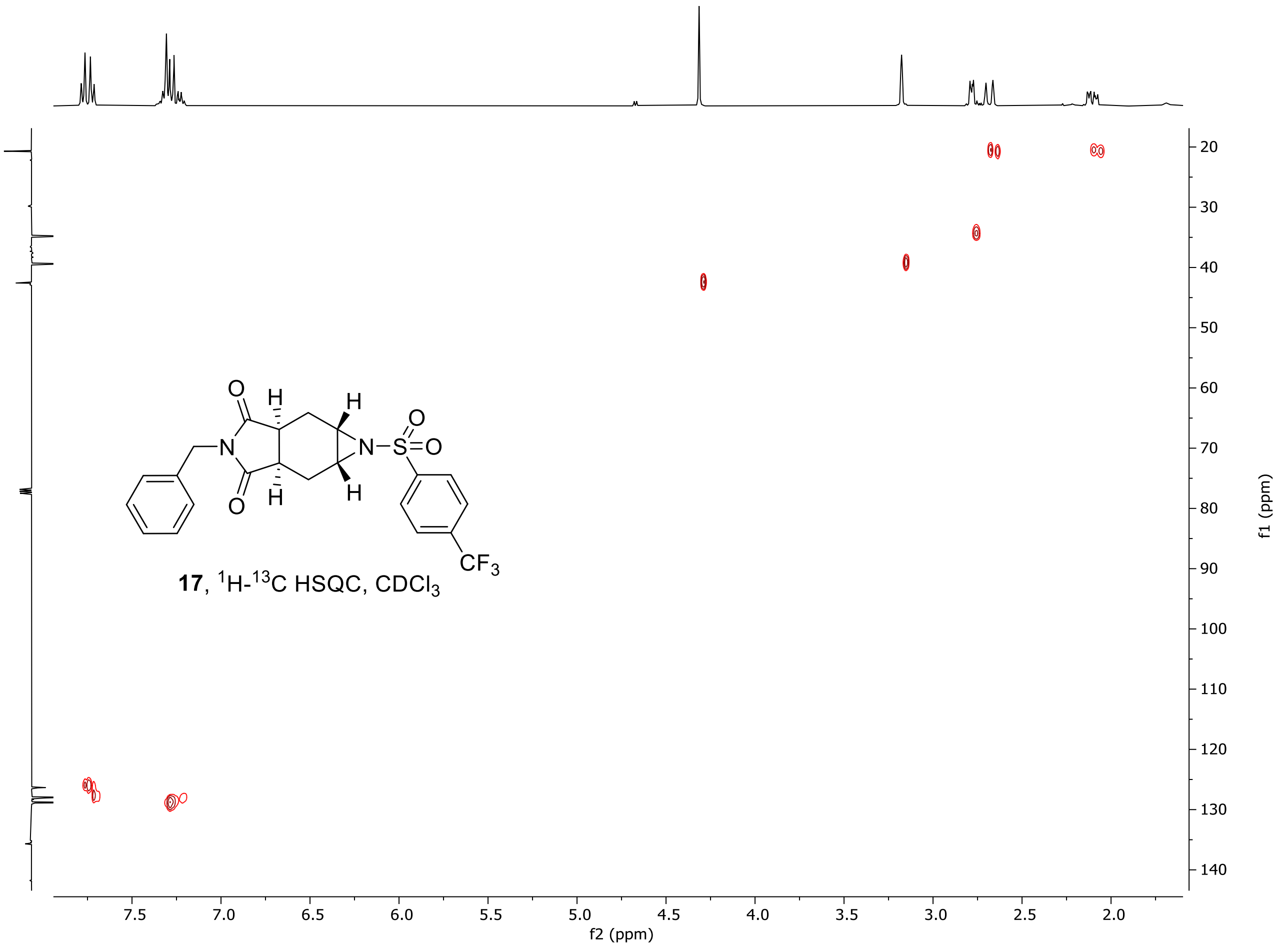
17, 1H-13C HSQC, CDCl3
CF3
f2 (ppm)
f1 (ppm)

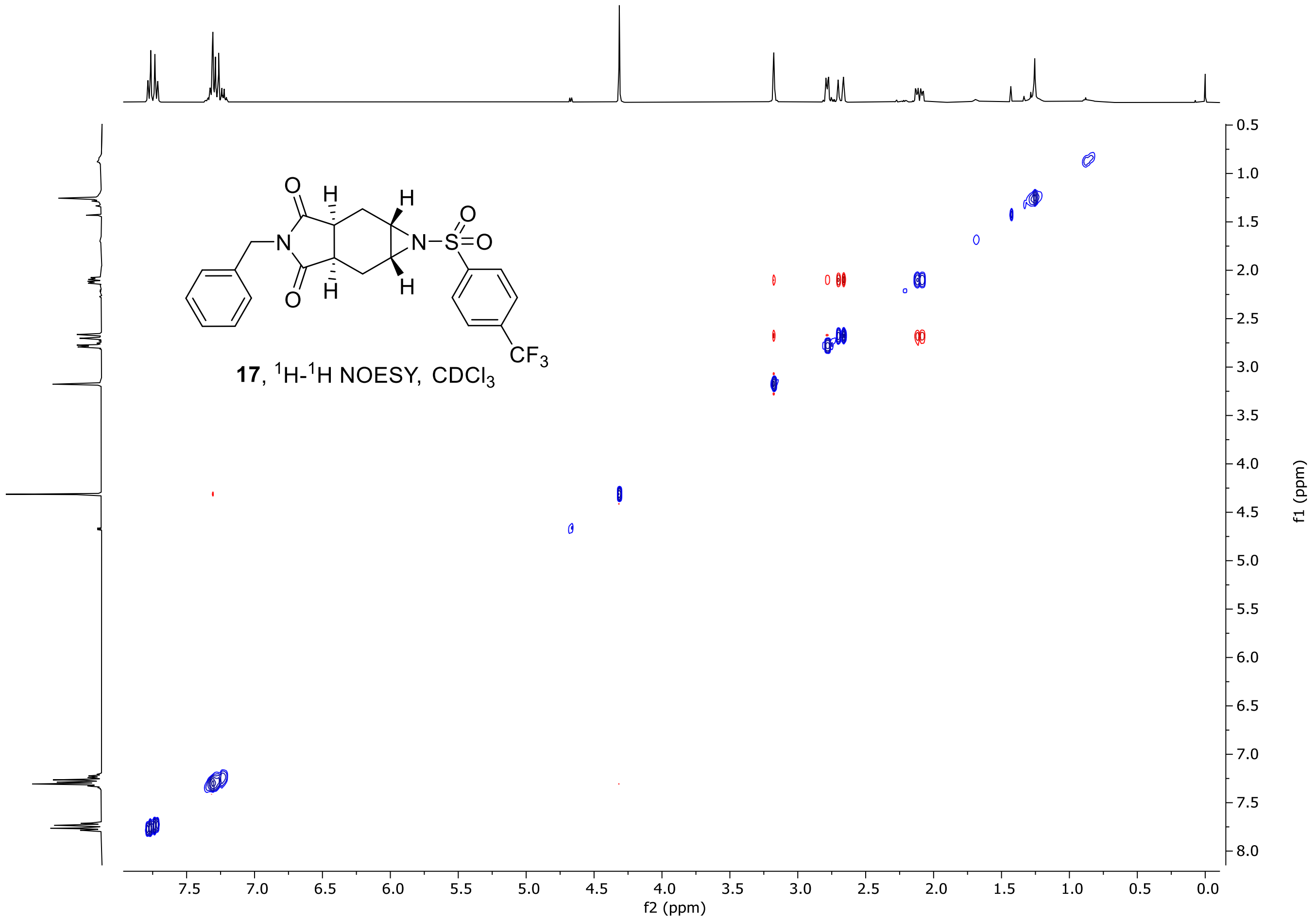
17, 1H-1H NOESY, CDCl3
CF3
f2 (ppm)
f1 (ppm)

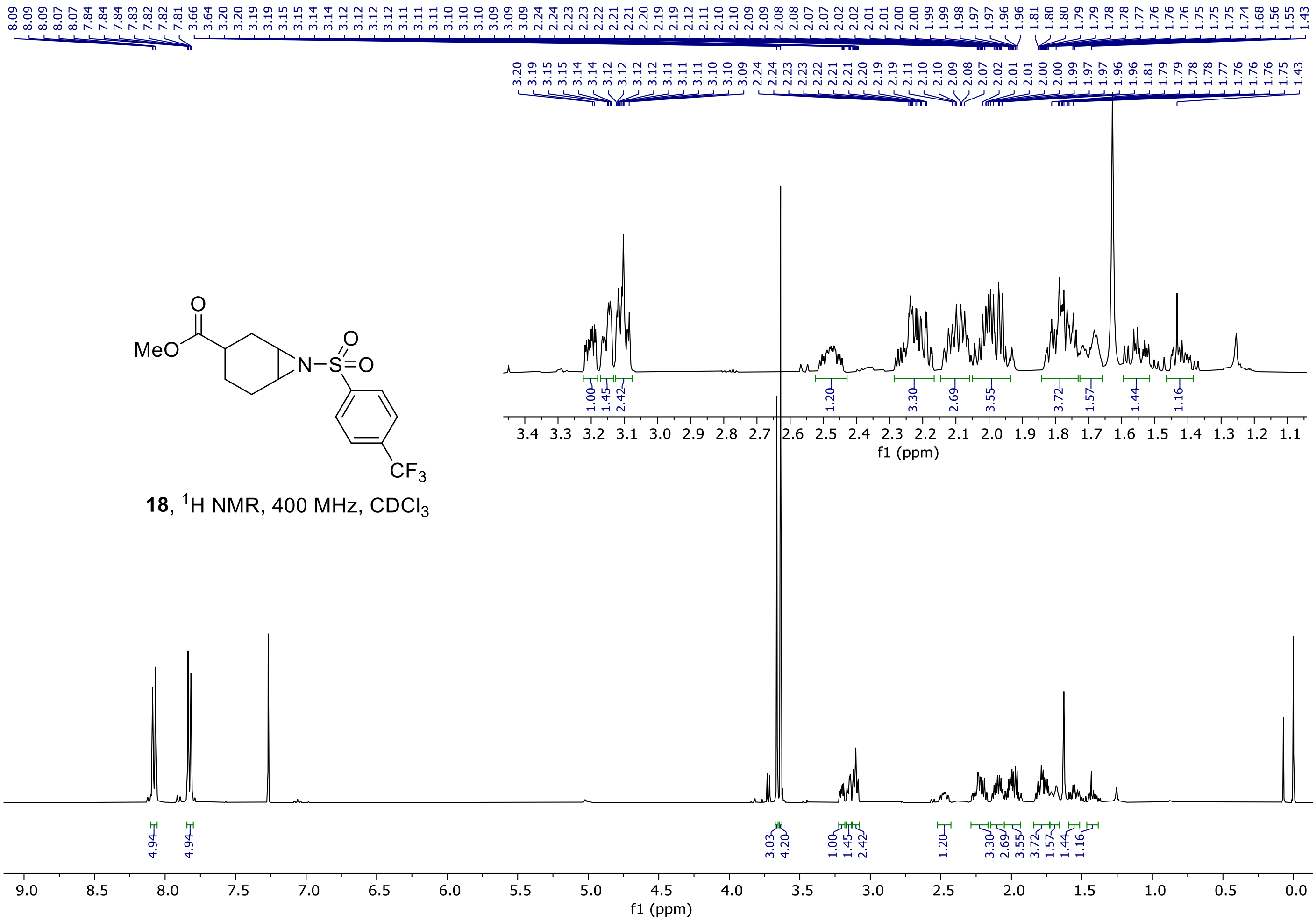

18, ^{1}H NMR, 400 MHz, $CDCl_3$

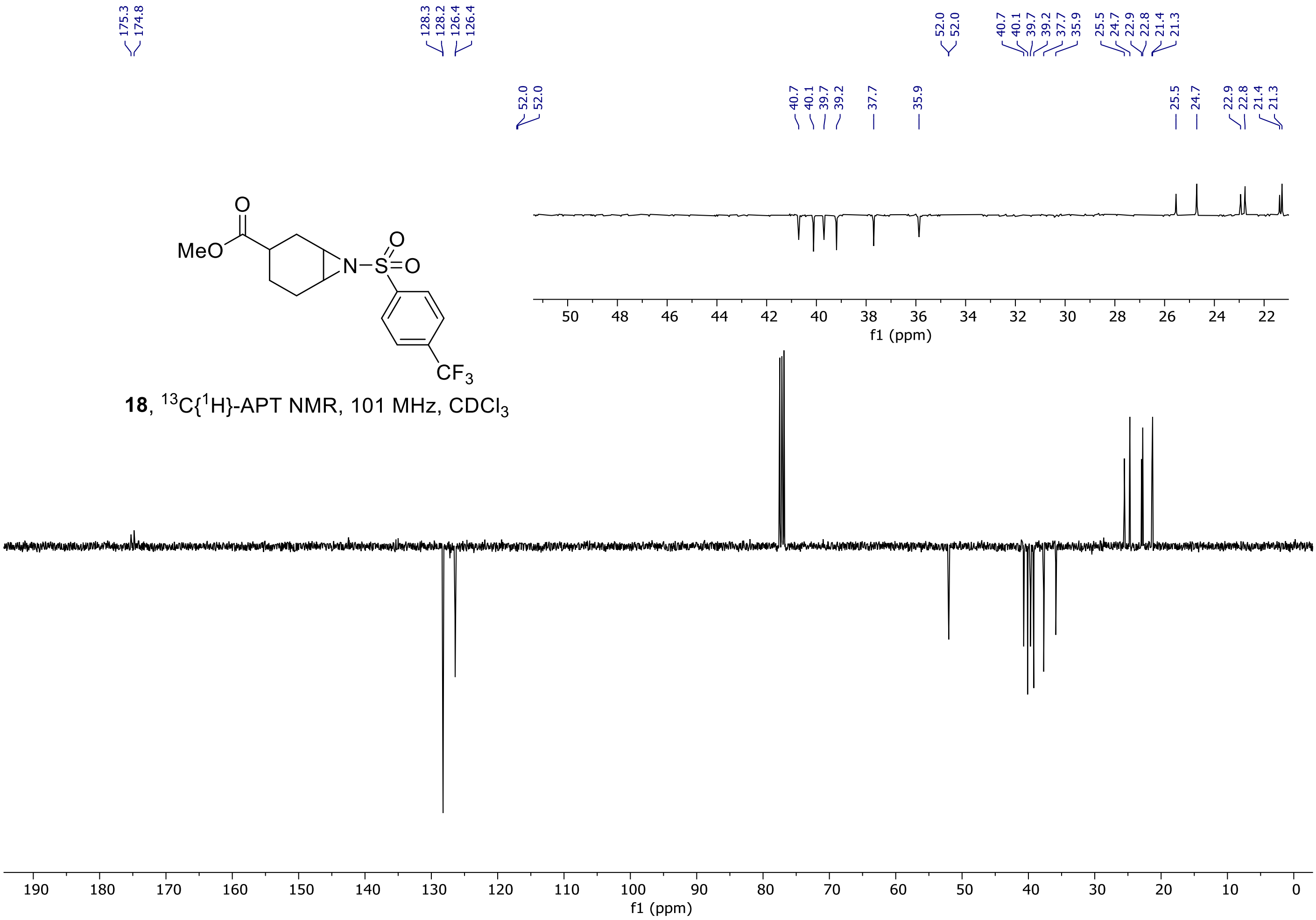

18, $^{13}C\{^{1}H\}$-APT NMR, 101 MHz, $CDCl_3$

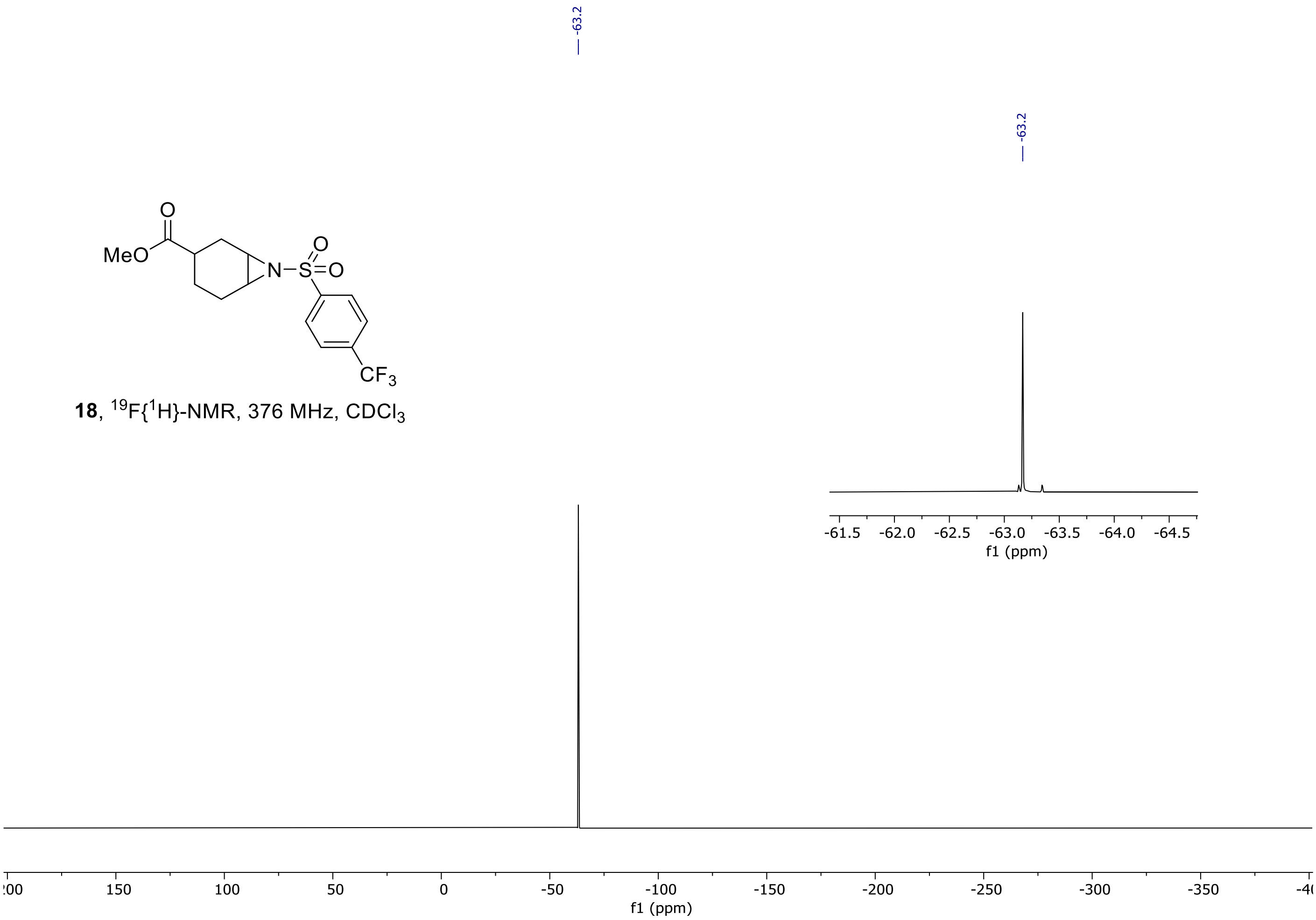

18, $^{19}F\{^1H\}$-NMR, 376 MHz, $CDCl_3$

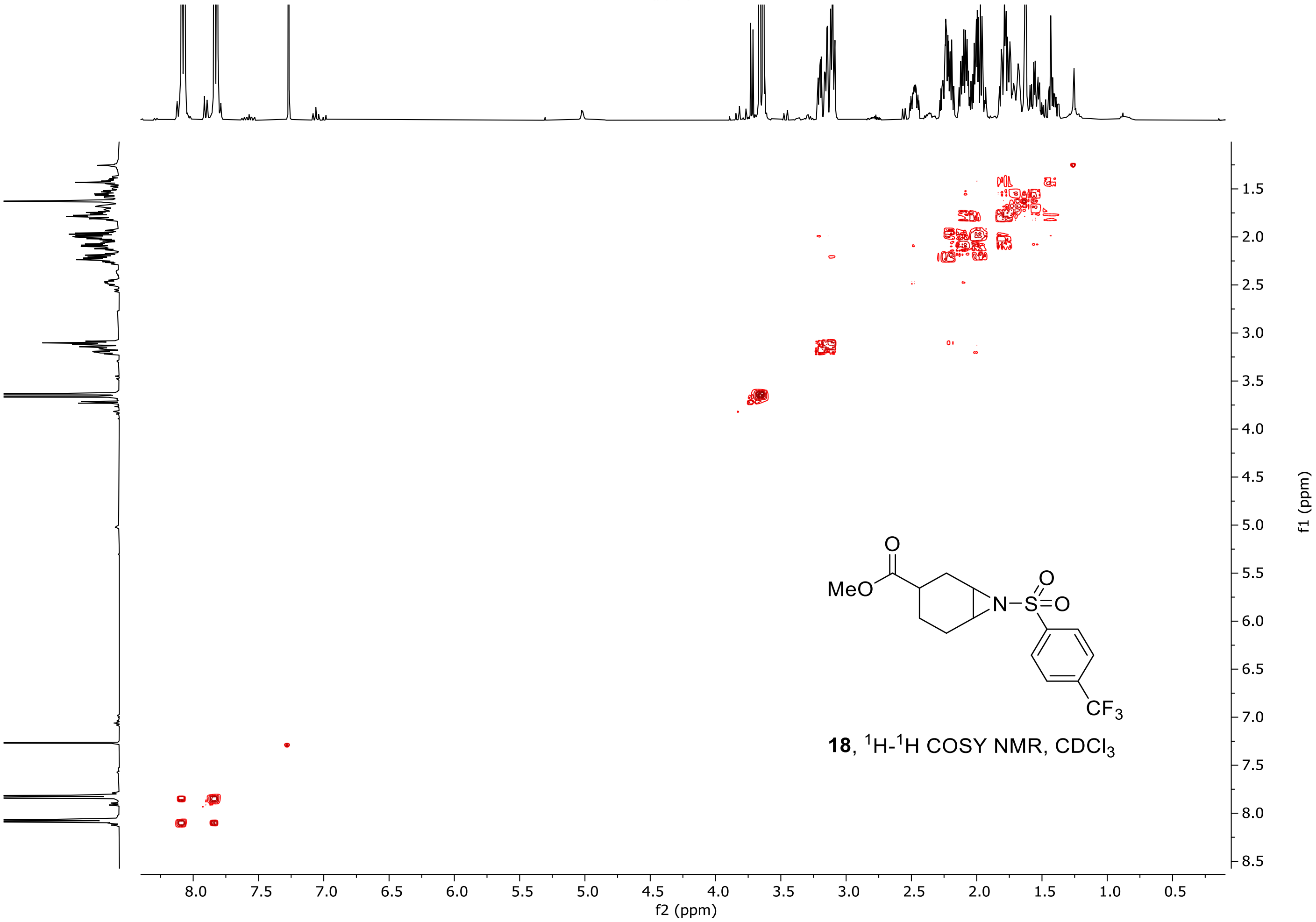

18, ^1H-^1H COSY NMR, $CDCl_3$

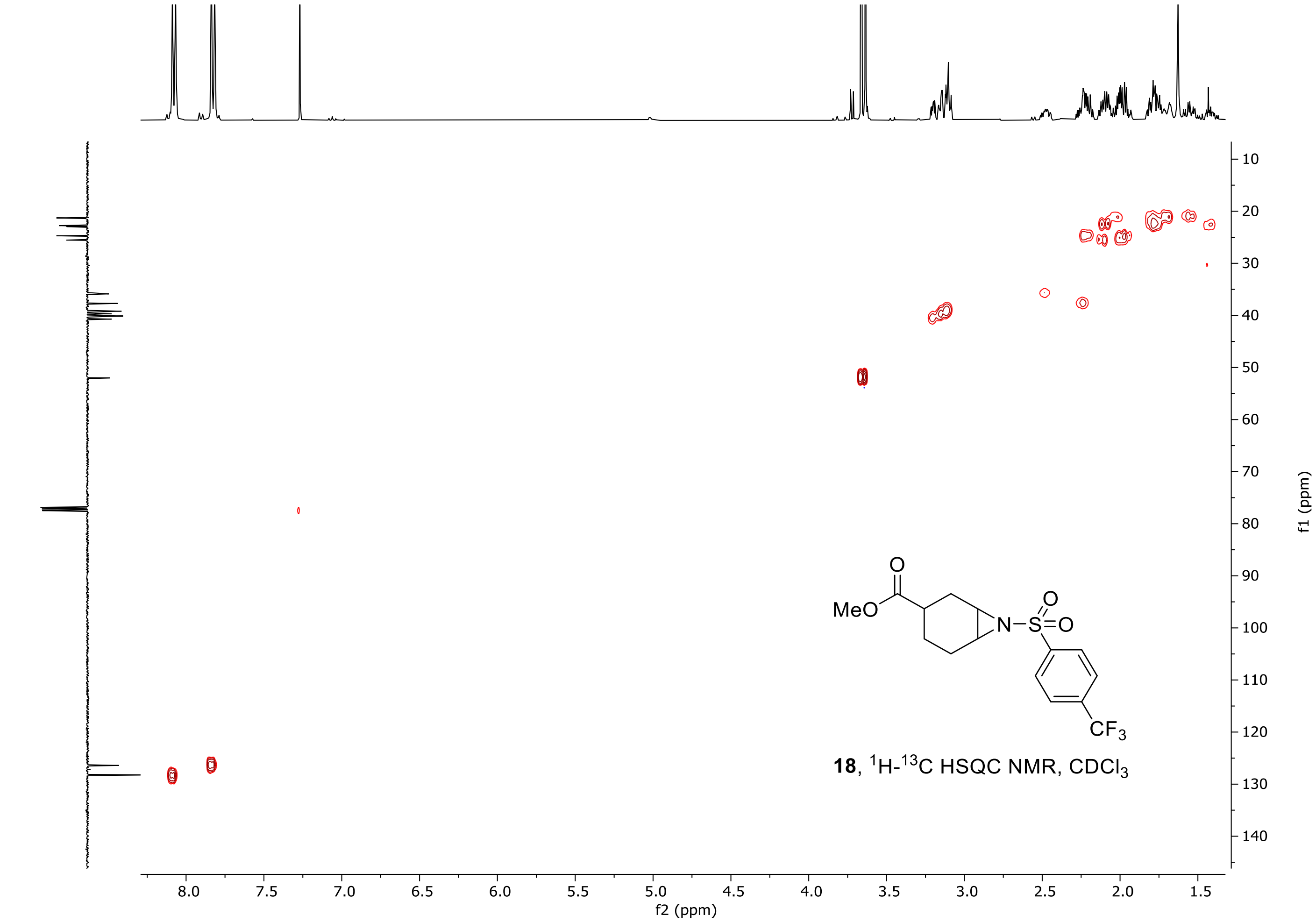
MeO
O
N
S
O
O
CF3
18, 1H-13C HSQC NMR, CDCl3
f2 (ppm)
f1 (ppm)

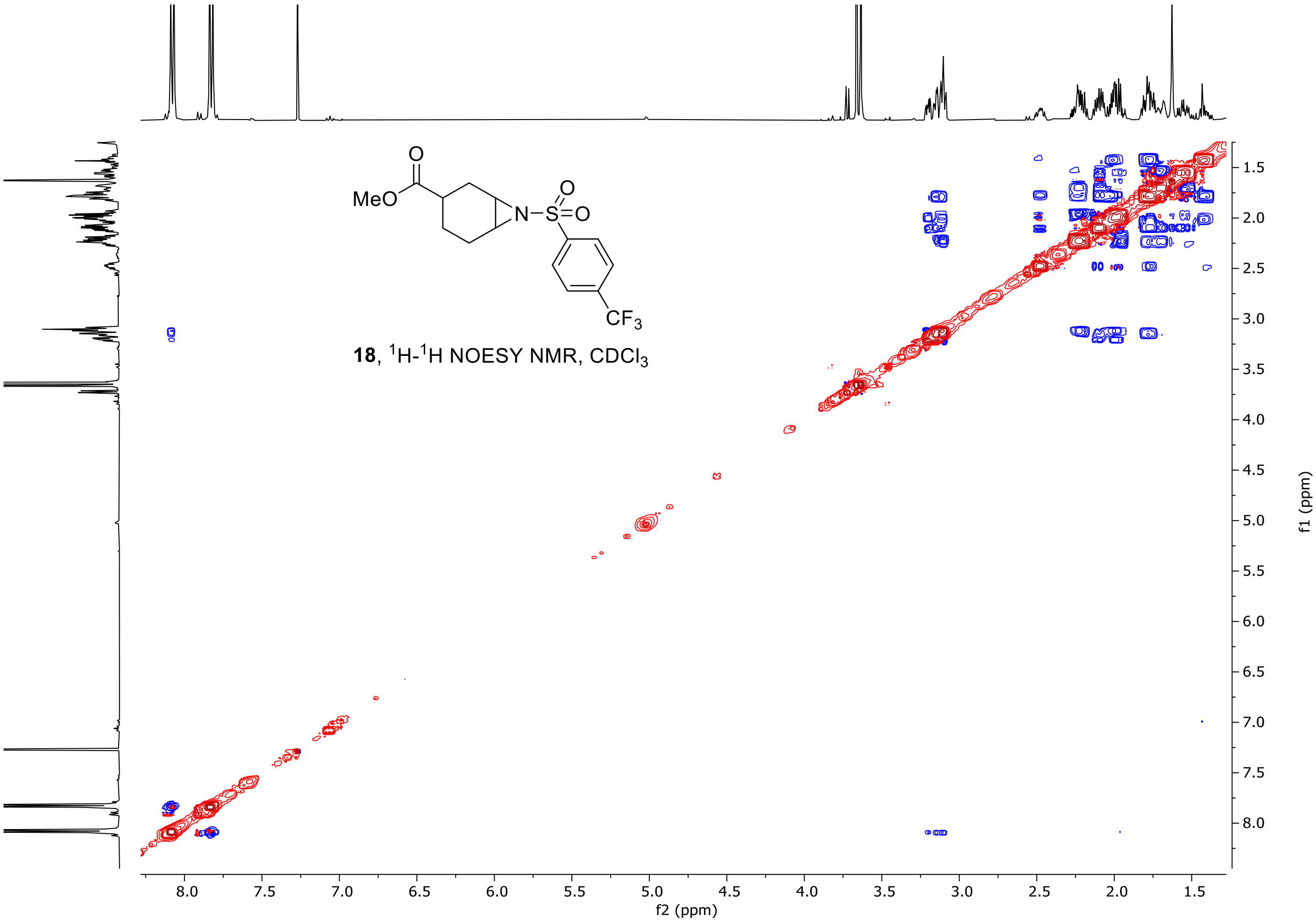
MeO
O
N
S
O
O
CF3
18, 1H-1H NOESY NMR, CDCl3
f2 (ppm)
f1 (ppm)

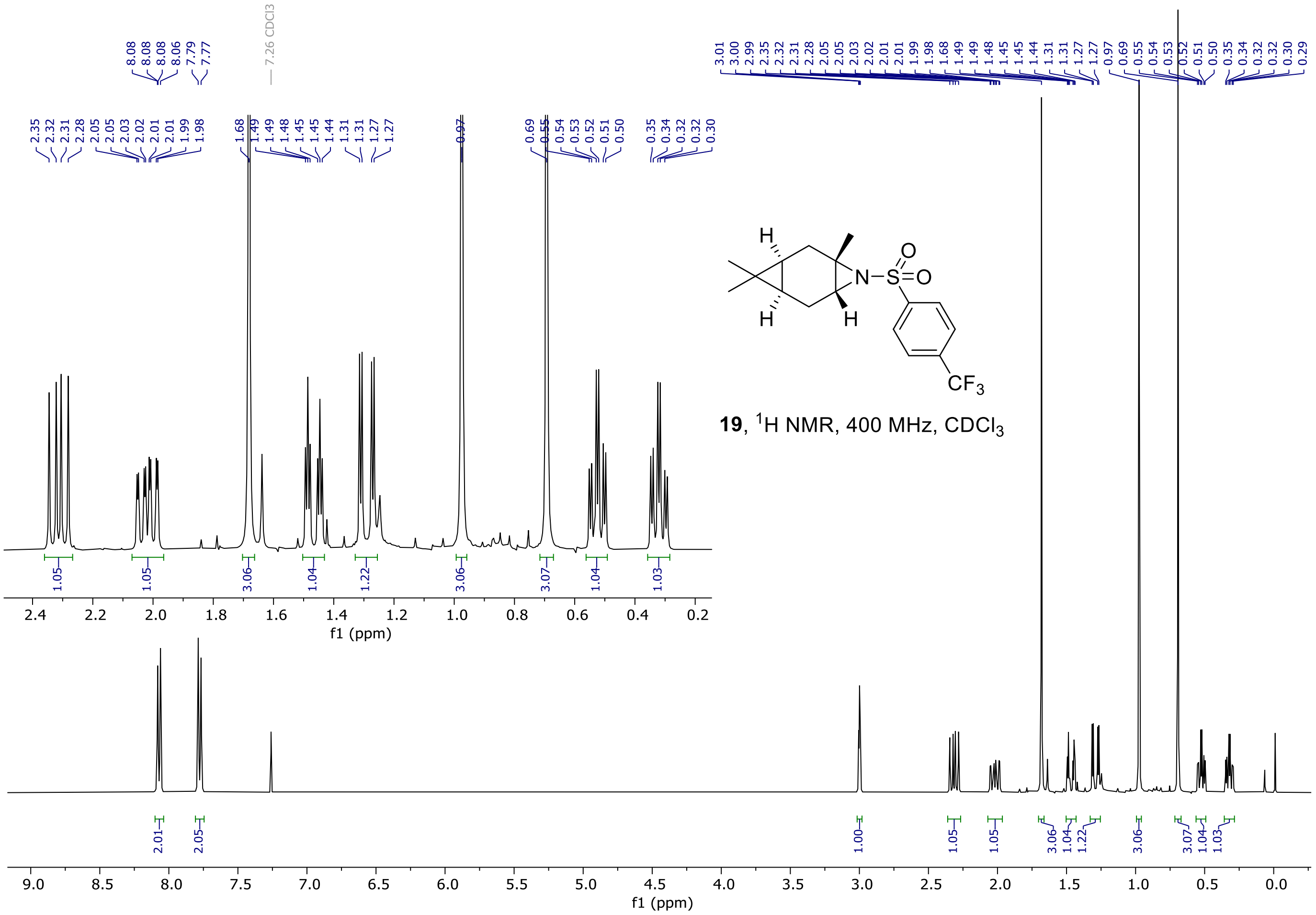

19, ^{1}H NMR, 400 MHz, $CDCl_3$

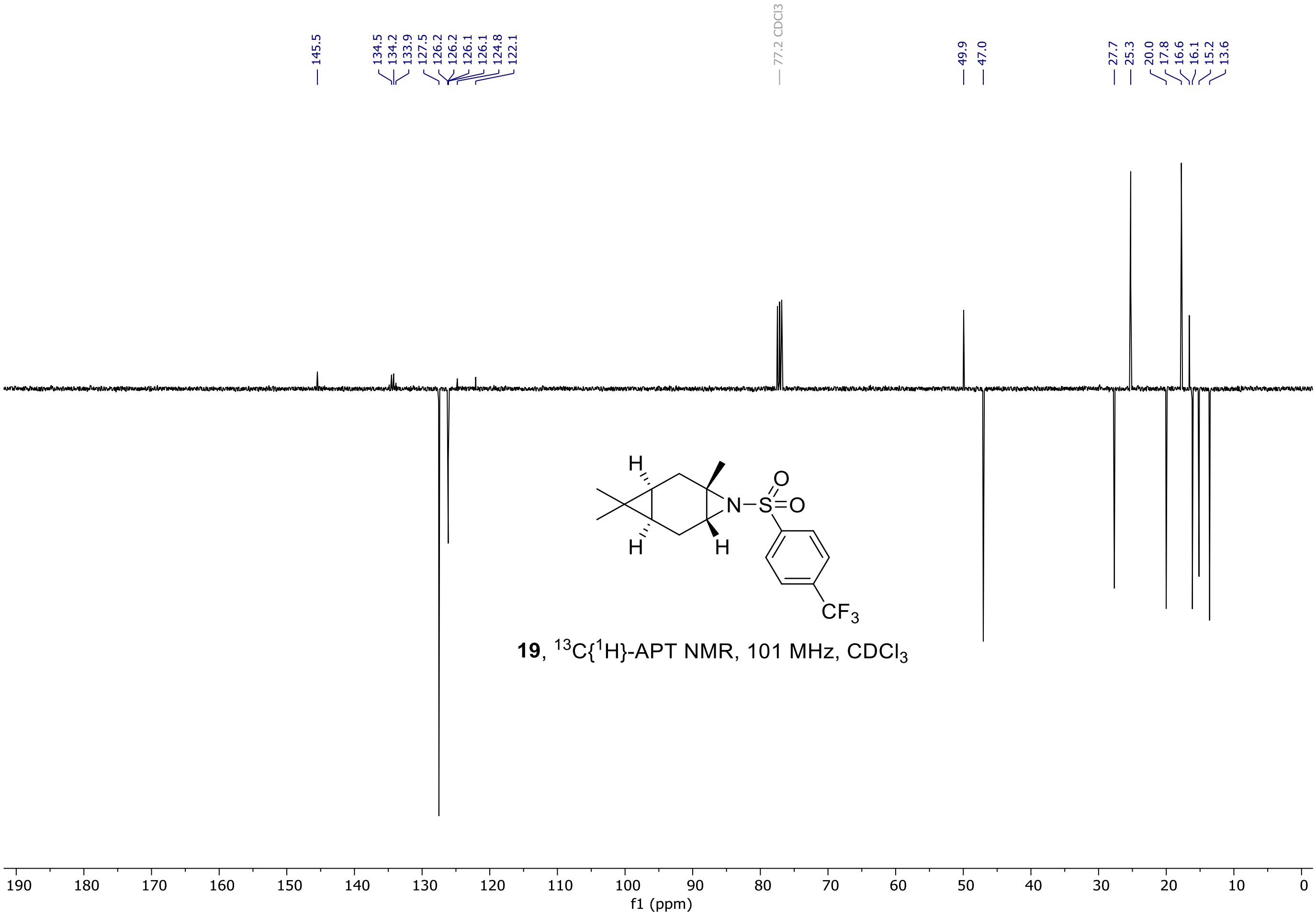

19, ^{13}C{^{1}H}-APT NMR, 101 MHz, $CDCl_3$

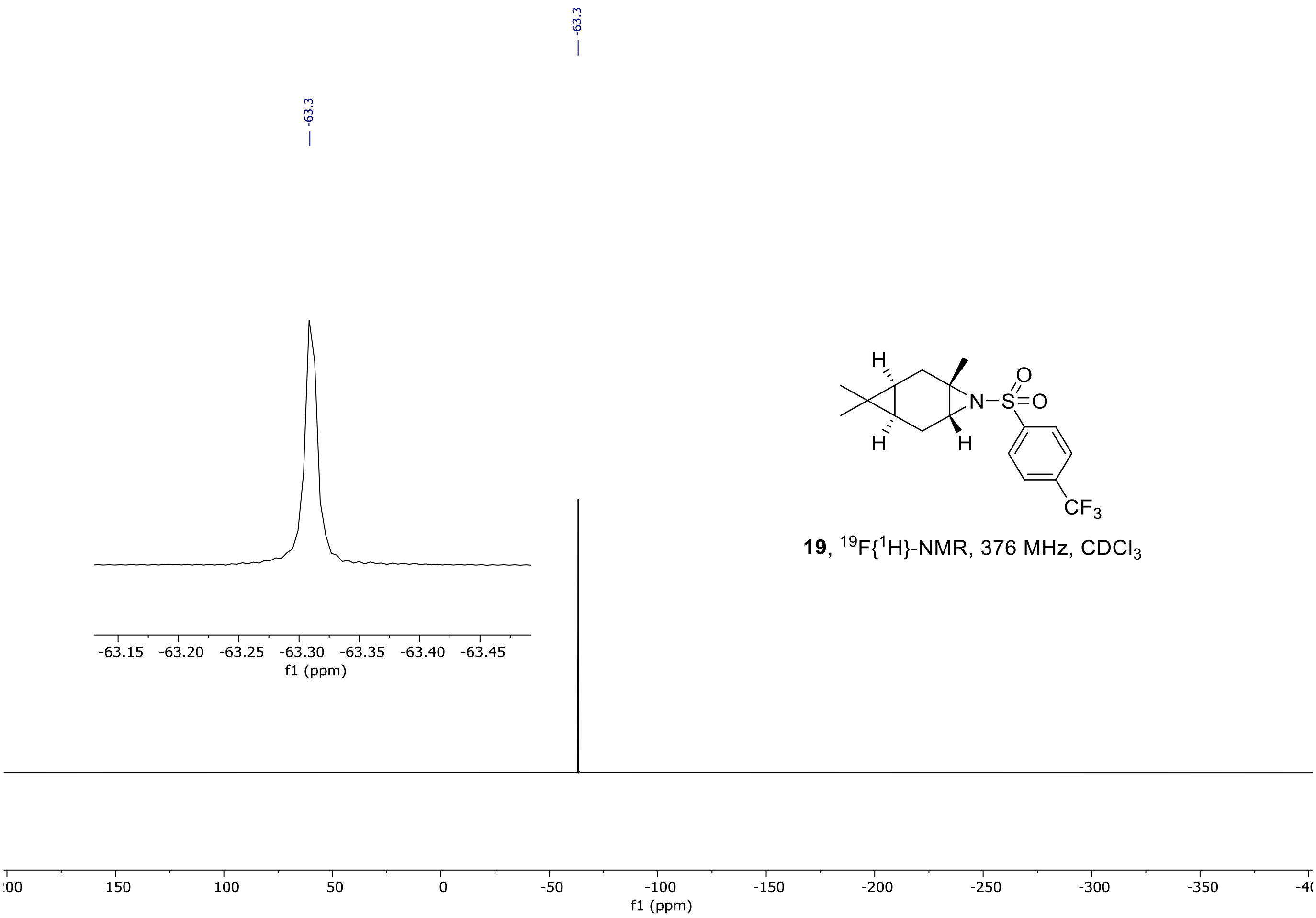
-63.3
-63.3
19, 19F{1H}-NMR, 376 MHz, CDCl3
f1 (ppm)
CF3

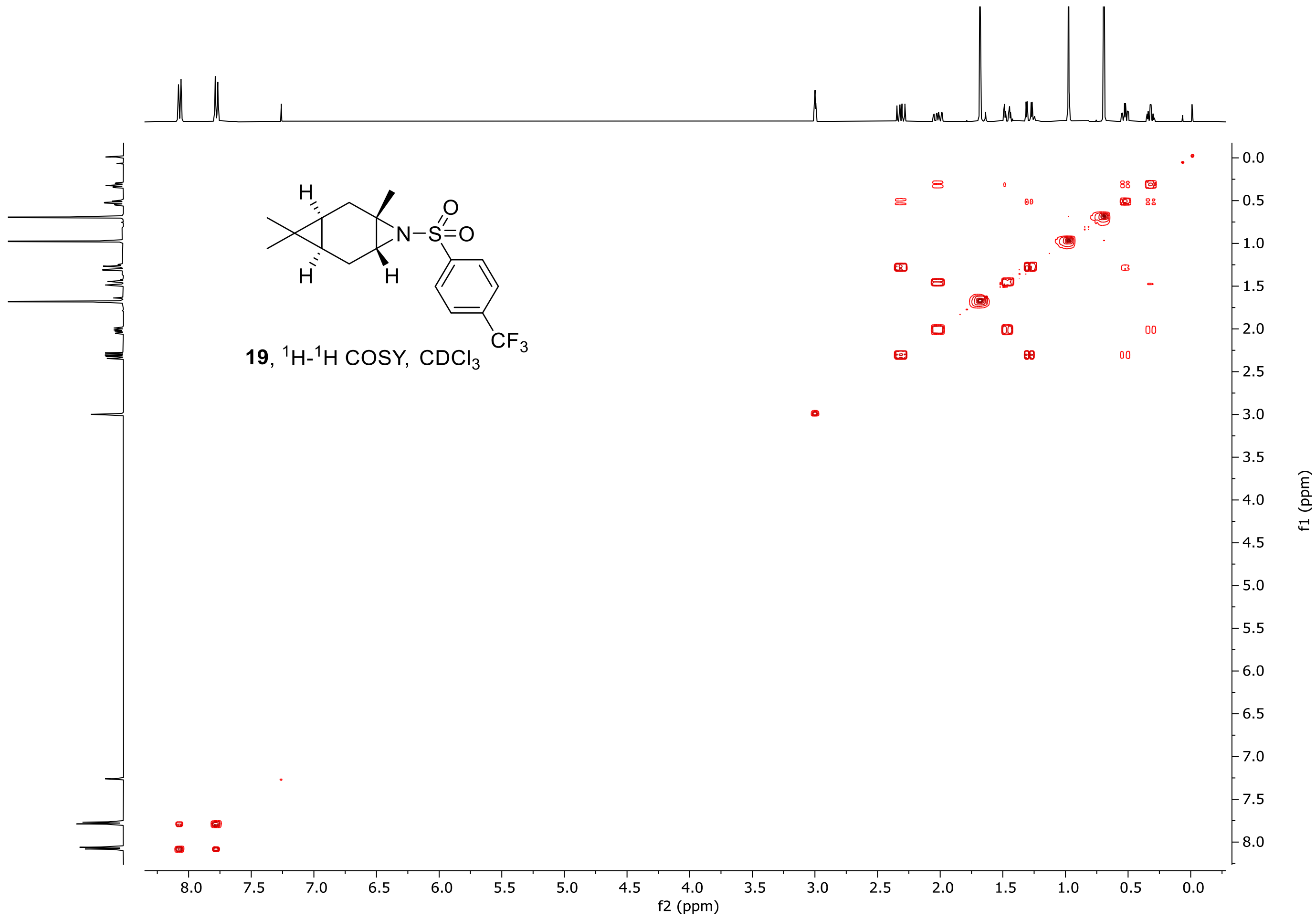
19, 1H-1H COSY, CDCl3
f2 (ppm)
f1 (ppm)
CF3

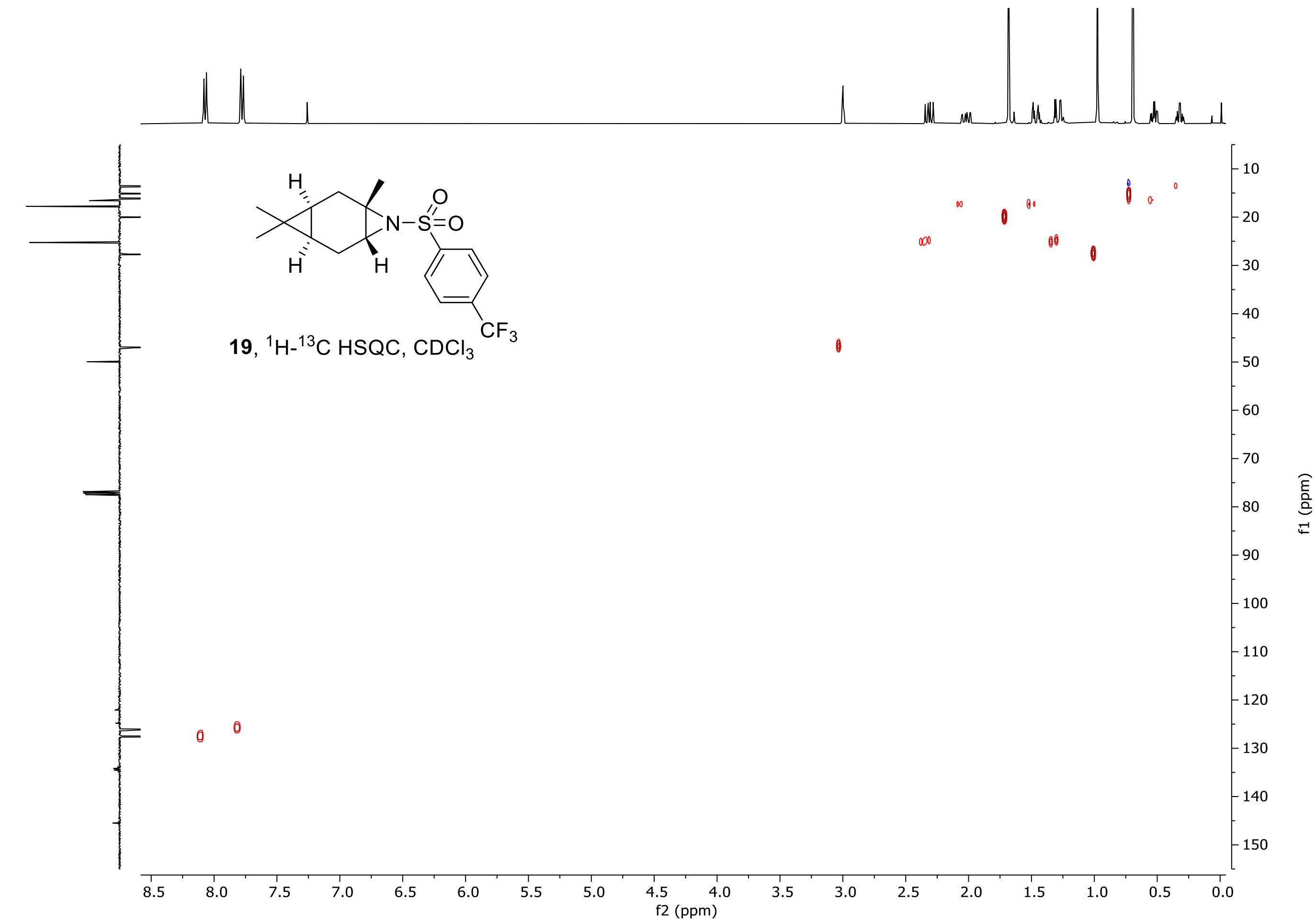
19, ^{1}H-^{13}C HSQC, $CDCl_3$
f2 (ppm)
f1 (ppm)

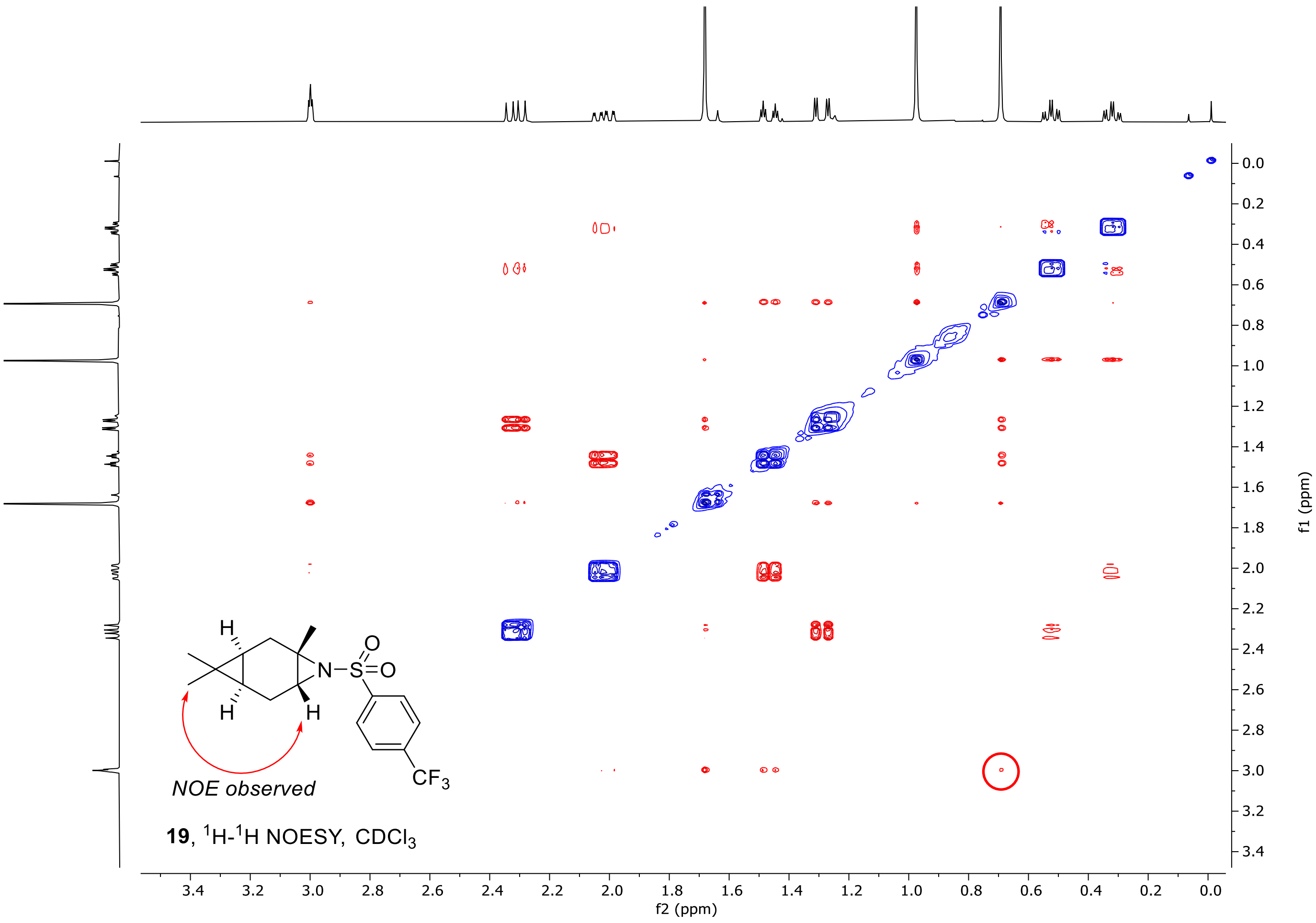
NOE observed
19, ^{1}H-^{1}H NOESY, $CDCl_3$
f2 (ppm)
f1 (ppm)

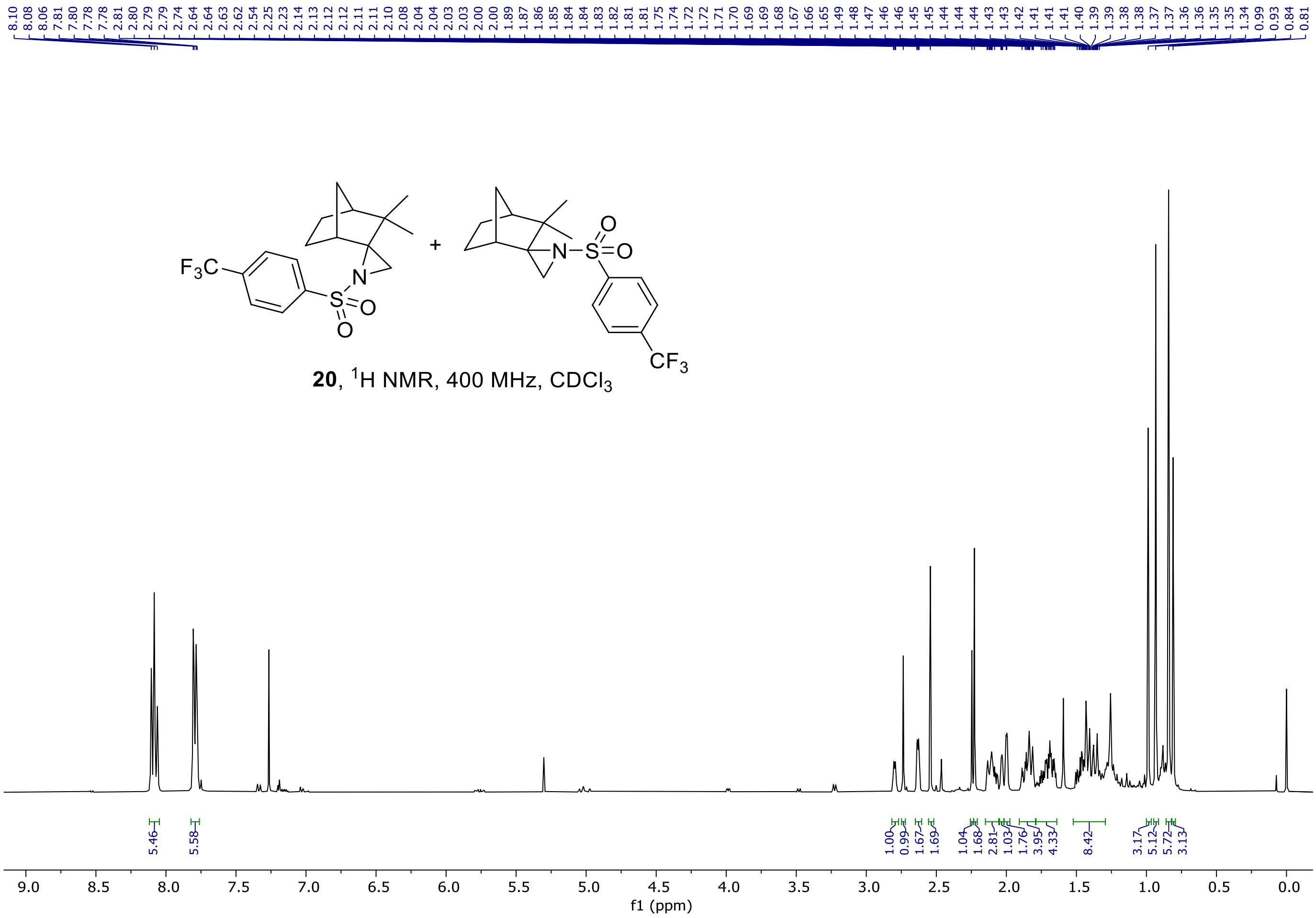
8.10 8.08 8.06 7.81 7.80 7.78 2.81 2.80 2.79 2.79 2.74 2.64 2.64 2.63 2.62 2.54 2.25 2.23 2.14 2.13 2.12 2.12 2.11 2.11 2.10 2.08 2.04 2.04 2.03 2.03 2.00 2.00 1.89 1.87 1.86 1.85 1.84 1.84 1.83 1.82 1.81 1.81 1.75 1.74 1.72 1.72 1.71 1.70 1.69 1.69 1.68 1.67 1.66 1.65 1.49 1.48 1.47 1.46 1.46 1.45 1.45 1.44 1.44 1.44 1.43 1.43 1.42 1.41 1.41 1.41 1.40 1.39 1.39 1.38 1.38 1.37 1.37 1.36 1.36 1.35 1.35 1.34 0.99 0.93 0.84 0.81
F3C
N
S
O
O
+
N
S
O
O
CF3
20, 1H NMR, 400 MHz, CDCl3
5.46 5.58 1.00 0.99 1.67 1.69 1.04 1.68 2.81 1.03 1.76 3.95 4.33 8.42 3.17 5.12 5.72 3.13
9.0 8.5 8.0 7.5 7.0 6.5 6.0 5.5 5.0 4.5 4.0 3.5 3.0 2.5 2.0 1.5 1.0 0.5 0.0
f1 (ppm)

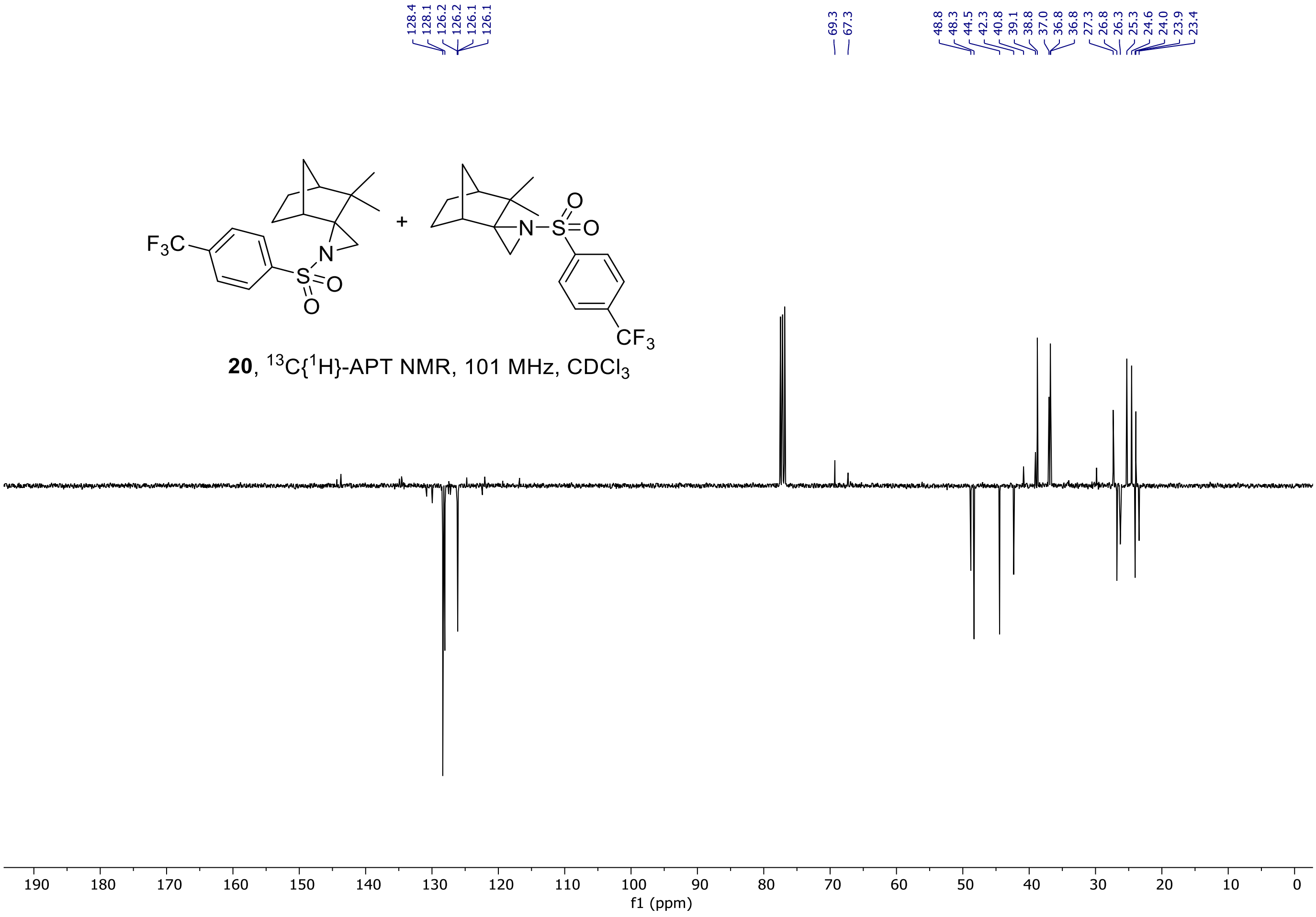
128.4 128.1 126.2 126.2 126.1 126.1
69.3 67.3
48.8 48.3 44.5 42.3 40.8 39.1 38.8 37.0 36.8 27.3 26.8 26.3 25.3 24.6 24.0 23.9 23.4
F3C
N
S
O
O
+
N
S
O
O
CF3
20, 13C{1H}-APT NMR, 101 MHz, CDCl3
190 180 170 160 150 140 130 120 110 100 90 80 70 60 50 40 30 20 10 0
f1 (ppm)

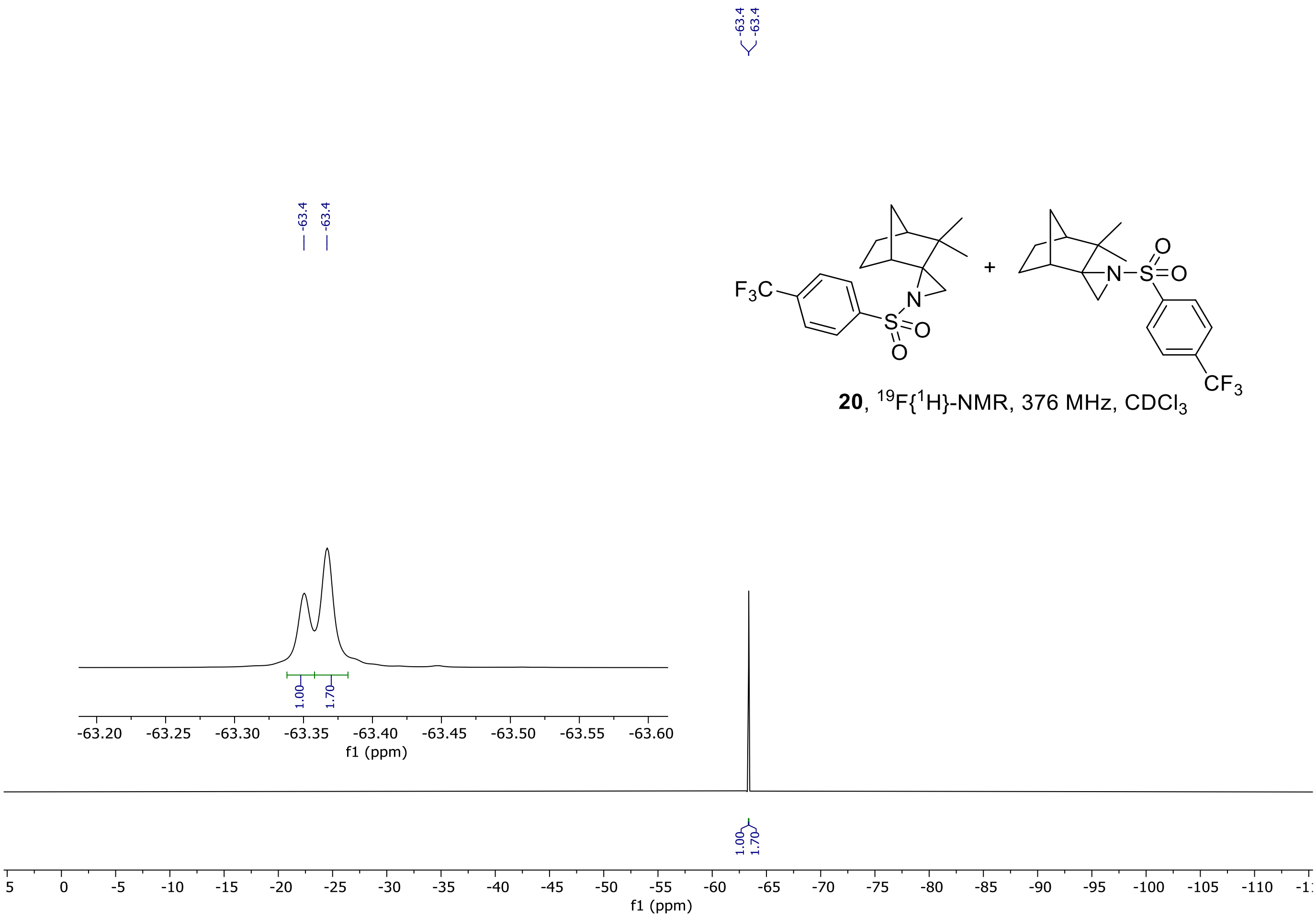

20, $^{19}F\{^{1}H\}$-NMR, 376 MHz, $CDCl_3$

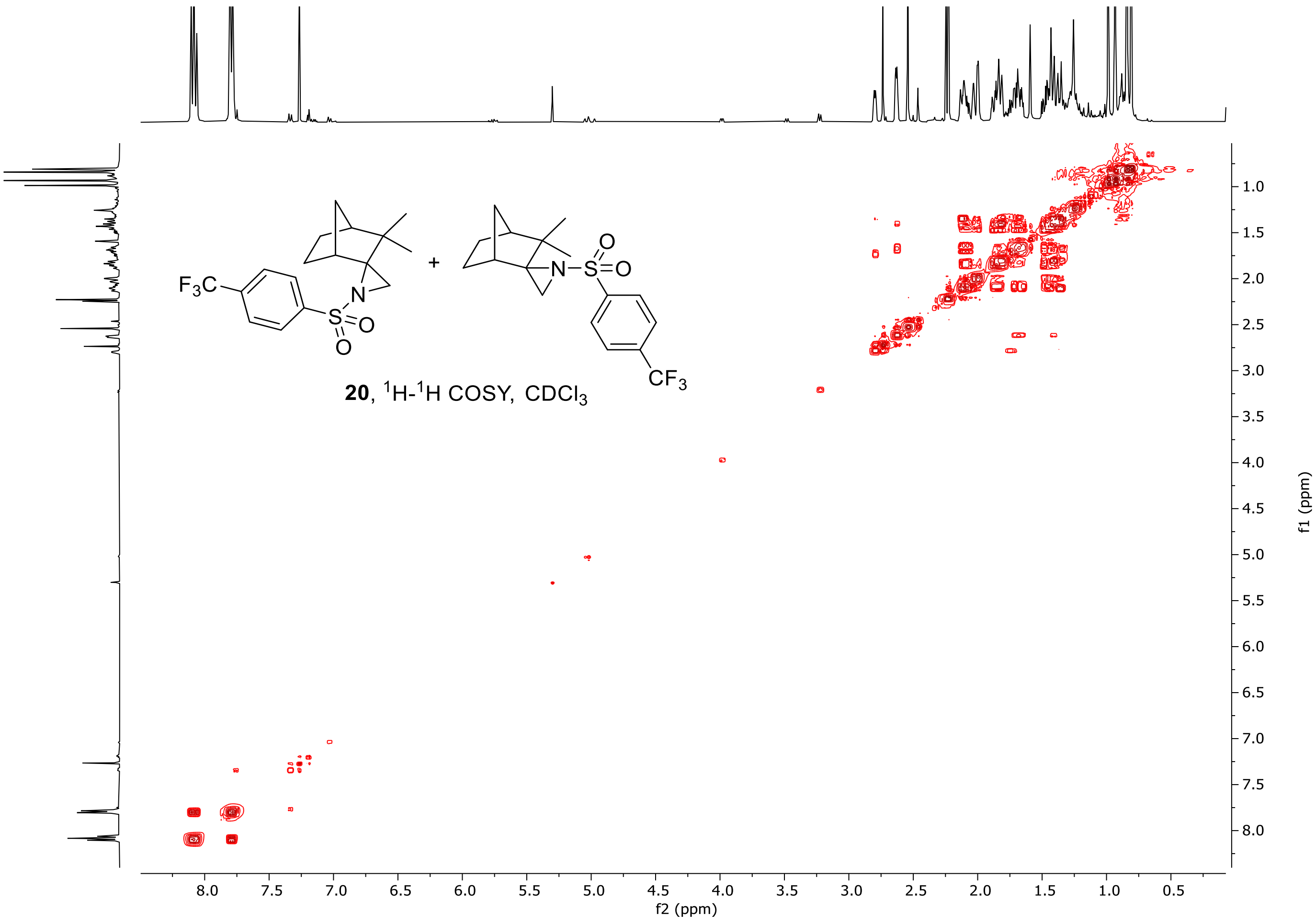

20, ^{1}H-^{1}H COSY, $CDCl_3$

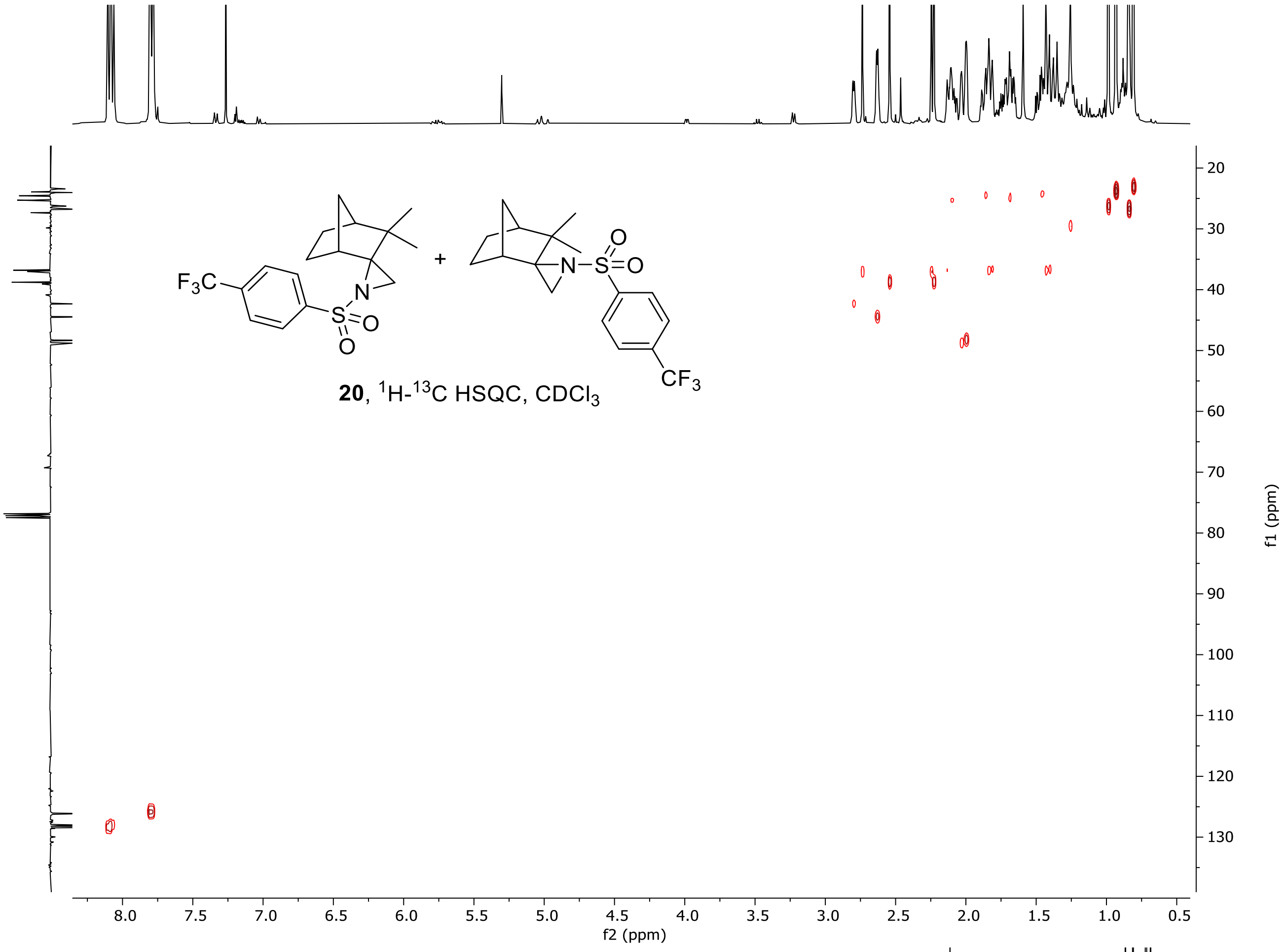
F3C
N
S
O
O
+
N
S
O
O
CF3
20, 1H-13C HSQC, CDCl3
f2 (ppm)
f1 (ppm)

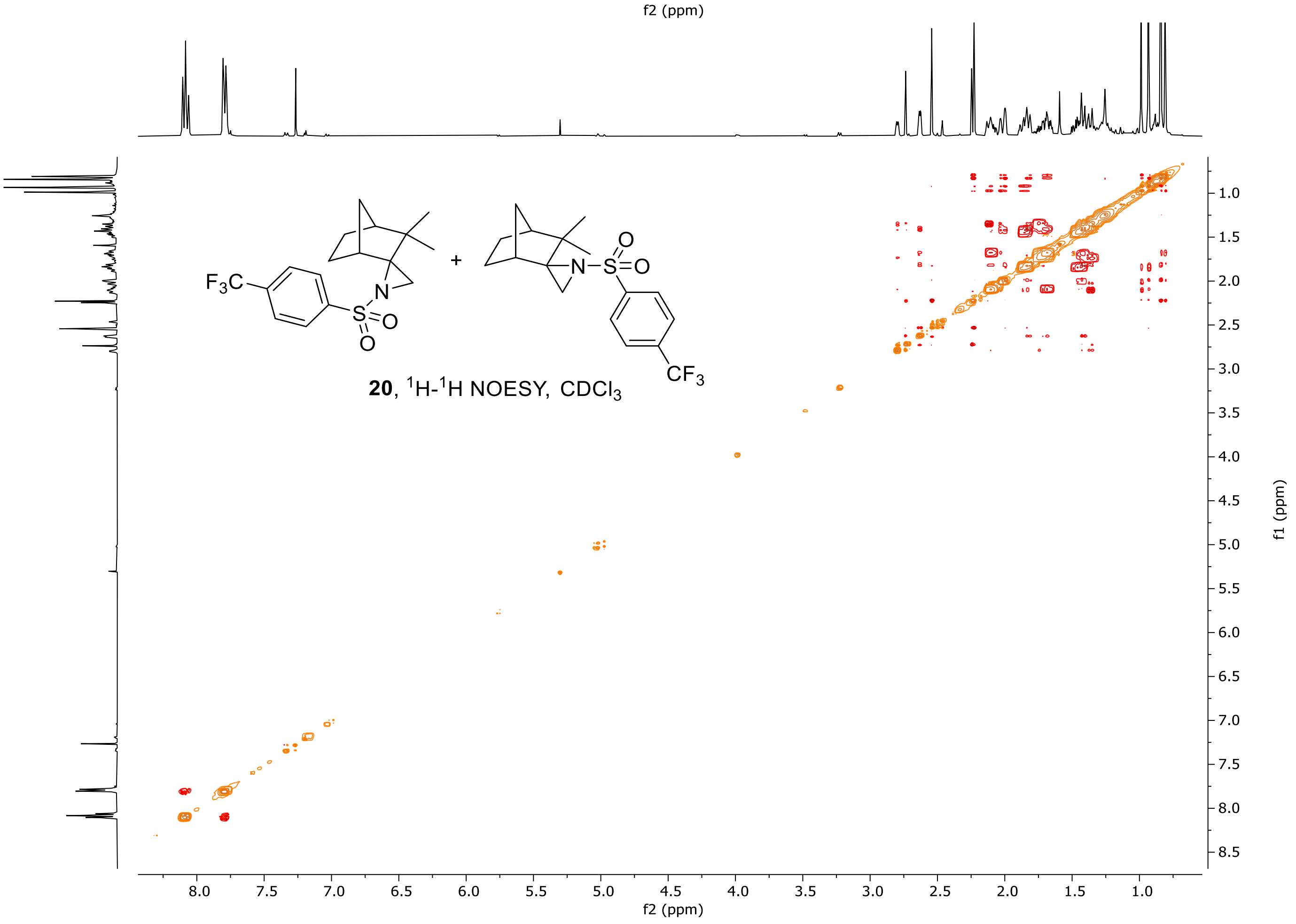
F3C
N
S
O
O
+
N
S
O
O
CF3
20, 1H-1H NOESY, CDCl3
f2 (ppm)
f1 (ppm)

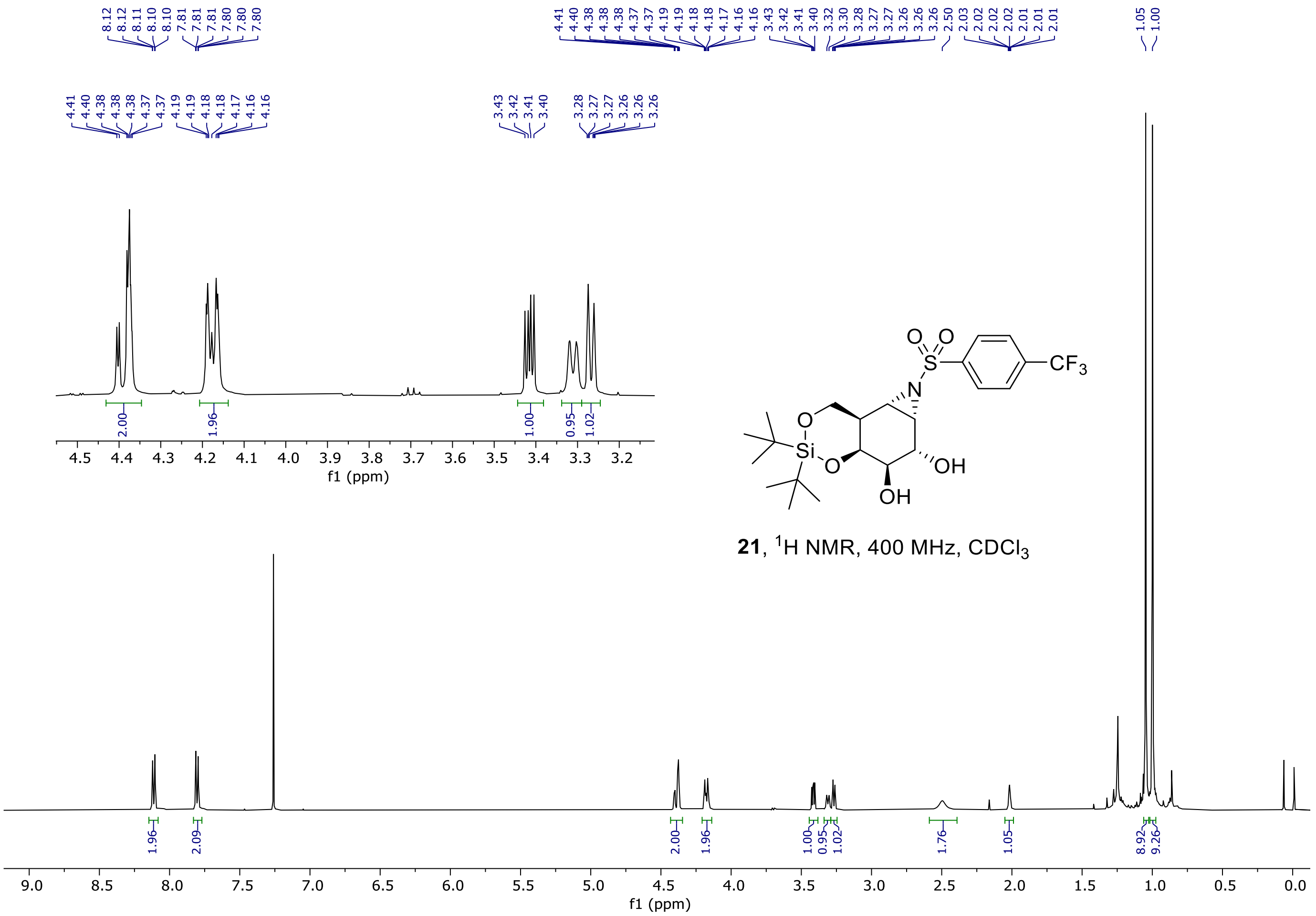

21, ^{1}H NMR, 400 MHz, $CDCl_3$

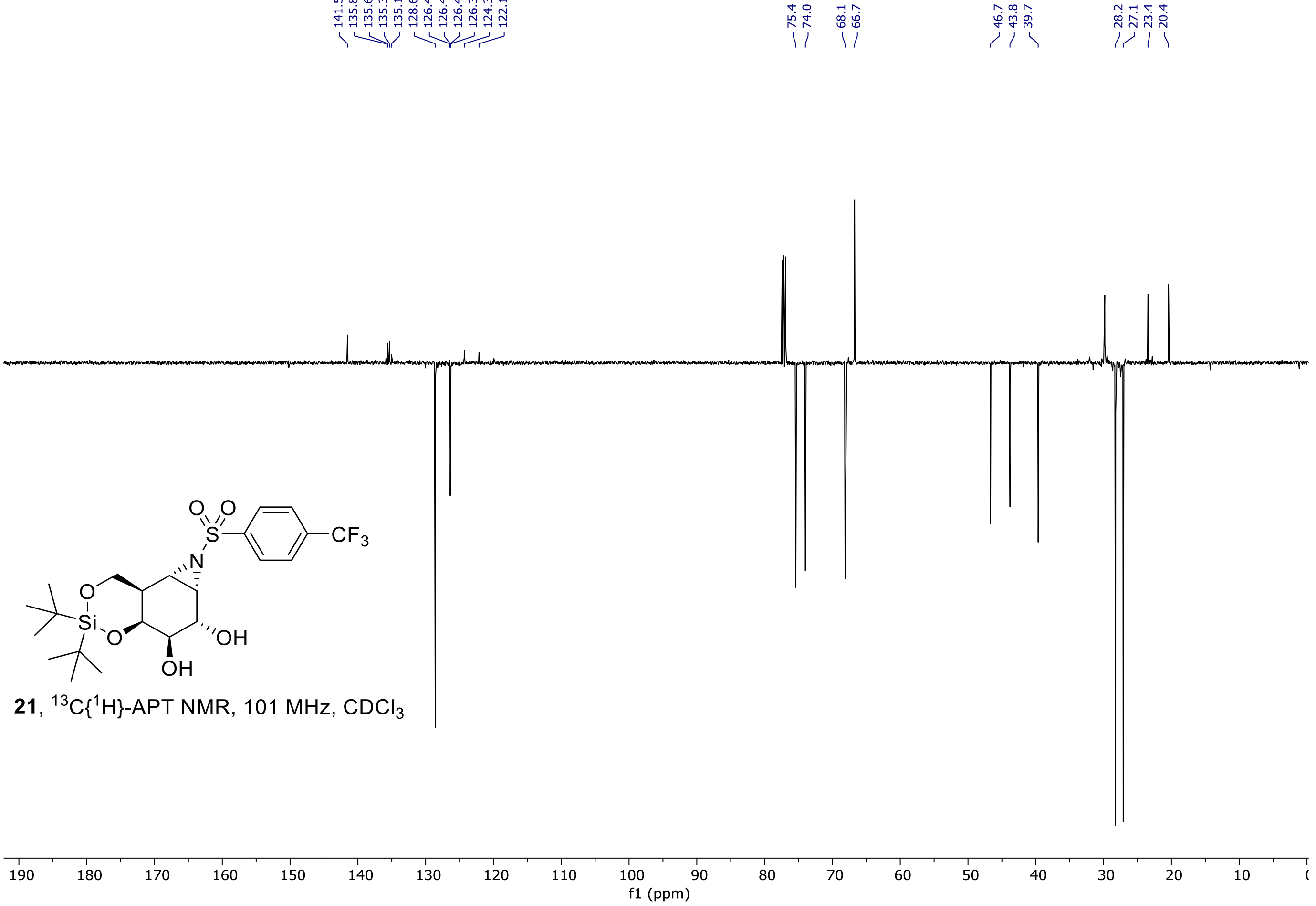

21, ^{13}C{^{1}H}-APT NMR, 101 MHz, $CDCl_3$

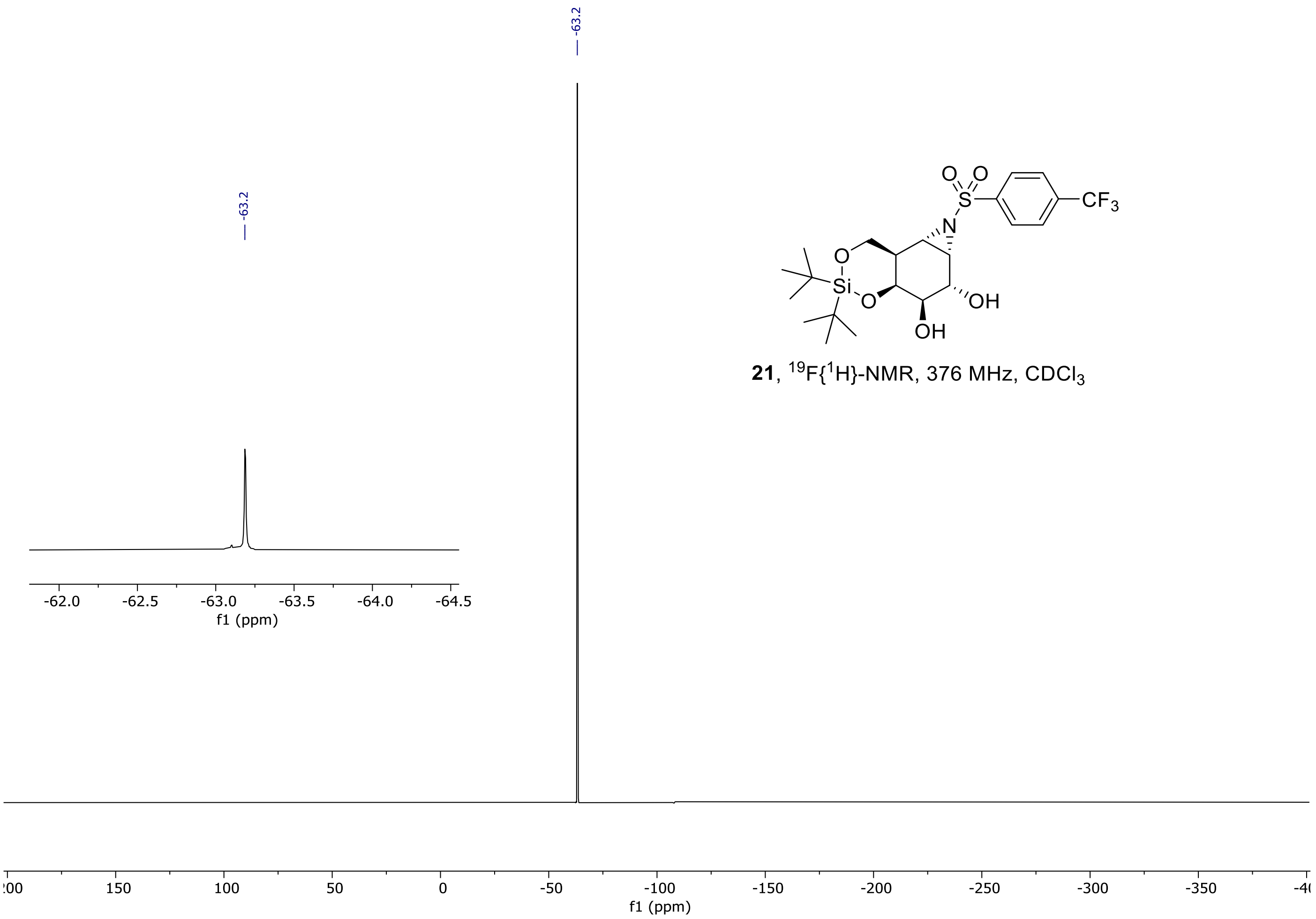

21, $^{19}F\{^{1}H\}$-NMR, 376 MHz, $CDCl_3$

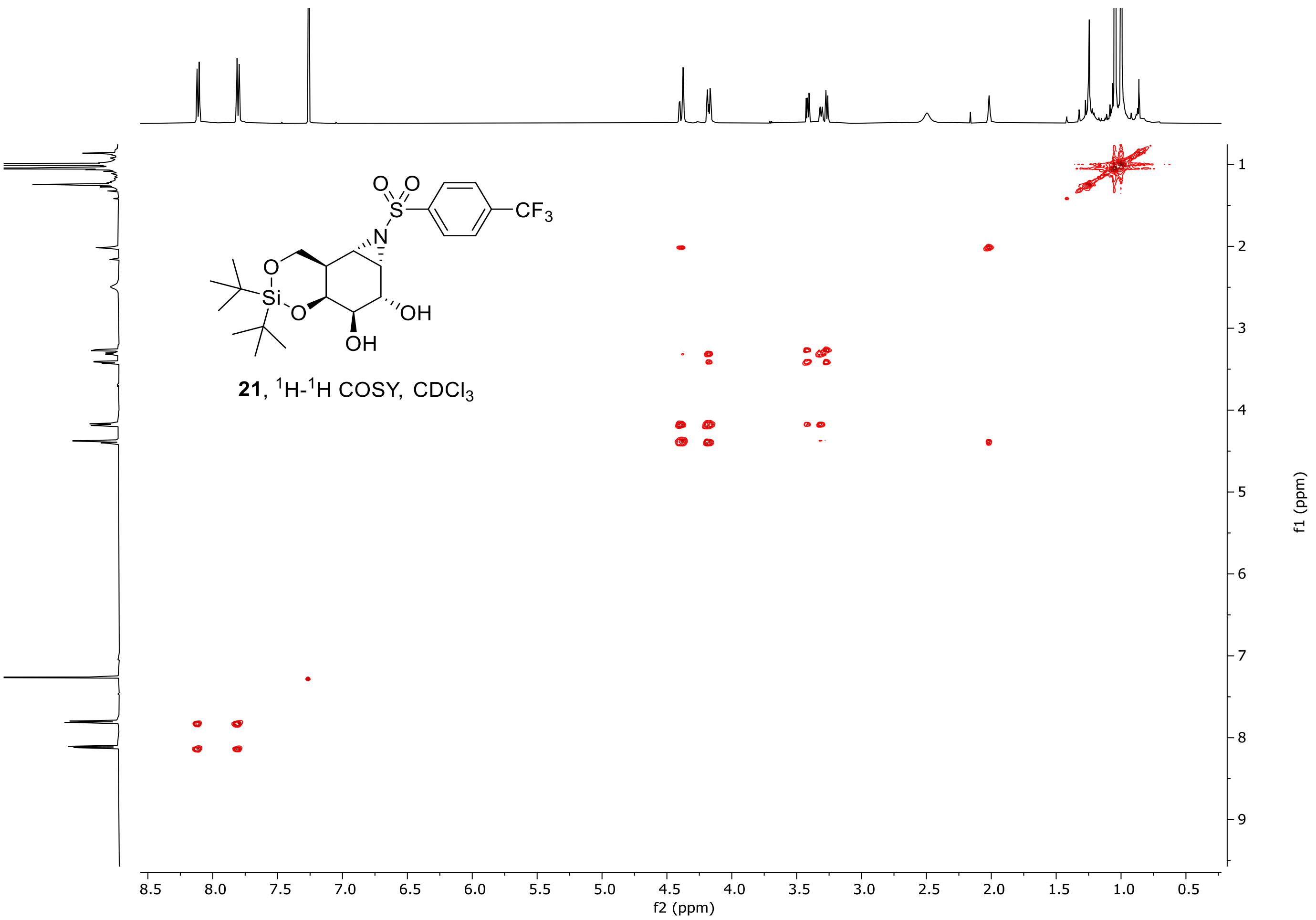

21, ^{1}H-^{1}H COSY, $CDCl_3$

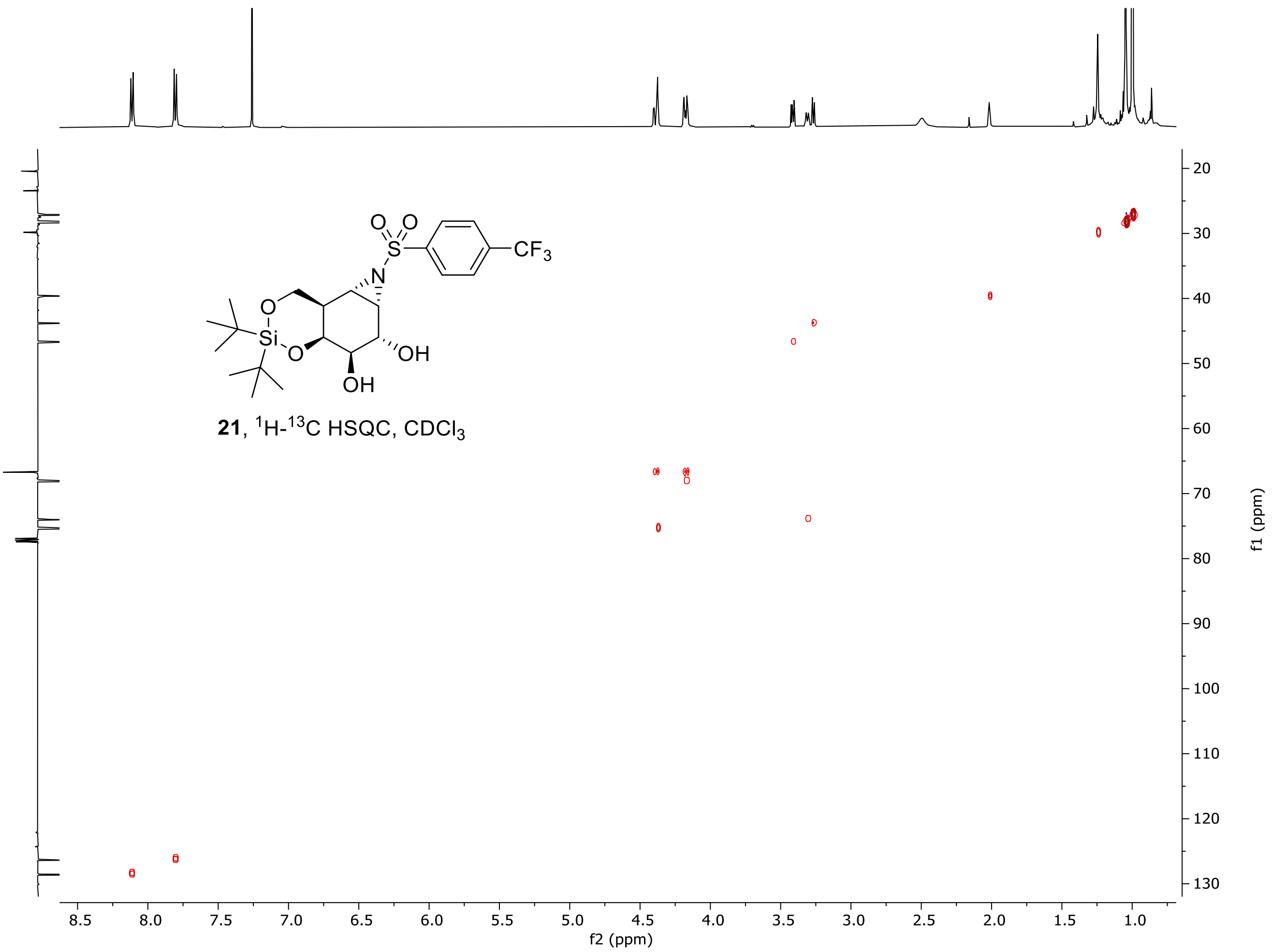
21, ^{1}H-^{13}C HSQC, CDCl$_3$
f2 (ppm)
f1 (ppm)

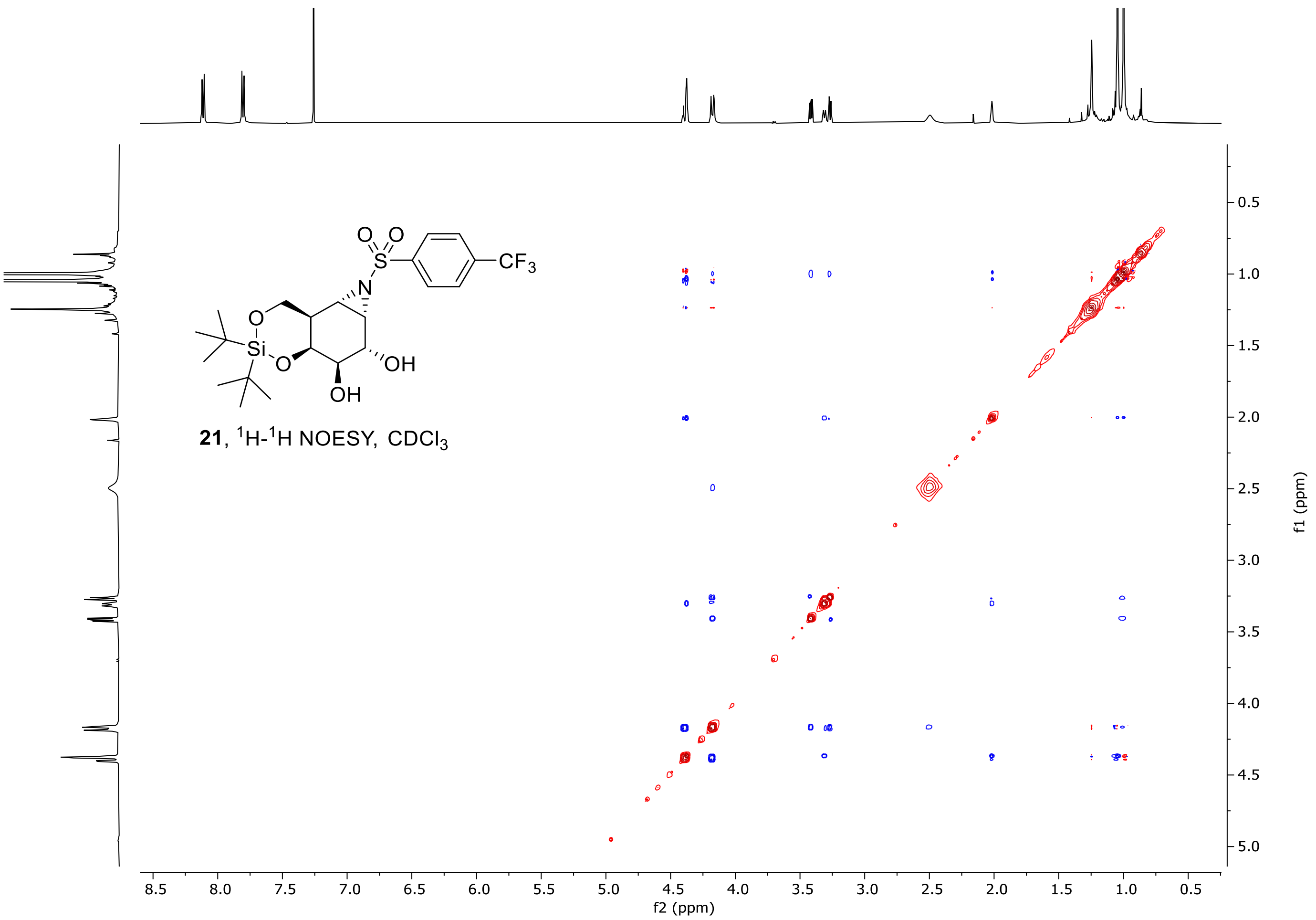
21, ^{1}H-^{1}H NOESY, CDCl$_3$
f2 (ppm)
f1 (ppm)
